# Tuning the Triazolotriazine Scaffold Toward Selective A_3_ Adenosine Receptor Antagonists: From Molecular Recognition to Neuroprotection

**DOI:** 10.1002/cmdc.70528

**Published:** 2026-10-18

**Authors:** Veronika Kost, Filippo Prencipe, Federica Panozzi, Veronica Salmaso, Sonja Kachler, Karl‐Norbert Klotz, Elisabetta Coppi, Simone Adorinni, Stefano Moro, Anna Maria Pugliese, Stephanie Federico, Giampiero Spalluto, Tatiana Da Ros

**Affiliations:** ^1^ Department of Chemical and Pharmaceutical Sciences University of Trieste Trieste Italy; ^2^ Department of Neuroscience, Psychology Drug Research and Child Health (NEUROFARBA) University of Florence Florence Italy; ^3^ Molecular Modeling Section (MMS) Department of Pharmaceutical and Pharmacological Sciences University of Padua Padua Italy; ^4^ Rudolf‐Virchow‐Zentrum – Center for Integrative and Translational Bioimaging University of Würzburg Würzburg Germany; ^5^ Institut für Pharmakologie und Toxikologie University of Würzburg Würzburg Germany

**Keywords:** A_3_ adenosine receptor, adenosine receptors, G protein‐coupled receptor (GPCR) antagonists, ischemia, oxygen glucose deprivation (OGD)

## Abstract

A series of 2,5,7‐trisubstituted [1,2,4]triazolo[1,5‐*a*][1,3,5]triazines (TTs) was developed to identify selective A_3_ AR antagonists. Guided by previous structure–activity relationship studies, the C2‐furyl substituent was replaced with phenyl or 4‐methoxyphenyl moieties to improve A_3_ AR affinity and selectivity. C2 phenyl substitution, together with appropriate C5 and C7 amino substituents, critically modulates receptor recognition. In particular, benzylamino substitution at C5 markedly enhanced A_3_ AR affinity, yielding compound **15** as the most potent antagonist of the series (*K*_i_ = 0.485 nM), whereas incorporation of a 4‐methoxyphenyl group at C2 substantially improved subtype selectivity, exemplified by compound **30** (>2800‐fold selective for A_3_ AR). Molecular docking at the recently determined antagonist‐bound structure of the A_3_ AR rationalized these trends and identified key structural determinants of A_3_ AR preference. The biological relevance of A_3_ AR blockade was investigated in rat hippocampal slices subjected to oxygen‐glucose deprivation. Compound **15** delayed anoxic depolarization and, when administered during ischemia, prevented its onset in most slices and promoted near‐complete recovery of synaptic transmission. These findings identify TT derivatives as promising and highly selective A_3_ AR antagonists with potential neuroprotective applications in cerebral ischemia.

## Graphical abstract

Structure‐based optimization of the triazolo‐triazine scaffold afforded highly potent and selective adenosine A_3_ receptor antagonists. Replacement of the classical 2‐furyl group with phenyl‐based substituents markedly improved A_3_ receptor affinity and selectivity. The lead compound showed subnanomolar affinity and protected hippocampal synaptic function in an in vitro model of cerebral ischemia.

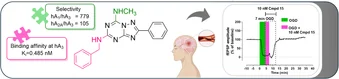

## 1 Introduction

Extracellular adenosine serves as a local modulator exerting a general cytoprotective role in the body [1]. Adenosine is the natural ligand of four G protein‐coupled receptor (GPCR) subtypes, A_1_, A_2A,_ A_2B,_ and A_3_ adenosine receptors (AR) [1, 2], and each of them presents a peculiar pharmacological profile and a specific tissue distribution [2]. Both selective and dual‐target agonists and antagonists have attracted significant pharmacological interest owing to their therapeutic potential, with several compounds already approved for clinical use and many more currently under clinical investigation [3, 4, 5, 6]. This evidence has motivated our research efforts toward the development of novel AR ligands, with particular emphasis on understanding the structure–activity and structure–selectivity relationships that determine receptor affinity and subtype selectivity.

The [1,2,4]triazolo[1,5‐*a* [1,3,5]triazine (TT) scaffold has been extensively investigated in the field of ARs [7, 8, 9, 10, 11, 12, 13, 14]. The most significant achievements have been obtained for the A_2A_ AR subtype, with ZM 241385 (Figure 1) representing the prototypical antagonist and one of the most widely used pharmacological tools for this receptor [1, 21, 22, 23]. In our previous studies, we also identified TT derivatives exhibiting high affinity for the A_1_ and A_3_ AR subtypes [12, 13]; however, receptor subtype selectivity remained generally limited. Notably, all these series shared a 2‐furyl substituent at the C2 position of the TT nucleus. This structural feature was also common to several classes of AR antagonists developed between the 1990s and the early 2000s, including triazolo‐quinazolines (e.g., CGS 15943), triazolo‐pyrazolo‐pyrimidines (e.g., SCH 58261), triazolo‐pyrimidines (e.g., compound **I**) and thiazolo‐pyrimidines (e.g., compound **II**), among others [1, 16, 17].

**FIGURE 1:**
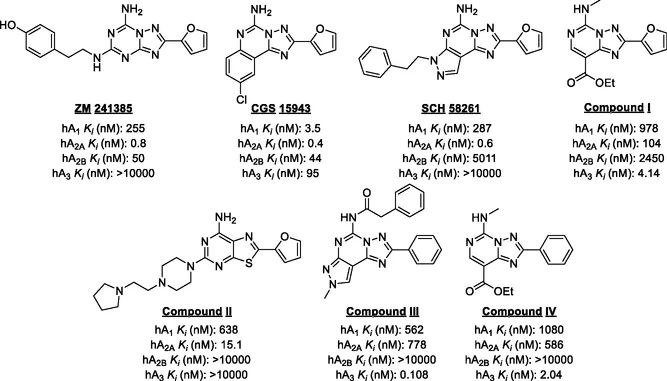
Structures and binding profiles at the four AR subtypes of the reference ligands: ZM 241385 [1, 15], CGS 15943 [1, 15], SCH 58261 [1, 15], compound **I** [16], compound **II** [17, 18], compound **III** [19], compound **IV** [20].

Inspired by the successful replacement of the furan ring with phenyl moieties at the corresponding position of pyrazolo‐triazolo‐pyrimidine (e.g., compound **III**) [19] and triazolo‐pyrimidine (e.g., compound **IV**) scaffolds [20], which, in combination with amino substituents at the C5 position (corresponding to C7 in the TT scaffold), led to enhanced A_3_ AR selectivity, we sought to investigate the impact of analogous modifications within the TT series [19, 20], obtaining compounds **1–30** (see Scheme 1). This strategy would further highlight the remarkable versatility of the TT scaffold [24, 25, 26], whose synthetic accessibility enables the straightforward introduction and fine‐tuning of three independent substitution patterns, making it an attractive platform for the development of selective AR ligands.

**SCHEME 1:**
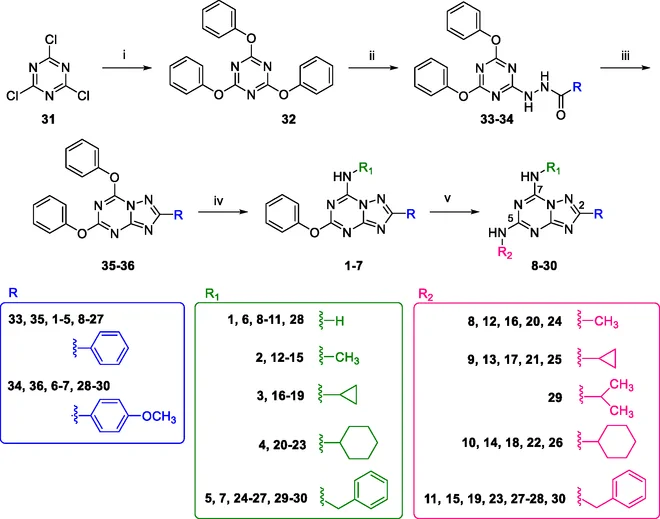
Synthesis of the 2,5,7‐trisubstituted‐[1,2,4]triazolo[1,5‐*a*[1,3,5] triazine nucleus and final compounds **1–30**. Reagents and conditions: (i) PhOH, rfx, 5 h; (ii) required hydrazide, DBU, THF, rt, 12 h; (iii) P_2_O_5_, HMDSO, xylene, from rt to 90 °C, 2–3 h; (iv) MeOH or EtOH, from rt to 90 °C, 3–4 h; (v) EtOH, 100–120 °C, sealed tube, 2–72 h.

Among the AR subtypes, A_3_ AR has emerged as an attractive therapeutic target because of its involvement in inflammation, chronic pain, cancer, and cerebral ischemia [27, 28]. Although its expression in the central nervous system is relatively low under physiological conditions [29, 30], extracellular adenosine accumulation during ischemia is sufficient to engage this low‐affinity receptor subtype [31, 32]. Several studies employing oxygen glucose deprivation (OGD) models have shown that selective A_3_ AR antagonism delays anoxic depolarization (AD) and mitigates ischemia‐induced neuronal dysfunction, supporting the therapeutic potential of A_3_ AR blockers [33, 34, 35]. Therefore, to assess the translational relevance of the newly developed compounds, the lead derivative was evaluated in an OGD model of cerebral ischemia, a well‐established in vitro paradigm that reproduces key metabolic and functional features of ischemic stroke.

## 2 Results and Discussion

### 2.1 Chemistry

The synthetic route adopted for the preparation of the target 2,5,7‐trisubstituted‐[1,2,4]triazolo[1,5‐*a*[1,3,5] triazines **1–30** has been described previously [25, 36, 37] and is outlined in Scheme 1 for completeness. The synthetic procedure began with the commercially available 2,4,6‐trichloro[1,3,5]triazine (**31**), which is converted into 2,4,6‐triphenoxy[1,3,5]triazine (**32**) in phenol at reflux. Compound **32** was reacted with either benzohydrazide or 4‐methoxybenzohydrazide in the presence of 1,8‐diazabicycloundec‐7‐ene (DBU) at room temperature to yield the corresponding *N*′‐(4,6‐diphenoxy‐1,3,5‐triazin‐2‐yl)benzohydrazide (**33**) and *N*′‐(4,6‐diphenoxy‐1,3,5‐triazin‐2‐yl)‐4‐methoxybenzohydrazide (**34**), respectively. Subsequently, the hydrazide intermediates **33** and **34** were subjected to dehydrative cyclization promoted by hexamethyldisiloxane and phosphorus pentoxide in anhydrous xylene at 90 °C to provide the well‐known 5,7‐diphenoxy[1,2,4]triazolo[1,5‐*a*[1,3,5] triazine nucleus, bearing at the C2 position a phenyl (**35**) and a *para*‐methoxyphenyl moiety (**36**), respectively. Finally, from compounds **35** and **36,** two consecutive nucleophilic aromatic substitutions at the C7 and subsequently at the C5 positions of the [1,2,4]triazolo[1,5*‐a*[1,3,5]triazine core in the presence of the appropriate amines and methanol or ethanol as solvents provided the *N* ^5^,*N* ^7^‐substituted final compounds **1–30**. Notably, compounds **1, 6**, **10–11**, and **28** were previously reported by our group in the context of a CK1δ‐focused medicinal chemistry campaign [36, 37]. These compounds were deliberately retained in the present series to establish structure–activity relationship (SAR) continuity and to assess whether structural features associated with CK1δ inactivity could be repurposed toward the current biological target.

### 2.2 SAR Studies

The synthesized compounds (**1–30**) were tested at the human (h) A_1_, A_2A,_ and A_3_ ARs expressed in CHO cells: [^3^H]CCPA (A_1_), [^3^H]NECA (A_2A_), and [^3^H]HEMADO (A_3_) were used as radioligands in binding assays, whereas the activity at the hA_2B_ AR was determined through the measurement of the inhibition of NECA‐stimulated adenylyl cyclase activity. Tables 1 and 2 summarize the receptor‐binding affinities of the synthesized compounds (**1–30**). The derivatives are divided into two main groups, substituted at C2 with a phenyl (**1–5, 8–27,** Table 1) or 4‐methoxyphenyl group (**6–7, 28–30,** Table 2).

**TABLE 1:** Binding and selectivity profiles of synthesized 2‐phenyl‐substituted compounds **1–5**, **8–27**.

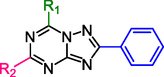								
**n°**	R_1_ (С7)	R_2_ (С5)	hA_1_ *K*_i_, nM^a^	hA_2A_ *K*_i_, nM^b^	hA_2B_ *K*_i_, nM^c^	hA_3_ *K*_i_, nM^d^	hA_1_/hA_3_	hA_2A_/hA_3_
**1**	NH_2_	OPh	517 (393–680)	31.8 (24.4–41.5)	>30,000	182 (150–222)	3	0.17
**8**	NH_2_	NHCH_3_	1880 (1770–1,990)	472 (399–558)	>30,000	1370 (1030–1810)	1	0.34
**9**	NH_2_	NHcC_3_H_5_	1050 (931–1178)	193 (160–232)	>30,000	446 (310–641)	2	0.43
**10**	NH_2_	NHcC_6_H_11_	104 (82.9–132)	35.5 (23.4–53.8)	>30,000	160 (96.9–266)	1	0.22
**11**	NH_2_	NHCH_2_Ph	77.8 (72.8–83.2)	9.15 (6.86–12.2)	>10,000	5.05 (3.19–7.98)	15	2
**2**	NHCH_3_	OPh	>100,000	1430 (1330–1540)	>30,000	10.6 (7.89–14.1)	>9434	135
**12**	NHCH_3_	NHCH_3_	3110 (5950–6270)	3310 (2510–4380)	>30,000	44.7 (33.1–60.5)	70	74
**13**	NHCH_3_	NHcC_3_H_5_	2180 (2030–2350)	1140 (1040–1240)	>30,000	14.8 (12.5–17.6)	147	77
**14**	NHCH_3_	NHcC_6_H_11_	3060 (1670–5610)	397 (314–501)	>30,000	7.63 (6.54–8.89)	401	52
**15**	NHCH_3_	NHCH_2_Ph	378 (326–439)	99.3 (72.0–137)	>30,000	0.485 (0.394–0.598)	779	205
**3**	NHcC_3_H_5_	OPh	462 (431–495)	301 (171–531)	>30,000	13.5 (10.4–17.5)	34	22
**16**	NHcC_3_H_5_	NHCH_3_	559 (459–680)	1300 (722–2350)	>30,000	80.4 (56.9–114)	7	16
**17**	NHcC_3_H_5_	NHcC_3_H_5_	268 (250–289)	630 (522–761)	>10,000	24.8 (16.3–37.7)	11	25
**18**	NHcC_3_H_5_	NHcC_6_H_11_	151 (82–277)	250 (143–438)	>30,000	6.3 (4.91–8.09)	24	40
**19**	NHcC_3_H_5_	NHCH_2_Ph	58.2 (39.5–85.7)	29.1 (24.3–34.7)	>30,000	0.803 (0.629–1.02)	72	36
**4**	NHcC_6_H_11_	OPh	>30,000	>30,000	>30,000	571 (432–756)	>53	>53
**20**	NHcC_6_H_11_	NHCH_3_	321 (240–428)	1740 (1400–2150)	>30,000	345 (252–473)	1	5
**21**	NHcC_6_H_11_	NHcC_3_H_5_	185 (150–228)	1660 (1540–1790)	>30,000	156 (133–181)	1	11
**22**	NHcC_6_H_11_	NHcC_6_H_11_	15,400 (10,000–23,600)	>100,000	>30,000	930 (621–1392)	17	>108
**23**	NHcC_6_H_11_	NHCH_2_Ph	>100,000	>100,000	>30,000	158 (105–239)	>633	>633
**5**	NHCH_2_Ph	OPh	>100,000	>100,000	>30,000	1280 (1120–1470)	>78	>78
**24**	NHCH_2_Ph	NHCH_3_	3590 (3570–3610)	1210 (973–1510)	>30,000	157 (100–244)	23	8
**25**	NHCH_2_Ph	NHcC_3_H_5_	2390 (1920–2980)	881 (759–1020)	>30,000	118 (86.8–159)	20	7
**26**	NHCH_2_Ph	NHcC_6_H_11_	>100,000	55,100 (50,100–60,600)	>30,000	22,800 (15,900–32,800)	>4	2
**27**	NHCH_2_Ph	NHCH_2_Ph	1310 (664–2580)	400 (267–598)	>30,000	44.9 (37.9–53.1)	29	9

a Displacement of [^3^H]CCPA binding at the hA_1_ AR expressed in CHO cells (replicates = 3–6).

b Displacement of [^3^H]NECA binding at the hA_2A_ AR expressed in CHO cells (replicates = 3–6).

c Inhibition of NECA‐stimulated adenylyl cyclase activity in CHO cells expressing the hA_2B_ AR (replicates = 3–6).

d Displacement of [^3^H]HEMADO binding at the hA_3_ AR expressed in CHO cells (replicates = 3–6). *K*_i_ (nM) values are expressed as geometric means, with 95% confidence limits.

**TABLE 2:** Binding and selectivity profiles of synthesized 2‐(4‐methoxy)phenyl‐substituted compounds **6–7**, **28–30**.

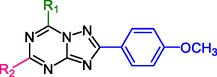								
**n°**	R_1_ (С7)	R_2_ (С5)	hA_1_ a *K*_i_, nM	hA_2A_ b *K*_i_, nM	hA_2B_ c *K*_i_, nM	hA_3_ d *K*_i_, nM	hA_1_/hA_3_	hA_2A_/hA_3_
**6**	NH_2_	OPh	>100,000	>80,000	>30,000	87.8 (61.8–125)	>1139	>911
**28**	NH_2_	NHCH_2_Ph	∼100,000	497 (331–746)	>30,000	1.65 (1.64–1.66)	>60,606	301
**7**	NHCH_2_Ph	OPh	>100,000	>100,000	>30,000	362 (214–613)	>276	>276
**29**	NHCH_2_Ph	NHCH(CH_3_)_2_	12,300 (11,800–12,800)	24,300 (21,600–27,400)	>30,000	71.5 (61.0–83.8)	172	340
**30**	NHCH_2_Ph	NHCH_2_Ph	>100,000	>100,000	>10,000	34.8 (26.4–45.7)	>2874	>2874

a Displacement of [^3^H]CCPA binding at the hA_1_ AR expressed in CHO cells (replicates = 3–6).

b Displacement of [^3^H]NECA binding at the hA_2A_ AR expressed in CHO cells (replicates = 3–6).

c Inhibition of NECA, stimulated adenylyl cyclase activity in CHO cells expressing the hA_2B_ AR (replicates = 3–6).

d Displacement of [^3^H]HEMADO binding at the hA_3_ AR expressed in CHO cells (replicates = 3–6). *K*_i_ (nM) values are expressed as geometric means, with 95% confidence limits.

#### 2.2.1 General Trends at hA_1_, hA_2A_, hA_2B_ ARs

The analysis of the data presented in Tables 1 and 2 reveals that all compounds synthesized in this study exhibited no measurable affinity within the tested concentration range for the hA_2B_ AR subtype. Regarding the hA_1_ AR, the overall affinity across the compound series was low. In particular, the 2‐(4‐methoxy)phenyl derivatives (**6–7 and 28–30**) demonstrated the weakest binding, as indicated by *K*_i_ values predominantly exceeding 100,000 nM. Among all derivatives, the most potent compounds, namely **19**, **11**, and **10**, displayed *K*_i_ values at the hA_1_ AR in the high‐nanomolar range (58.2, 77.8, and 104 nM, respectively). These three compounds also showed relatively good affinity for the hA_2A_ AR, with *K*_i_ values of 29.1 nM (**19**), 9.15 nM (**11**), and 35.5 nM (**10**), alongside compound **1** (31.8 nM). The remaining synthesized derivatives demonstrated substantially lower affinity levels for the hA_2A_ AR, ranging from high nanomolar to several hundred micromolar. Similar to hA_1_ AR binding trends, the compounds bearing a 2‐(4‐methoxy)phenyl moiety at the C2 position (**6–7 and 28–30**) showed the highest *K*_i_ values, indicating minimal affinity for hA_2A_ AR, with only compound **29** displaying a *K*_i_ of 497 nM.

#### 2.2.2 Effect of C2 Substitution on Binding Affinity and Selectivity for hA_3_ AR

In our previous SAR investigation of the [1,2,4]triazolo[1,5‐*a*[1,3,5]triazine scaffold, we established that the presence of a furyl group at the C2 position confers significant affinity for the ARs [13]. However, even with modulation of substitution on the central scaffold, furyl‐substituted derivatives failed to exhibit notable affinity for the hA_3_ AR combined with high selectivity among the other AR subtypes. Taking inspiration from our experience on other nuclei [19, 20], where replacement of the furyl group with a phenyl ring addressed this limitation, we decided to apply the same strategy on the TT scaffold.

The first indication of an improvement in selectivity for hA_3_ AR was observed within the series of compounds substituted at the C7 position with a benzylamino group. Specifically, we examined the binding affinities (*K*_i_) of trisubstituted derivatives bearing identical substituents at C5 (‐OPh) and C7(‐NHCH_2_Ph) while varying the C2 functionality. Both the phenyl and 4‐methoxyphenyl derivatives, **5** (*K*_i_ = 1,280 nM) and **7** (*K*_i_ = 362 nM), exhibited affinity for the hA_3_ receptor in the micro and submicromolar range while showing no binding to other AR subtypes (Table 1). In contrast, the C2 furyl analog (**37**, Table 3) [13] retained comparable affinity for hA_3_ (*K*_i_ = 375 nM) but demonstrated reduced selectivity (hA_2A_/hA_3_ = 1.49). Thus, in the latter subgroup of C2‐substituted derivatives, the selectivity for hA_3_ AR grows in the following order: furyl < phenyl < 4‐methoxyphenyl.

**TABLE 3:** Binding and selectivity profiles of reference 2‐furyl‐TT **37–39** [13].

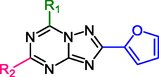					
**n°**	R_1_ (С7)	R_2_ (С5)	hA_1_ *K*_i_, nM	hA_2A_ *K*_i_, nM	hA_3_ *K*_i_, nM
**37**	NHCH_2_Ph	OPh	>100,000	560	375
**38**	NHCH_2_Ph	NHCH_2_Ph	784	20.4	37.0
**39**	NH_2_	NHCH_2_Ph	94.6	1.11	30.8

A similar trend was evident among the 5,7‐bis(benzylamino) derivatives (Figure 2a). While the hA_1_/hA_3_ ratio was comparable for Ph‐ (**27**)‐ and Fu‐ (**38**)‐substituted compounds, the hA_2A_/hA_3_ values showed that the 2‐phenyl derivative (**27**) is 16 times more selective than the furyl one (**38**, 9 versus 0.55 fold [13], respectively). Notably, the introduction of a 4‐methoxyphenyl group at the C2 position (**30**) yielded an outstanding >2874 fold selectivity for hA_3_ AR over both hA_1_ and hA_2A_ AR subtypes, thereby identifying compound **30** as the most selective hA_3_ AR antagonist within the triazolo‐triazine scaffold. Regarding the binding affinity, all three compounds in the series demonstrated *K*_i_ values in the tens‐of‐nanomolar range (Figure 2a), with **30** being the most potent among them, with *K*_i_ = 34.8 nM for hA_3_ AR.

**FIGURE 2:**
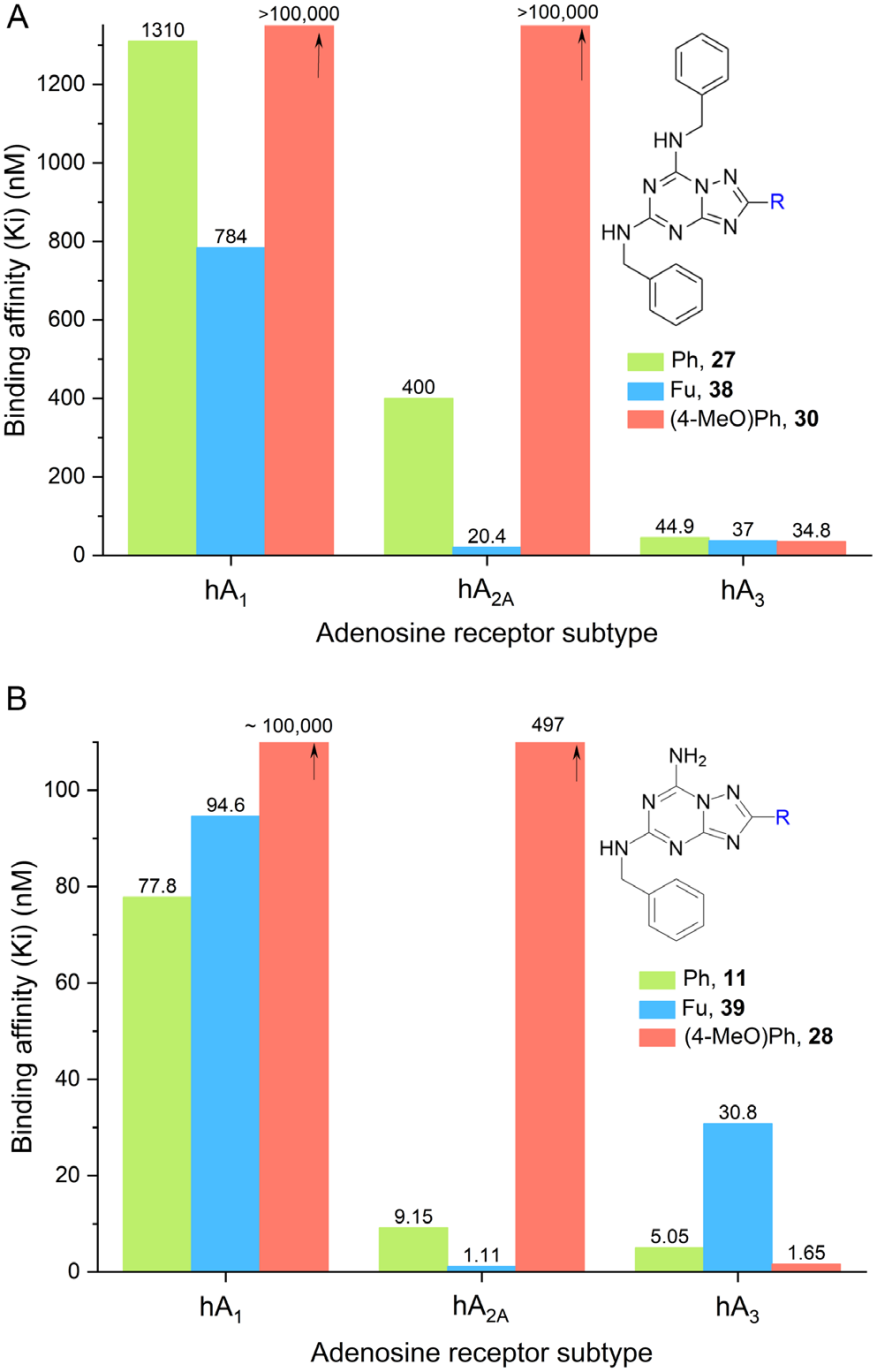
Binding affinities (*K*_i_, nM) to ARs for (A) **27** and **30**, (B) **11** and **28**, bearing Ph and (4‐MeO)Ph groups at the C2 position, compared to the corresponding furyl analogs **38** and **39**. The *K*_i_ values for 2‐Fu derivatives were collected from [13] and shown for comparison.

The pronounced effect of the 4‐methoxyphenyl group on both affinity and selectivity for the hA_3_ AR is further confirmed by the results obtained for the 5,7‐diamine derivatives, which possess a free amino group at C7 and a benzylamino group at the C5 position (Figure 2b). As shown in Table 2, the 4‐methoxyphenyl derivative (**28**) demonstrated a remarkable selectivity, exceeding approximately 60,000‐fold versus hA_1_, and exhibited a 300‐fold higher affinity for hA_3_ AR compared to hA_2A_ AR. Notably, **28** ranks as the third most potent hA_3_ AR antagonist among the synthesized compounds in this study, with a binding affinity, expressed as *K*_i_, of 1.65 nM. An affinity enhancement was also observed for the 2‐phenyl‐substituted 5,7‐diamine (**11**), which showed a hA_3_ AR binding affinity of 5.05 nM, representing more than a sixfold improvement relative to its furyl analog (**39**, Table 3) [13]. Compounds **11** and **28**, with Ph and (4‐MeO)Ph substituents at C2, respectively, were previously evaluated as protein kinase CK1δ inhibitors and were found to be inactive [36].

Within the same structural family (C2: 4‐MeOPh), compounds **7** and **29**, both featuring a benzylamino group at C7, maintained this trend and displayed comparable selectivity levels, ranging from 172‐ to 340‐fold for hA_3_ over other AR subtypes. It is important to note that, except for **28** (*K*_i_ = 1.65 nM), the other selective compounds (**29**, **6**, **23**, and **7**) exhibited relatively low binding affinities for hA_3_, with *K*_i_ values of 71.5, 87.8, 158, and 362 nM, respectively.

#### 2.2.3 Effect of C5 Substitution on hA_3_ AR Binding Affinity

Regarding the triazolo‐triazine scaffold, the type of substituent introduced at the C5 position plays a pivotal role in modulating the affinity of AR antagonists [13]. Based on our previous work on hA_2A_ AR antagonists, the introduction of a benzylamino moiety at the C5 position was a key factor in enhancing affinity toward hA_3_ AR [13]. Indeed, incorporating a ─NHCH_2_Ph group at the C5 position yielded the most potent compound identified in this study, namely, **15**, with a *K*_i_ value of 0.485 nM for hA_3_ AR. The binding affinity evaluation within the subseries of compounds bearing the methylamine moiety at C7 (**2**, **12**–**15**) revealed that these derivatives display *K*_i_ values for hA_3_ in the low‐ to sub‐nanomolar range (Table 1). According to the specific C5 substituent, a clear trend emerged in which binding affinity strengthened in the following order: methylamino < cyclopropylamino < phenoxy < cyclohexylamino < benzylamino group (Figure 3a). Therefore, the benzylamino group at the C5 atom was considered promising for binding affinity to hA_3_ AR, and we consequently compared a subseries of compounds bearing this moiety at C5 with varying C7 functionality. Thus, as shown in Figure 3b, in addition to the methylamino derivative (**15**), the incorporation of either a cyclopropylamino group (**19**, *K*_i_ = 0.803 nM) or a free amino group (**11**, *K*_i_ = 5.05 nM) at C7 also confers sub‐ and low‐nanomolar binding affinity for the hA_3_ adenosine receptor subtype. Notably, this subseries contained the two most potent hA_3_ AR antagonists identified in this study, both of which possessed a ─NHCH_2_Ph group at C5 and were classified as 2‐phenyl derivatives.

**FIGURE 3:**
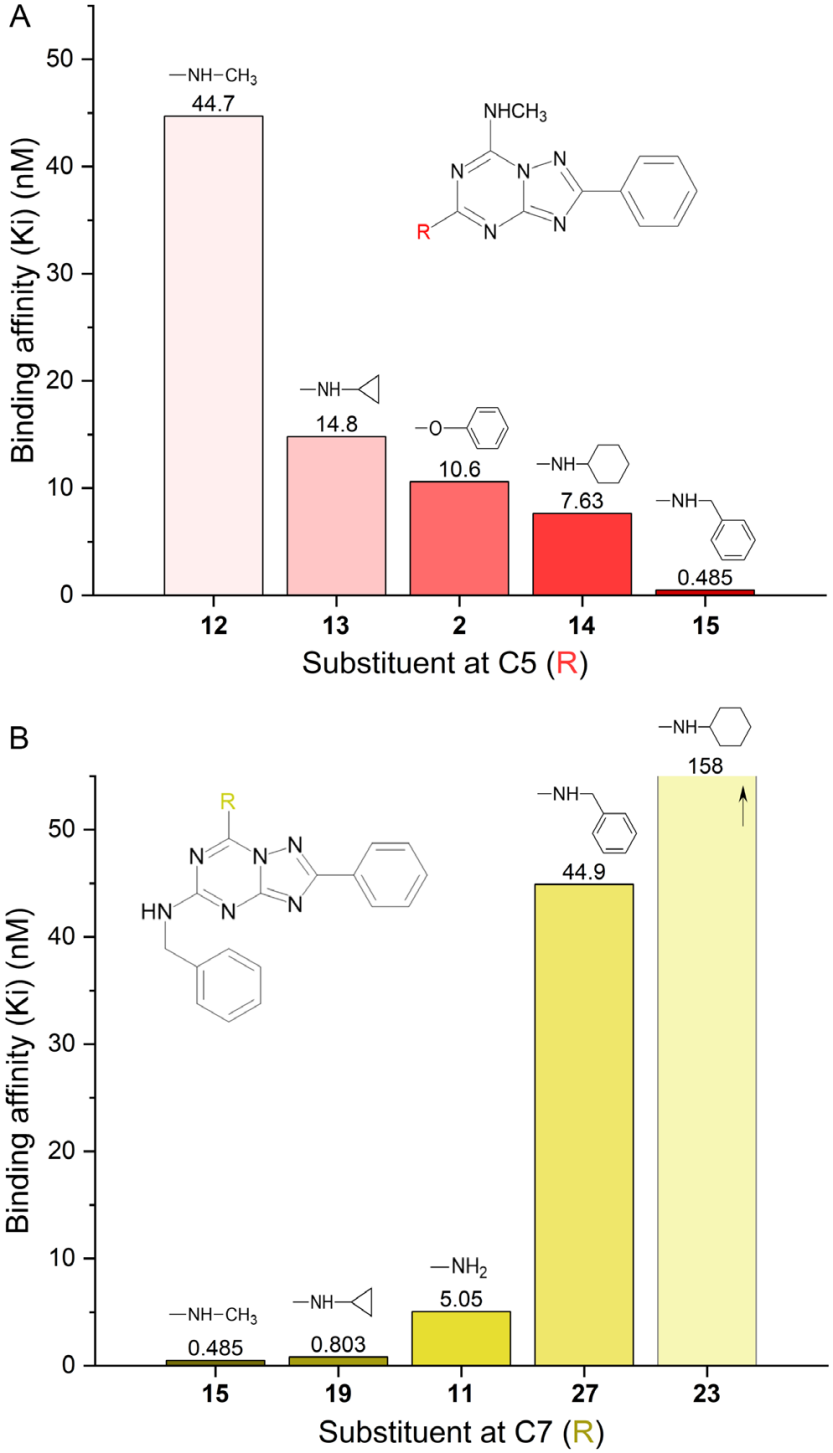
Binding affinities (*K*_i_, nM) to hA_3_ ARs for (A) *N* ^7^‐methyl‐5‐substituted‐2‐phenyl‐[1,2,4]triazolo[1,5‐*a*[1,3,5] triazine‐7‐amines **2, 12–15**, (B) *N* ^5^‐benzyl‐*N* ^7^‐substituted‐2‐phenyl‐[1,2,4]triazolo[1,5‐*a*[1,3,5] triazine‐5,7‐diamines **11**, **15, 19, 23, 27**; Ph group at C2 position.

#### 2.2.4 Effect of C7 Substitution on Selectivity for hA_3_ AR

While C5 substitution primarily accounts for compound affinity, the nature of the functional group at C7 seems to contribute to selectivity [13]. Analyzing the binding affinity ratios with respect to hA_3_ AR in the 2‐phenyl series (Table 1), we observed that all compounds displaying notable selectivity (>100‐fold) consistently possessed a methylamino moiety at C7 (**2** and **15**). In detail, compound **2** showed a *K*_i_ of 10.6 nM and exceptional selectivity (>9434‐fold) for hA_3_ AR over hA_1_ AR, with a hA_2A_/hA_3_ value of 135‐fold. Furthermore, the most potent hA_3_ AR antagonist, **15** (*K*_i_ = 0.485 nM), proved to be 779‐ and 205‐fold more selective for hA_3_ AR compared to hA_1_ AR and hA_2A_ AR, respectively. The other compounds of the same series (**12–14**) also displayed selectivity greater than 50‐fold toward both hA_1_ AR and hA_2A_ AR. Another interesting C7 series is the cyclohexylamine series, where derivative **23** demonstrates relatively good selectivity for hA_3_ AR, exceeding 633‐fold over both hA_1_ AR and hA_2A_ AR.

### 2.3 Molecular Modeling

Molecular docking was performed in order to provide a structural hypothesis for the recognition of this series of compounds by the three AR subtypes for which binding data were collected. Experimental structures of the receptors in their inactive, antagonist‐bound state were retrieved from the Protein Data Bank (PDB) and used as templates: 5N2S for hA_1_ AR (X‐ray diffraction, 3.3 Å, in complex with the xanthine antagonist PSB36) [38], 5NM4 for hA_2A_ AR (room‐temperature serial crystallography, 1.7 Å) [39], and 9EHS for hA_3_ AR (cryo‐electron microscopy, 2.7 Å, in complex with the covalent antagonist LUF7602) [40]. The recent determination of an antagonist‐bound hA_3_ AR structure is particularly relevant in the present context since the orthosteric site of this subtype could previously be described only through homology models built on hA_2A_ AR templates.

Compounds were docked with GOLD using the ChemScore scoring function [41, 42], and the resulting poses were required to reproduce the two pharmacophoric features shared by essentially all AR antagonist chemotypes, namely, an aromatic moiety engaged in a π–π stacking with Phe171/Phe168/Phe168 in extracellular loop 2 (EL2) and a hydrogen‐bond acceptor in proximity of Asn254/Asn253/Asn250 (hA_1_/hA_2A_/hA_3_ AR numbering, respectively). Since a sizeable fraction of the docking poses was characterized by repulsive (positive) van der Waals interaction energies, indicative of residual steric strain, all poses were locally refined with the induced‐fit refinement stage of the MOE docking protocol, with tethered side chains, and re‐scored with GBVI/WSA dG. Poses still displaying positive van der Waals or electrostatic interaction energies after refinement were discarded, and one representative pose per compound was retained—whenever possible—on the basis of the GBVI/WSA dG score, excluding binding modes inconsistent with the recognition mode established for AR antagonists, i.e., those placing the exocyclic NH groups far from Asn254/Asn253/Asn250 or leaving no hydrophobic substituent deep in the orthosteric cavity. The selected poses at hA_3_, hA_2A_, and hA_1_ ARs are collected in Videos S1, S2, and S3, respectively.

For a large part of the series, two alternative and similarly ranked binding modes were retained at the hA_3_ AR. The most active hA_3_ AR antagonist, compound **15**, bearing a benzylamino group at position C5 and a methylamino group at position C7, is used here as a prototype to describe them. In both cases, the NH at position C5 lies in proximity to Asn250, suggesting a possible hydrogen bond, and the central triazolotriazine scaffold is engaged in a π–π stacking interaction with Phe168. In the first binding mode, which resembles that of the prototypical AR antagonist ZM 241385 (hA_2A_ AR‐selective), the aryl substituent at position C2 is placed deep into the binding pocket, the endocyclic N1 and the exocyclic NH at C7 face Asn250—although an ideal bidentate hydrogen bond is not observed—and the substituent at position C5 points toward transmembrane helix (TM) 2 (Figure 4A). In the alternative binding mode, Asn250 is approached by the exocyclic NH at C7 and by N6 in place of N1, the C5 substituent occupies the bottom of the binding pocket, and the C2 substituent points toward TM2 (Figure 4B).

**FIGURE 4:**
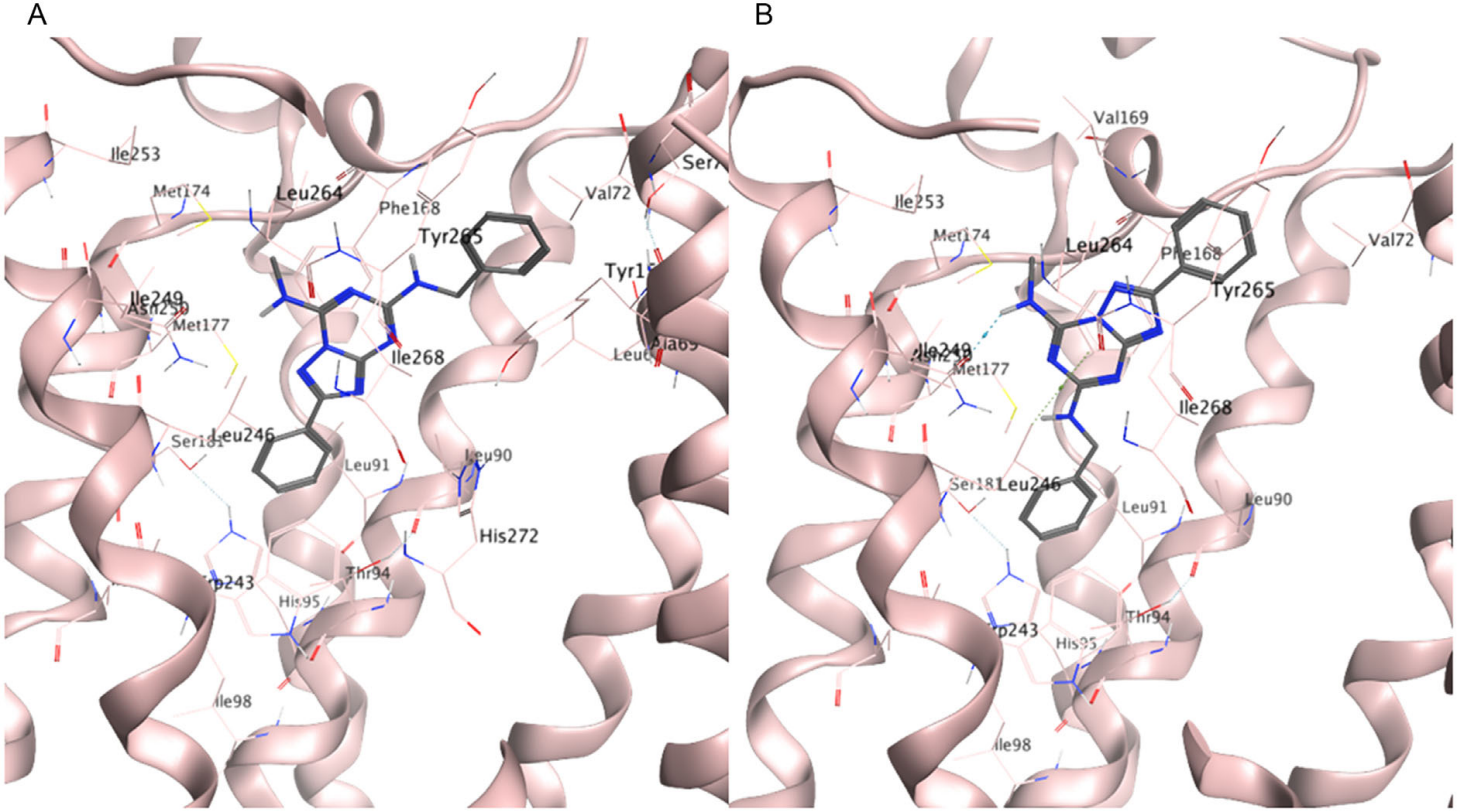
Predicted binding modes of compound **15** (gray) within the orthosteric site of hA_3_ AR (PDB ID: 9EHS, pink). (A) ZM 241385‐like pose, with the C2‐phenyl ring directed toward the bottom of the cavity and the C5‐benzylamino group oriented toward TM2. (B) Alternative pose, in which the C5‐benzylamino group occupies the bottom of the cavity while the C2‐phenyl ring points toward TM2/EL2. Polar contacts, where present, are shown as dashed lines.

Both conformations appear to be accessible to most of the compounds in the series. After exclusion of the unreliable poses, the best GBVI/WSA dG pose corresponds to a ZM‐like conformation for compounds **1**, **3**, **4**, **10**, **12**, **14**, **15**, **16**, **18**, **19**, **21**, and **23**, and to the alternative conformation for compounds **2**, **6**, **7**, **9**, **11**, **13**, **17**, **22**, **24**, **25**, **26**, **27**, **28**, and **29**; for compounds **1**, **2**, **6**, **10**, **11**, **13**, **15**, **18**, **19**, **23**, and **28**, both binding modes were retained among the acceptable poses. The fact that the two orientations are ranked almost equally by the scoring function and that they essentially differ in the exchange of the two aromatic substituents between the deep and the outer region of the cavity indicates that the present docking protocol is not able to discriminate between them.

The per‐residue decomposition of the ligand–receptor interaction energy (Figure 5) identifies Asn250 as the dominant electrostatic contributor across the whole series, in agreement with its established role as the hydrogen‐bonding anchor of AR antagonists. His272 provides a second, generally favorable electrostatic contribution, whose sign is, however, inverted for a subset of derivatives, indicating that this contact is more sensitive than the former to the orientation adopted by the exocyclic substituents. Weaker but recurrent favorable electrostatic contributions are computed for Ser181 and Trp243 at the bottom of the cavity, whereas the electrostatic term associated with Phe168 is frequently repulsive—a contribution that is largely offset by the fact that Phe168 is, by a wide margin, the strongest hydrophobic contributor of the whole pocket, consistent with the π–π stacking described above. The hydrophobic interaction pattern is remarkably conserved across the series and involves Leu68, Val72, Leu90, Leu91, Phe168, Trp243, Leu246, Ile268, and His272.

**FIGURE 5:**
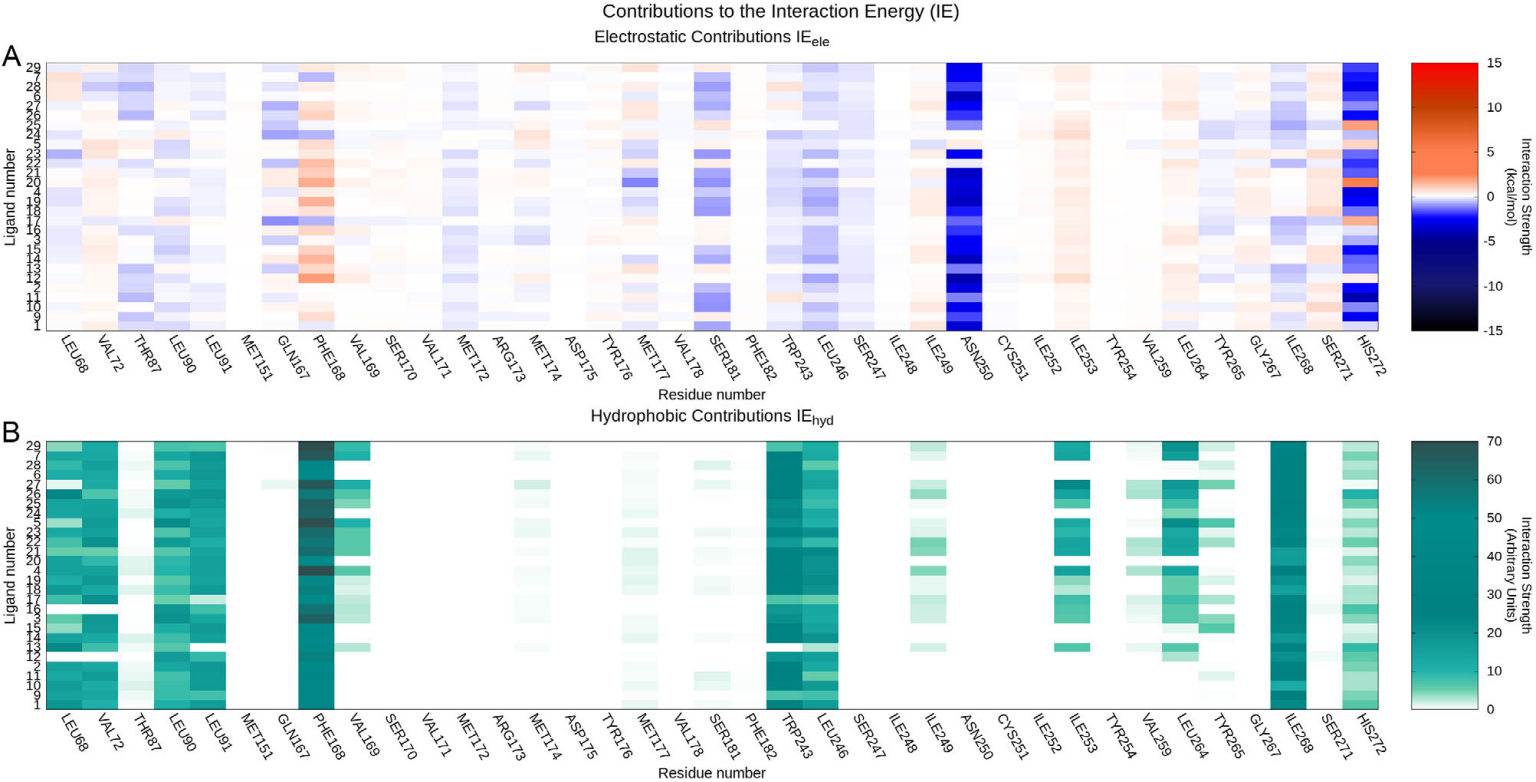
Per‐residue decomposition of the interaction energy between each compound, in its selected pose, and the residues lining the orthosteric site of hA_3_ AR. (A) Electrostatic contributions (IE_ele_, kcal mol^−1^), negative values (blue) denoting favorable interactions. (B) Hydrophobic contributions (IE_hyd_, arbitrary units), larger values (dark green) denoting stronger interactions; note that the two panels adopt opposite sign conventions.

Hydrophobic substituents at position C7 engage additional contacts with Val169, Ile249, Ile253, and Leu264, which line the entrance of the binding cleft between TM6 and TM7. Two of these positions are of particular interest for subtype selectivity: hA_3_ AR is the only subtype bearing a hydrophobic valine (Val169) at the EL2 position occupied by a glutamate conserved in the other AR subtypes (Glu170 in hA_1_ AR and Glu169 in hA_2A_ AR), and it is likewise the only subtype in which position 6.58 (Ballesteros–Weinstein numbering) [43] is occupied by a hydrophobic isoleucine (Ile253) rather than by a conserved threonine (Thr257 in hA_1_ AR, Thr256 in hA_2A_ AR). Both differences make the outer rim of the hA_3_ AR cavity more lipophilic than in the other subtypes and may contribute to the preference of the present uniformly apolar C7 substituents for this receptor. Notably, mutagenesis and molecular dynamics studies have shown that the hA_3_ AR binding pocket can undergo local rearrangements that partially compensate for a Val169Glu mutation and preserve agonist binding, underscoring the structural plasticity of the receptor [44]. Nevertheless, in our docking models, the C7 substituents establish hydrophobic contacts with both Val169 and Ile253(6.58) (Figure 5B), supporting a role for these subtype‐specific residues in shaping the recognition of the present antagonist series.

The systematic improvement of binding affinity provided by the ‐NHCH_2_Ph substitution at position C5 (compounds **11**, **15**, **19**, **23**, and **27**) may be traced to additional contacts with residues on TM7, and in particular with Tyr265 (Figure 5, Video S1). Depending on the binding mode adopted, this region is occupied either by the benzyl group at C5 (ZM‐like conformation) or by the aryl substituent at C2 (alternative conformation), so that in both cases, an aromatic moiety satisfies the same interaction. Notably, mutagenesis studies revealed that substitution of Tyr265 with Ala decreases the affinity in the isoxazole series [45]. Furthermore, Tyr265 is the residue to which the covalent antagonist LUF7602 is anchored in the 9EHS structure [40], which supports the relevance of this subpocket for antagonist recognition at the hA_3_ AR. It should nevertheless be kept in mind that the covalent link itself perturbs the geometry of the LUF7602 complex with respect to the key residues Asn250 and His272 [40], so that the receptor conformation employed here inevitably carries this bias.

At hA_2A_ AR, a ZM‐like binding mode was predicted for the compounds bearing an unsubstituted amino group at position C7 (**1**, **8**–**11**), which are essentially hA_2A_/hA_3_ AR‐unselective (Video S2). In these poses, the NH_2_ group is engaged in hydrogen bonds with Asn253 (TM6) and Glu169 (EL2). Glu169 is in turn involved in an intra‐receptor salt bridge with His264 on EL3, which prevents the analogous ZM‐like placement of derivatives carrying any substituent on the C7 amino group, even one as small as the methyl group of compound **15** (Figure 6B). Alternative conformations of the Glu169–His264 pair are known to be accessible (e.g., PDB ID: 3PWH) [46], but destabilization of this salt bridge has been associated with increased ligand dissociation rates [47], which makes it unlikely that the bulkier members of the present series may be accommodated in a ZM‐like fashion without a kinetic penalty. This offers a structural rationale for the drop in hA_2A_ AR affinity observed as soon as the C7 amino group is substituted (e.g., **1** vs. **2**, **10** vs. **14**, and **11** vs. **15**).

**FIGURE 6:**
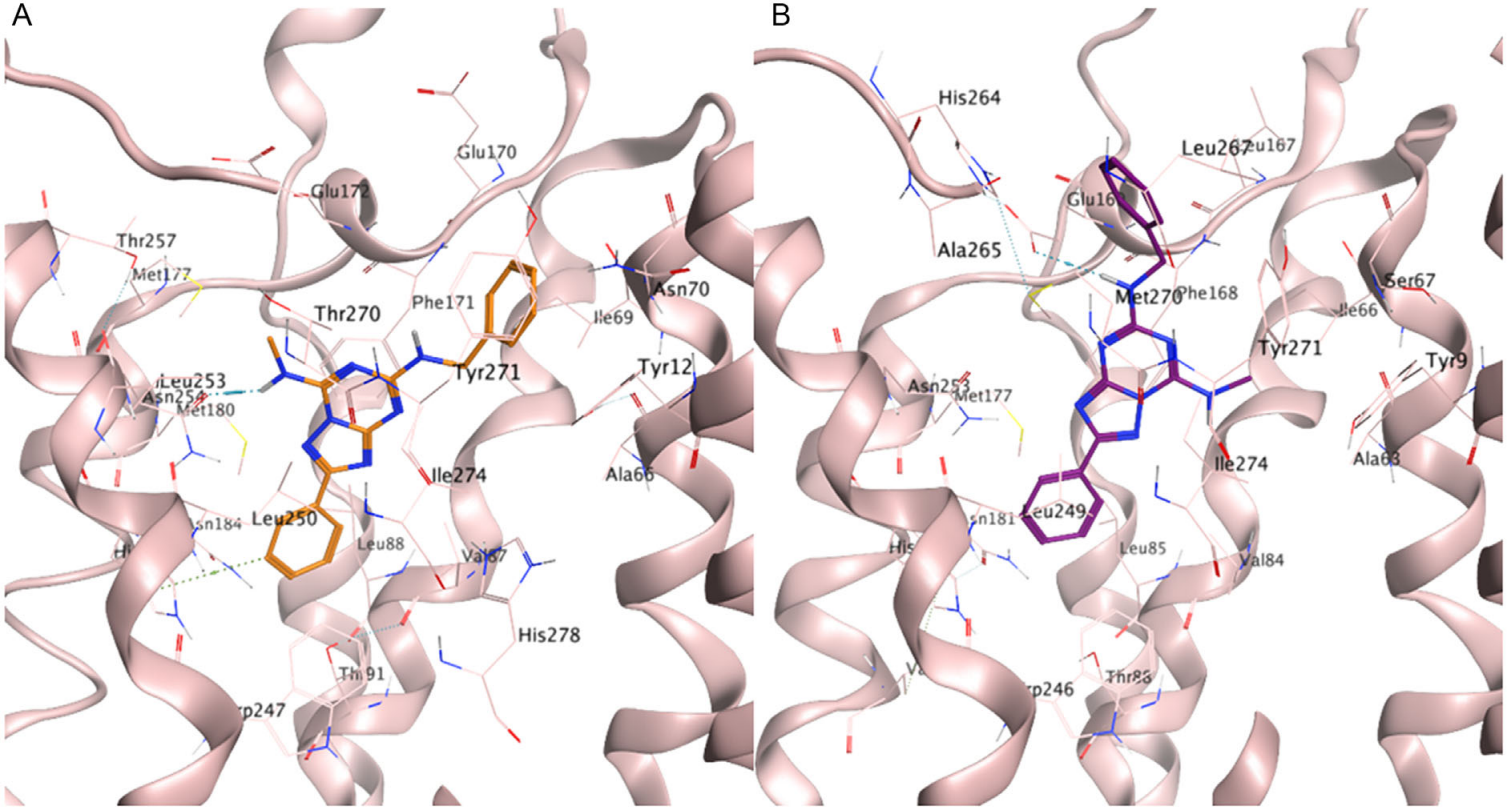
Predicted binding modes of compound **15** at hA_1_ AR (PDB ID: 5N2S; (A) ligand in orange) and hA_2A_ AR (PDB ID: 5NM4; (B), ligand in purple); both receptors are shown in pink. At hA_1_ AR, the compound adopts a ZM 241 385‐like pose, whereas at hA_2A_ AR the intra‐receptor Glu169–His264 salt bridge on the extracellular side prevents the analogous placement and the ligand is accommodated in a shallower, alternative orientation.

At hA_1_ AR, most compounds were also predicted to adopt a ZM‐like pose (Video S3 and Figure 6A), despite the generally poor experimental affinity. Even if a solid rational cannot be derived from the docking‐predicted bound states alone, a possible explanation is provided by subtype‐dependent differences at positions 5.42 and 6.52, where hA_3_ AR contains Ser181 and Ser247 instead of the bulkier conserved residues found in the other subtypes (Asn and His). These substitutions create a more spacious sub‐pocket that may better accommodate bulky substituents, consistent with the affinity and selectivity profiles reported in Tables 1 and 2.

A number of limitations of the present analysis should be explicitly acknowledged. The binding hypotheses reported here are the outcome of a rigid‐receptor docking protocol followed by a local, tethered refinement; no attempt was made to reproduce the experimental affinity ranking by means of the docking scores, and GBVI/WSA dG values were used exclusively as a criterion to select one representative pose per compound within each receptor. The persistence of two alternative and similarly scored binding modes at hA_3_ AR for a large part of the series is in itself an indication that this static description does not establish which of them is actually populated in solution; extended or supervised molecular dynamics simulations, together with the synthesis of conformationally constrained analogs, would be required to discriminate between them. Finally, the hA_1_ AR template is a thermostabilized construct, and the hA_3_ AR template is a BRIL‐fusion, nanobody‐stabilized complex bound to a covalent ligand, so that the side‐chain positions at the periphery of the orthosteric site should be regarded as approximate.

### 2.4 In Vitro OGD Studies

To characterize the effects of the selective A_3_AR antagonist, compound **15**, on AD development, in a first series of experiments, we applied an OGD insult 30 min long, a time that always allows AD appearance in all experimental groups. This duration of OGD is consistently associated with tissue damage [34, 48, 49, 50]. We compared the latency and amplitude of the depolarizing direct current (d.c.) shifts recorded in the absence or presence of **15** (10 nM). As illustrated in Figure 7, 30 min of OGD induced AD in all hippocampal slices, with a mean latency of 5.87 ± 0.31 min (*n* = 9) and a mean amplitude of 6.85 ± 0.89 mV (*n* = 9). In the presence of 10 nM **15**, the onset of the d.c. shift was significantly delayed to 8.25 ± 0.33 min (Figure 7B,C, *n* = 8), whereas the AD amplitude was not significantly affected (4.99 ± 0.75 mV).

**FIGURE 7:**
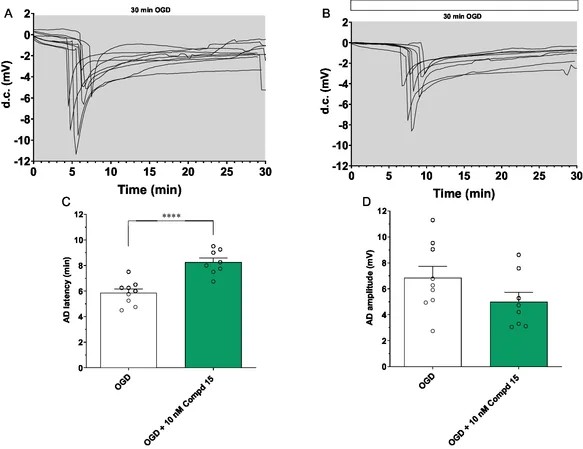
The selective A_3_AR antagonist, **15**, significantly delayed the appearance of AD induced by 30 min OGD in the CA1 region of rat hippocampal slices. (A,B) The graphs show original voltage traces of d.c. shift recorded during 30 min OGD in untreated slices ((A) *n* = 9 taken from 8 rats) or treated with 10 nM of compound **15** ((B) *n* = 8 slices taken from 7 rats). Gray bar: OGD time duration. Open bar: time of drug application. (C,D) Each column represents the mean ± SEM of AD latency (C), measured from the beginning of the OGD insult, or AD amplitude (D), recorded in CA1 hippocampal slices during 30‐min OGD insults in the absence or in the presence of 10 nM of compound **15**. Note that the A_3_AR antagonist significantly delayed AD development. **** *p* < 0.0001 unpaired two‐tailed Student's *t*‐test in comparison to untreated OGD slices.

Compound **15**, applied 15 min before OGD, throughout the insult, and for 5 min after reperfusion (data not shown), did not alter basal synaptic transmission prior to OGD. Specifically, the fEPSP amplitude was −0.93 ± 0.09 mV (mean ± SEM) before drug application and −0.97 ± 0.09 mV after 15 min of **15** superfusion in oxygenated aCSF (*n* = 8).

In a next experimental series, we tested the effects of 7 min of OGD, in the absence or in the presence of the new selective A_3_ AR antagonist **15**, on synaptic potentials and AD onset in the CA1 region of the rat hippocampus. In agreement with our previous results [34, 51], in untreated OGD slices, AD, recorded as negative d.c. shifts, presented a peak latency of 6.00 ± 0.16 min (calculated from the beginning of the insult) and an amplitude of 5.95 ± 1.25 mV (Figure 8A, *n* = 10). Furthermore, 7 min of OGD exposure consistently produced an irreversible loss of synaptic transmission evoked by Schaffer‐collateral stimulation since potentials did not recover their amplitude when superfused in oxygenated aCSF (Figure 8A,C, *n* = 10, 3.02% ± 1.90%).

**FIGURE 8:**
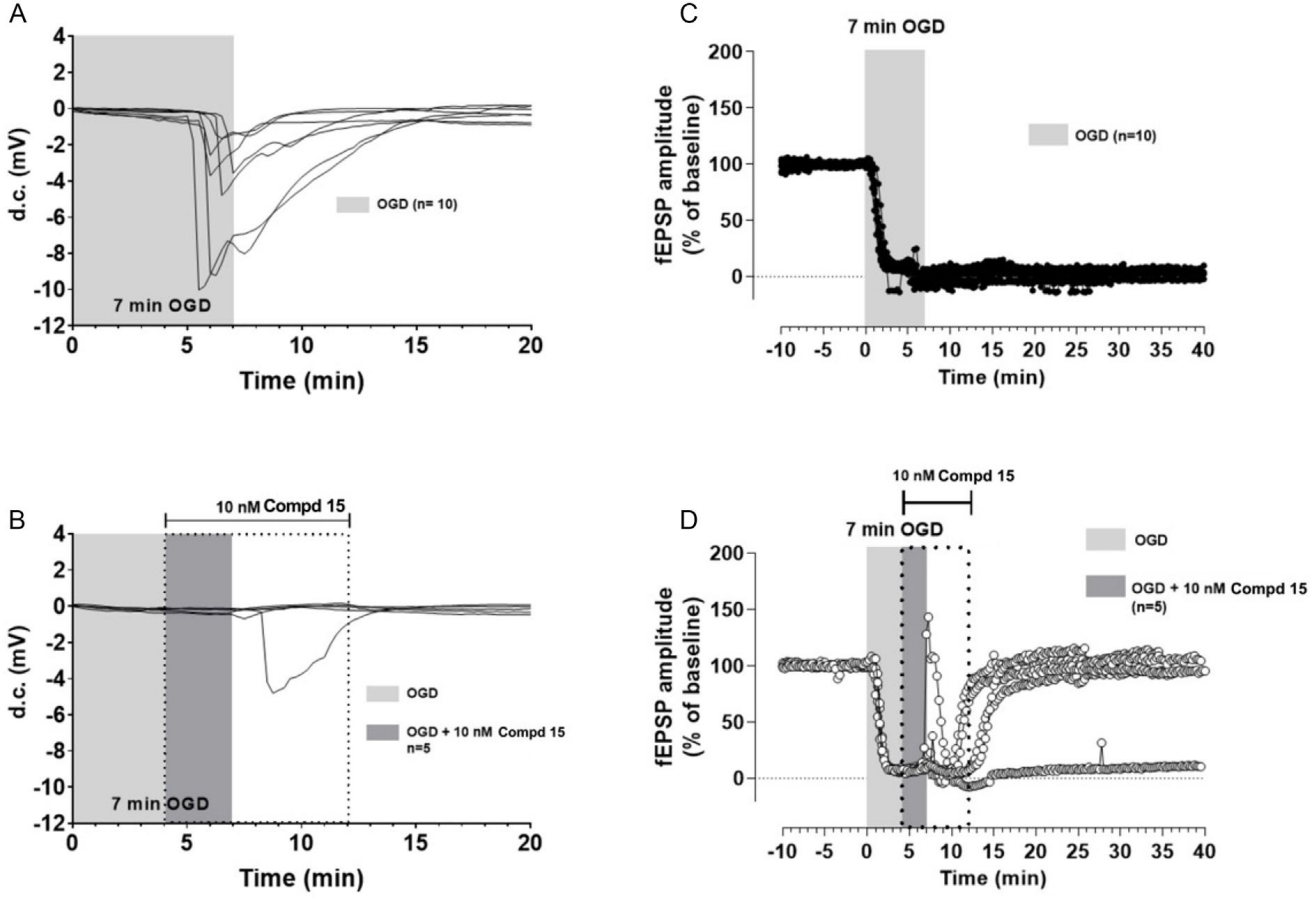
Compound 15 significantly reduced synaptic failure induced by severe OGD also when applied in the last 3 min of the insult. (A,B) Anoxic depolarization (AD) was recorded as a negative direct current (d.c.) shift in response to 7 min OGD in untreated OGD slices ((A) *n* = 10 slices taken from 10 rats) or in 10 nM compound **15**‐treated slices ((B) *n* = 5 slices taken from 5 animals). Note that **15** was applied during the last 3 min of OGD and the subsequent 5 min in oxygenated aCSF. The compound prevented the appearance of AD in 4 out of 5 slices examined. (C) The graph shows the time‐course of the effect of 7 min OGD on fEPSP amplitude, expressed as a percentage of pre‐OGD baseline, in untreated OGD slices (*n* = 1 0). Note that, in these conditions, the ischemic‐like insult caused a gradual reduction, up to disappearance, of fEPSP amplitude that did not recover after washing in oxygenated aCSF. (D) The graph shows the time‐course of the effect of 7 min OGD on fEPSP amplitude, expressed as a percentage of pre‐OGD baseline, in the presence of 10 nM of compound **15** (*n* = 5). Note that after reperfusion in oxygenated solution, a recovery of fEPSP was found in all slices in which AD did not develop (4 out of 5). Light gray bar: OGD; Dark gray bar: OGD + **15**; dotted square: time of drug application.

The effects of 10 nM compound **15** on synaptic transmission and AD development were tested by applying the compound during the last 3 min of the 7 min‐long OGD insult and for 5 min after the end of OGD. Compound **15** applied was able to prevent the AD onset in 4 out of 5 slices tested. In these four slices, field excitatory postsynaptic potentials (fEPSPs) exhibited an almost complete recovery, reaching 99.0% ± 3.5% of the pre‐OGD baseline 30 min after the end of OGD (Figure 8B,D, *n* = 4).

According to our previous findings [34, 51], these results demonstrated that compound **15** significantly protected the CA1 region of hippocampal slices during OGD by delaying the onset of AD and preventing irreversible synaptic failure even when applied during the final minutes of the ischemic insult.

We can exclude the possibility that the effects of compound **15**, at the concentration used in the present study, were mediated by the blockade of A_1_ ARs. Indeed, A_1_ AR blockade in the CA1 region of the rat hippocampus is well known to induce a significant increase in both the amplitude and slope of fEPSPs [52]. Furthermore, in contrast to the A_3_ AR antagonist, the selective A_1_ AR antagonist DPCPX impairs the recovery of synaptic potentials following a hypoxic insult [53].

Concerning the A_2A_ AR, we show in Table 1 that compound **15** has a *K*_i_ = 99.3 nM at the human A_2A_ AR, and the rat counterpart usually aligns in affinity or efficacy values [21]. Hence, we can rule out that compound **15**, applied at 10 nM in rat hippocampal slices, may bind to the A_2A_ AR.

## 3 Experimental Section

### 3.1 Chemistry

#### 3.1.1 General

Reagents were obtained from commercial suppliers and used without further purification. Reactions were monitored by TLC on precoated silica gel plates (Macherey–Nagel, 60FUV254). Final compounds and intermediates were purified by flash chromatography using as stationary phase silica gel (Macherey–Nagel, silica 60, 240–400 mesh). When used, light petroleum ether (PE) refers to the fractions boiling at 40–60 °C. The ^1^H NMR (Nuclear Magnetic Resonance), ^13^C NMR, and Heteronuclear Single Quantum Coherence (HSQC) bidimensional spectra were recorded on Bruker Avance NEO 400 MHz Nanobay and 600 MHz Onebay NMR spectrometers; chemical shifts (*δ*) are reported in parts per million (ppm) and referenced to the residual solvent peak, with splitting patterns abbreviated to: s (singlet), d (doublet), t (triplet), m (multiplet), and bs (broad signal). Coupling constants (J) are given in Hz. ESI‐MS analyses were performed using an ESI Bruker 4000 Esquire spectrometer and, for HRMS, Orbitrap Exploris 240 and 480. Compound purity was determined by HPLC using the instrument SHIMADZU CBM‐20A, column Gemini 5 μm NX‐C18 (Phenomenex), following different methods: an elution gradient was performed for 20 min at a flow rate of 1 mL/min from water‐acetonitrile 70:30 to 15:85 (method A) or water‐methanol 35:65 to 5:95 (method B), detecting at 254 nm. Intermediate compounds **32–36** and final compounds **1**, **6**,**10**, **11**, and **28** were prepared according to previously reported procedures. Their analytical and spectroscopic data were in agreement with those reported in the literature [36, 37].

#### 3.1.2 General Procedure for the Synthesis of 5‐Phenoxy‐[1,2,4]triazolo[1,5‐*a*[1,3,5]triazine‐7‐amines (2–5, 7)

The appropriate 5,7‐diphenoxy‐[1,2,4]triazolo[1,5‐*a*[1,3,5]triazine (**35**, **36**, 1 eq) was dissolved in ethanol (10 mL/mmol), followed by the addition of the required amine (1–4 eq) under stirring at 50–90 °C in a sealed tube for 5–18 h. Upon completion of the reaction, the solvent was removed, the residue was purified by column chromatography, and recrystallized if necessary.

*N*‐methyl‐5‐phenoxy‐2‐phenyl‐[1,2,4]triazolo[1,5‐*a*[1,3,5]triazine‐7‐amine (**2**). 5,7‐diphenoxy‐2‐phenyl‐[1,2,4]triazolo[1,5‐*a*[1,3,5]triazine (**35**, 100 mg, 0.262 mmol), 33 wt% methylamine solution in ethanol (2 eq., 65 µl), 18 h, column chromatography eluent DCM 98/EtOAc 2. Yield 91.6% (76.5 mg, white solid). ^1^H NMR (400 MHz, DMSO‐*d*_6_) δ 9.27 (q, *J* = 4.6 Hz, 1H), 8.24–8.18 (m, 2H), 7.67–7.59 (m, 3H), 7.56 (t, *J* = 7.8 Hz, 2H), 7.43–7.32 (m, 3H), 3.09 (d, *J* = 4.6 Hz, 3H). ^13^C NMR (101 MHz, DMSO‐*d*_6_) *δ* 165.23, 164.11, 159.11, 152.77, 151.10, 131.05, 130.66, 129.96, 129.38, 127.21, 125.97, 122.31, 27.95. ESI‐HRMS m/z C_17_H_14_N_6_O: experimental 319.1300 [M + H]*^+^* *,* theoretical 319.1302 [M + H]^+^, Δ = 0.0002. Purity by HPLC (method A): 98.9%.

*N*‐cyclopropyl‐5‐phenoxy‐2‐phenyl‐[1,2,4]triazolo[1,5‐*a*[1,3,5]triazin‐7‐amine (**3**). 5,7‐diphenoxy‐2‐phenyl‐[1,2,4]triazolo[1,5‐a[1,3,5]triazine (**35**, 355 mg, 0.931 mmol), cyclopropylamine (1.3 eq., 1.192 mmol, 83 µl), 12 h, column chromatography eluent DCM, followed by DCM 95/EtOAc 5 and DCM 95/MeOH 5, recrystallized from EtOAc‐PE. Yield 84% (269 mg, white solid). ^1^H NMR (400 MHz, DMSO‐*d*_6_) δ 9.45 (d, *J* = 3.9 Hz, 1H), 8.23–8.16 (m, 2H), 7.64–7.49 (m, 5H), 7.41–7.32 (m, 3H), 3.13–2.94 (m, 1H), 1.00–0.73 (m, 4H). ^13^C NMR (101 MHz, DMSO‐*d*_6_) *δ* 165.17, 164.11, 159.13, 152.79, 151.98, 131.03, 130.66, 129.94, 129.34, 127.29, 125.94, 122.32, 24.52, 6.36. ESI‐HRMS m/z: C_19_H_16_N_6_O: experimental 345.1459 [M + H]^+^, theoretical 345.1458 [M + H]^+^, Δ = 0.0001. Purity by HPLC (method A): 98.3%.

*N*‐cyclohexyl‐5‐phenoxy‐2‐phenyl‐[1,2,4]triazolo[1,5‐*a*[1,3,5]triazine‐7‐amine (**4**) was obtained. 5,7‐diphenoxy‐2‐phenyl‐[1,2,4]triazolo[1,5‐a[1,3,5]triazine (**35**, 760 mg, 1.993 mmol), cyclohexylamine (1 eq., 228 µl), 4 h, column chromatography eluent PE/EtOAc (gradient, 85/15; 70/30; 60/40; 50/50; 0/100), recrystallized from Et_2_O‐PE. Yield was 90% (693 mg, white solid). ^1^H NMR (600 MHz, DMSO‐*d*_6_) δ 9.13 (s, 1H), 8.18–8.14 (m, 2H), 7.56–7.52 (m, 3H), 7.48 (t, *J* = 7.7 Hz, 2H), 7.37–7.22 (m, 3H), 3.95–3.84 (m, 1H), 1.91–1.87 (m, 2H), 1.80–1.75 (m, 2H), 1.67–1.49 (m, 3H), 1.32–1.23 (m, 2H), 1.15–1.09 (m, 1H). ^13^C NMR (151 MHz, DMSO‐*d*_6_) δ 165.22, 164.10, 159.41, 152.81, 149.87, 131.02, 130.69, 129.89, 129.33, 127.32, 125.92, 122.36, 51.22, 31.99, 25.40. ESI‐MS (acetonitrile) *m/z:* 387.2 [M + H]^+^, 409.2 [M + Na]^+^, 425.1 [M + K]^+^. Purity by HPLC (method A): 98.9%.

*N*‐Benzyl‐5‐phenoxy‐2‐phenyl‐[1,2,4]triazolo[1,5‐*a*[1,3,5]triazine‐7‐amine (**5**) was obtained. 5,7‐diphenoxy‐2‐phenyl‐[1,2,4]triazolo[1,5‐a[1,3,5]triazine (**35**, 760 mg, 1.993 mmol), benzylamine (1.5 eq., 327 µl), 18 h, column chromatography eluent DCM/MeOH (gradient, 98/2; 90/10), recrystallized from Et_2_O‐PE. Yield: 84% (662 mg, white solid). ^1^H NMR (600 MHz, DMSO‐*d*_6_) δ 9.88 (s, 1H), 8.23–8.19 (m, 2H), 7.64–7.56 (m, 3H), 7.54 (t, *J* = 7.8 Hz, 2H), 7.45–7.24 (m, 8H), 4.68 (s, 2H). ^13^C NMR (151 MHz, DMSO‐*d*_6_) *δ* 165.17, 164.29, 159.38, 152.81, 150.80, 138.15, 131.09, 130.65, 129.96, 129.37, 128.83, 128.26, 127.75, 127.27, 125.99, 122.35, 44.35. ESI‐HRMS m/z: C_23_H_18_N_6_O: experimental 345.1615 [M + H]^+^, theoretical 395.1615 [M + H]^+^, Δ = 0.0000. Purity by HPLC (method A): 98.8%.

*N*‐Benzyl‐2‐(4‐methoxyphenyl)‐5‐phenoxy‐[1,2,4]triazolo[1,5‐*a*[1,3,5]triazine‐7‐amine (**7**) was obtained. 2‐(4‐methoxyphenyl)‐5,7‐diphenoxy‐[1,2,4]triazolo[1,5‐a[1,3,5]triazine (**36**, 140 mg, 0.340 mmol), benzylamine (4 eq., 149 µl), 14 h, column chromatography eluent DCM 98/EtOAc 2, recrystallized from EtOAc‐Et_2_O. Yield was 78% (112 mg, white solid). ^1^H NMR (600 MHz, DMSO‐d6) δ 9.82 (t, *J* = 6.3 Hz, 1H), 8.14 (d, *J* = 8.6 Hz, 2H), 7.53 (t, *J* = 7.8 Hz, 2H), 7.42–7.29 (m, 8H), 7.16 (d, *J* = 8.7 Hz, 2H), 4.67 (d, *J* = 6.2 Hz, 2H), 3.89 (s, 3H). ^13^C NMR (151 MHz, DMSO‐*d*_6_) δ 165.10, 164.27, 161.64, 159.30, 152.82, 150.69, 138.19, 129.94, 128.89, 128.83, 128.24, 127.73, 125.96, 123.03, 122.36, 114.79, 55.80, 44.30. ESI‐HRMS m/z: C_24_H_20_N_6_O_2_: experimental 425.1721 [M + H]^+^, theoretical 425.1721 [M + H]^+^, Δ = 0.0000. Purity by HPLC (method B): 98.6%.

#### 3.1.3 Synthesis of *N* ^5^,*N* ^7^‐Dimethyl‐2‐phenyl‐[1,2,4]triazolo[1,5‐*a*[1,3,5]triazine‐5,7‐diamine (12)

The 5,7‐diphenoxy‐2‐phenyl‐[1,2,4]triazolo[1,5‐*a*[1,3,5]triazine (**35**, 290 mg, 0.760 mmol) was dissolved in 5 mL of ethanol, and 33 wt% methylamine solution in ethanol (4 eq., 379 µl) was added. The solution was stirred and heated at 90 °C in a sealed tube for 5 h. The reaction was monitored by TLC. Upon completion of the reaction, the solvent was removed, and the residue was purified by gradient column chromatography starting with DCM/EtOAc 98/2. Yield was 47.9% (93.0 mg, white solid). ^1^H NMR (600 MHz, DMSO‐*d*_6_) *δ* 8.36–8.32 (m, 1H), 8.11 (d, *J* = 7.3 Hz, 2H), 7.55–7.48 (m, 4H), 2.95 (d, *J* = 4.6 Hz, 3H), 2.82 (d, *J* = 4.6 Hz, 3H). ^13^C NMR (151 MHz, DMSO‐*d*_6_) *δ* 163.01, 161.86, 159.66, 149.34, 131.36, 130.50, 129.18, 127.05, 28.18, 27.64. ESI‐HRMS m/z: C_12_H_13_N_7_: experimental 256.1303 [M + H]^+^, theoretical 256.1305 [M + H]^+^, Δ = 0.0002. Purity by HPLC (method A): 98.4%.

#### 3.1.4 General Procedure for the Synthesis of *N* ^5^,*N* ^7^‐Substituted‐[1,2,4]triazolo[1,5‐*a*[1,3,5]triazine‐5,7‐diamines (8, 9, 13–27, 29, 30)

The corresponding *N* ^7^‐substituted 5‐phenoxy‐[1,2,4]triazolo[1,5‐*a*[1,3,5]triazin‐7‐amine (**1–7**) was dissolved in ethanol (10 mL/mmol), followed by the addition of the required amine (3–20 eq) under heating at 100–120 °C in a sealed tube for 2–72 h. The solvent was then removed under reduced pressure, and the residue was purified by column chromatography using a suitable eluting system and recrystallized if necessary.

*N* ^5^‐Methyl‐2‐phenyl‐[1,2,4]triazolo[1,5‐*a*[1,3,5]triazine‐5,7‐diamine (**8**) was obtained. 5‐phenoxy‐2‐phenyl‐[1,2,4]triazolo[1,5‐*a* [1,3,5]triazin‐7‐amine (**1**, 60 mg, 0.197 mmol), 33 wt% methylamine solution in ethanol (13 eq., 319 µl), 5 h, column chromatography eluent DCM 95/MeOH 5, recrystallized from EtOAc‐Et_2_O. Yield: 63% (30 mg, white solid). ^1^H NMR (600 MHz, DMSO‐*d*_6_) δ 8.17 (d, *J* = 7.1 Hz, 2H), 7.59–7.53 (m, 3H), 7.38–7.33 (m, 1H), 2.87 (d, *J* = 4.7 Hz, 3H). ^13^C NMR (151 MHz, DMSO‐*d*_6_) δ 163.07, 162.02, 159.97, 150.41, 131.37, 130.51, 129.15, 127.08, 28.14. ESI‐HRMS m/z: C_11_H_11_N_7_: experimental 242.1147 [M + H]^+^, theoretical 242.1149 [M + H]^+^, Δ = 0.0002. Purity by HPLC (method A): 98.9%.

*N* ^5^‐Cyclopropyl‐2‐phenyl‐[1,2,4]triazolo[1,5‐*a*[1,3,5]triazine‐5,7‐diamine (**9**). 5‐phenoxy‐2‐phenyl‐[1,2,4]triazolo[1,5‐*a*[1,3,5]triazine‐7‐amine (**1**, 60 mg, 0.197 mmol), cyclopropylamine (3 eq., 41 µl), 24 h, column chromatography eluent DCM 98/MeOH 2, recrystallized from EtOAc‐Et_2_O‐PE. Yield was 25% (13 mg, white solid). ^1^H NMR (600 MHz, DMSO‐*d*_6_) *δ* 8.14–8.10 (m, 3H), 7.67–7.40 (m, 4H), 2.87–2.70 (m, 1H), 0.71–0.67 (m, 2H), 0.54–0.51 (m, 2H). ^13^C NMR (151 MHz, DMSO‐*d*_6_) *δ* 163.15, 159.87, 154.43, 150.36, 131.37, 130.54, 129.16, 127.12, 24.23, 6.68. ESI‐HRMS m/z: C_13_H_13_N_7_: experimental 268.1306 [M + H]^+^, theoretical 268.1305 [M + H]^+^, Δ = 0.0001. Purity by HPLC (method A): 98.9%.

From this reaction, N^5^,N^7^‐dicyclopropyl‐2‐phenyl‐[1,2,4]triazolo[1,5‐*a*[1,3,5]triazine‐5,7‐diamine (**17**) was obtained as a byproduct. Yield: 25% (15 mg, white solid). ^1^H NMR (600 MHz, DMSO‐*d*_6_) δ 8.52 (s, 1H), 8.13 (d, *J* = 7.2 Hz, 2H), 7.77 (s, 1H), 7.53–7.48 (m, 3H), 2.91 (s, 1H), 2.80 (s, 1H), 0.88–0.65 (m, 6H), 0.62–0.45 (m, 2H). ^13^C NMR (151 MHz, DMSO‐*d*_6_) δ 163.04, 162.49, 159.60, 150.04, 131.34, 130.52, 129.15, 127.14, 24.20, 6.59. ESI‐MS (methanol) *m/z:* 308.1 [M + H]^+^, 330.1 [M + Na]^+^. Purity by HPLC (method A): 95.9%.

*N* ^5^‐Cyclopropyl‐N^7^‐methyl‐2‐phenyl‐[1,2,4]triazolo[1,5‐*a*[1,3,5]triazine‐5,7‐diamine (**13**). *N*‐methyl‐5‐phenoxy‐2‐phenyl‐[1,2,4]triazolo[1,5‐*a*[1,3,5]triazine‐7‐amine (**2**, 70 mg, 0.220 mmol), cyclopropylamine (20 eq., 305 µl), 72 h, column chromatography eluent DCM/EtOAc (gradient, 95/5; 90/10). Yield was 24% (15 mg, white solid). ^1^H NMR (600 MHz, CD_3_OD) *δ* 8.16–8.13 (m, 2H), 7.48–7.44 (m, 3H), 3.12–3.02 (m, 3H), 2.89–2.76 (m, 1H), 0.82–0.74 (m, 2H), 0.60–0.57 (m, 2H). ^13^C NMR (151 MHz, CD_3_OD) *δ* 163.01, 159.45, 155.28, 149.31, 130.49, 129.93, 128.21, 126.70, 26.13, 23.25, 5.80. ESI‐HRMS m/z: C_14_H_15_N_7_: experimental 282.1461 [M + H]^+^, theoretical 282.1462 [M + H]^+^, Δ = 0.0001. Purity by HPLC (method B): 98.4%.

*N* ^5^‐Cyclohexyl‐*N* ^7^‐methyl‐2‐phenyl‐[1,2,4]triazolo[1,5‐*a*[1,3,5]triazine‐5,7‐diamine (**14**). *N*‐methyl‐5‐phenoxy‐2‐phenyl‐[1,2,4]triazolo[1,5‐*a*[1,3,5]triazine‐7‐amine (**2**, 46 mg, 0.145 mmol), cyclohexylamine (6 eq., 99 µl), 15 h, column chromatography eluent DCM 95/EtOAc 5. Yield: 13% (6 mg, white solid). ^1^H NMR (600 MHz, CD_3_OD) *δ* 8.05–8.01 (m, 2H), 7.38–7.34 (m, 3H), 3.81–3.72 (m, 1H), 3.05–2.93 (m, 3H), 1.96–1.92 (m, 2H), 1.73–1.68 (m, 2H), 1.60–1.55 (m, 1H), 1.38–1.09 (m,5H). ^13^C NMR (151 MHz, CD_3_OD) δ 162.79, 160.83, 159.56, 149.41, 130.91, 129.92, 128.21, 126.67, 49.81, 32.41, 26.12, 25.40, 24.87. ESI‐HRMS m/z: C_17_H_21_N_7_: experimental 324.1932 [M + H]^+^, theoretical 324.1931 [M + H]^+^, Δ = 0.0001. Purity by HPLC (method B): 95.0%.

*N* ^5^‐Benzyl‐*N* ^7^‐methyl‐2‐phenyl‐[1,2,4]triazolo[1,5‐*a*[1,3,5]triazine‐5,7‐diamine (**15**) was used. *N*‐methyl‐5‐phenoxy‐2‐phenyl‐[1,2,4]triazolo[1,5‐*a*[1,3,5]triazine‐7‐amine (**2**, 50 mg, 0.157 mmol), benzylamine (6 eq., 103 µl), 18 h, column chromatography eluent DCM 95/EtOAc 5. Yield was 38% (20 mg, white solid). ^1^H NMR (400 MHz, CD_3_OD) *δ* 8.06–8.00 (m, 2H), 7.39–7.32 (m, 3H), 7.29 (d, *J* = 7.1 Hz, 2H), 7.21 (t, *J* = 7.5 Hz, 2H), 7.16–7.08 (m, 1H), 4.60–4.52 (m, 2H), 2.99 (s, 3H). ^13^C NMR (101 MHz, CD_3_OD) *δ* 161.79, 159.43, 149.48, 139.31, 130.42, 129.94, 128.22, 128.01, 127.10, 126.68, 126.60, 44.29, 26.16. ESI‐HRMS m/z: C_18_H_17_N_7_: experimental 332.1617 [M + H]^+^, theoretical 332.1618 [M + H]^+^, Δ = 0.0001. Purity by HPLC (method A): 96.1%.

*N* ^7^‐Cyclopropyl‐*N* ^5^‐methyl‐2‐phenyl‐[1,2,4]triazolo[1,5‐*a*[1,3,5]triazine‐5,7‐diamine (**16**) was used. *N*‐cyclopropyl‐5‐phenoxy‐2‐phenyl‐[1,2,4]triazolo[1,5‐*a*[1,3,5]triazine‐7‐amine (**3**, 100 mg, 0.290 mmol), 33 wt% methylamine solution in ethanol (3 eq., 109 µl), 6 h, column chromatography eluent DCM/EtOAc (gradient, 100/0; 97.5/2.5; 98/2), recrystallized from EtOAc‐PE‐Et_2_O Yield 33% (27 mg, white solid). ^1^H NMR (600 MHz, CDCl_3_) *δ* 8.32–8.10 (m, 2H), 7.46–7.42 (m, 3H), 6.27 (bs, 1H), 5.33 (bs, 1H), 3.08 (d, *J* = 4.9 Hz, 3H), 2.90 (s, 1H), 1.04–0.84 (m, 2H), 0.87–0.69 (m, 2H). ^13^C NMR (151 MHz,CDCl_3_) *δ* 164.37, 161.90, 160.63, 159.38, 149.83, 130.74, 130.38, 128.70, 127.38, 28.54, 23.42, 7.24. ESI‐HRMS m/z: C_14_H_15_N_7_: experimental 282.1462 [M + H]^+^, theoretical 282.1462 [M + H]^+^, Δ = 0.0000. Purity by HPLC (method A): 96.1%.

*N* ^5^‐Cyclohexyl‐*N* ^7^‐cyclopropyl‐2‐phenyl‐[1,2,4]triazolo[1,5‐*a*[1,3,5]triazine‐5,7‐diamine (**18**). *N*‐cyclopropyl‐5‐phenoxy‐2‐phenyl‐[1,2,4]triazolo[1,5‐*a*[1,3,5]triazine‐7‐amine (**3**, 84 mg, 0.244 mmol), cyclohexylamine (3 eq., 84 µl), 12 h, column chromatography eluent DCM 98/MeOH 2, recrystallized from DCM‐PE. Yield was 46% (39 mg, white solid). ^1^H NMR (400 MHz, DMSO‐*d*_6_) *δ* 8.46 (s, 1H), 8.11 (d, *J* = 6.9 Hz, 2H), 7.50 (s, 4H), 3.77 (s, 1H), 2.94 (s, 1H), 2.06–1.51 (m,5H), 1.47–1.03 (m, 5H), 0.88–0.65 (m, 4H). ^13^C NMR (101 MHz, DMSO‐*d*_6_) *δ* 162.96, 160.43, 159.62, 150.15, 131.30, 130.51, 129.14, 127.10, 50.00, 32.69, 25.76, 25.42, 24.19, 6.52. ESI‐HRMS m/z: C_19_H_23_N_7_: experimental 350.2089 [M + H]^+^, theoretical 350.2088 [M + H]^+^, Δ = 0.0001. Purity by HPLC (method A): 99.8%.

*N* ^5^‐Benzyl‐*N* ^7^‐cyclopropyl‐2‐phenyl‐[1,2,4]triazolo[1,5‐*a*[1,3,5]triazine‐5,7‐diamine (**19**) was used. *N*‐cyclopropyl‐5‐phenoxy‐2‐phenyl‐[1,2,4]triazolo[1,5*‐a*[1,3,5]triazine‐7‐amine (**3**, 72.6 mg, 0.211 mmol), benzylamine (3 eq., 62 µl), 12 h, column chromatography eluent DCM 98/MeOH 2, recrystallized from Et_2_O‐PE. Yield was 20% (15 mg, white solid). ^1^H NMR (600 MHz, CDCl_3_) *δ* 8.27–8.13 (m, 2H), 7.51–7.19 (m, 8H), 4.73 (d, *J* = 5.2 Hz, 2H), 3.02–2.82 (m, 1H), 1.02–0.86 (m, 2H), 0.83–0.70 (m, 2H). ^13^C NMR (151 MHz, CDCl_3_) *δ* 164.37, 161.14, 159.26, 150.02, 138.42, 130.46, 128.82, 128.72, 128.22, 127.69, 127.56, 127.40, 45.75, 23.45, 7.25. ESI‐HRMS m/z: C_20_H_19_N_7_: experimental 358.1771 [M + H]^+^, theoretical 358.1775 [M + H]^+^, Δ = 0.0004. Purity by HPLC (method B): 96.9%.

*N* ^7^‐Cyclohexyl‐*N* ^5^‐methyl‐2‐phenyl‐[1,2,4]triazolo[1,5‐*a*[1,3,5]triazine‐5,7‐diamine (**20**) was used. N‐cyclohexyl‐5‐phenoxy‐2‐phenyl‐[1,2,4]triazolo[1,5‐*a*[1,3,5]triazine‐7‐amine (**4**, 120 mg, 0.311 mmol), 33 wt% methylamine solution in ethanol (3 eq., 116 µl), 2–3 h, column chromatography eluent DCM 99/MeOH 1, recrystallized from Et_2_O‐PE. Yield was 47% (47 mg, white solid). ^1^H NMR (600 MHz, CD_3_OD) *δ* 8.15–8.11 (m, 2H), 7.48–7.44 (m, 3H), 4.02 (s, 1H), 2.95 (s, 3H), 2.13–2.00 (m, 2H), 1.84 (d, *J* = 12.6 Hz, 2H), 1.70 (d, *J* = 12.8 Hz, 1H), 1.59–1.34 (m, 4H), 1.33–1.22 (m, 1H). ^13^C NMR (151 MHz, CD_3_OD) *δ* 162.86, 162.22, 159.68, 147.95, 130.42, 129.97, 128.25, 126.73, 50.06, 32.01, 26.80, 25.11, 24.98. ESI‐HRMS m/z: C_17_H_21_N_7_: experimental 324.1932 [M + H]^+^, theoretical 324.1931 [M + H]^+^, Δ = 0.0001. Purity by HPLC (method B): 95.1%.

*N* ^7^‐Cyclohexyl‐*N* ^5^‐cyclopropyl‐2‐phenyl‐[1,2,4]triazolo[1,5‐*a*[1,3,5]triazine‐5,7‐diamine (**21**). N‐cyclohexyl‐5‐phenoxy‐2‐phenyl‐[1,2,4]triazolo[1,5‐*a*[1,3,5]triazine‐7‐amine (**4**, 120 mg, 0.311 mmol), cyclopropylamine (6 eq., 129 µl), 48 h, column chromatography eluent DCM, followed by DCM 95/MeOH 5, recrystallized from Et_2_O‐PE. Yield was 30% (32 mg, white solid). ^1^H NMR (400 MHz, CD_3_OD) *δ* 8.16–8.09 (m, 2H), 7.47–7.41 (m, 3H), 4.01 (s, 1H), 2.84–2.74 (m, 1H), 2.03 (s, 2H), 1.82 (d, *J* = 12.3 Hz, 2H), 1.68 (d, *J* = 12.8 Hz, 1H), 1.56–1.18 (m, 5H), 0.76 (m, 2H), 0.60–0.51 (m, 2H). ^13^C NMR (101 MHz, CD_3_OD) *δ* 163.01, 159.59, 158.93, 147.97, 130.45, 129.96, 128.24, 126.75, 50.19, 31.97, 25.11, 25.00, 23.26, 5.81. ESI‐HRMS m/z: C_19_H_23_N_7_: experimental 350.2086 [M + H]^+^, theoretical 350.2088 [M + H]^+^, Δ = 0.0002. Purity by HPLC (method B): 98.3%.

*N* ^5^,*N* ^7^‐Dicyclohexyl‐2‐phenyl‐[1,2,4]triazolo[1,5‐*a*[1,3,5]triazine‐5,7‐diamine (**22**). *N*‐cyclohexyl‐5‐phenoxy‐2‐phenyl‐[1,2,4]triazolo[1,5‐*a*[1,3,5]triazine‐7‐amine (**4**, 120 mg, 0.311 mmol), cyclohexylamine (6 eq., 213 µl), 12 h, column chromatography eluent DCM, followed by DCM 95/MeOH 5, recrystallized from EtOAc‐Et_2_O. Yield was 22% (27 mg, white solid). ^1^H NMR (400 MHz, CD_3_OD) *δ* 8.16–8.09 (m, 2H), 7.49–7.43 (m, 3H), 4.08–3.95 (m, 1H), 3.91–3.77 (m, 1H), 2.14–1.99 (m, 4H), 1.92–1.61 (m, 6H), 1.57–1.18 (m, 10H). ^13^C NMR (101 MHz, CD_3_OD) *δ* 162.79, 160.81, 159.71, 148.09, 130.44, 129.94, 128.23, 126.70, 49.97, 49.85, 32.42, 32.07, 25.41, 25.11, 25.02, 24.92. ESI‐HRMS m/z: C_22_H_29_N_7_: experimental 392.2556 [M + H]^+^, theoretical 392.2557 [M + H]^+^, Δ = 0.0001. Purity by HPLC (method B): 98.1%.

*N* ^5^‐Benzyl‐*N* ^7^‐cyclohexyl‐ 2‐phenyl‐[1,2,4]triazolo[1,5‐*a*[1,3,5]triazine‐5,7‐diamine (**23**). N‐cyclohexyl‐5‐phenoxy‐2‐phenyl‐[1,2,4]triazolo[1,5‐*a*[1,3,5]triazin‐7‐amine (**4**, 120 mg, 0.311 mmol), benzylamine (3 eq., 102 µl), 12 h, column chromatography eluent DCM, recrystallized from EtOAc‐PE. Yield was 51% (63 mg, white solid). ^1^H NMR (400 MHz, DMSO‐*d*_6_) *δ* 8.23–8.10 (m, 3H), 7.61–7.50 (m, 3H), 7.44–7.33 (m, 4H), 7.32–7.23 (m, 1H), 4.55 (dd, *J* = 13.5, 6.2 Hz, 2H), 4.07–4.00 (m, 1H), 2.02–1.06 (m, 10H). ^13^C NMR (101 MHz, DMSO‐*d*_6_) *δ* 162.87, 162.59, 161.19, 161.07, 159.31, 147.91, 140.41, 139.90, 130.82, 130.12, 128.71, 128.24, 128.14, 127.31, 127.25, 126.81, 126.73, 126.66, 49.68, 43.90, 31.85, 31.60, 25.12, 25.05. ESI‐HRMS m/z: C_23_H_25_N_7_: experimental 400.2241 [M + H]^+^, theoretical 400.2244 [M + H]^+^, Δ = 0.0003. Purity by HPLC (method B): 95.5%.

*N* ^7^‐Benzyl‐*N* ^5^‐methyl‐2‐phenyl‐[1,2,4]triazolo[1,5‐*a*[1,3,5]triazine‐5,7‐diamine (**24**). *N*‐Benzyl‐5‐phenoxy‐2‐phenyl‐[1,2,4]triazolo[1,5‐*a*[1,3,5]triazine‐7‐amine (**5**, 120 mg, 0.304 mmol), 33 wt% methylamine solution in ethanol (3 eq., 114 µl), 3–4 h, column chromatography eluent DCM/MeOH (gradient, 95/5; 90/10), recrystallized from Et_2_O‐PE. Yield was 48% (49 mg, white solid). ^1^H NMR (600 MHz, CD_3_OD) *δ* 8.16–8.12 (m, 2H), 7.47–7.40 (m, 5H), 7.33 (t, *J* = 7.4 Hz, 2H), 7.26 (d, *J* = 7.4 Hz, 1H), 4.82–4.68 (m, 2H), 2.95 (s, 3H). ^13^C NMR (151 MHz, CD_3_OD) δ 163.00, 162.14, 159.71, 148.88, 138.14, 130.44, 129.94, 128.21, 128.16, 127.47, 127.24, 127.01, 126.73, 43.51, 26.79. ESI‐HRMS m/z: C_18_H_17_N_7_: experimental 332.1616 [M + H]^+^, theoretical 332.1618 [M + H]^+^, Δ = 0.0002. Purity by HPLC (method B): 96.9%.

*N* ^7^‐Benzyl‐*N* ^5^‐cyclopropyl‐2‐phenyl‐[1,2,4]triazolo[1,5‐*a*[1,3,5]triazine‐5,7‐diamine (**25**). *N*‐Benzyl‐5‐phenoxy‐2‐phenyl‐[1,2,4]triazolo[1,5‐*a*[1,3,5]triazine‐7‐amine (**5**, 120 mg, 0.304 mmol), cyclopropyl (6 eq., 127 µl), 18 h, column chromatography eluent DCM, followed by DCM 95/MeOH 5, recrystallized from Et_2_O‐PE. Yield was 31% (34 mg, white solid). ^1^H NMR (600 MHz, DMSO‐d_6_) *δ* 9.05 (bs, 1H), 8.19 (d, *J* = 7.5 Hz, 2H), 7.77 (s, 1H), 7.60–7.54 (m, 4H), 7.45 (d, *J* = 7.7 Hz, 1H), 7.39 (t, *J* = 7.5 Hz, 2H), 7.32 (t, *J* = 7.4 Hz, 1H), 4.77–4.66 (m, 2H), 2.84 (bs, 1H), 0.79–0.69 (m, 2H), 0.64–0.53 (m, 2H). ^13^C NMR (151 MHz, DMSO‐*d*_6_) *δ* 163.22, 162.44, 159.79, 149.07, 138.94, 131.31, 130.58, 129.19, 128.80, 128.65, 127.84, 127.52, 127.11, 43.65, 24.29, 6.57. ESI‐HRMS m/z: C_20_H_19_N_7_: experimental 358.1772 [M + H]^+^, theoretical 358.1775 [M + H]^+^, Δ = 0.0003. Purity by HPLC (method B): 95.5%.

*N* ^7^‐Benzyl‐*N* ^5^‐cyclohexyl‐2‐phenyl‐[1,2,4]triazolo[1,5‐*a*[1,3,5]triazine‐5,7‐diamine (**26**). *N*‐Benzyl‐5‐phenoxy‐2‐phenyl‐[1,2,4]triazolo[1,5‐*a*[1,3,5]triazine‐7‐amine (**5**, 120 mg, 0.304 mmol), cyclohexylamine (3 eq., 105 µl), 12 h, column chromatography eluent DCM/MeOH (gradient, 95/5; 90/10), recrystallized from EtOAc‐Et_2_O. Yield was 48% (58 mg, white solid). ^1^H NMR (400 MHz, CD_3_OD) *δ* 8.16–8.11 (m, 2H), 7.55–7.18 (m, 8H), 4.74 (d, *J* = 6.4 Hz, 2H), 3.86 (bs, 1H), 2.14–1.57 (m, 5H), 1.51–1.10 (m,5H). ^13^C NMR (101 MHz, CD_3_OD) *δ* 162.94, 160.75, 159.70, 149.06, 138.17, 130.43, 129.95, 128.22, 128.17, 127.25, 127.10, 126.99, 126.72, 49.89, 43.44, 32.51, 32.38, 25.38, 24.94. ESI‐HRMS m/z: C_23_H_25_N_7_: experimental 400.2244 [M + H]^+^, theoretical 400.2244 [M + H]^+^, Δ = 0.0000. Purity by HPLC (method B): 95.1%.

*N* ^5^,*N* ^7^‐Dibenzyl‐2‐phenyl‐[1,2,4]triazolo[1,5‐*a*[1,3,5]triazine‐5,7‐diamine (**27**). *N*‐Benzyl‐5‐phenoxy‐2‐phenyl‐[1,2,4]triazolo[1,5‐*a*[1,3,5]triazine‐7‐amine (**5**, 120 mg, 0.304 mmol), benzylamine (3 eq., 100 µl), 12 h, column chromatography eluent DCM, followed by DCM 95/MeOH 5, recrystallized from EtOAc‐Et_2_O. Yield was 50% (61 mg, white solid). ^1^H NMR (600 MHz, DMSO‐*d*_6_) *δ* 9.10 (t, *J* = 6.3 Hz, 1H), 8.20–8.15 (m, 3H), 7.56 (q, *J* = 7.4, 6.8 Hz, 3H), 7.49 (d, *J* = 7.6 Hz, 1H), 7.43–7.26 (m, 9H), 4.70 (dd, *J* = 35.8, 6.2 Hz, 2H), 4.57 (d, *J* = 6.3 Hz, 2H). ^13^C NMR (151 MHz, DMSO‐*d*_6_) *δ* 163.56, 163.22, 161.66, 161.44, 159.83, 159.73, 149.46, 149.26, 140.55, 140.23, 138.93, 131.28, 130.59, 129.18, 128.81, 128.76, 128.68, 128.21, 127.97, 127.70, 127.67, 127.55, 127.18, 127.09, 44.36, 43.75. ESI‐HRMS m/z: C_24_H_21_N_7_: experimental 408.1931 [M + H]^+^, theoretical 408.1931 [M + H]^+^, Δ = 0.0000. Purity by HPLC (method B): 98.9%.

*N* ^7^‐Benzyl‐*N* ^5^‐isopropyl‐2‐(4‐methoxyphenyl)‐[1,2,4]triazolo[1,5‐*a*[1,3,5]triazine‐5,7‐diamine (**29**). *N*‐Benzyl‐2‐(4‐methoxyphenyl)‐5‐phenoxy‐[1,2,4]triazolo[1,5‐*a*[1,3,5]triazin‐7‐amine (**7**, 40 mg, 0.094 mmol), isopropylamine (3 eq., 24 µl), 18 h, column chromatography eluent DCM 98/EtOAc 2, recrystallized from EtOAc‐Et_2_O. Yield was 45% (16 mg, white solid). ^1^H NMR (600 MHz, CD_3_OD) *δ* 8.07 (d, *J* = 8.5 Hz, 2H), 7.41 (d, *J* = 7.4 Hz, 2H), 7.33 (t, *J* = 7.6 Hz, 2H), 7.26 (t, *J* = 7.4 Hz, 1H), 7.00 (d, *J* = 8.6 Hz, 2H), 4.74 (s, 2H), 4.21–4.16 (m, 1H), 3.85 (s, 3H), 1.34–1.10 (m, 6H). ^13^C NMR (151 MHz, CD_3_OD) *δ* 163.06, 161.60, 160.88, 148.97, 138.22, 128.27, 128.18, 127.49, 127.12, 126.99, 122.39, 113.57, 54.42, 43.43, 42.51, 21.25. ESI‐HRMS m/z: C_21_H_23_N_7_O: experimental 390.2036 [M + H]^+^, theoretical 390.2037 [M + H]^+^, Δ = 0.0001. Purity by HPLC (method B): 96.4%.

*N* ^5^,*N* ^7^‐Dibenzyl‐2‐(4‐methoxyphenyl)‐[1,2,4]triazolo[1,5‐*a*[1,3,5]triazine‐5,7‐diamine (**30**). *N*‐Benzyl‐2‐(4‐methoxyphenyl)‐5‐phenoxy‐[1,2,4]triazolo[1,5‐*a*[1,3,5]triazin‐7‐amine (**7**, 107 mg, 0.252 mmol), benzylamine (7 eq., 193 µl), 18 h, column chromatography eluent DCM 99/MeOH 1, recrystallized from EtOAc‐Et_2_O. Yield was 86% (94 mg, white solid). ^1^H NMR (400 MHz, DMSO‐*d*_6_) *δ* 8.98 (t, *J* = 6.4 Hz, 1H), 8.12–8.01 (m, 3H), 7.47–7.19 (m, 10H), 7.07 (d, *J* = 8.8 Hz, 2H), 4.64 (dd, *J* = 24.3, 6.2 Hz, 2H), 4.51 (d, *J* = 6.3 Hz, 2H), 3.83 (s, 3H). ^13^C NMR (101 MHz, DMSO‐*d*_6_) *δ* 163.17, 161.40, 161.27, 159.65, 149.22, 140.28, 138.99, 128.81, 128.75, 128.67, 128.20, 127.95, 127.68, 127.53, 127.10, 123.69, 114.59, 55.74, 44.35, 43.72. ESI‐MS (acetonitrile) *m/z:* 438.3 [M + H]^+^, 460.2 [M + Na]^+^, and 476.2 [M + K]^+^. Purity by HPLC (method B): 95.7%.

### 3.2 Binding at Human A_1_, A_2A_, A_2B_, and A_3_ AR

Pharmacological experiments were conducted according to previously reported methodologies [54]. Membrane preparations for radioligand binding studies were obtained from CHO cells stably expressing the human adenosine receptor subtypes, generated as described earlier [54]. Briefly, membrane fractions were isolated using a two‐step centrifugation protocol. An initial centrifugation at 1000 × *g* was performed to remove the nuclei and cellular debris, followed by centrifugation of the resulting supernatant at 100,000 × *g* to collect crude membranes. The membrane pellet was resuspended in the appropriate binding buffer, rapidly frozen in liquid nitrogen, and stored at −80 °C until use. Test compounds were initially dissolved in DMSO and subsequently diluted with the assay buffer to the required concentrations. At least six concentrations were evaluated for each compound, while the final DMSO concentration in the assays did not exceed 2% (v/v). Radioligand binding experiments at human AR employed 1 nM [^3^H]CCPA for A_1_ AR, [^3^H]NECA at a concentration of 10 nM for A_2A_, and 1 nM [^3^H]HEMADO for A_3_ ARs. Nonspecific binding was assessed using 1 mM theophylline in A_1_ AR assays and 100 μM R‐PIA in assays performed with [^3^H]HEMADO. Following a 3 h incubation period at room temperature, samples were filtered and washed as previously described [54, 55, 56]. Inhibition constants (*K*_i_) were derived from competition binding experiments using the SCTFIT software package. The reported values represent the mean of 3–6 independent determinations and are presented with their corresponding 95% confidence intervals.

#### 3.2.1 Adenylyl Cyclase Activity

Functional assays at the hA_2B_ AR were carried out through adenylyl cyclase experiments following previously described protocols with minor adjustments [55, 57]. Briefly, homogenates of hA_2_B‐CHO cells were centrifuged at high speed, and the resulting membrane pellet was resuspended in Tris/HCl buffer (50 mM, pH 7.4) and immediately used in the assay. Compound activity was evaluated by measuring the inhibition of NECA‐induced adenylyl cyclase activity (NECA, 5 μM). Membrane preparations were incubated with approximately 150,000 cpm of [α‐^32^P]ATP in the reaction mixture for 20 min in the absence of EGTA and NaCl [58]. IC_50_ values were determined by fitting the data to the Hill equation and are reported as the mean of 3–6 independent replicates. The hill slopes were close to 1 in all cases. Dissociation constants (*K*_i_) were then calculated with the Cheng and Prusoff equation [57].

### 3.3 Molecular Modeling

The following experimental structures of the AR in their inactive, antagonist‐bound state were retrieved from the PDB: 5N2S for hA_1_ AR (X‐ray diffraction, 3.3 Å) [38], 5NM4 for hA_2A_ AR (room‐temperature serial crystallography, 1.7 Å) [39], and 9EHS for hA_3_ AR (cryo‐electron microscopy, 3.2 Å) [40]. Protein structures were prepared using the Structure Preparation tool of MOE (Molecular Operating Environment (MOE), 2024.0601, Chemical Computing Group ULC, 910‐1010 Sherbrooke St. W., Montreal, QC H3A 2R7, **2026**). In particular, fusion partners and chimeric regions were deleted, and the missing IL3 was reconstructed in the case of hA_2A_ and hA_3_ AR, where the chimeric construct replaces IL3. Missing atoms were added, and for residues modeled in alternate conformations, the state with the highest occupancy was retained. Thermostabilizing and construct mutations were reverted to wild‐type residues. Protonation states were assigned with the Protonate3D tool at pH 7.4 and a temperature of 310 K. Hydrogen atoms and back‐mutated residues were energy‐minimized with the AMBER:EHT force field after computation of the partial charges.

The sodium ion known to act as an allosteric modulator of class A GPCRs [59], together with the two water molecules of its first solvation shell, as resolved in the hA_2A_ AR structure 5NM4, was retained. The same ion and water molecules were also transferred to the hA_1_ and hA_3_ AR structures, for which these coordinates were not determined, by superposition of the receptor structures, followed by minimization of the sodium ion and of the water molecules in place.

Ligands were manually drawn in MOE, assigned their predominant protonation state at pH 7.4, and energy‐minimized with the MMFF94x force field. Docking was performed with GOLD [41] using the ChemScore scoring function [42]. The conformational search was focused on a sphere of 11 Å radius centered on the center of mass of Phe171/Phe168/Phe168 and Asn254/Asn253/Asn250, respectively, in hA_1_, hA_2A,_ and hA_3_ AR.

A consensus pharmacophore was built in MOE from the antagonists co‐crystallized or co‐bound to the three PDB structures. This resulted in a 2.3 Å‐radius aromatic feature in the proximity of Phe171/Phe168/Phe168 and in a 2.3 Å‐radius hydrogen‐bond acceptor sphere in the proximity of Asn254/Asn253/Asn250. The pharmacophore was used for pose filtering.

Poses were minimized employing the docking tool of MOE, exclusively for the refinement step: induced‐fit settings were used with side‐chain tethering (default settings), and GBVI/WSA dG was used for rescoring.

Poses were filtered with the pharmacophore model, and poses displaying positive van der Waals or electrostatic interaction potentials were discarded; both terms were computed with an in‐house SVL script exploiting MOE functionalities. One pose per compound was selected whenever possible, namely, the one with the best GBVI/WSA dG, after discarding unreasonable binding modes (i.e., not placing a hydrophobic substituent deep into the orthosteric pocket or not placing the exocyclic NH groups close to Asn254/Asn253/Asn250, as typical for AR antagonists). Per‐residue hydrophobic and electrostatic interaction energies were computed between each ligand, in its selected pose, and the amino acids of the receptor‐binding site, using in‐house SVL scripts and MOE functionalities. The results were plotted as heat maps generated with Python. The per‐residue analysis and the images of the selected poses were assembled into videos produced with in‐house Python scripts, employing UCSF Chimera [60] for molecular rendering.

### 3.4 Extracellular Recordings

All experimental procedures were carried out following the Italian regulations for the care and use of laboratory animals (EU Directive 2010/63/EU) and were approved by appropriate institutional and state authorities of the University of Florence (authorization number: 17E9C.N.SEO). In compliance with Italian law and EU directives, all efforts were made to minimize animal suffering and to fulfill the 3Rs principle. All animals were located in a temperature‐controlled room (22 ± 1 °C) in groups of two‐five per cage, with food and water ad libitum, and with a 12 h light/dark cycle.

#### 3.4.1 Slice Preparation

Experiments were carried out on rat hippocampal slices, prepared as previously described [50]. Male Wistar rats (Envigo, Italy, 150–200 g body weight) were deeply anesthetized with ether and decapitated with a guillotine. The hippocampus was rapidly removed and placed in ice‐cold oxygenated (95% O_2_–5% CO_2_) artificial cerebral spinal fluid (aCSF) of the following composition (mM): NaCl 125, KCl 3, NaH_2_PO_4_ 1.25, MgSO_4_ 1, CaCl_2_ 2, NaHCO_3_ 25, and D‐glucose 10. Slices (400 µm nominal thickness) were cut with a McIlwain tissue chopper (The Mickle Lab. Engineering, Co. Ltd., Gomshall, U.K.) and maintained in oxygenated aCSF for at least 1 h at room temperature. A single slice was then placed on a nylon mesh, completely submerged in a small chamber (0.8 mL), and superfused with oxygenated aCSF (31–32 °C) at a constant flow rate of 2 mL/min. The treated solutions reached the preparation in 60 s, and this delay was considered in our calculations.

#### 3.4.2 Extracellular Recording

Test pulses (80 µs, 0.066 Hz) were delivered through a bipolar nichrome electrode positioned in the CA1 region of the stratum radiatum. Evoked fEPSPs were recorded with glass microelectrodes (2–10 M′Ω, Clark Electromedical Instruments, Panghourne, U.K.) filled with 150 mM NaCl and situated at the CA1 dendritic level. Responses were amplified (BM 622, Mangoni, Pisa, Italy), digitized (sample rate, 33.33 kHz), low‐pass filtered (10 kHz), and stored for later analysis using LTP software (version 2.30D) [61]. The test stimulus pulse was adjusted to obtain fEPSPs whose amplitudes were 40%–50% of the maximum and were maintained constant throughout the experiment. The amplitude of the fEPSP was routinely measured and expressed as the percentage of the average amplitude of the potentials measured during the 5 min preceding exposure of the hippocampal slices to in vitro ischemia. Since no appreciable differences between the fEPSP initial slope and amplitude were measured, only the value of the amplitude was reported in the figures.

#### 3.4.3 Application of OGD Protocol

In vitro OGD was obtained by perfusing the slice for 7 or 30 min with glucose‐free aCSF gassed with nitrogen (95% N_2_–5% CO_2_). At the end of the ischemic period, slices were superfused with normal glucose‐containing, oxygenated aCSF. In the first protocol, the selective A_3_AR antagonists were applied 10 min before, during 30 min of OGD, and 5 min after the end of the ischemic episode; in the second one, the compounds were applied during the last 3 min of the 7 min‐long OGD insult and 5 min after the OGD protocol in oxygenated aCSF. In all conditions, fEPSP amplitude was monitored during all the experiment. AD was recorded as negative extracellular d.c. shifts induced by OGD. This phenomenon is considered a sign that the cells around the tip of the glass electrode were depolarized [49]. AD latency was calculated from the beginning of the OGD insult and was expressed in min, while AD amplitude was calculated at the maximal negativity peak and was expressed in mV. In this work, the AD amplitude values were expressed as positive values.

#### 3.4.4 Drugs

The selective A_3_ AR antagonist compound **15** was dissolved in dimethyl sulfoxide (DMSO) prior to testing. Stock solutions of 1000–10,000 times the desired final concentrations were stored at −20 °C. The final concentration of DMSO (0.05% and 0.1% in aCSF) used in our experiments did not affect either the fEPSP slope or amplitude in all different protocols applied.

#### 3.4.5 Statistical Analysis

Data were expressed as the mean ± SEM (standard error of the mean). Kolmogorov–Smirnov normality test was performed to check data distribution: all data reported in the present research are normally distributed. Two‐tailed Student's unpaired *t*‐tests were performed to determine statistical significance (set at *p* < 0.05) between groups. Data were analyzed using “GraphPad Prism” (GraphPad Software, San Diego, CA, USA) software.

## 4 Conclusion

In this study, we designed and synthesized a new series of 2,5,7‐trisubstituted [1,2,4]triazolo[1,5‐*a*[1,3,5]triazines as adenosine receptor ligands, exploring the impact of replacing the classical C2‐furyl group with phenyl and 4‐methoxyphenyl moieties. Systematic SAR analysis demonstrated that modulation of the substituents at C2, C5, and C7 profoundly influences affinity and selectivity toward human adenosine receptor subtypes. In particular, incorporation of aromatic groups at C2, combined with appropriate amino substituents at C5 and C7, enabled the identification of highly potent and selective hA_3_ AR antagonists.

Among the synthesized compounds, derivative **15** emerged as the most potent hA_3_ AR ligand of the series (*K*_i_ = 0.485 nM), displaying excellent selectivity over the other AR subtypes. Furthermore, compound **30** exhibited the highest selectivity profile, exceeding 2800‐fold selectivity for hA_3_ AR versus hA_1_ AR and hA_2A_ AR. These findings establish the triazolo‐triazine scaffold as a versatile platform for the development of selective hA_3_ AR antagonists and highlight the beneficial effect of replacing the 2‐furyl moiety with phenyl‐based substituents.

From a structural point of view, molecular docking at the experimental structures of the three receptor subtypes provided a consistent rationale for the observed profiles. At hA_3_ AR, the triazolotriazine core is anchored by a hydrogen bond to Asn250 and by a π–π stacking interaction with Phe168, while the aromatic substituents at C2 and C5 can be exchanged between the deep and the outer regions of the cavity, giving rise to two alternative and similarly ranked binding modes. Subtype preference appears to be governed by two features unique to hA_3_ AR: the lipophilic rim formed by Val169 and Ile253 at the entrance of the binding cleft, which accommodates the apolar C7 substituents, and the more spacious bottom of the pocket generated by Ser181 and Ser247, which better tolerates bulky six‐membered aromatic groups at C2. At hA_2A_ AR, conversely, the intra‐receptor Glu169–His264 salt bridge restricts the ZM 241385‐like placement to the derivatives bearing an unsubstituted amino group at C7, in agreement with the drop in hA_2A_ AR affinity observed as soon as this position is substituted. Discriminating between the two binding modes predicted at hA_3_ AR will require molecular dynamics simulations and the synthesis of conformationally constrained analogs.

Importantly, the translational relevance of this chemical series was supported by in vitro studies in hippocampal slices subjected to oxygen‐glucose deprivation, where compound **15** delayed AD and markedly preserved synaptic function following ischemic insult. Overall, this work identifies novel hA_3_ AR antagonists with an attractive combination of potency, selectivity, and neuroprotective activity, providing promising lead compounds for the development of new therapeutic strategies targeting cerebral ischemia and other hA_3_ AR‐related pathological conditions.

## Conflicts of Interest

The authors declare no conflicts of interest.

## Supporting information

Supplementary Material

Supplementary Material

Supplementary Material

Supplementary Material

## Data Availability

The data that support the findings of this study are available in the Supporting Information of this article.
