# Supplementary material for: Tuning the Triazolotriazine Scaffold Toward Selective A3 Adenosine Receptor Antagonists: From Molecular Recognition to Neuroprotection
Source: ChemMedChem. 2026 Oct 18;21(20):e70528. doi: 10.1002/cmdc.70528 (PMC13677798; doi:10.1002/cmdc.70528)
Supplement: Supplementary file 1 [file CMDC-21-e70528-s003.pdf]

Tuning the Triazolotriazine Scaffold toward Selective A_3 Adenosine Receptor Antagonists: from Molecular Recognition to Neuroprotection

Veronika Kost,[a] Filippo Prencipe,[a] Federica Panozzi,[b] Veronica Salmaso,[c] Sonja Kachler,[d] Karl-Norbert Klotz,[e] Elisabetta Coppi,[b] Simone Adorinni,[a] Stefano Moro,[c] Anna Maria Pugliese,[b] Stephanie Federico,*[a] Giampiero Spalluto,[a] and Tatiana Da Ros*[a]

[a] Department of Chemical and Pharmaceutical Sciences
University of Trieste
Via Licio Giorgieri 1, 34127, Trieste, Italy
E-mail: sfederico@units.it, daros@units.it

[b] Department of Neuroscience, Psychology, Drug Research and Child Health (NEUROFARBA)
University of Florence
Viale Pieraccini 6, 50139, Florence, Italy

[c] Molecular Modeling Section (MMS), Department of Pharmaceutical and Pharmacological Sciences
University of Padua
Via Marzolo 5, 35131, Padua, Italy

[d] Rudolf-Virchow-Zentrum – Center for Integrative and Translational Bioimaging
University of Würzburg
Josef-Schneider-Str. 2, 97080 Würzburg (Germany)

[e] Institut für Pharmakologie und Toxikologie
University of Würzburg
Versbacher Str. 9, 97078 Würzburg (Germany)

SUPPORTING INFORMATION

N-methyl-5-phenoxy-2-phenyl-[1,2,4]triazolo[1,5-a][1,3,5]triazine-7-amine **2** 3
N-cyclopropyl-5-phenoxy-2-phenyl-[1,2,4]triazolo[1,5-a][1,3,5]triazine-7-amine **3** 6
N-cyclohexyl-5-phenoxy-2-phenyl-[1,2,4]triazolo[1,5-a][1,3,5]triazine-7-amine **4** 9
N-benzyl-5-phenoxy-2-phenyl-[1,2,4]triazolo[1,5-a][1,3,5]triazine-7-amine **5** 12
N-benzyl-2-(4-methoxyphenyl)-5-phenoxy-[1,2,4]triazolo[1,5-a][1,3,5]triazine-7-amine **7** 15
N5-methyl-2-phenyl-[1,2,4]triazolo[1,5-a][1,3,5]triazine-5,7-diamine **8** 18
N5-cyclopropyl-2-phenyl-[1,2,4]triazolo[1,5-a][1,3,5]triazine-5,7-diamine **9** 21
N5,N7-dimethyl-2-phenyl-[1,2,4]triazolo[1,5-a][1,3,5]triazine-5,7-diamine **12** 24
N5-cyclopropyl-N7-methyl-2-phenyl-[1,2,4]triazolo[1,5-a][1,3,5]triazine-5,7-diamine **13** 27
N5-cyclohexyl-N7-methyl-2-phenyl-[1,2,4]triazolo[1,5-a][1,3,5]triazine-5,7-diamine **14** 30
N5-benzyl-N7-methyl-2-phenyl-[1,2,4]triazolo[1,5-a][1,3,5]triazine-5,7-diamine **15** 33
N7-cyclopropyl-N5-methyl-2-phenyl-[1,2,4]triazolo[1,5-a][1,3,5]triazine-5,7-diamine **16** 36
N5,N7-dicyclopropyl-2-phenyl-[1,2,4]triazolo[1,5-a][1,3,5]triazine-5,7-diamine **17** 39
N5-cyclohexyl-N7-cyclopropyl-2-phenyl-[1,2,4]triazolo[1,5-a][1,3,5]triazine-5,7-diamine **18** 42
N5-benzyl-N7-cyclopropyl -2-phenyl-[1,2,4]triazolo[1,5-a][1,3,5]triazine-5,7-diamine **19** 45
N7-cyclohexyl-N5-methyl -2-phenyl-[1,2,4]triazolo[1,5-a][1,3,5]triazine-5,7-diamine **20** 48
N7-cyclohexyl-N5-cyclopropyl-2-phenyl-[1,2,4]triazolo[1,5-a][1,3,5]triazine-5,7-diamine **21** 51
N5,N7-dicyclohexyl-2-phenyl-[1,2,4]triazolo[1,5-a][1,3,5]triazine-5,7-diamine **22** 54
N7-cyclohexyl-N5-benzyl-2-phenyl-[1,2,4]triazolo[1,5-a][1,3,5]triazine-5,7-diamine **23** 57
N7-benzyl-N5-methyl-2-phenyl-[1,2,4]triazolo[1,5-a][1,3,5]triazine-5,7-diamine **24** 60
N7-benzyl-N5-cyclopropyl-2-phenyl-[1,2,4]triazolo[1,5-a][1,3,5]triazine-5,7-diamine **25** 63
N7-benzyl-N5-cyclohexyl-2-phenyl-[1,2,4]triazolo[1,5-a][1,3,5]triazine-5,7-diamine **26** 66
N5,N7-dibenzyl-2-phenyl-[1,2,4]triazolo[1,5-a][1,3,5]triazine-5,7-diamine **27** 69
N7-benzyl-N5-isopropyl-2-(4-methoxyphenyl)-[1,2,4]triazolo[1,5-a][1,3,5]triazine-5,7-diamine **29** 72

N^5,N^7-DIBENZYL-2-(4-METHOXYPHENYL)-[1,2,4]TRIAZOLO[1,5-A][1,3,5]TRIAZINE-5,7-DIAMINE **30** 75

N-methyl-5-phenoxy-2-phenyl-[1,2,4]triazolo[1,5-*a*][1,3,5]triazine-7-amine **2**

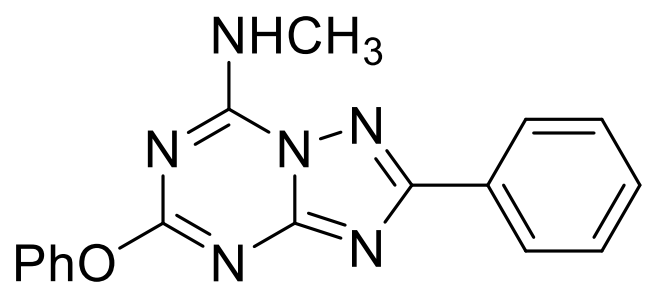

Chemical Formula: $C_{17}H_{14}N_6O$
Molecular Weight: 318.33

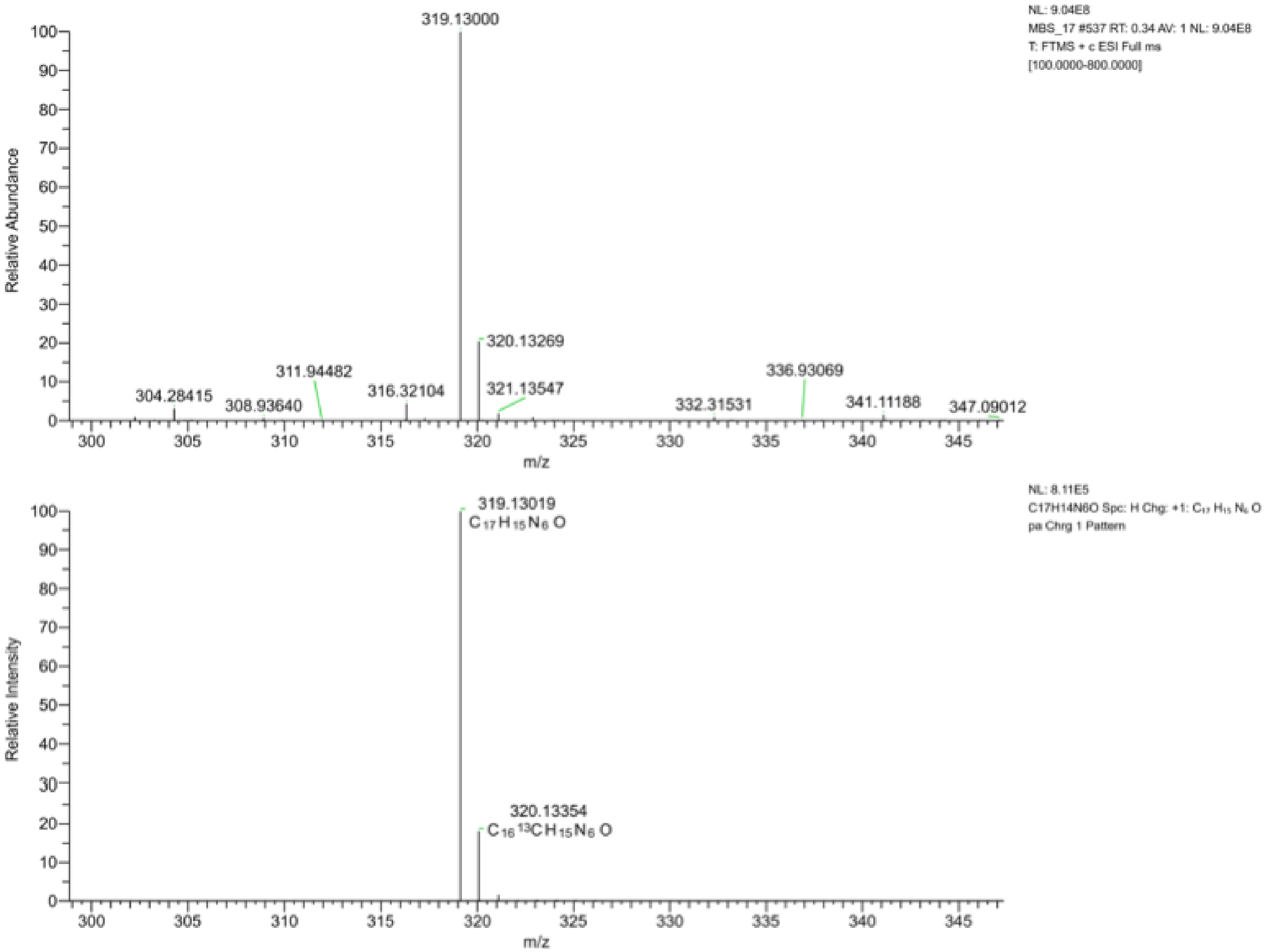

Figure S1. HRMS (ESI) spectrum of **Compound 2.**

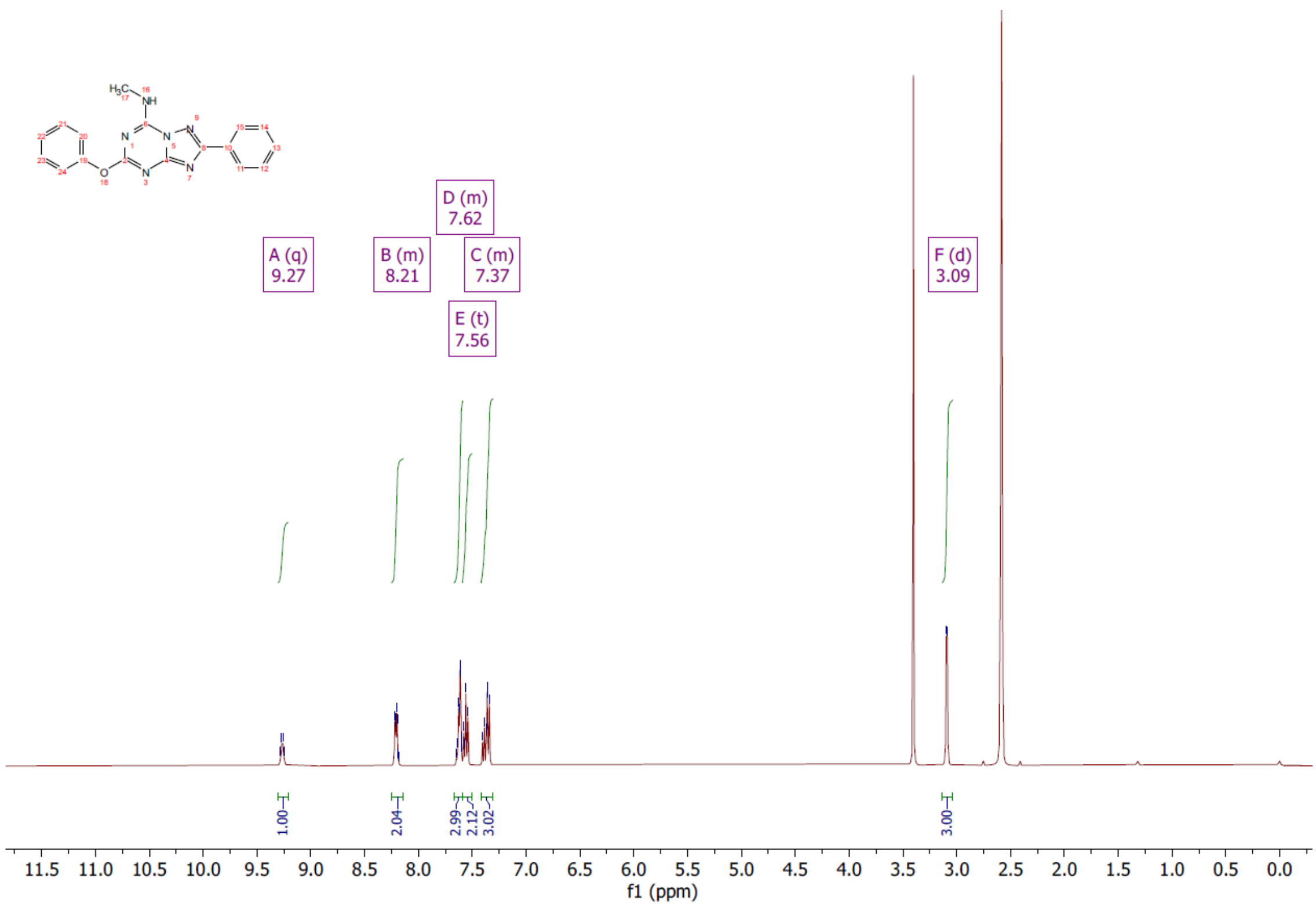

Figure S2. ^{1}H NMR spectrum of **Compound 2.**

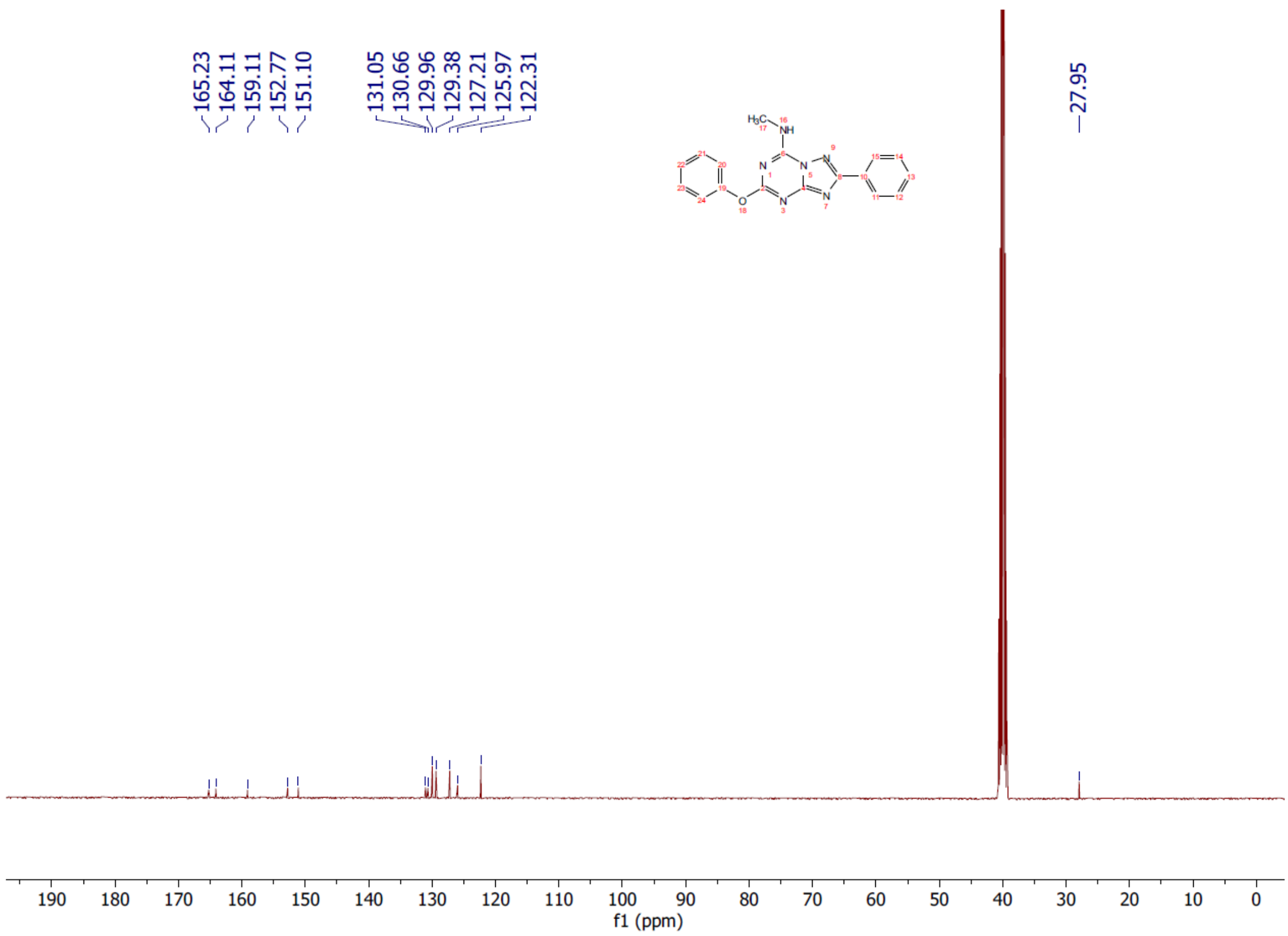

Figure S3. ^{13}C NMR spectrum of **Compound 2.**

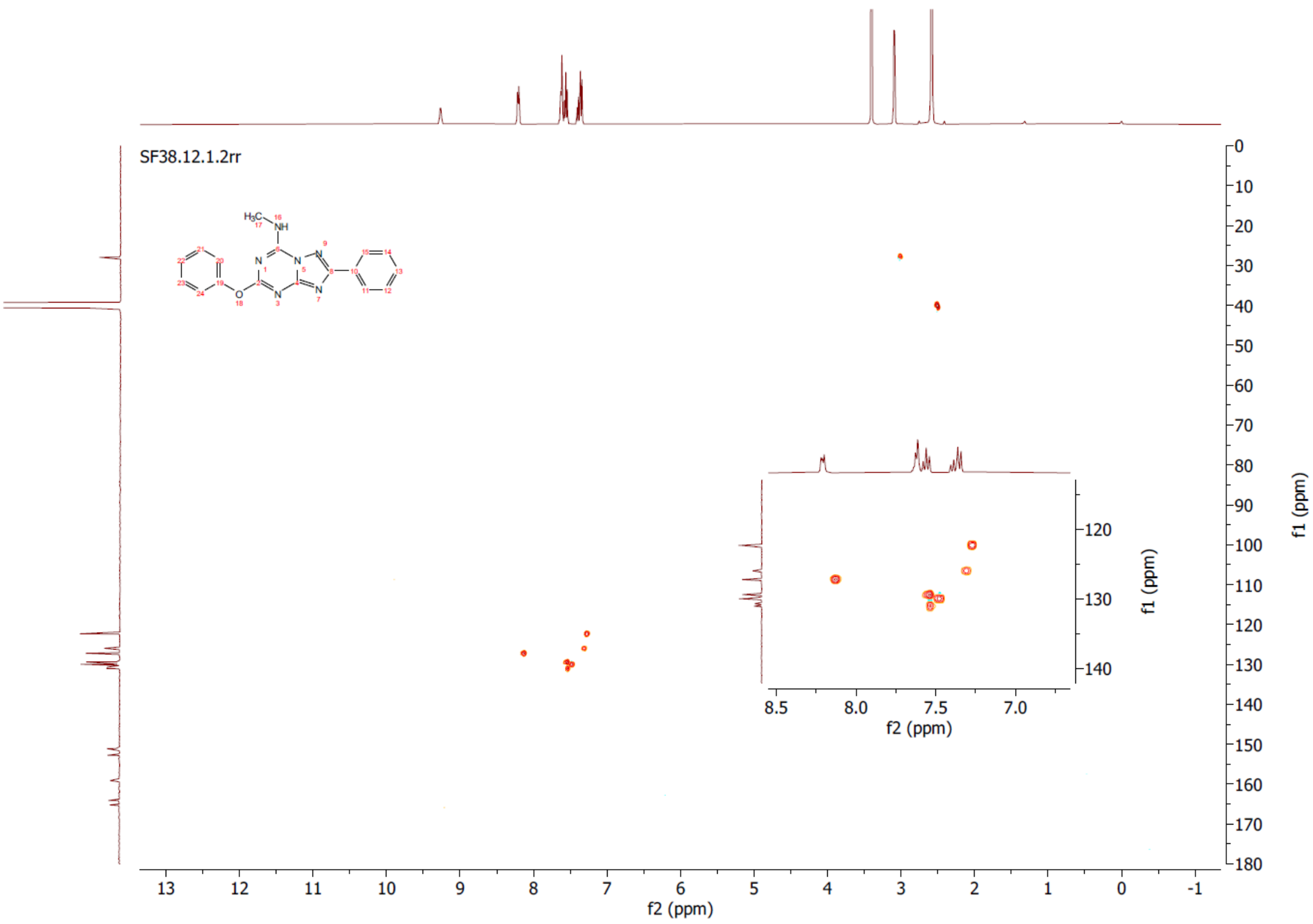

Figure S4. HSQC ^{1}H-^{13}C NMR spectrum of **Compound 2**

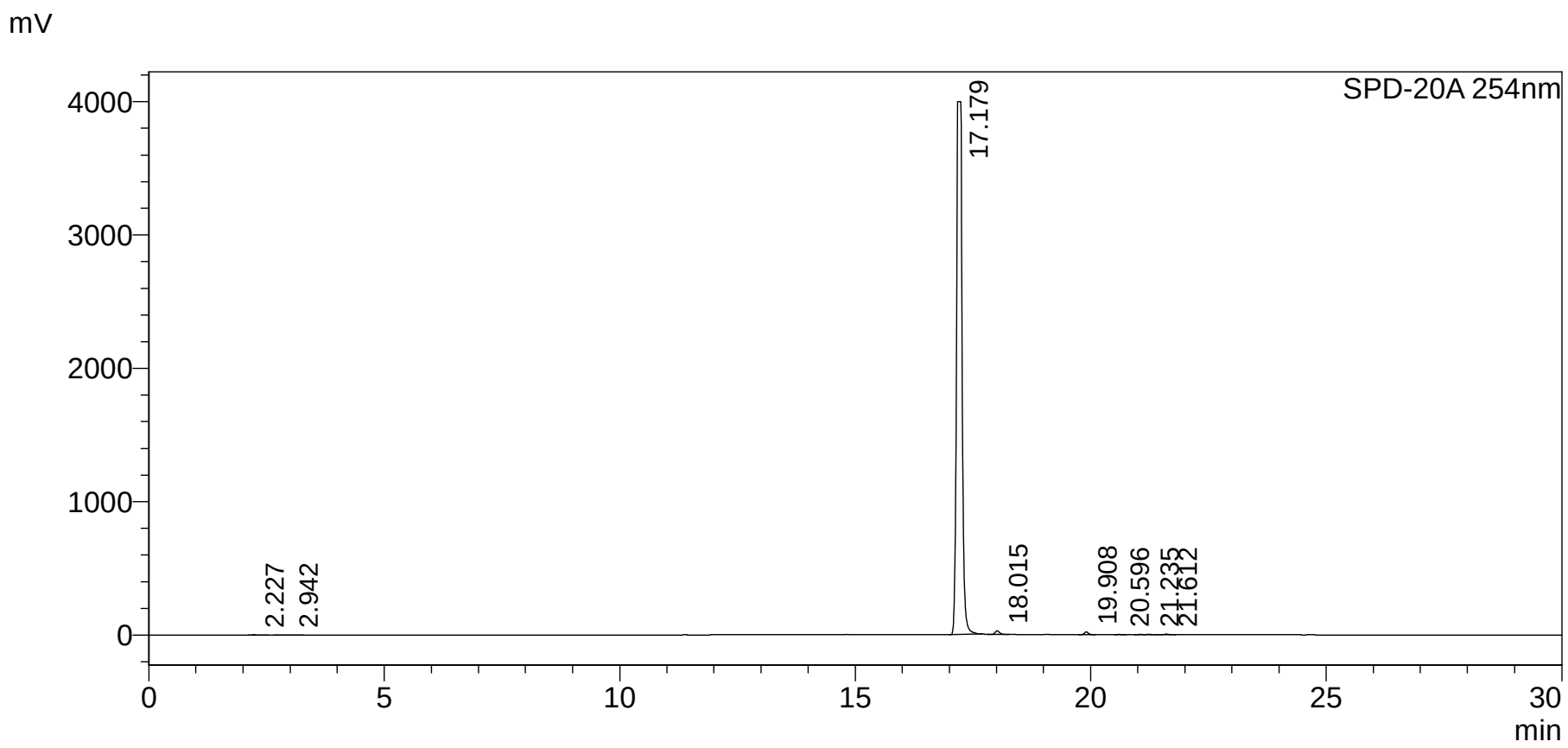

<Peak Table>

SPD-20A 254nm

Peak#	Ret. Time	Area	Area%
1	2.227	14247	0.043
2	2.942	6055	0.018
3	17.179	32954479	98.854
4	18.015	164444	0.493
5	19.908	128487	0.385
6	20.596	15058	0.045
7	21.235	26164	0.078
8	21.612	27491	0.082
Total		33336424	100.000

Figure S5. HPLC Chromatogram of **Compound 2**

N-cyclopropyl-5-phenoxy-2-phenyl-[1,2,4]triazolo[1,5-*a*][1,3,5]triazine-7-amine **3**

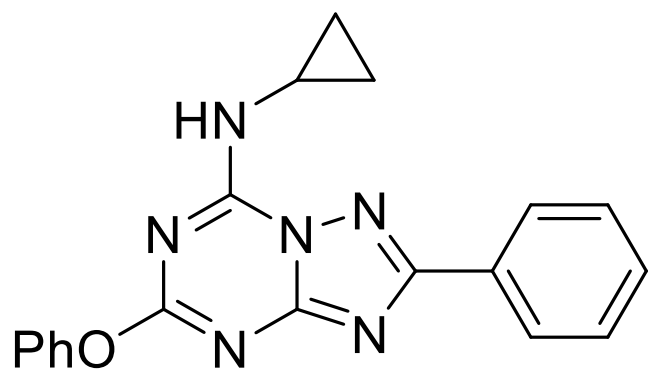

Chemical Formula: $C_{19}H_{16}N_6O$
Molecular Weight: 344,37

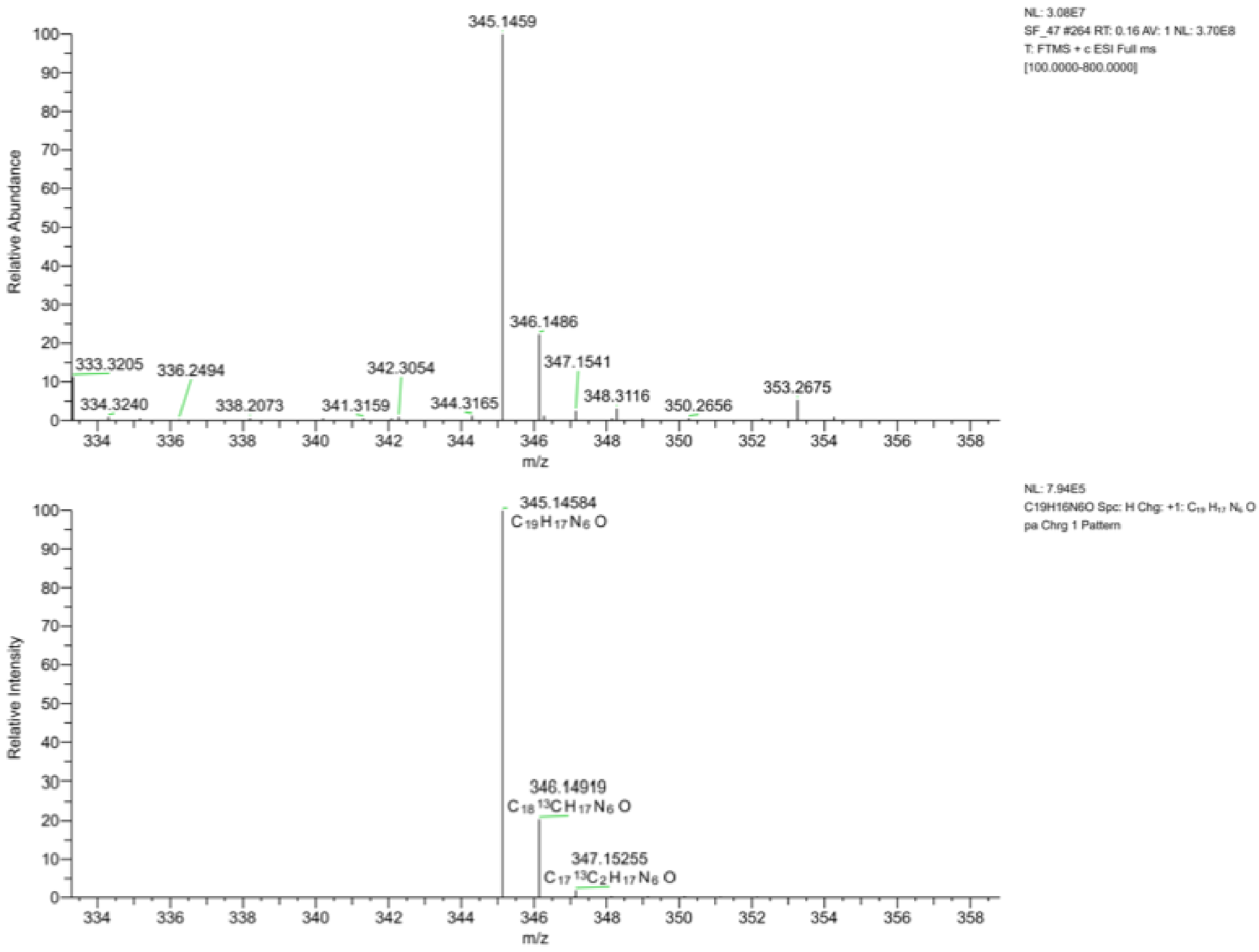

Figure S6. HRMS (ESI) spectrum of **Compound 3**.

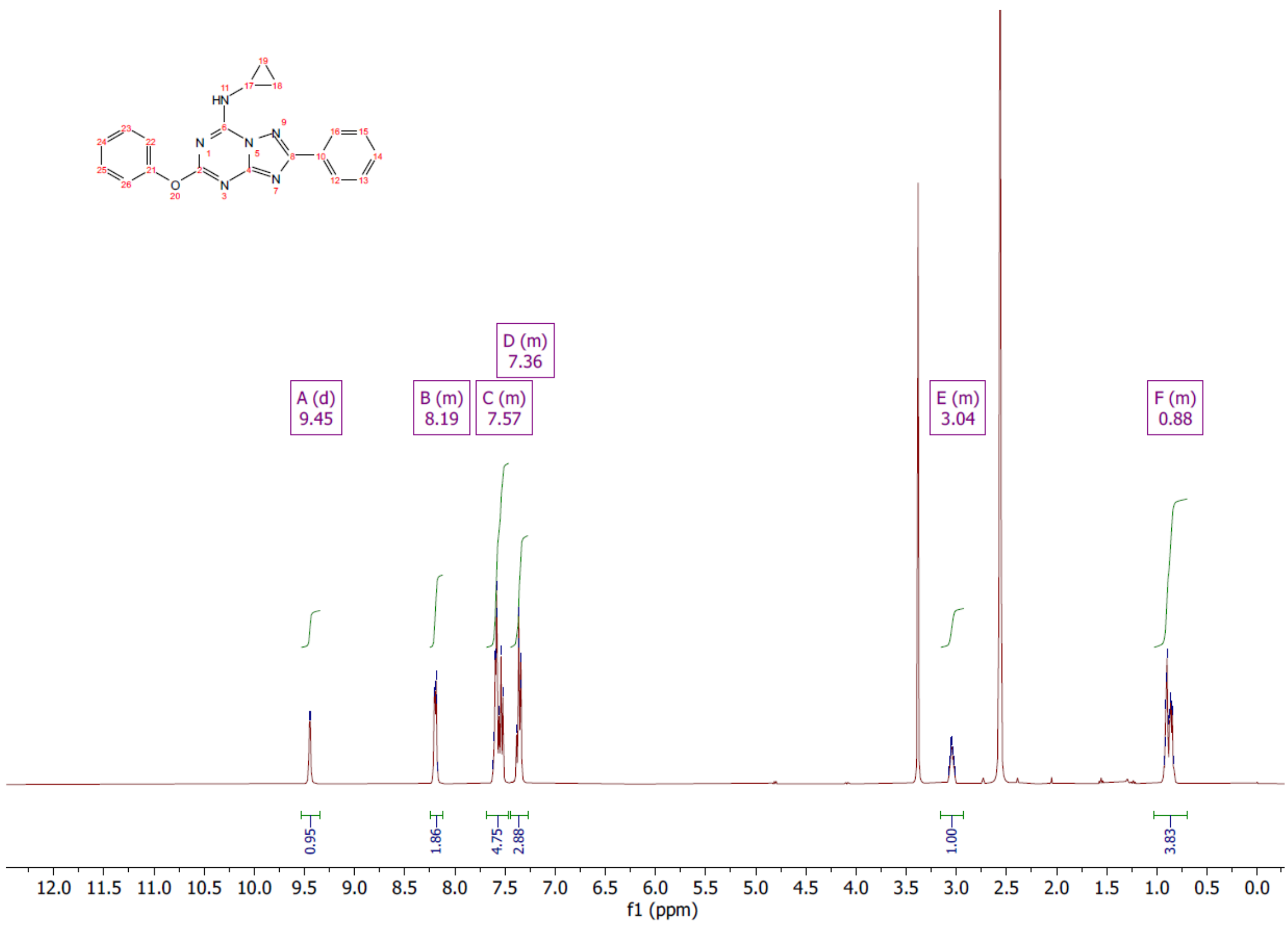

Figure S7. ^{1}H NMR spectrum of **Compound 3** (DMSO-d_6).

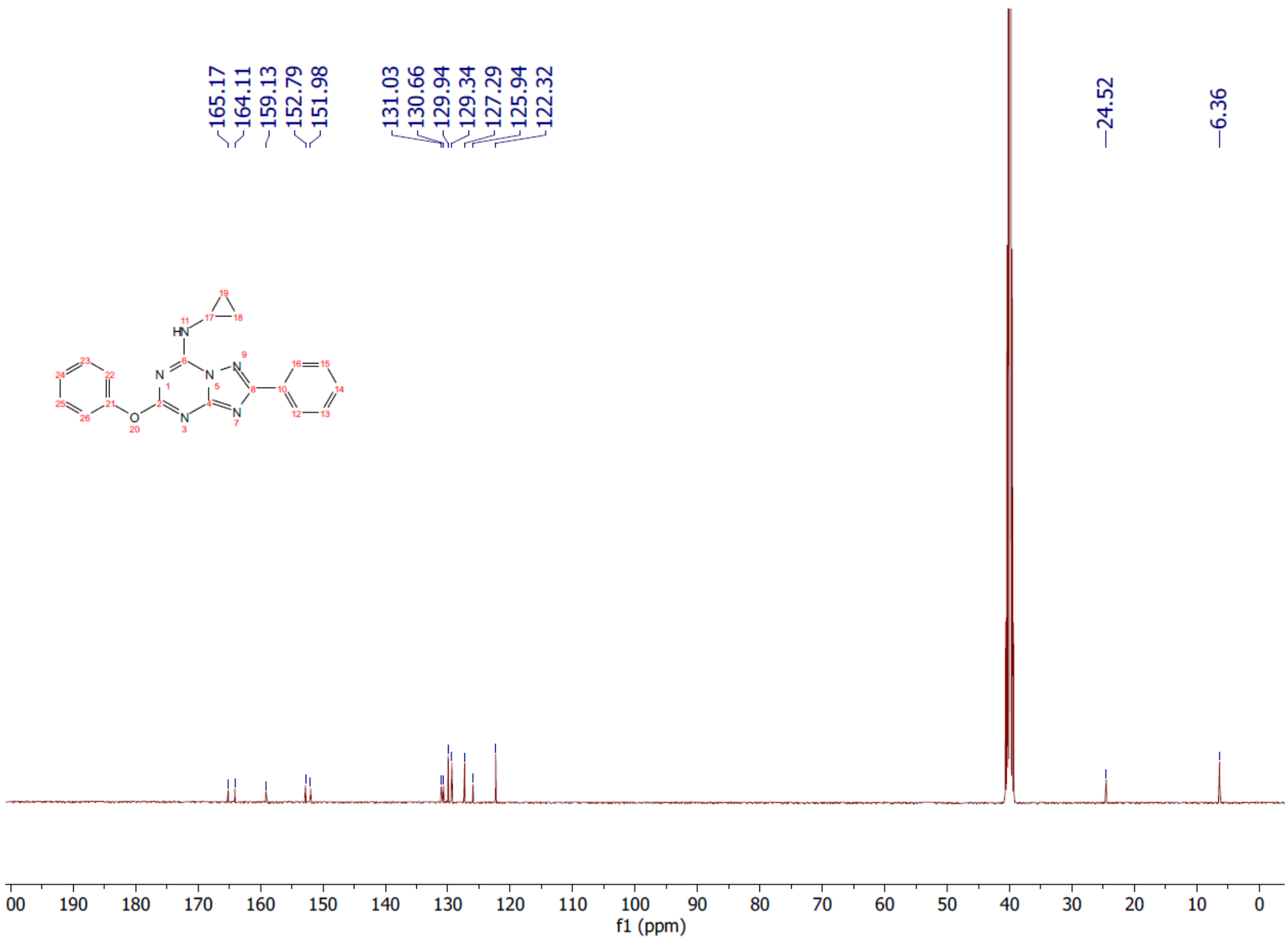

Figure S8. ^{13}C NMR spectrum of **Compound 3** (DMSO-d_6).

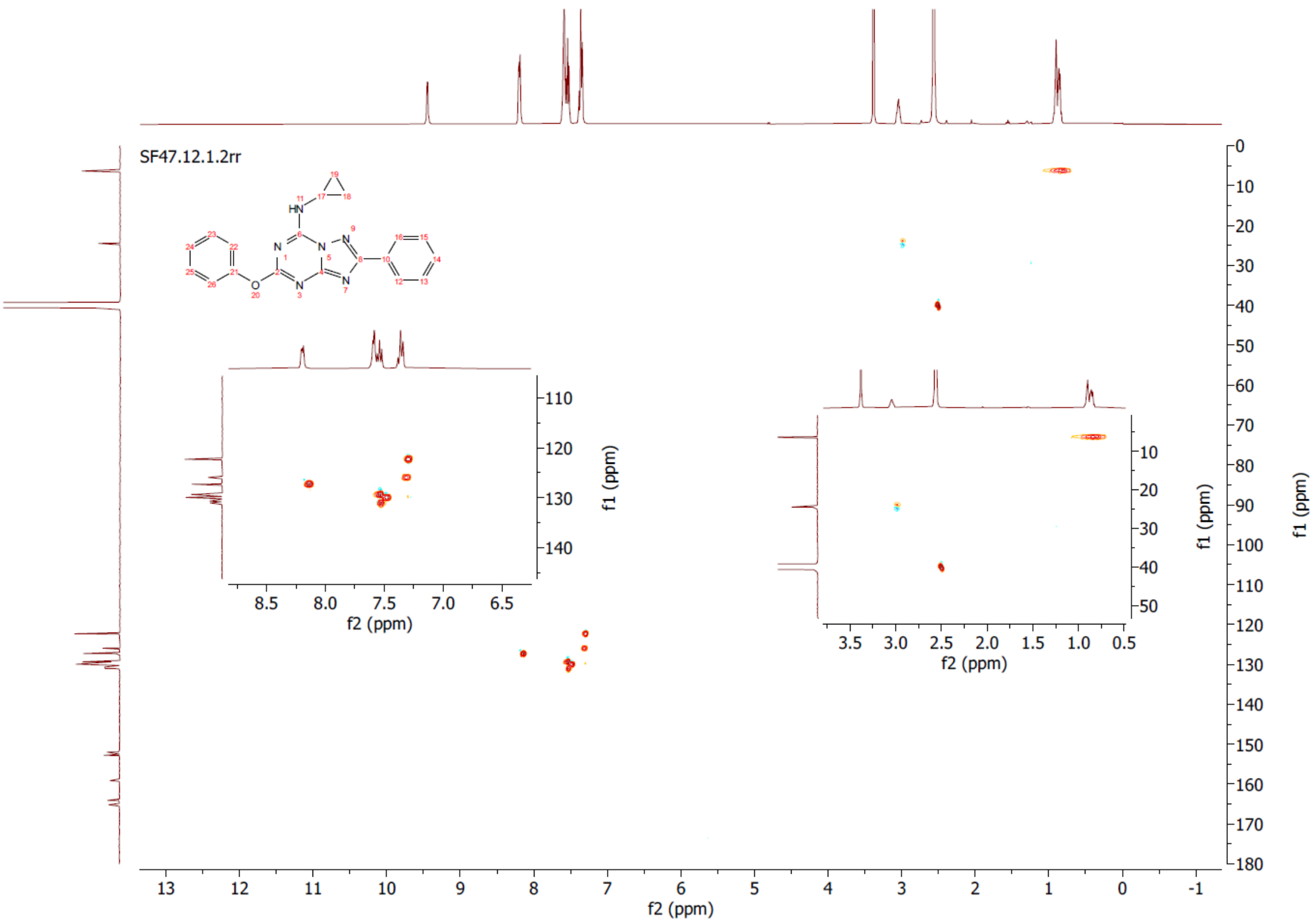

Figure S9. HSQC ^{1}H-^{13}C NMR spectrum of **Compound 3** (DMSO-d_6).

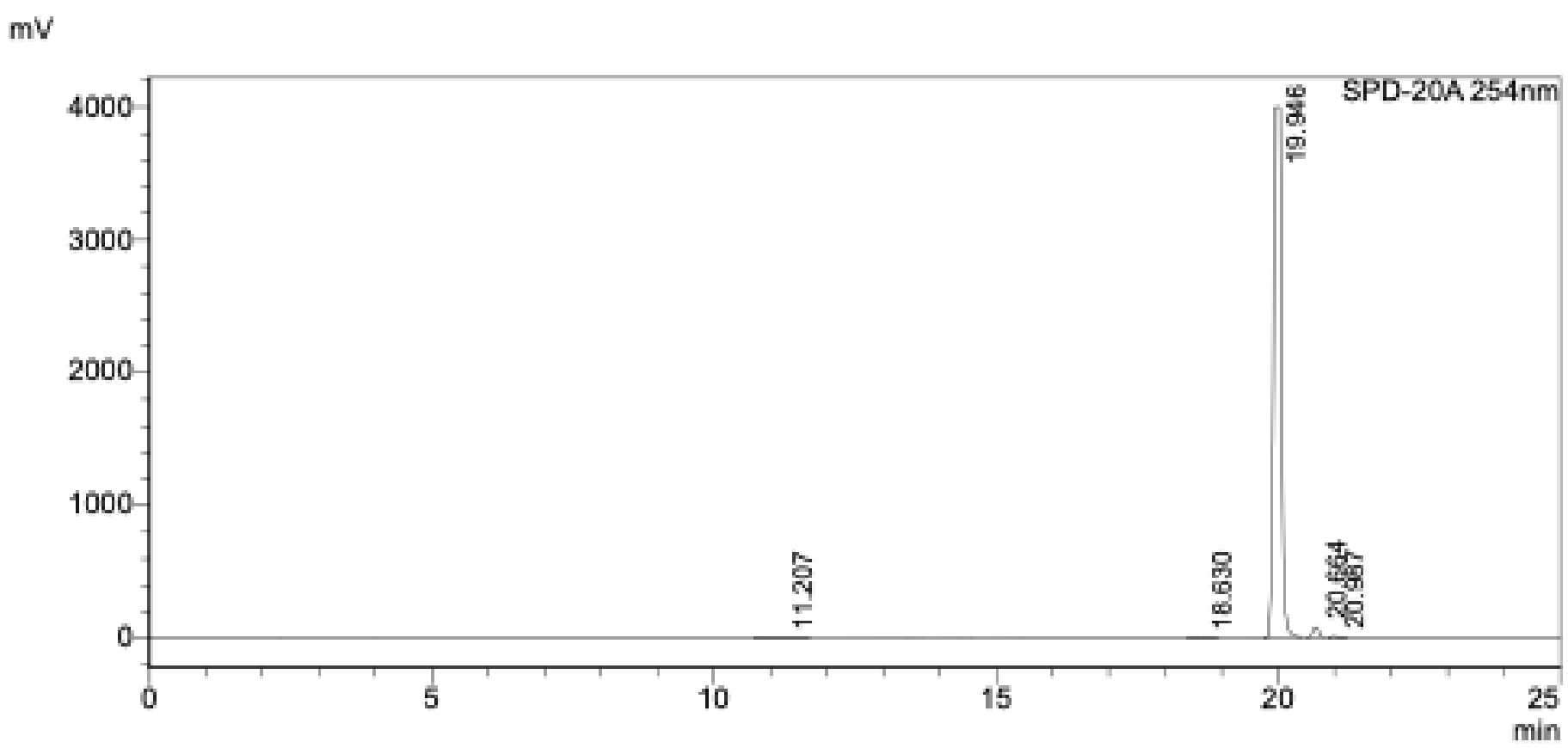

<Peak Table>

SPD-20A 254nm

Peak#	Ret. Time	Area	Area%
1	11.207	4943	0.013
2	18.630	41187	0.104
3	19.946	38784254	98.275
4	20.664	537482	1.362
5	20.987	97058	0.246
Total		39464924	100.000

Figure S10. HPLC Chromatogram of **Compound 3.**

N-cyclohexyl-5-phenoxy-2-phenyl-[1,2,4]triazolo[1,5-*a*][1,3,5]triazine-7-amine **4**

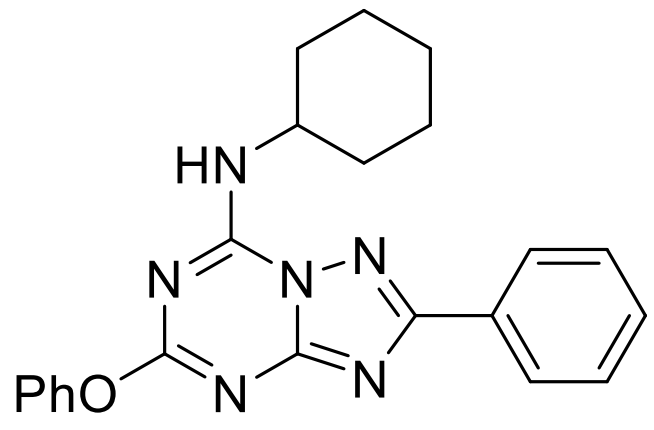

Chemical Formula: $C_{22}H_{22}N_6O$
Molecular Weight: 386,45

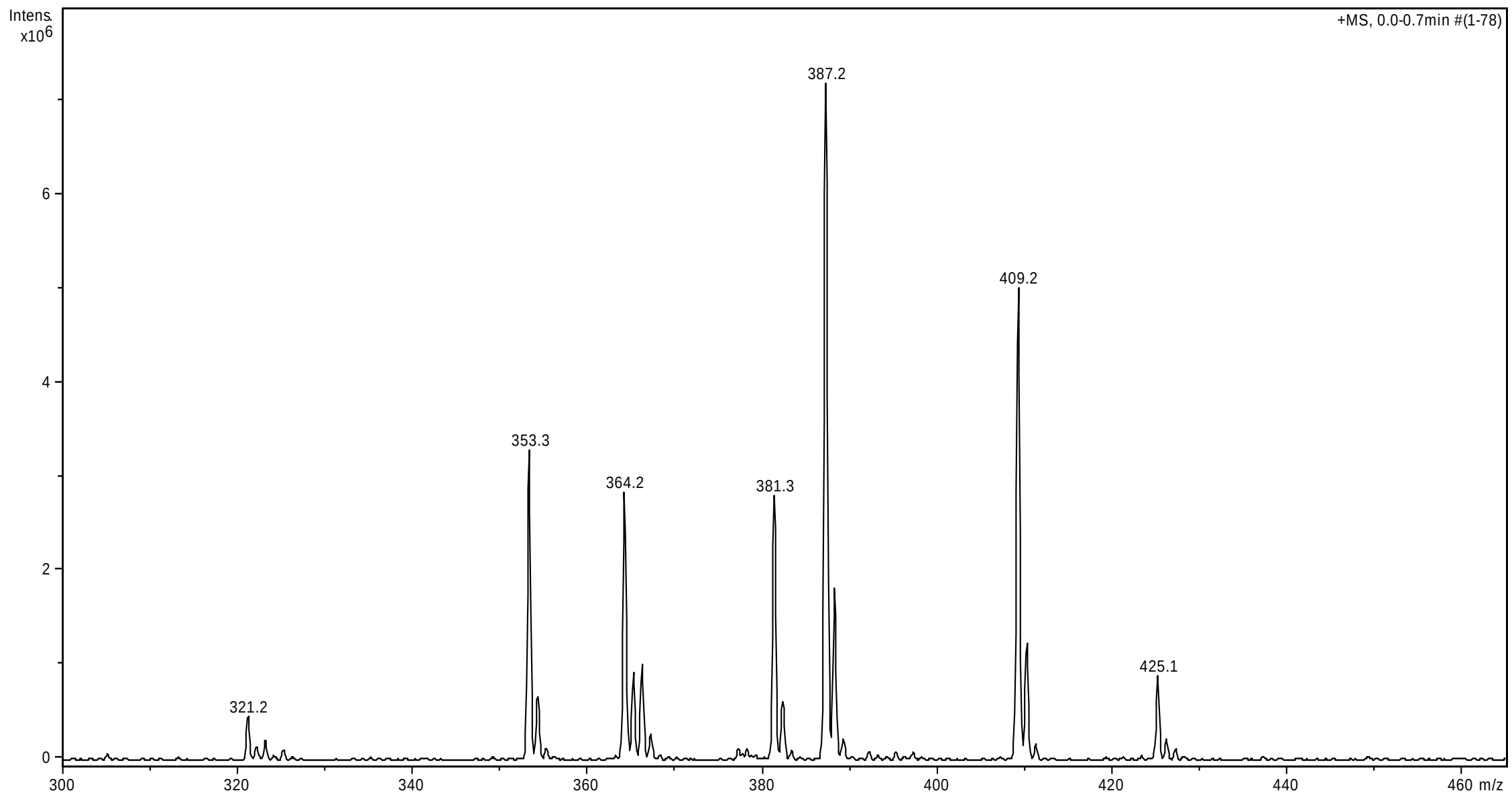

Figure S11. ESI-MS spectrum of **Compound 4**.

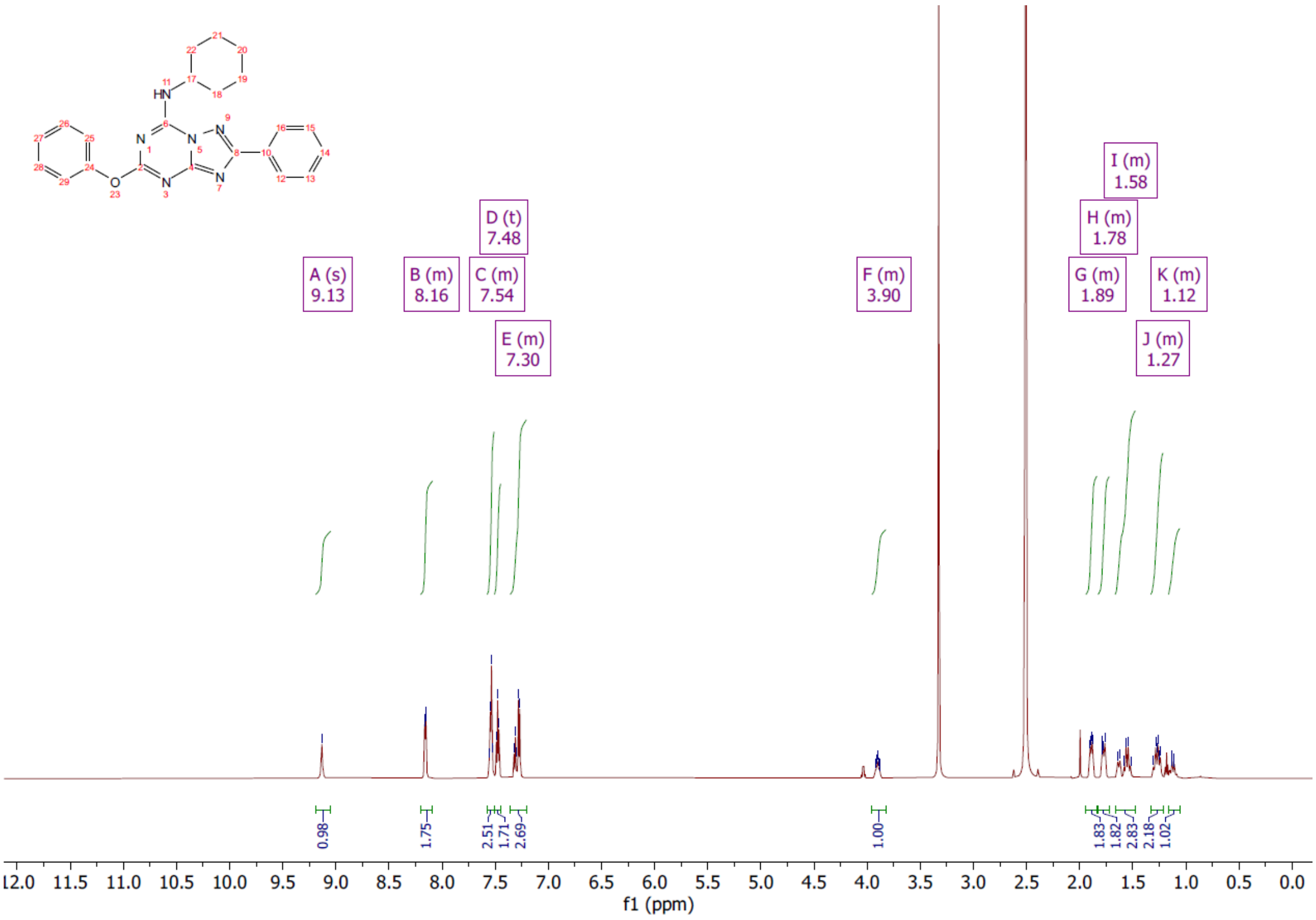

Figure S12. ^{1}H NMR spectrum of **Compound 4** (DMSO-d_6).

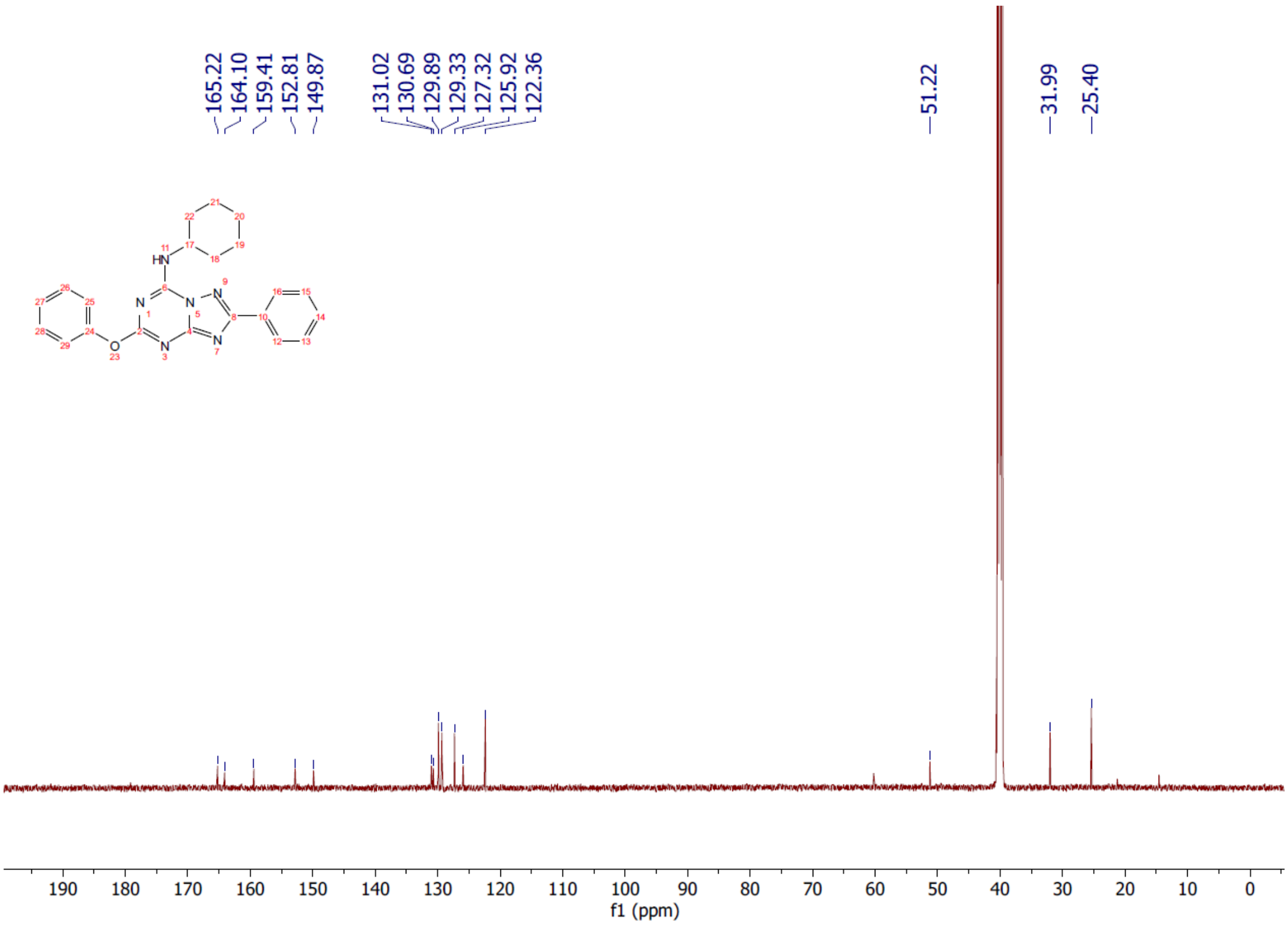

Figure S13. ^{13}C NMR spectrum of **Compound 4** (DMSO-d_6).

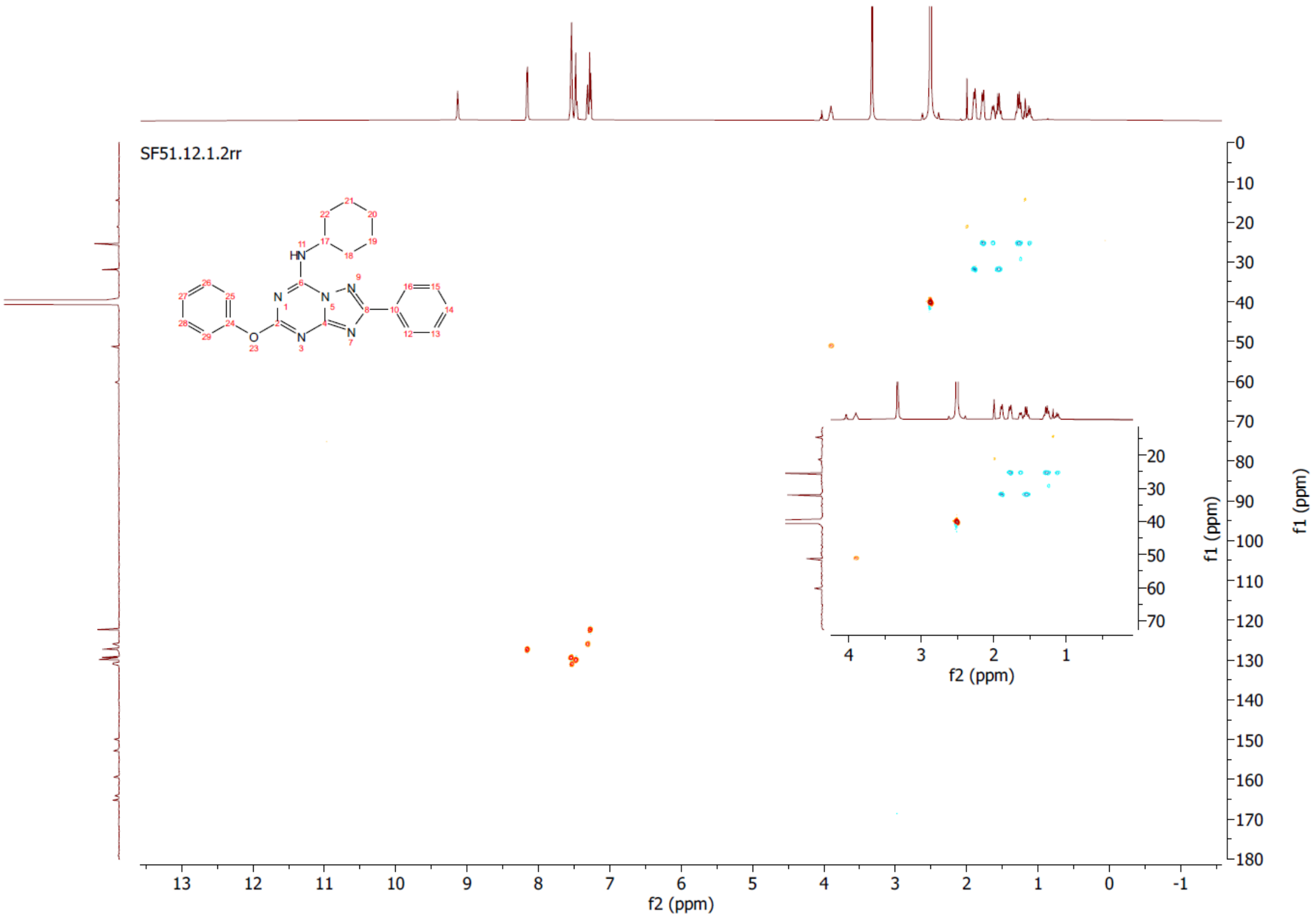

Figure S14. HSQC ^{1}H-^{13}C NMR spectrum of **Compound 4** (DMSO-d_6).

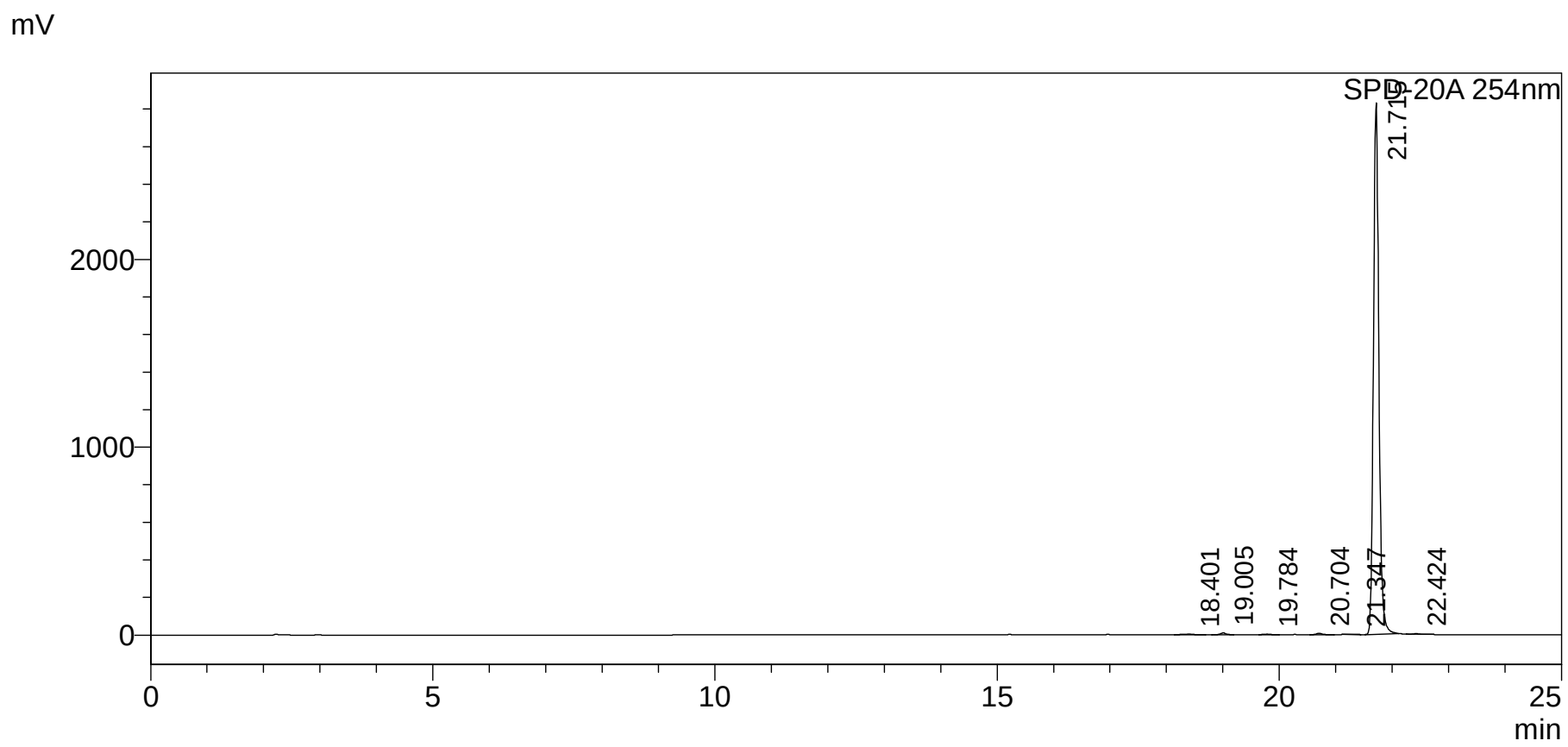

<Peak Table>

SPD-20A 254nm

Peak#	Ret. Time	Area	Area%
1	18.401	30667	0.159
2	19.005	61778	0.321
3	19.784	18652	0.097
4	20.704	54658	0.284
5	21.347	16635	0.086
6	21.715	19028952	98.937
7	22.424	22078	0.115
Total		19233420	100.000

Figure S15. HPLC Chromatogram of **Compound 4**.

N-benzyl-5-phenoxy-2-phenyl-[1,2,4]triazolo[1,5-*a*][1,3,5]triazine-7-amine **5**

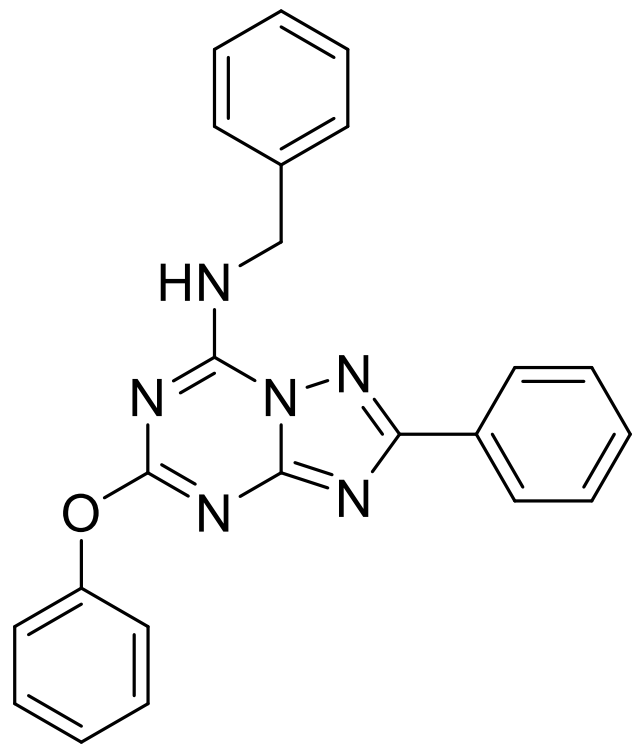

Chemical Formula: $C_{23}H_{18}N_6O$
Molecular Weight: 394.43

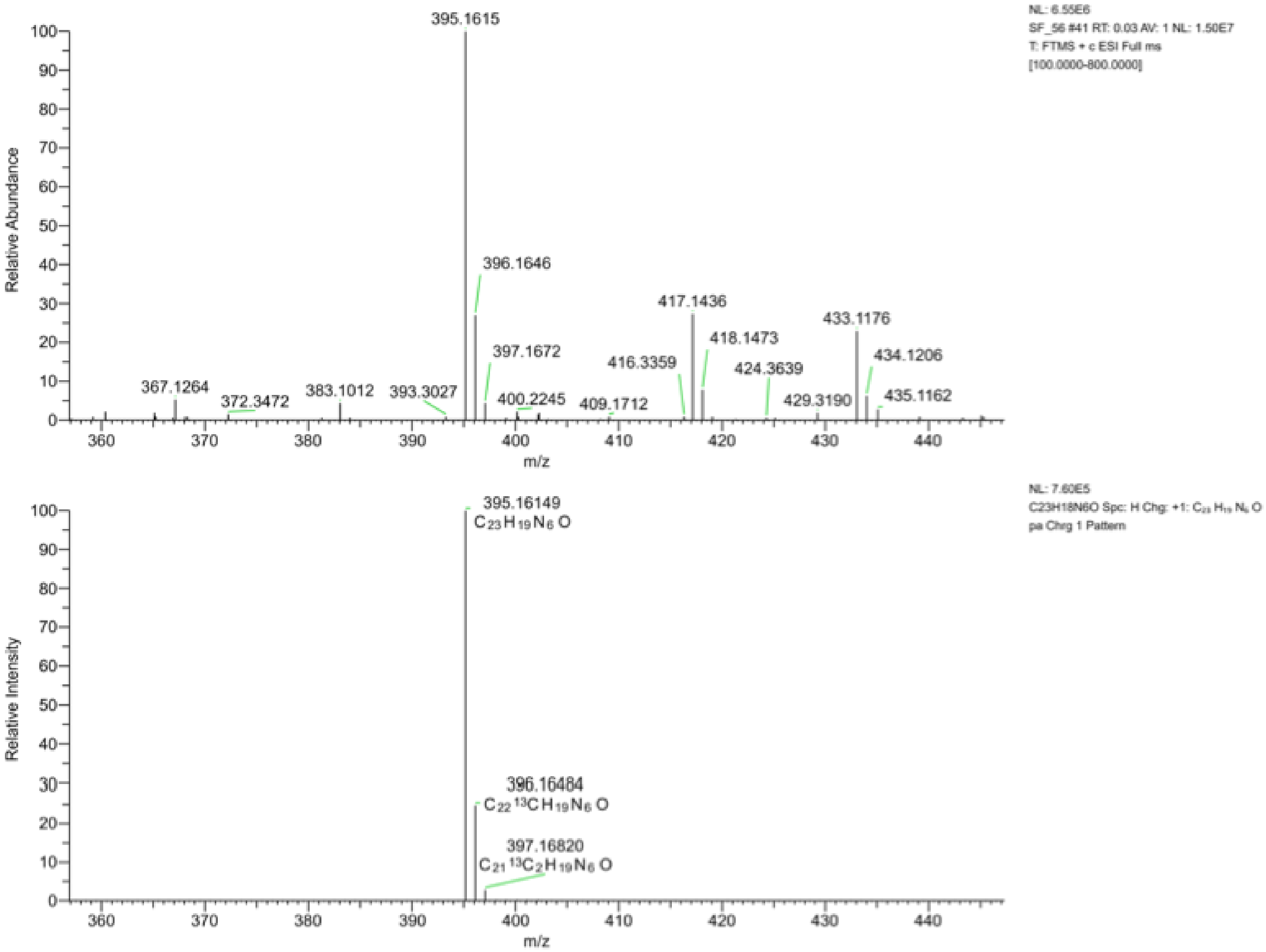

Figure S16. HRMS (ESI) spectrum of **Compound 5**.

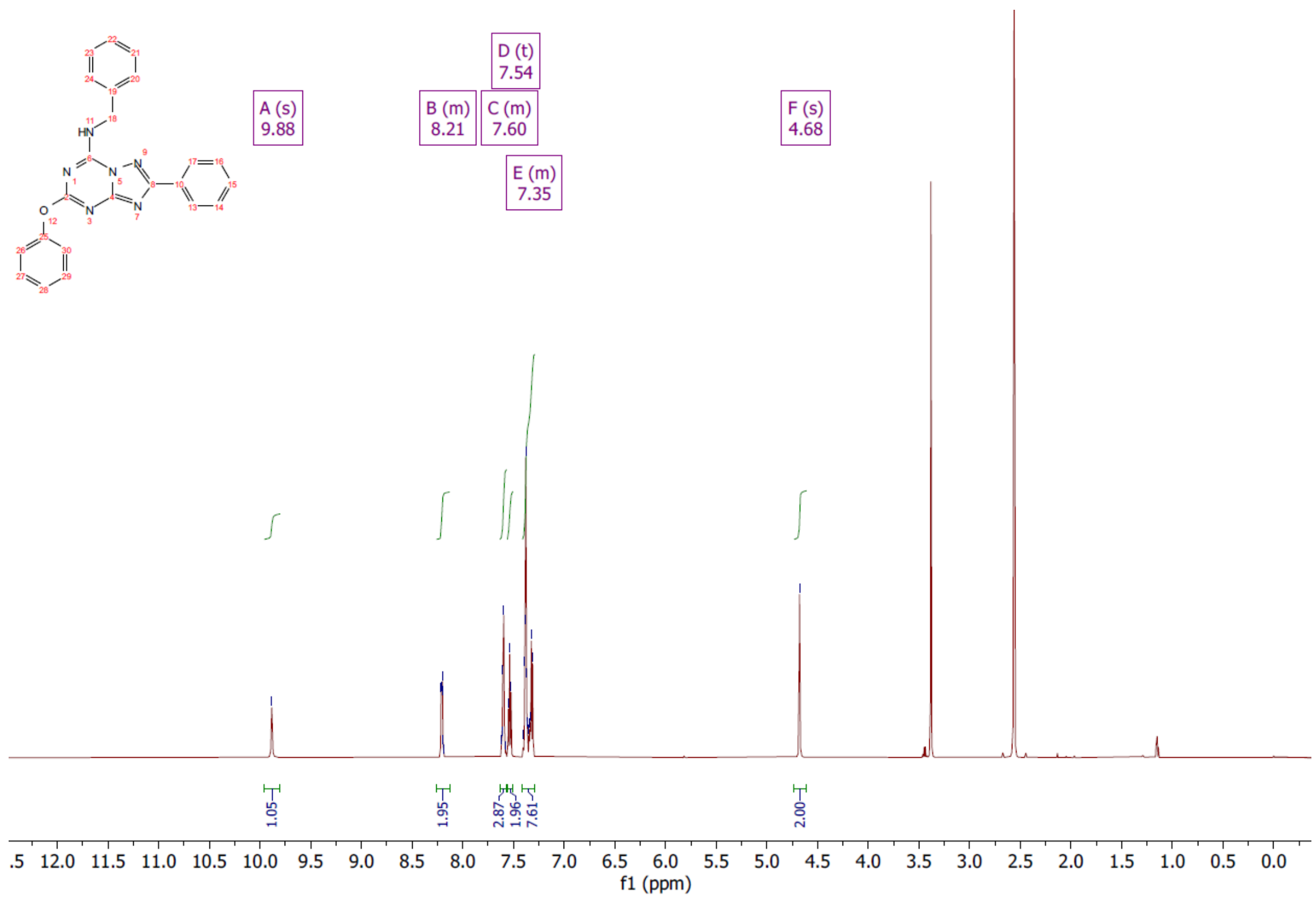

Figure S17. ^{1}H NMR spectrum of **Compound 5** (DMSO-d_6).

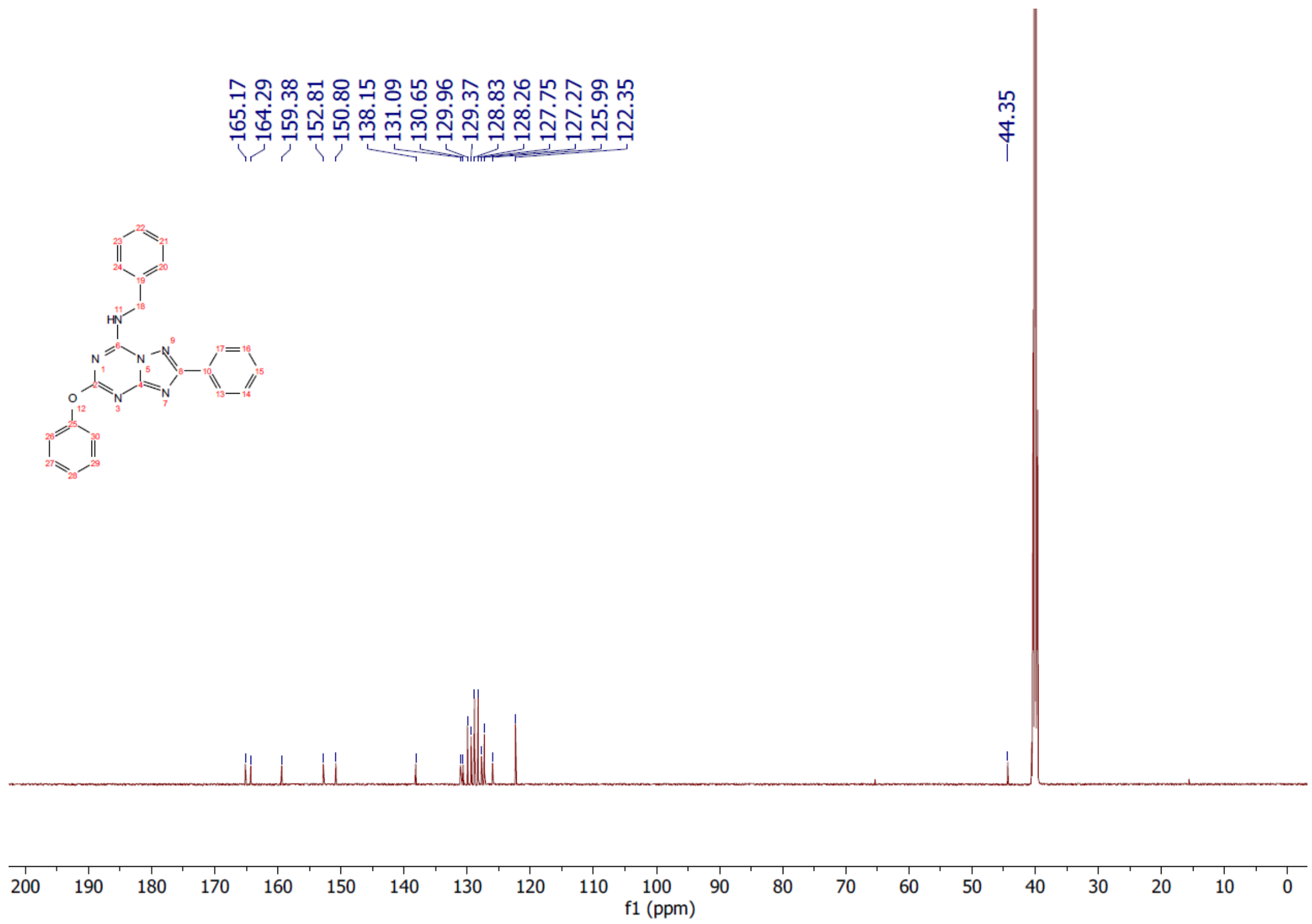

Figure S18. ^{13}C NMR spectrum of **Compound 5** (DMSO-d_6).

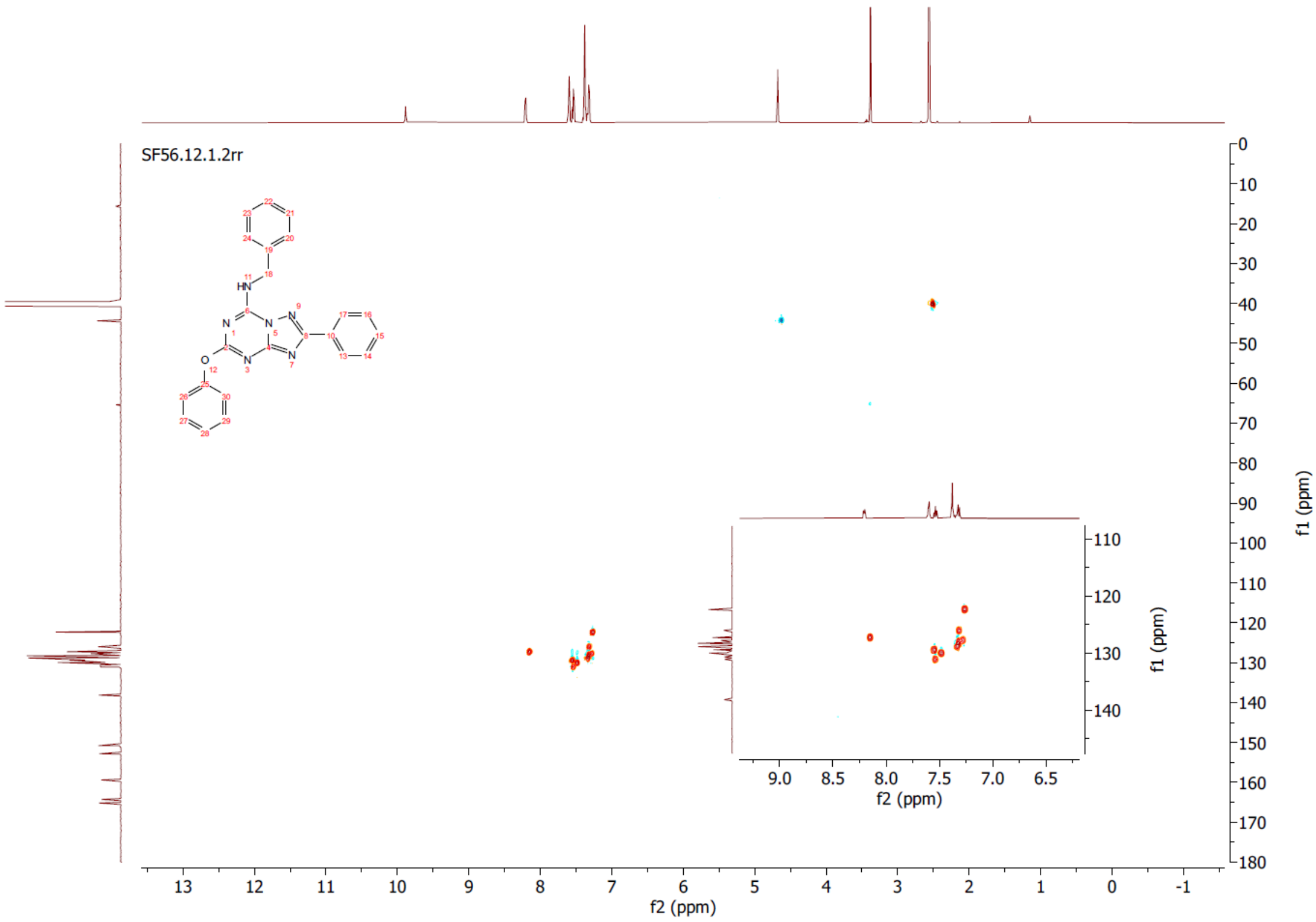

Figure S19. HSQC ^{1}H-^{13}C NMR spectrum of **Compound 5** (DMSO-d_6).

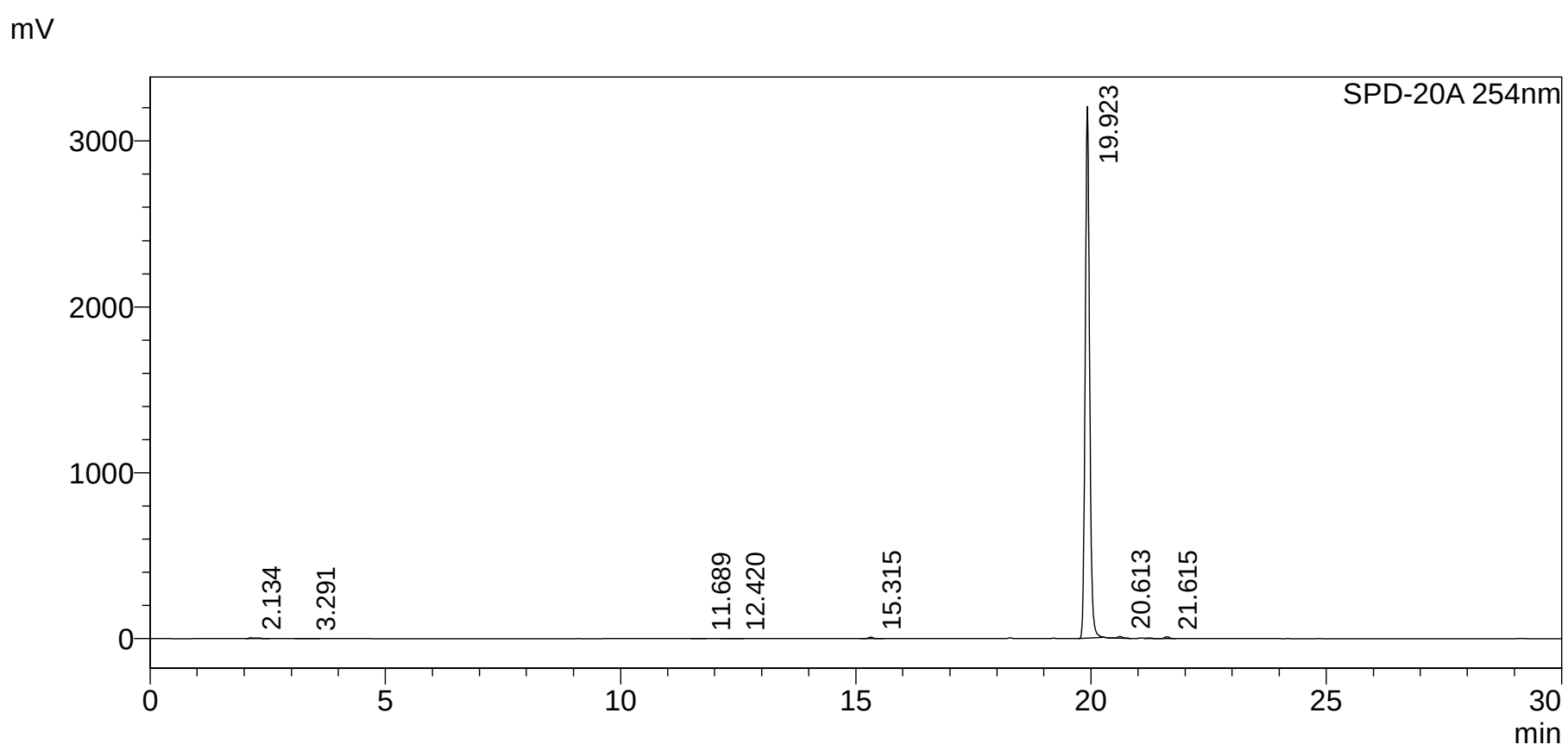

<Peak Table>

SPD-20A 254nm

Peak#	Ret. Time	Area	Area%
1	2.134	48689	0.230
2	3.291	3391	0.016
3	11.689	2435	0.012
4	12.420	3640	0.017
5	15.315	58255	0.275
6	19.923	20907443	98.829
7	20.613	57515	0.272
8	21.615	73848	0.349
Total		21155215	100.000

Figure S20. HPLC Chromatogram of **Compound 5.**

N-benzyl-2-(4-methoxyphenyl)-5-phenoxy-[1,2,4]triazolo[1,5-*a*][1,3,5]triazine-7-amine **7**

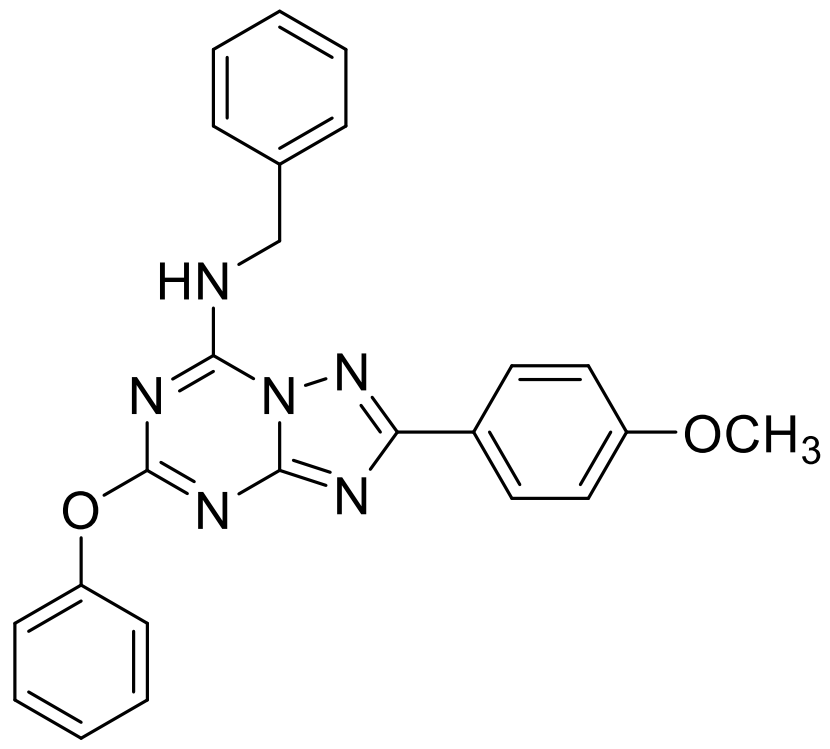

Chemical Formula: $C_{24}H_{20}N_6O_2$
Molecular Weight: 424.45

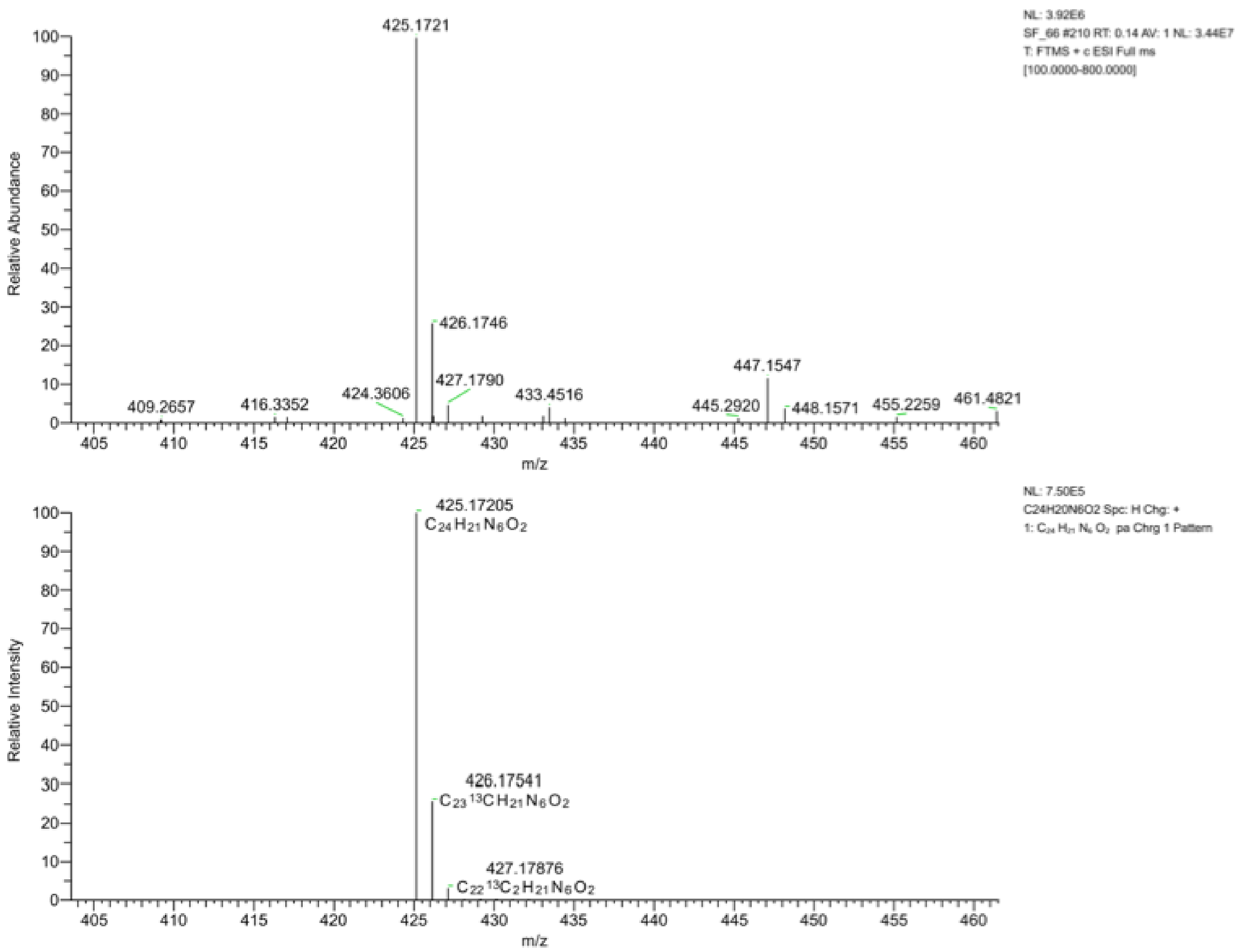

Figure S21. HRMS (ESI) spectrum of **Compound 7**.

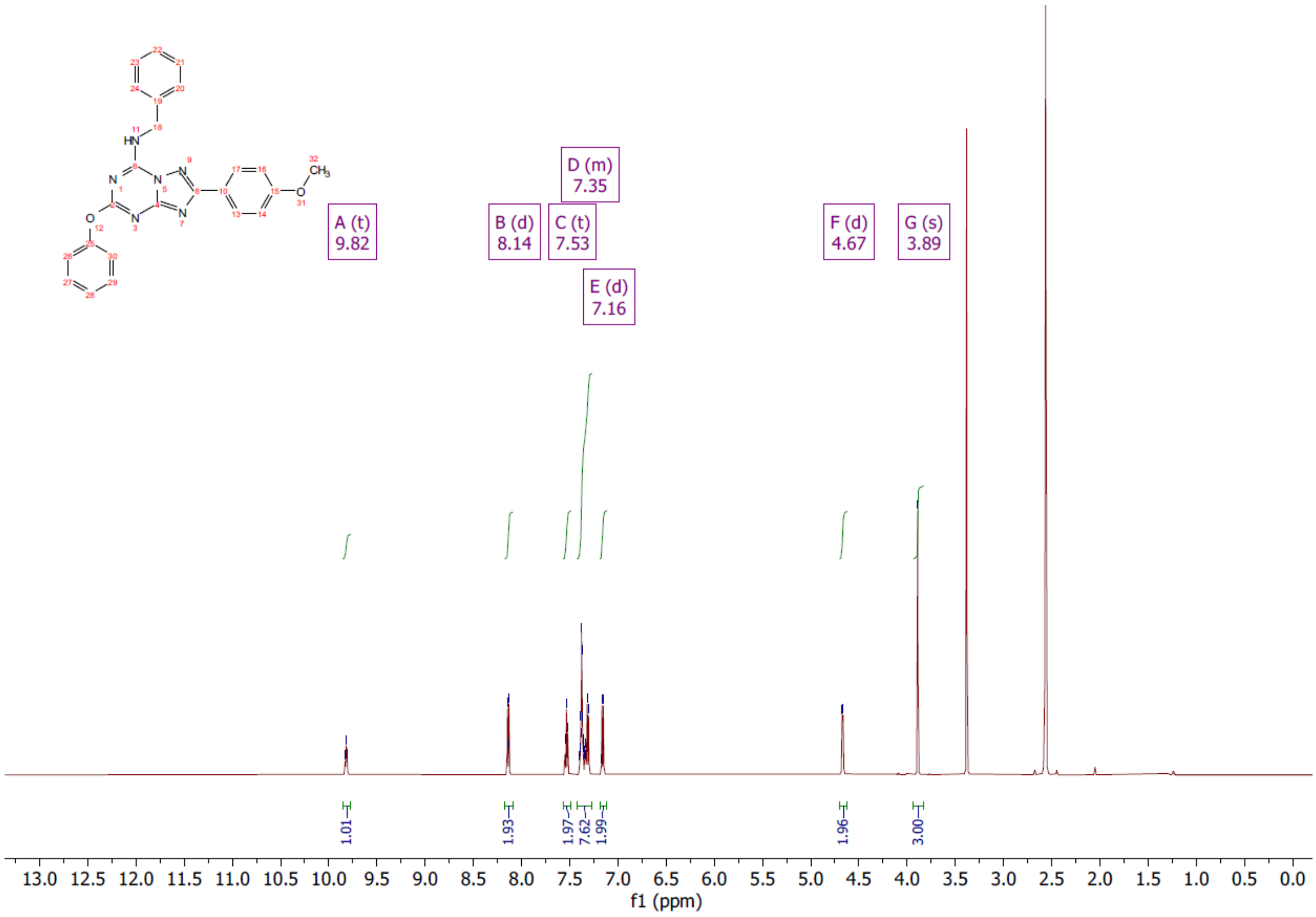

Figure S22. ^{1}H NMR spectrum of **Compound 7** (DMSO-d_6).

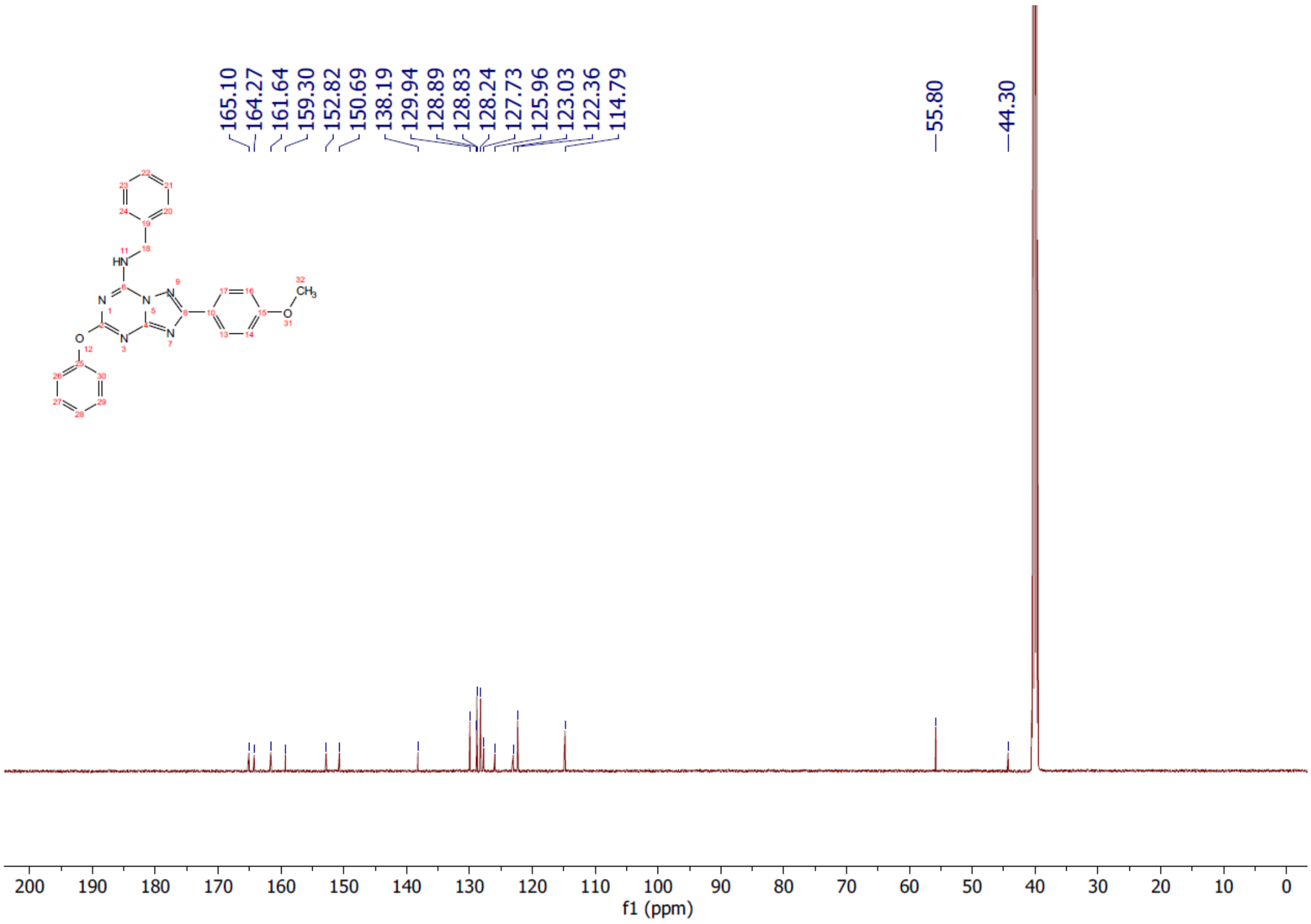

Figure S23. ^{13}C NMR spectrum of **Compound 7** (DMSO-d_6).

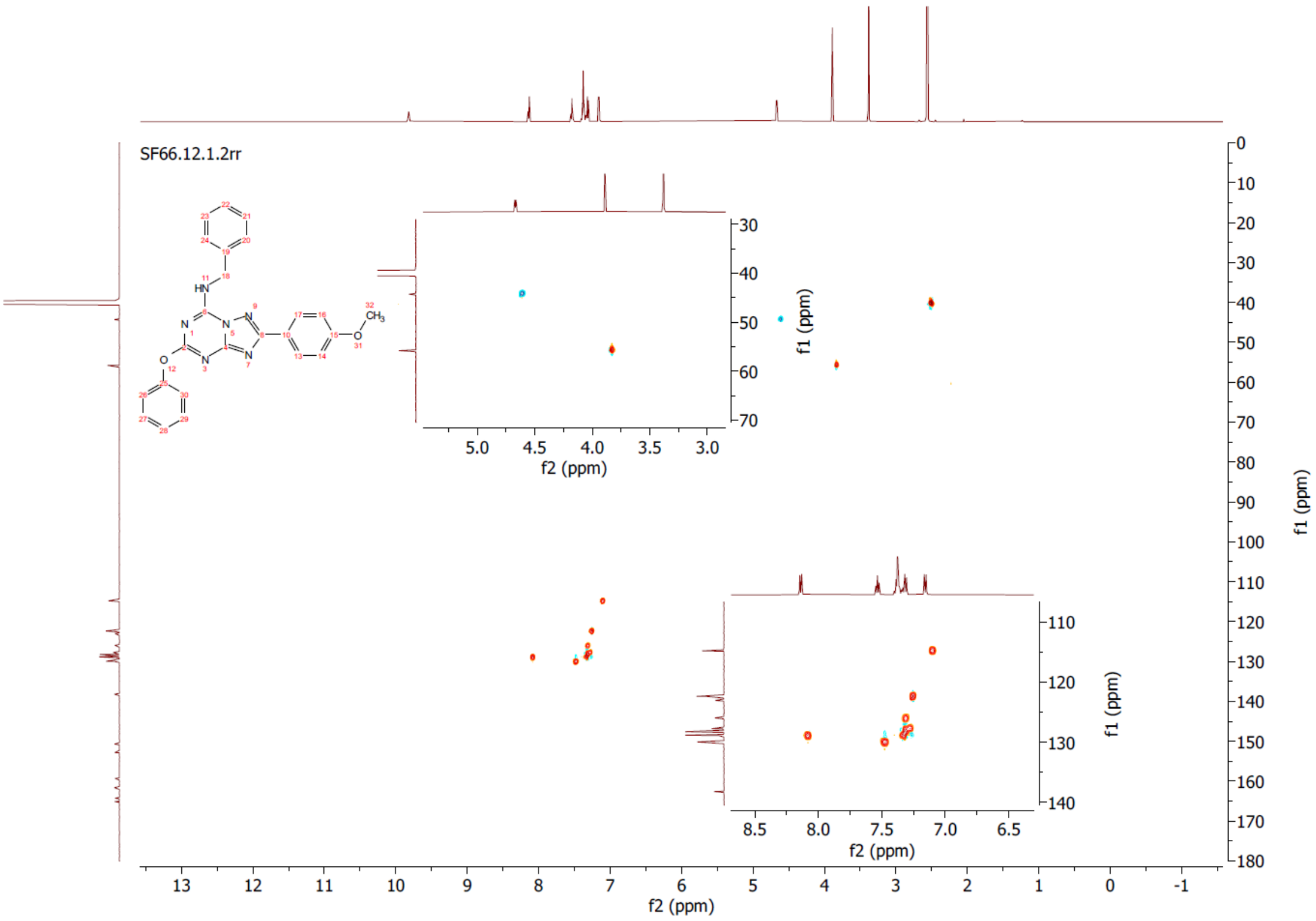

Figure S24. HSQC ^{1}H-^{13}C NMR spectrum of **Compound 7** (DMSO-d_6).

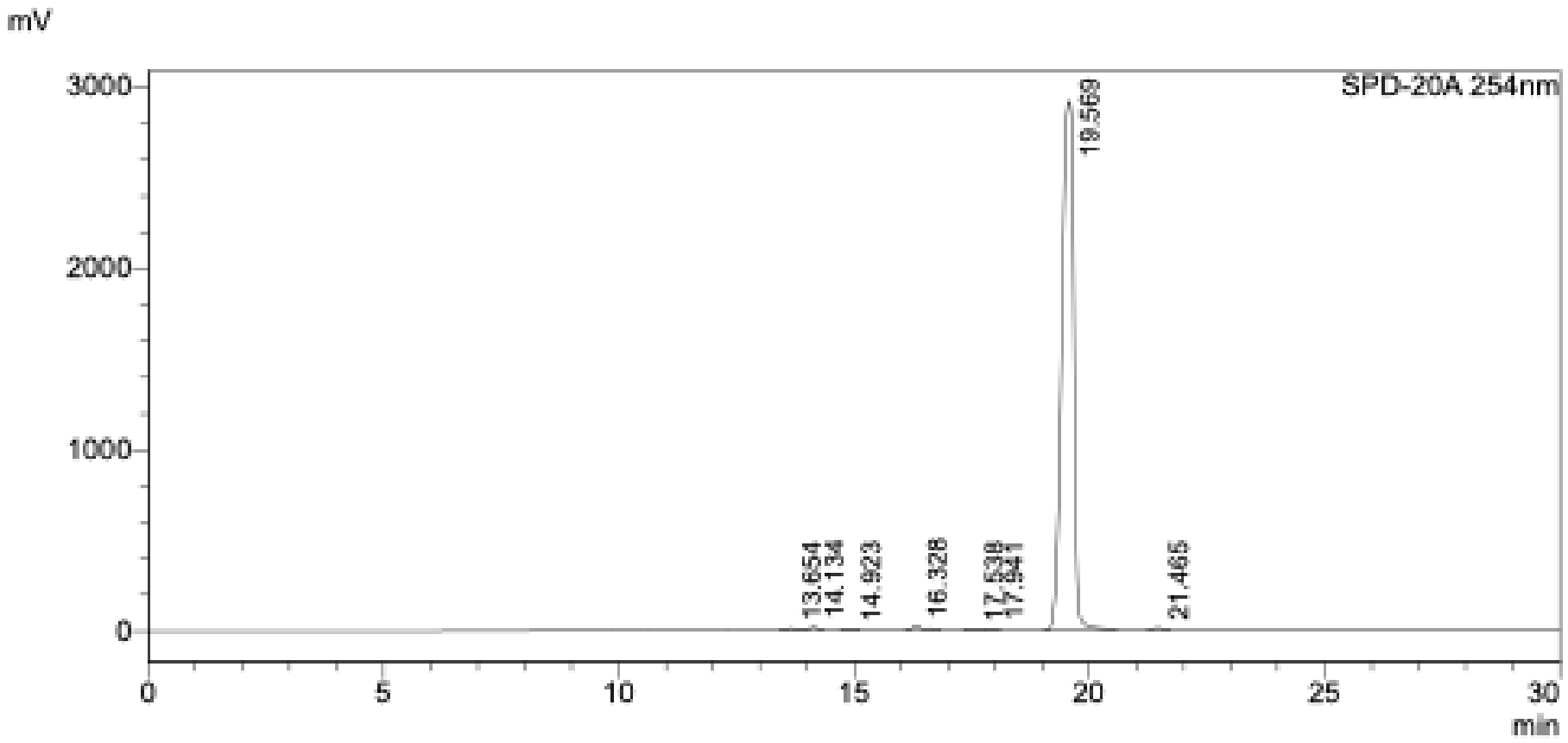

<Peak Table>

SPD-20A 254nm

Peak#	Ret. Time	Area	Height	Area%
1	13.654	93064	9280	0.178
2	14.134	143547	14652	0.274
3	14.923	39286	4098	0.075
4	16.328	286073	21130	0.547
5	17.538	46286	3738	0.088
6	17.941	40175	4270	0.077
7	19.569	51603125	2913030	98.633
8	21.465	66682	6303	0.127
Total		52318238	2976499	100.000

Figure S25. HPLC Chromatogram of **Compound 7.**

N5-methyl-2-phenyl-[1,2,4]triazolo[1,5-*a*][1,3,5]triazine-5,7-diamine **8**

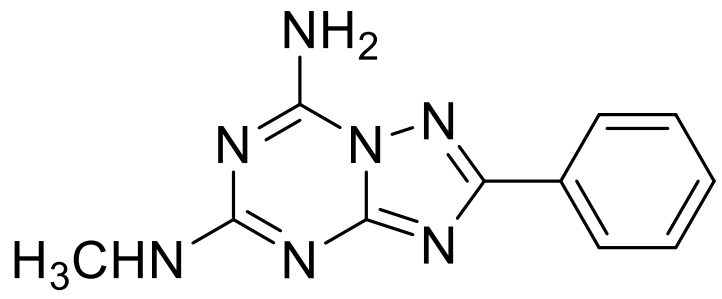

Chemical Formula: $C_{11}H_{11}N_7$
Molecular Weight: 241,25

Chemical Formula: $C_{11}H_{11}N_7$

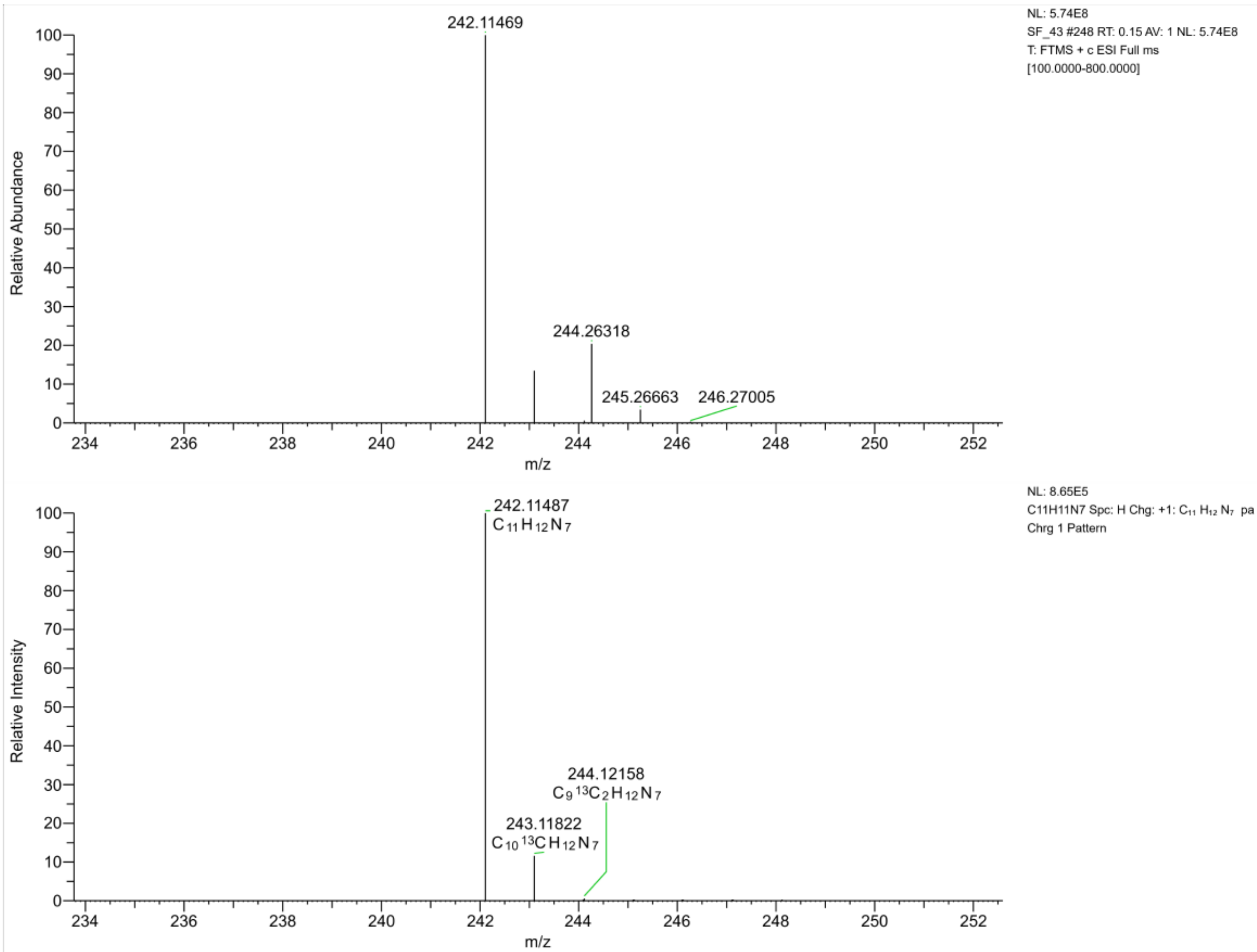

Figure S26. HRMS (ESI) spectrum of **Compound 8**.

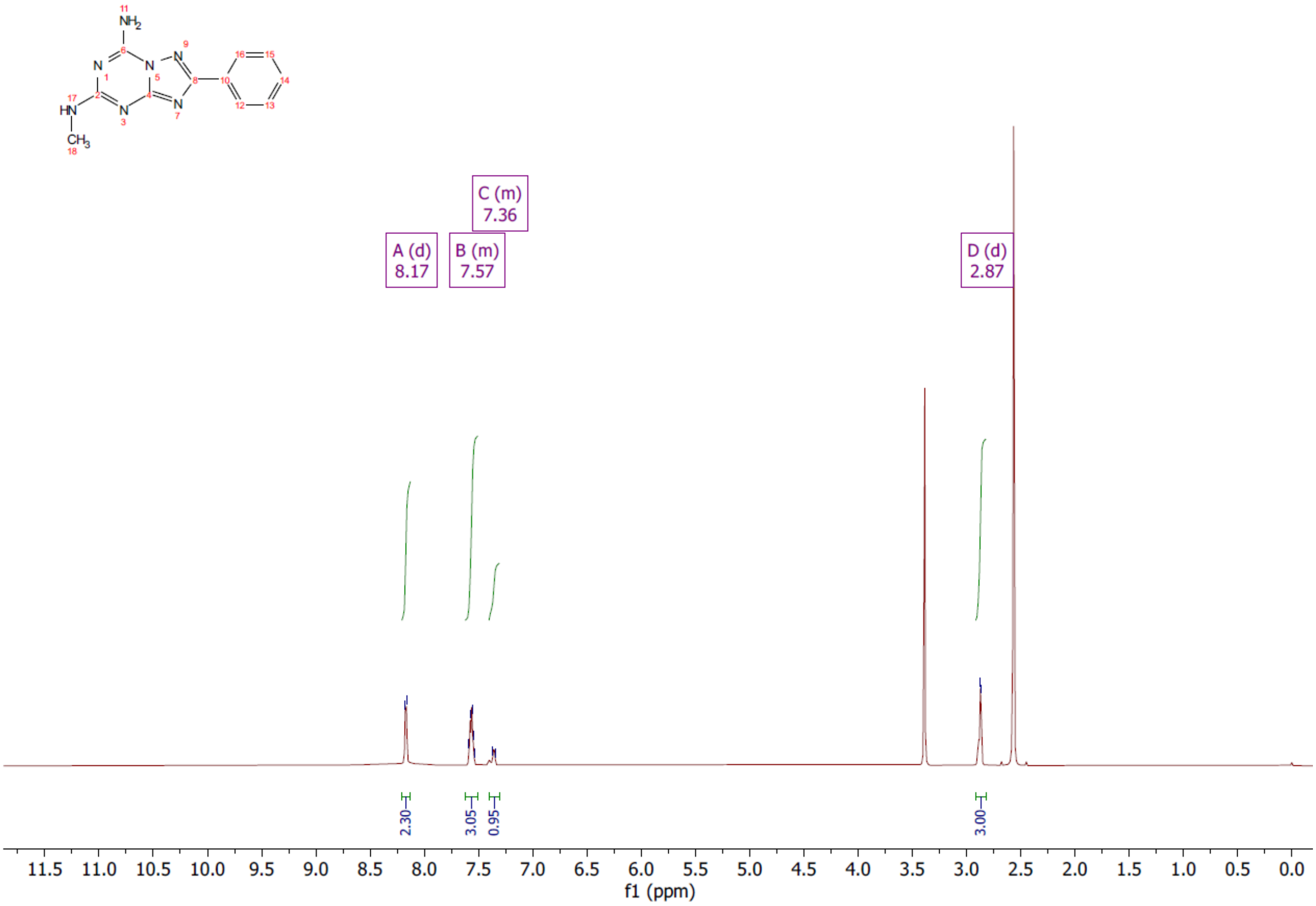

Figure S27. ^{1}H NMR spectrum of **Compound 8** (DMSO-d_6).

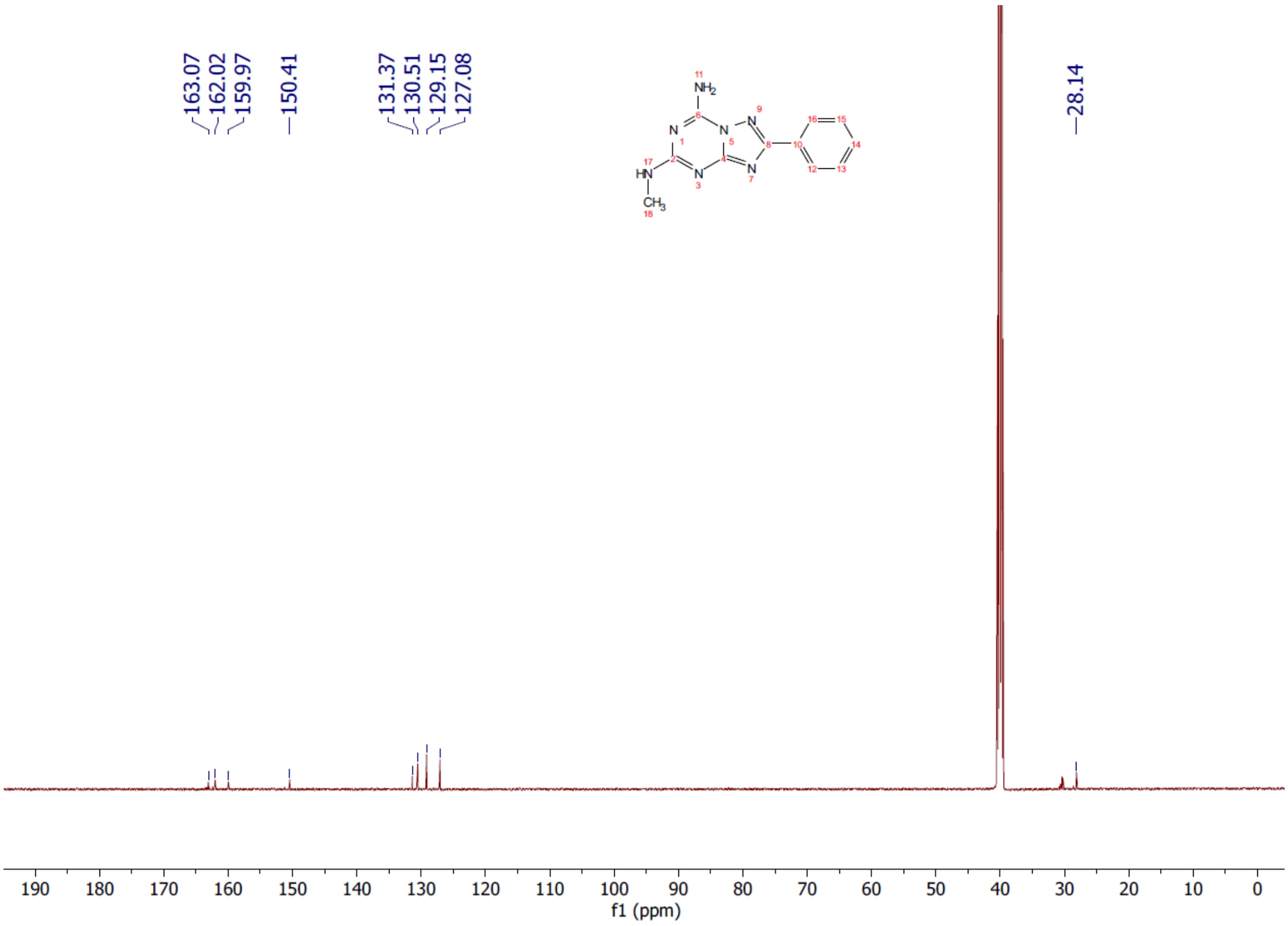

Figure S28. ^{13}C NMR spectrum of **Compound 8** (DMSO-d_6).

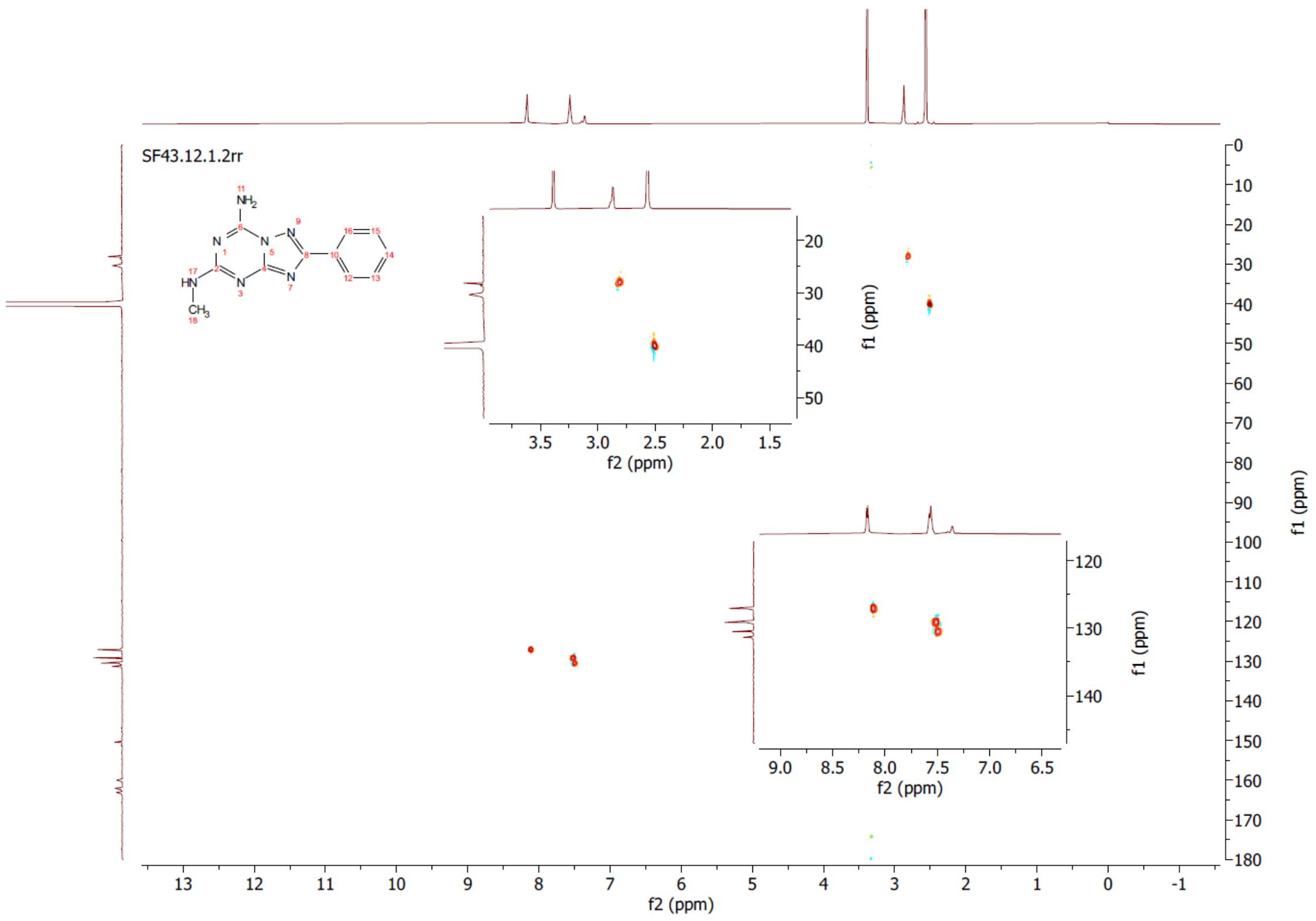

Figure S29. HSQC ^1H-^{13}C NMR spectrum of **Compound 8** (DMSO-d_6).

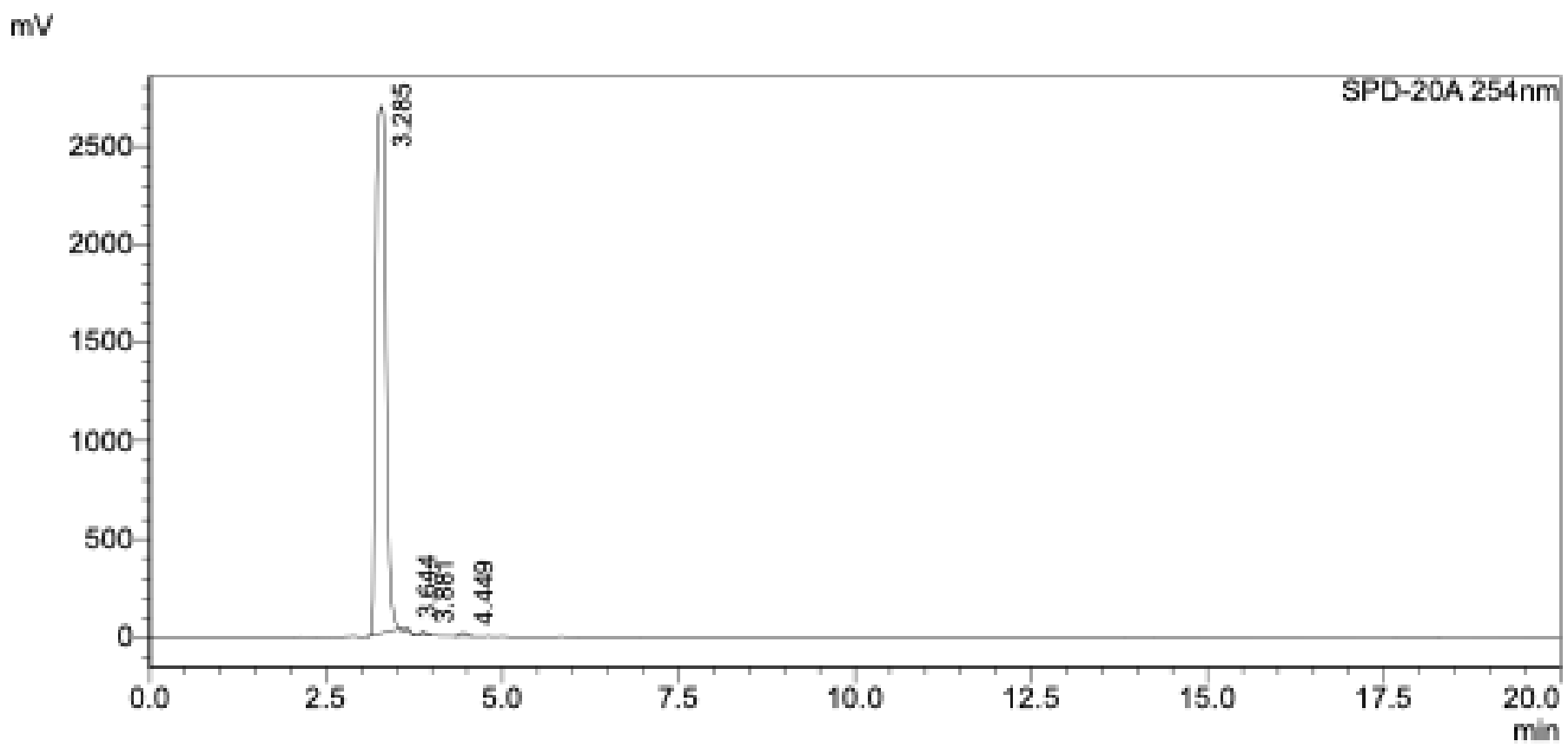

<Peak Table>

SPD-20A 254nm

Peak#	Ret. Time	Area	Height	Area%
1	3.285	26667012	2682259	98.902
2	3.644	105132	22660	0.390
3	3.881	90990	19726	0.337
4	4.449	99825	18265	0.370
Total		26962958	2742910	100.000

Figure S30. HPLC chromatogram of **Compound 8**.

N[5]-cyclopropyl-2-phenyl-[1,2,4]triazolo[1,5-*a*][1,3,5]triazine-5,7-diamine **9**

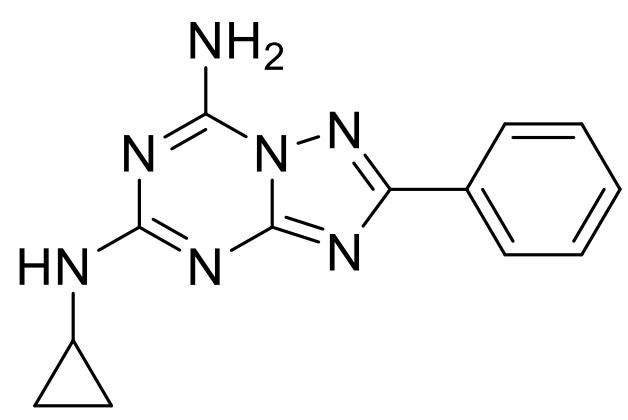

Chemical Formula: $C_{13}H_{13}N_7$
Molecular Weight: 267,29

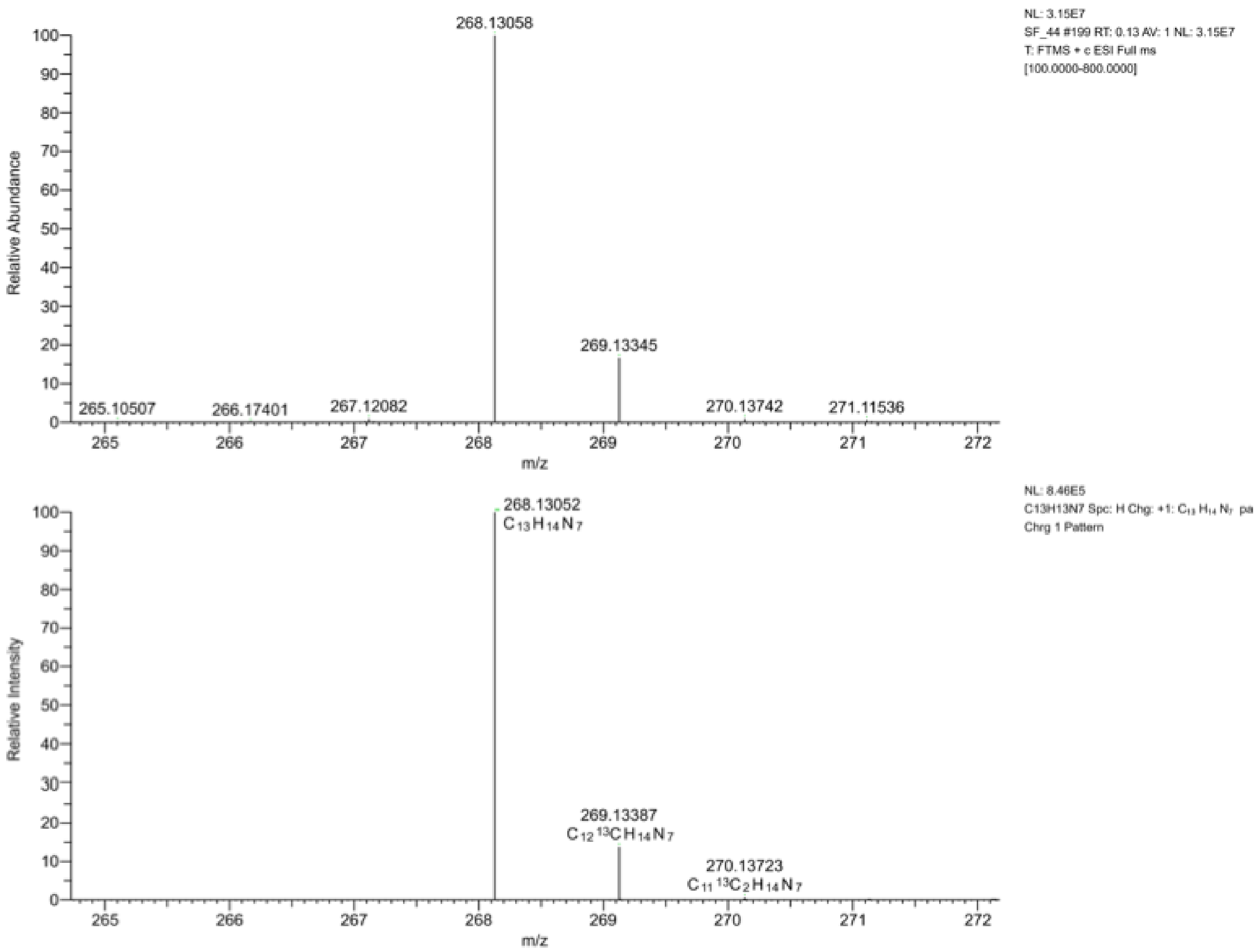

Figure S31. HRMS (ESI) spectrum of **Compound 9**.

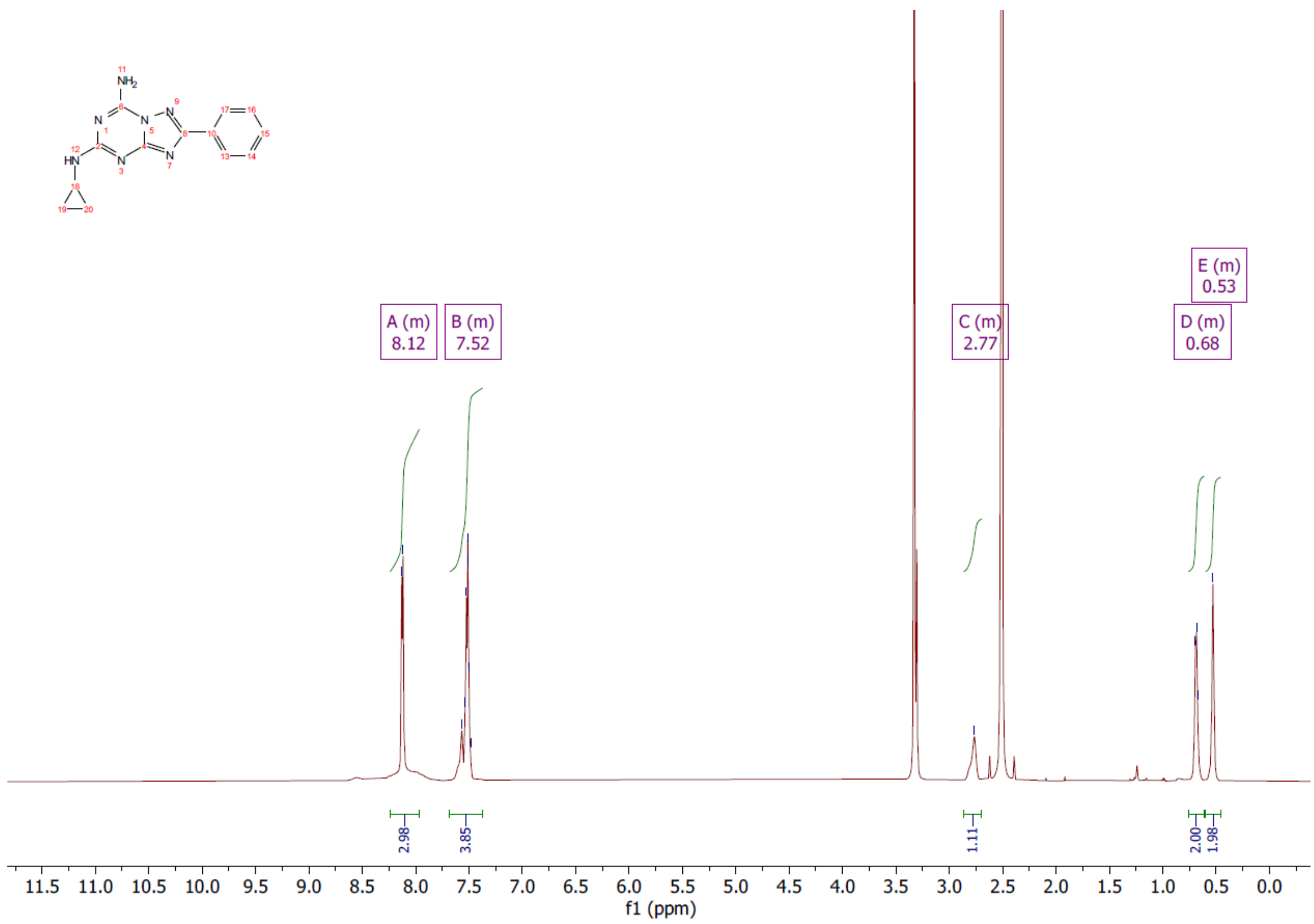

Figure S32. ^{1}H NMR spectrum of **Compound 9** (DMSO-d_6).

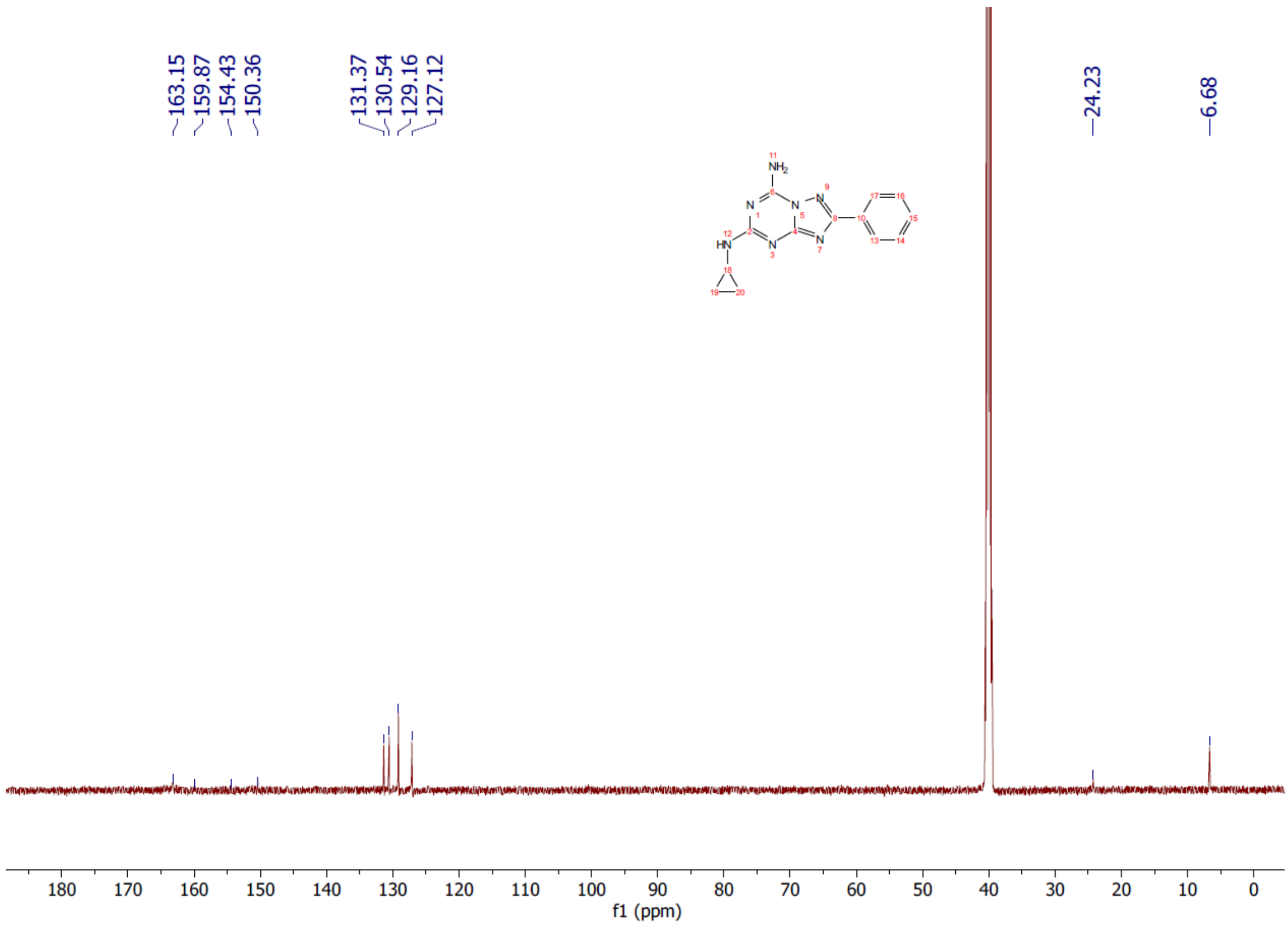

Figure S33. ^{13}C NMR spectrum of **Compound 9** (DMSO-d_6).

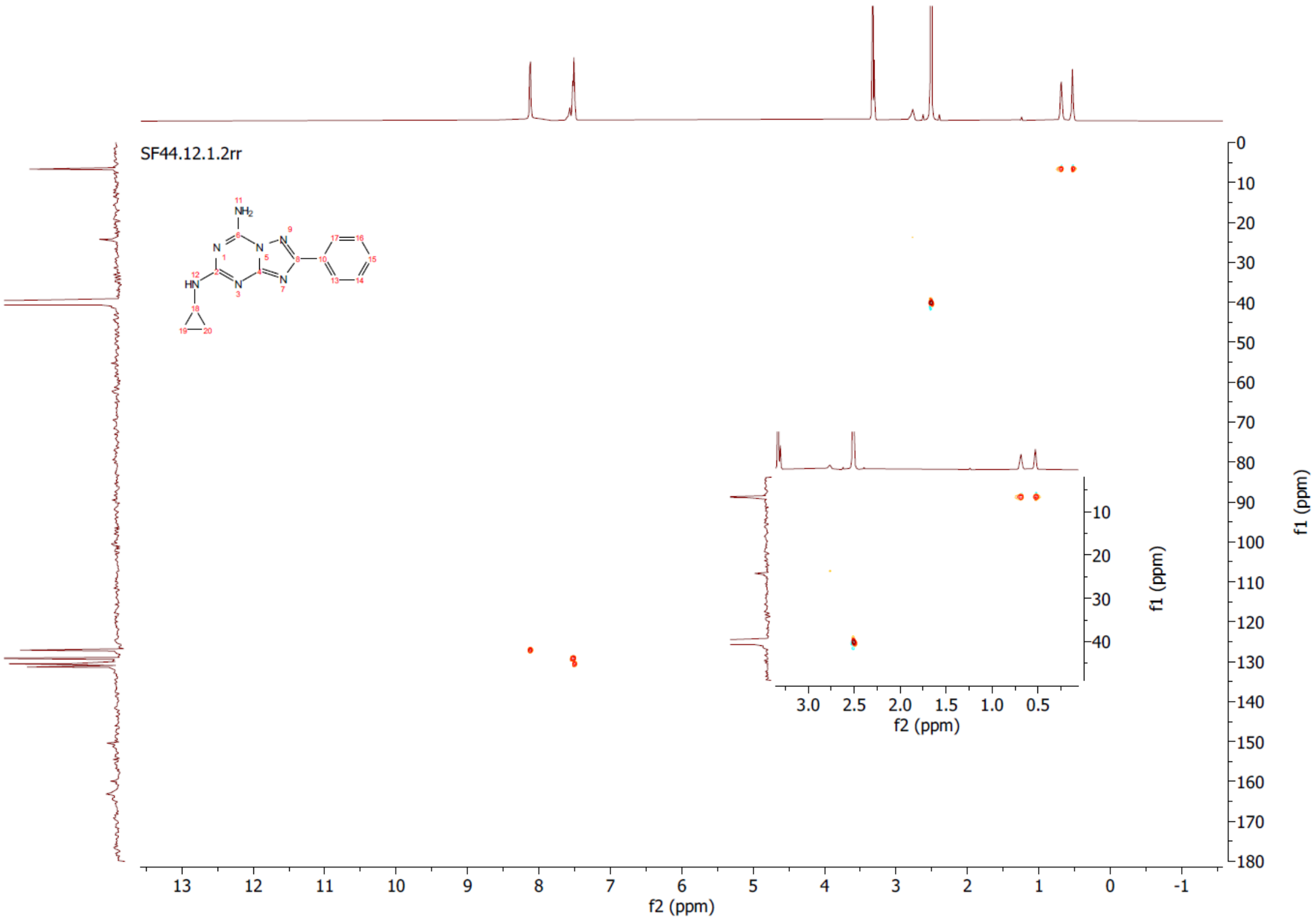

Figure S34. HSQC ^1H-^{13}C NMR spectrum of **Compound 9** (DMSO-d_6).

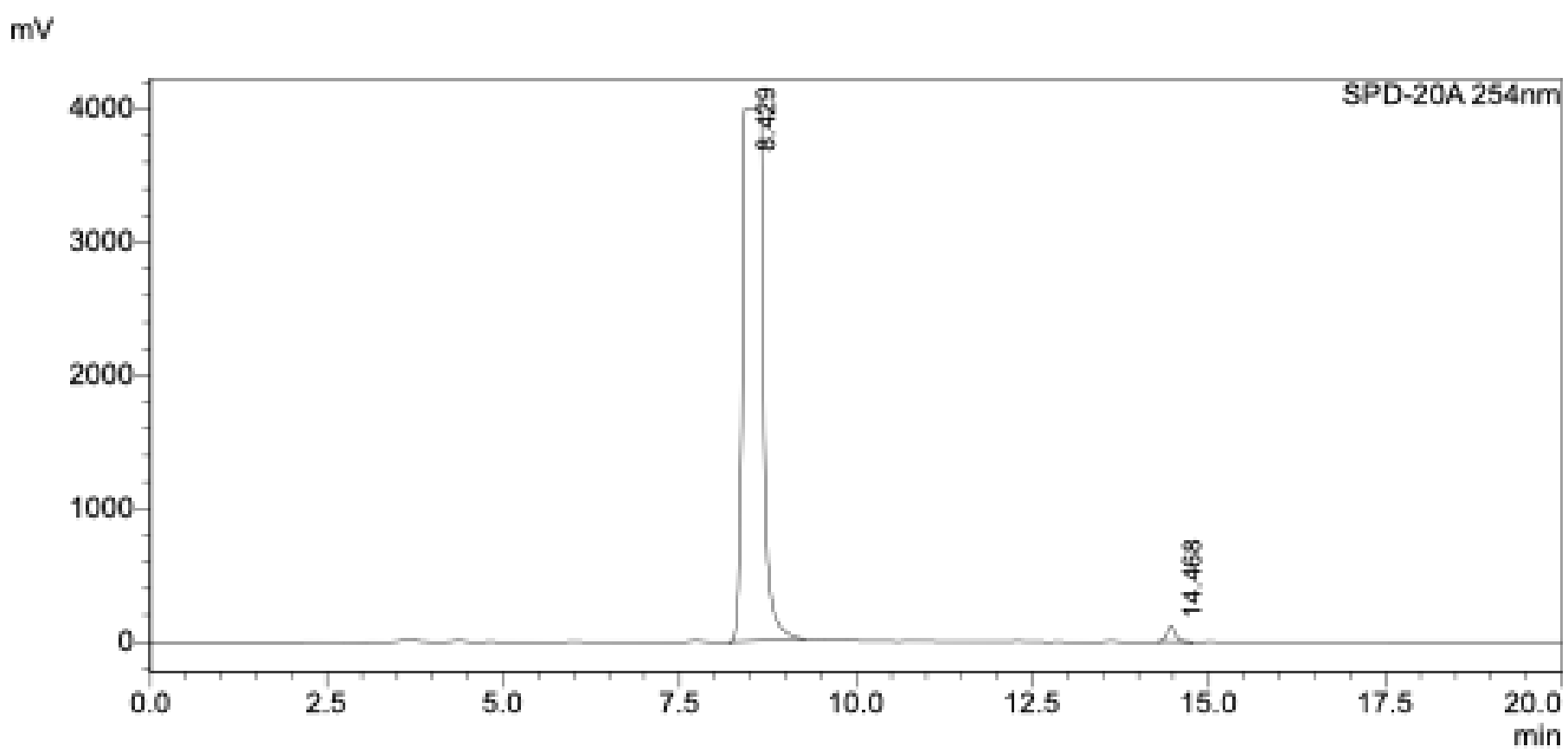

<Peak Table>

SPD-20A 254nm

Peak#	Ret. Time	Area	Height	Area%
1	8.429	79256414	3993809	98.851
2	14.468	921332	115276	1.149
Total		80177747	4109085	100.000

Figure S35. HPLC chromatogram of **Compound 9**.

N[5],N[7]-dimethyl-2-phenyl-[1,2,4]triazolo[1,5-*a*][1,3,5]triazine-5,7-diamine **12**

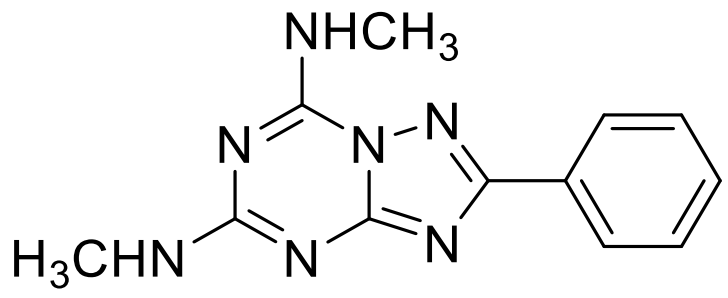

Chemical Formula: $C_{12}H_{13}N_7$
Molecular Weight: 255,28

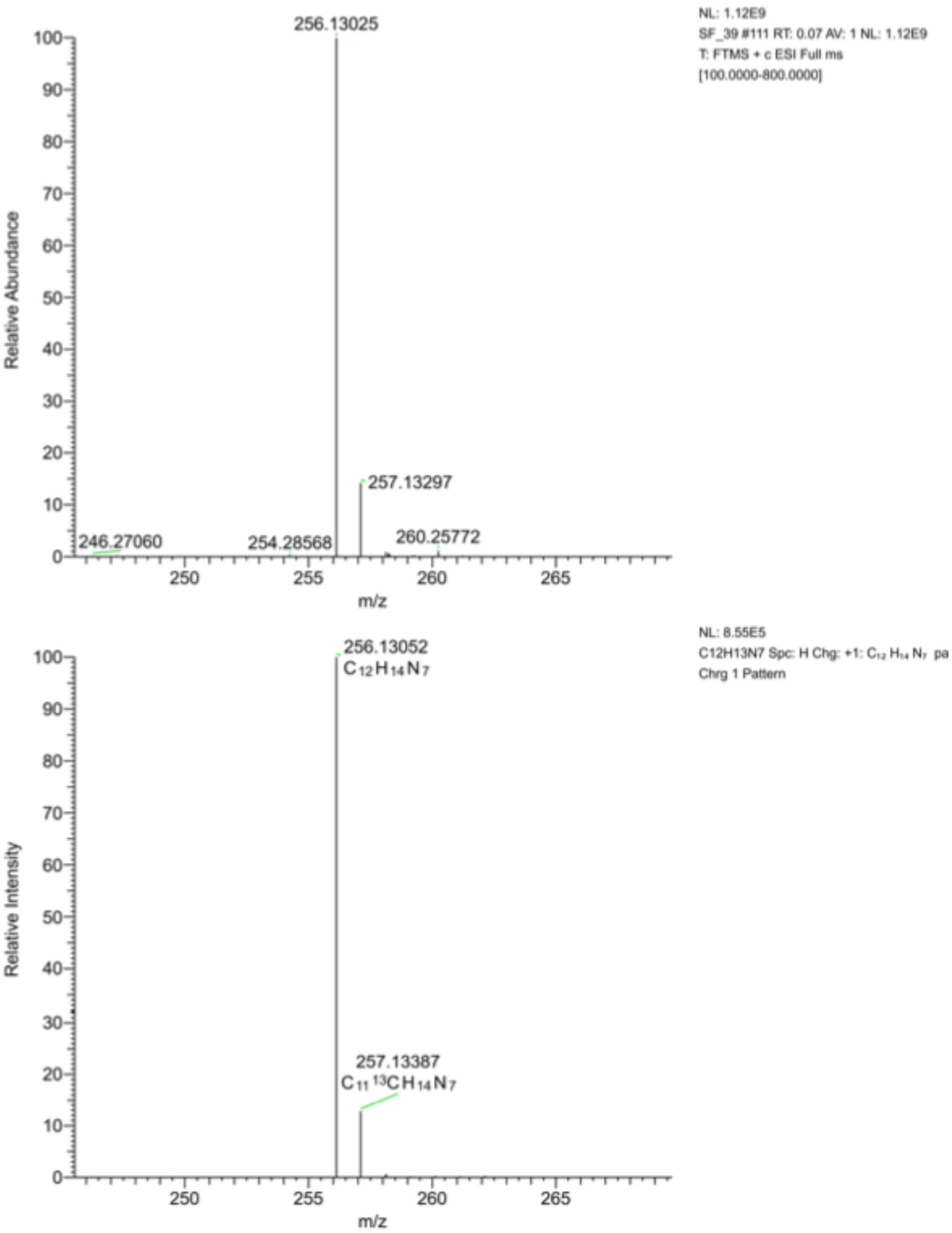

Figure S36. HRMS (ESI) spectrum of **Compound 12**.

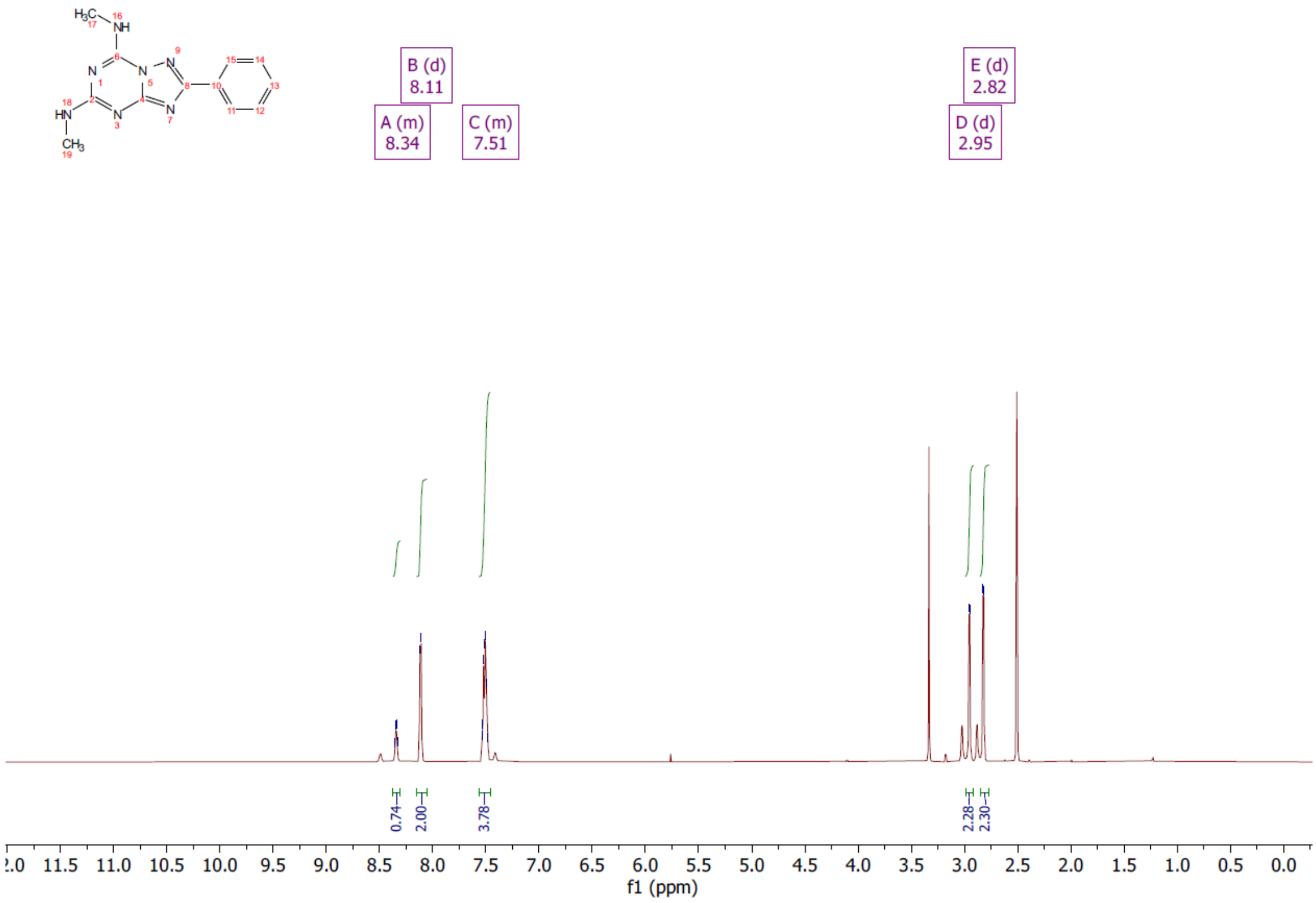

Figure S37. ^{1}H NMR spectrum of **Compound 12** (DMSO-d_6).

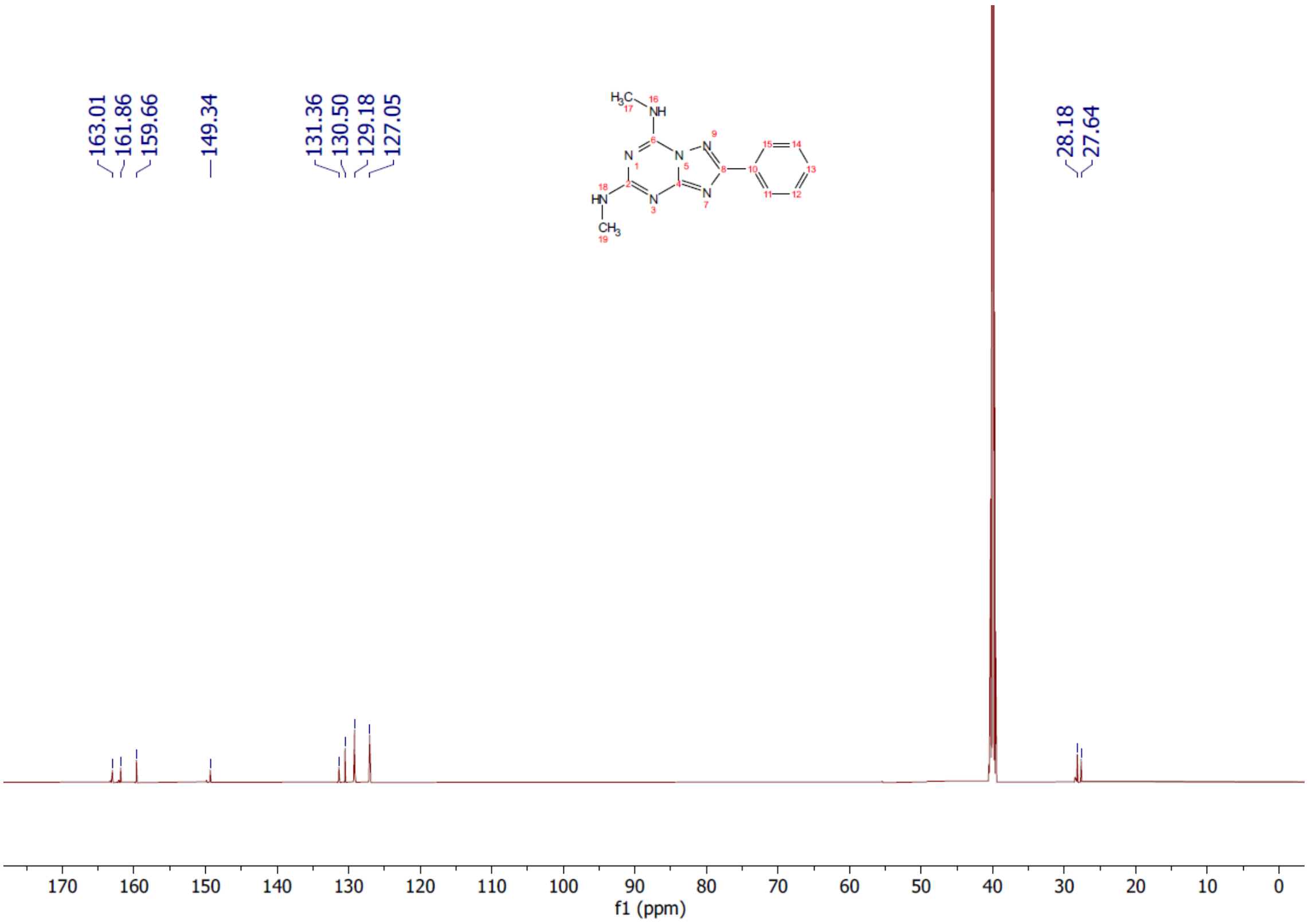

Figure S38. ^{13}C NMR spectrum of **Compound 12** (DMSO-d_6).

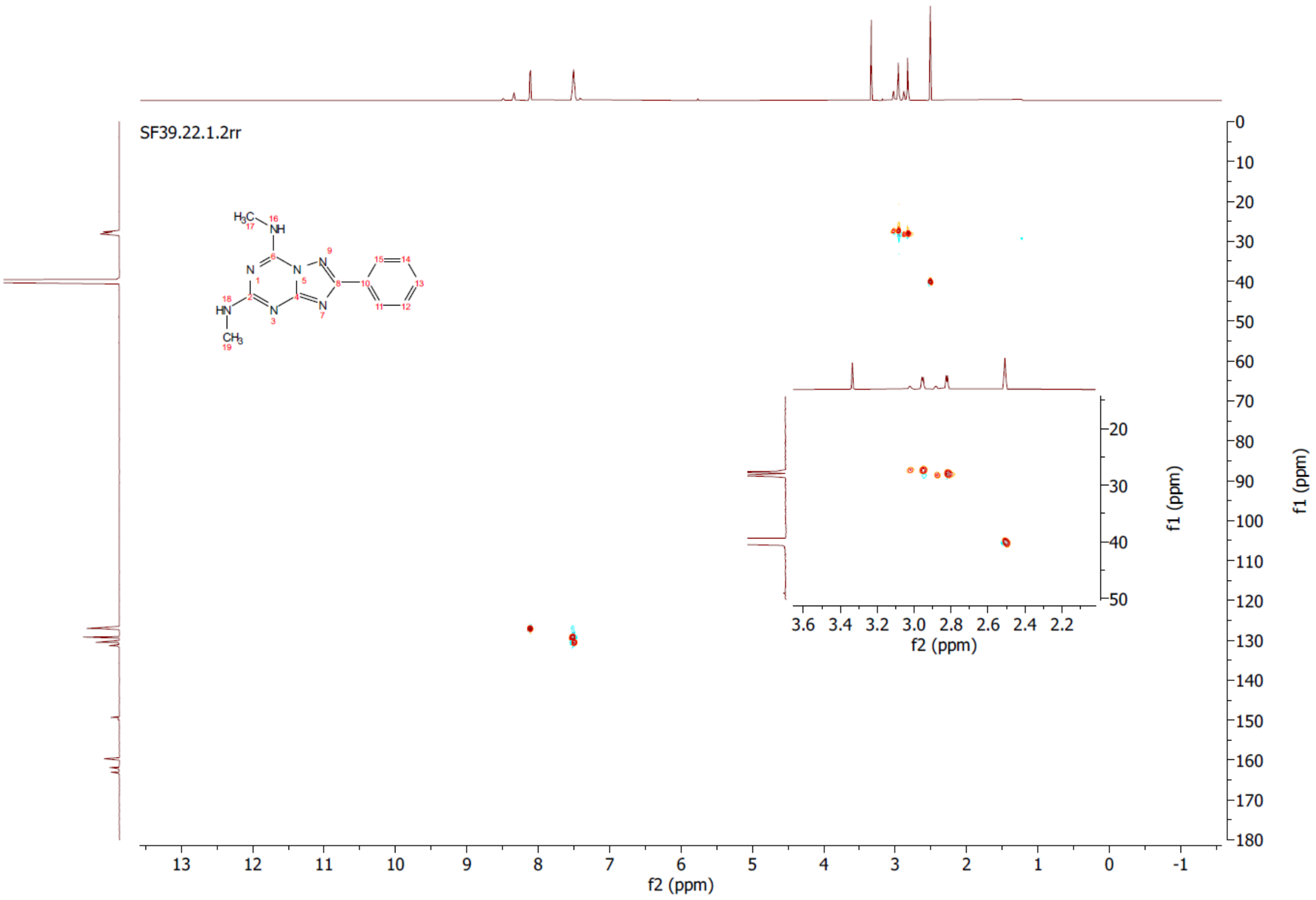

Figure S39. HSQC ^{1}H-^{13}C NMR spectrum of **Compound 12 (**DMSO-d_6).

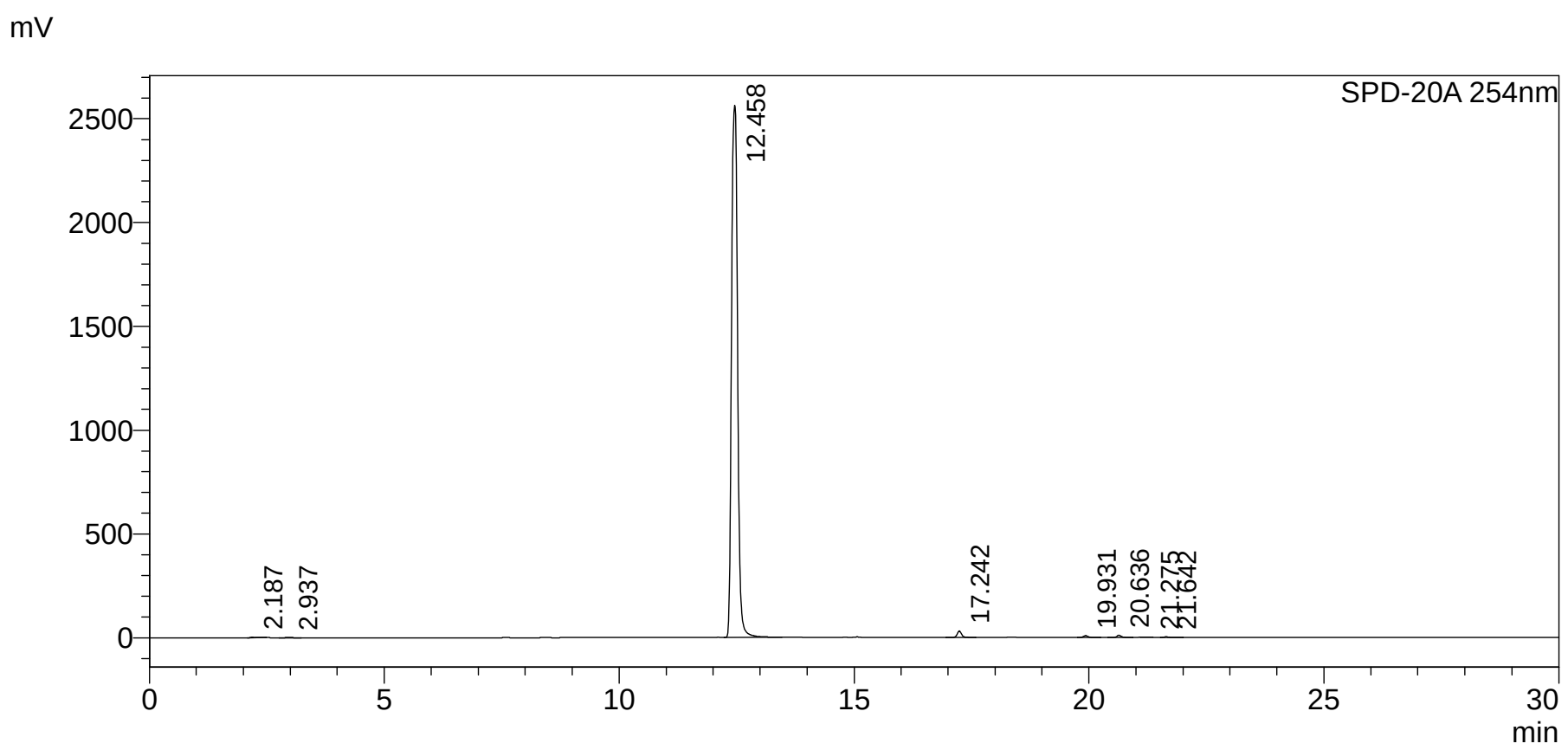

<Peak Table>

SPD-20A 254nm

Peak#	Ret. Time	Area	Area%
1	2.187	18255	0.077
2	2.937	9282	0.039
3	12.458	23278560	98.435
4	17.242	199693	0.844
5	19.931	54654	0.231
6	20.636	67087	0.284
7	21.275	6779	0.029
8	21.642	14232	0.060
Total		23648542	100.000

Figure S40. HPLC Chromatogram of **Compound 12.**

N[5]-cyclopropyl-N[7]-methyl-2-phenyl-[1,2,4]triazolo[1,5-*a*][1,3,5]triazine-5,7-diamine **13**

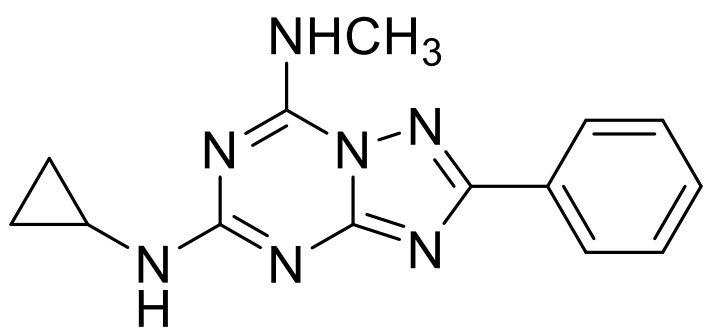

Chemical Formula: $C_{14}H_{15}N_7$
Molecular Weight: 281,32

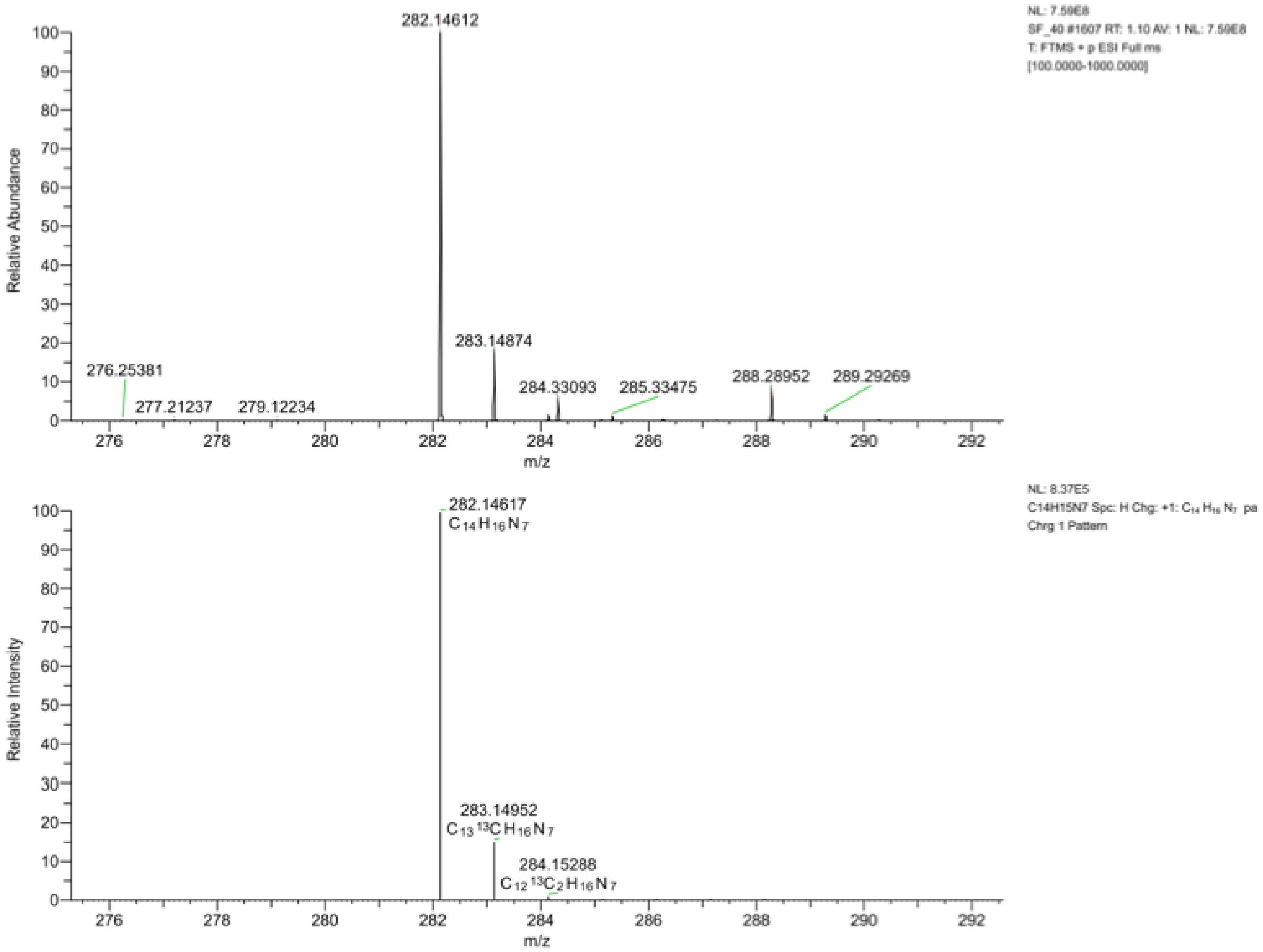

Figure S41. HRMS (ESI) spectrum of **Compound 13**.

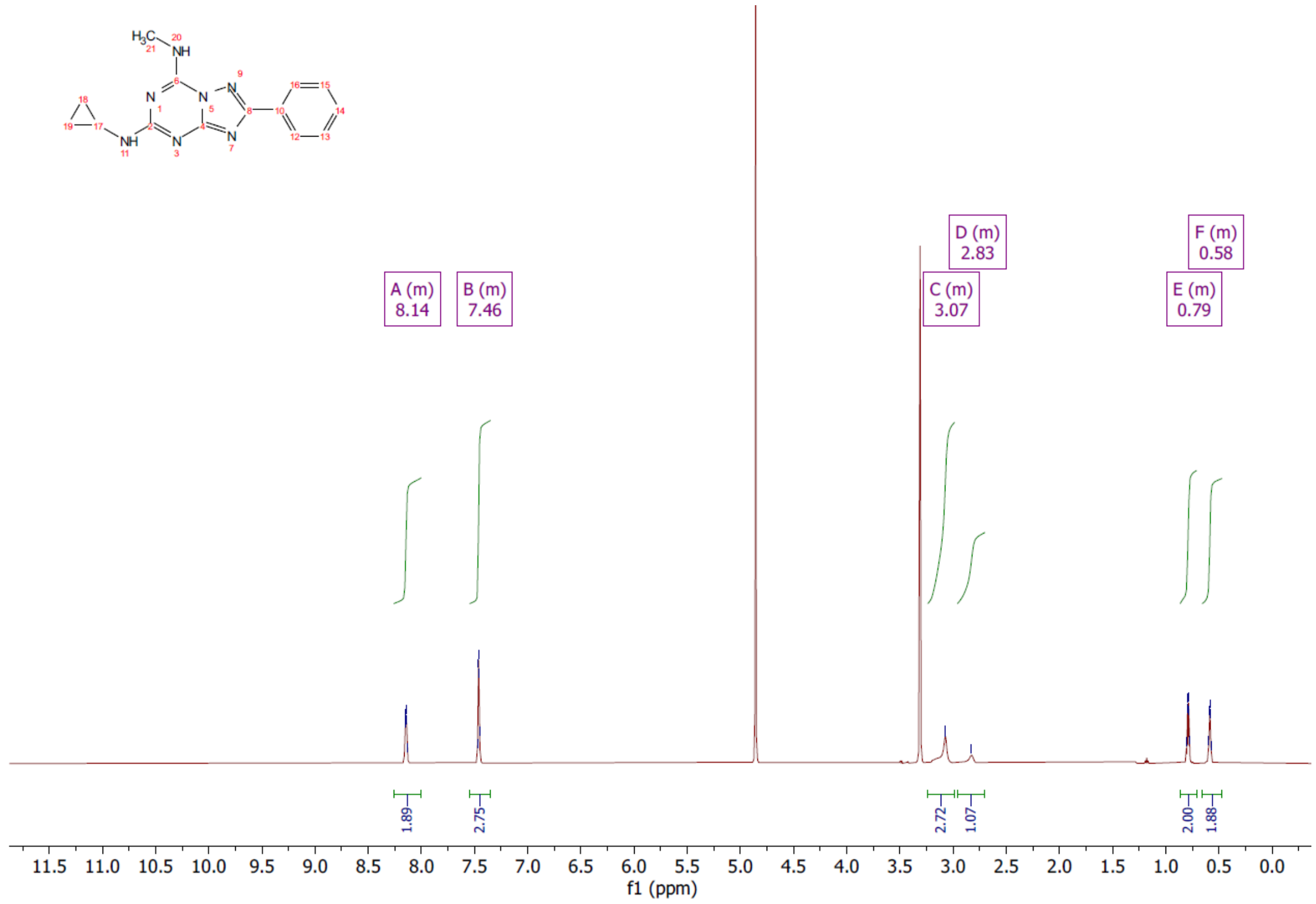

Figure S42. ^{1}H NMR spectrum of **Compound 13** (CD_3OD).

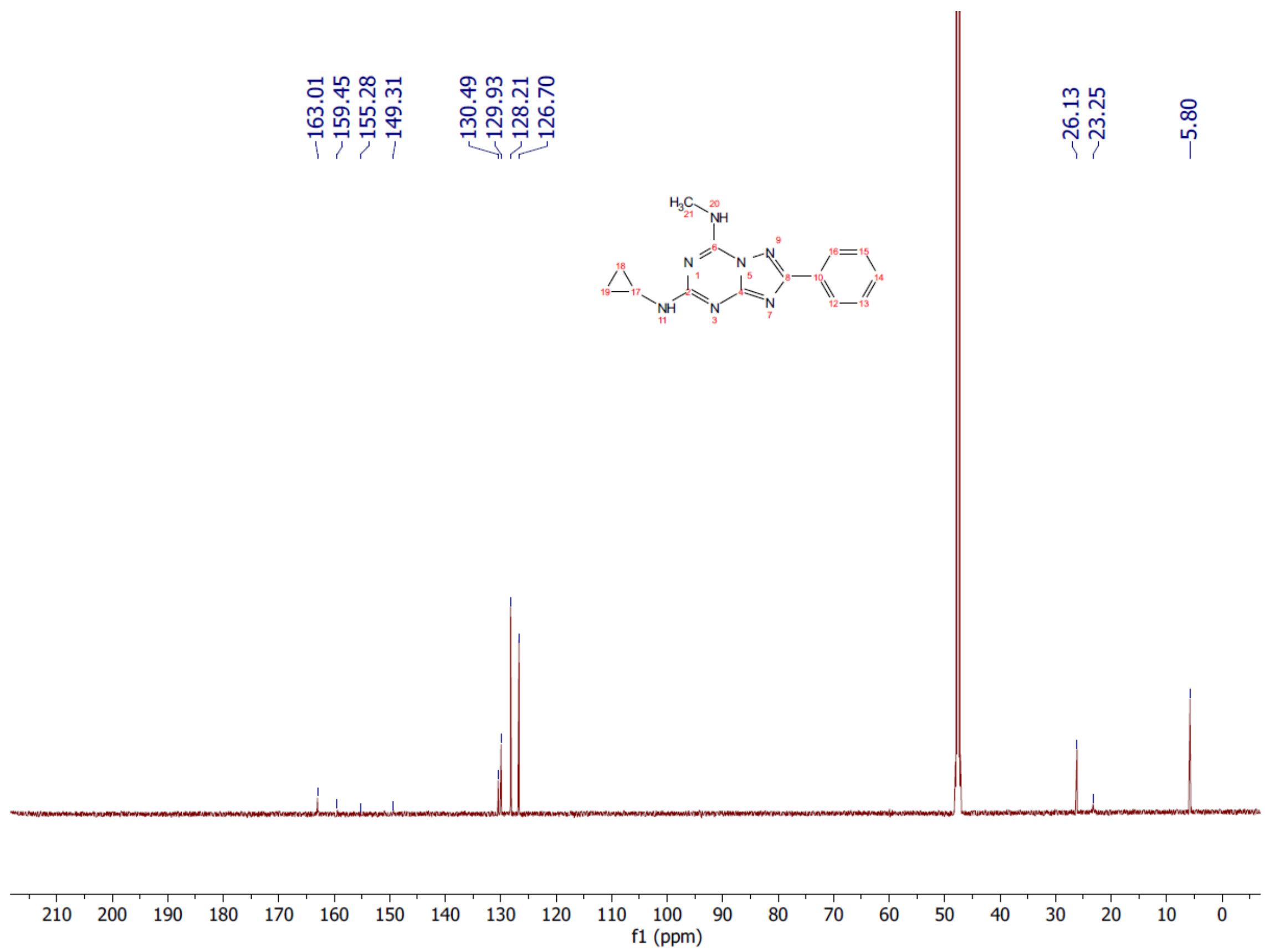

Figure S43. ^{13}C NMR spectrum of **Compound 13** (CD_3OD).

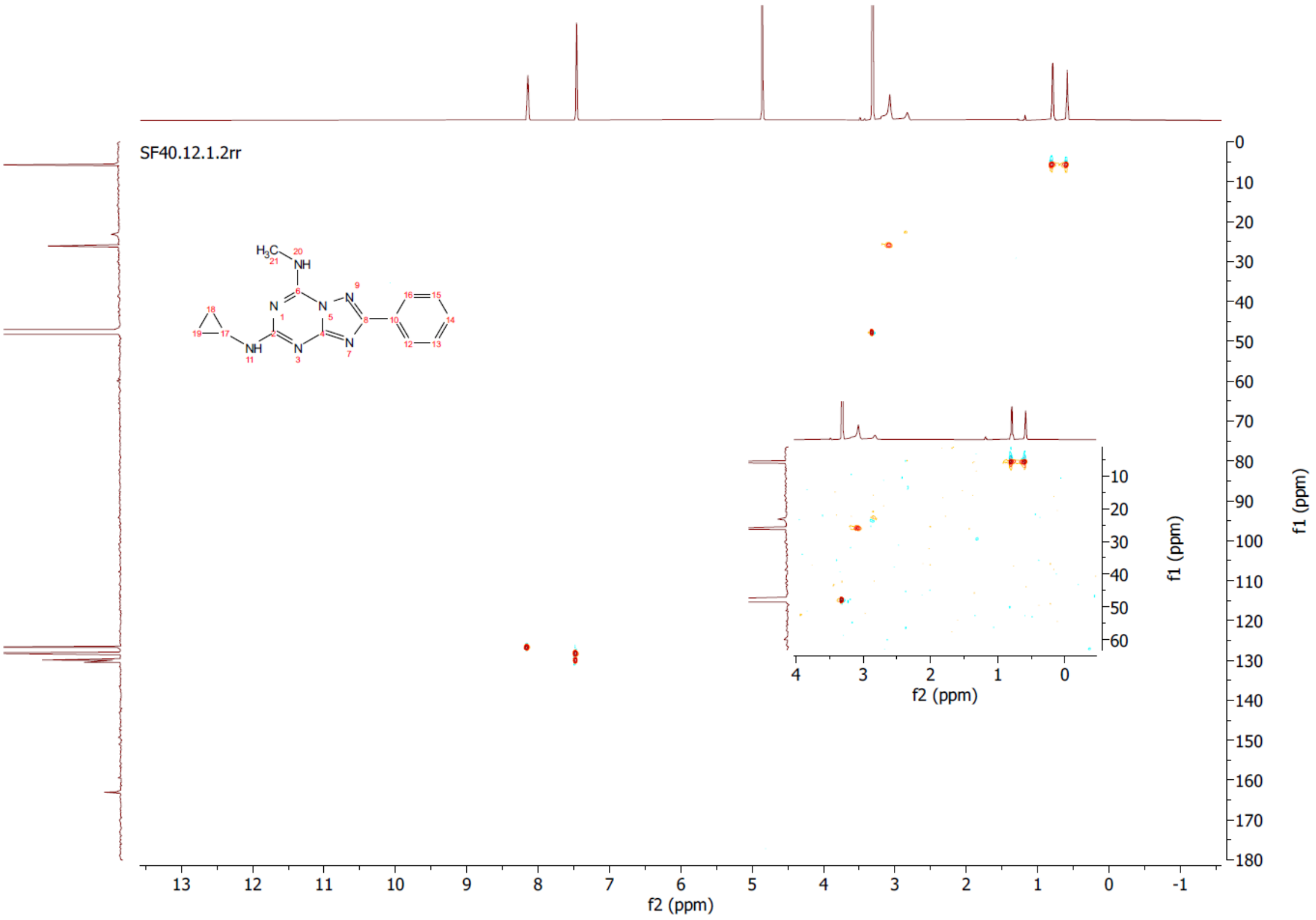

Figure S44. HSQC ^1H-^{13}C NMR spectrum of **Compound 13**.

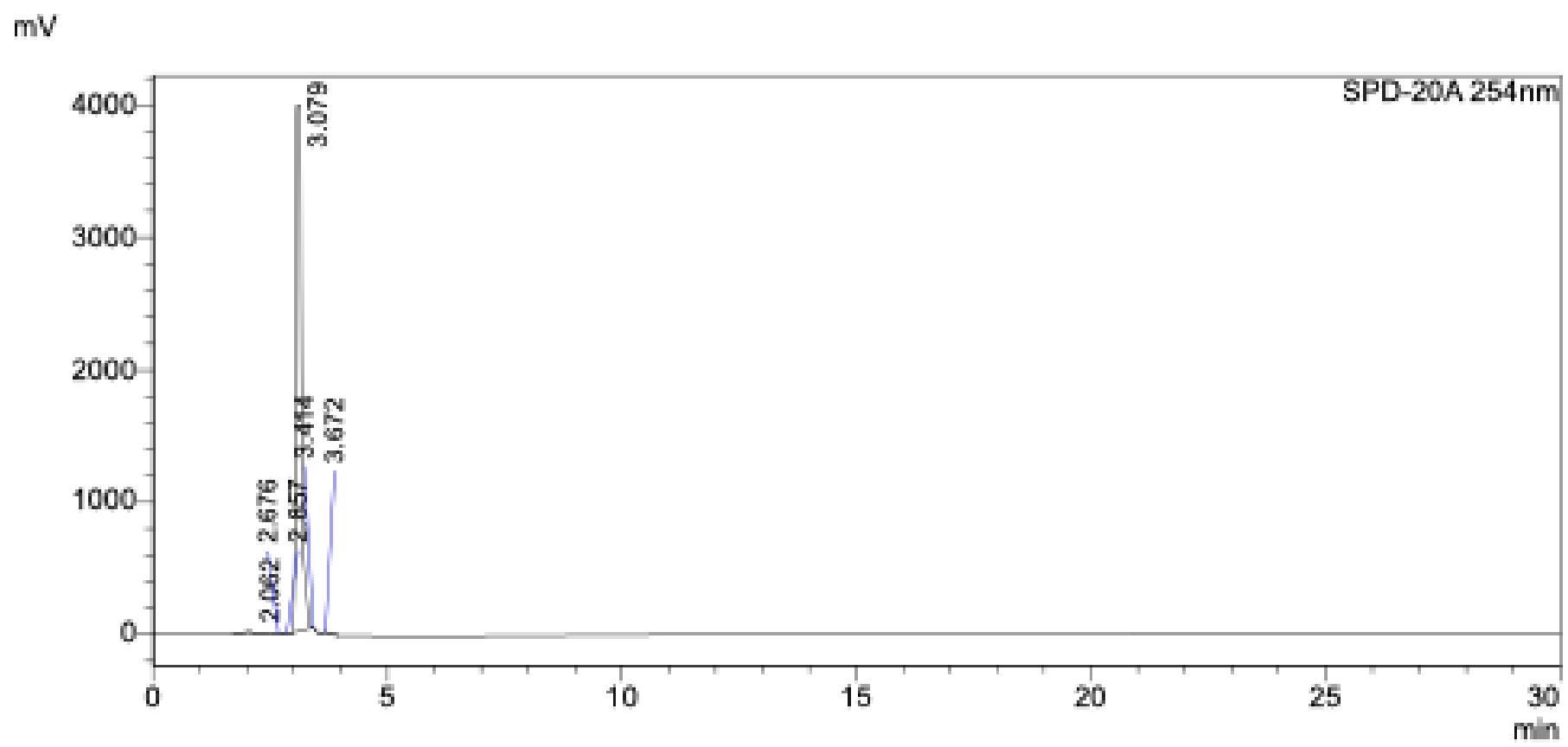

<Peak Table>

SPD-20A 254nm

Peak#	Ret. Time	Area	Height	Area%
1	2.062	304449	22198	0.967
2	2.676	24174	3406	0.077
3	2.857	14635	3693	0.046
4	3.079	30988249	3979809	98.435
5	3.414	21612	5527	0.069
6	3.672	127708	14371	0.406
Total		31480826	4029003	100.000

Figure S45. HPLC Chromatogram of **Compound 13**.

N[5]-cyclohexyl-N[7]-methyl-2-phenyl-[1,2,4]triazolo[1,5-*a*][1,3,5]triazine-5,7-diamine **14**

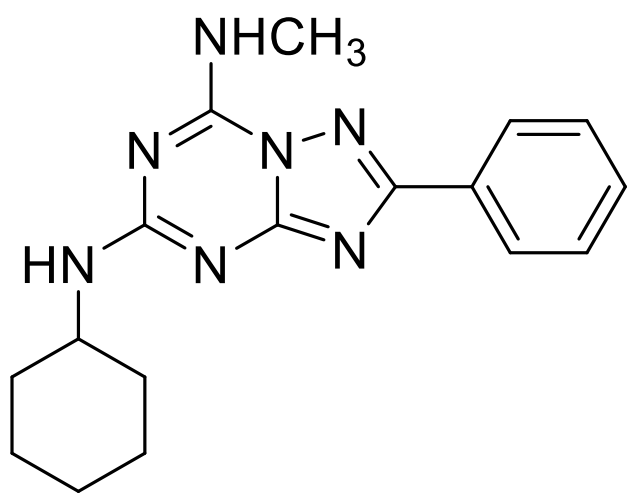

Chemical Formula: $C_{17}H_{21}N_7$
Molecular Weight: 323,40

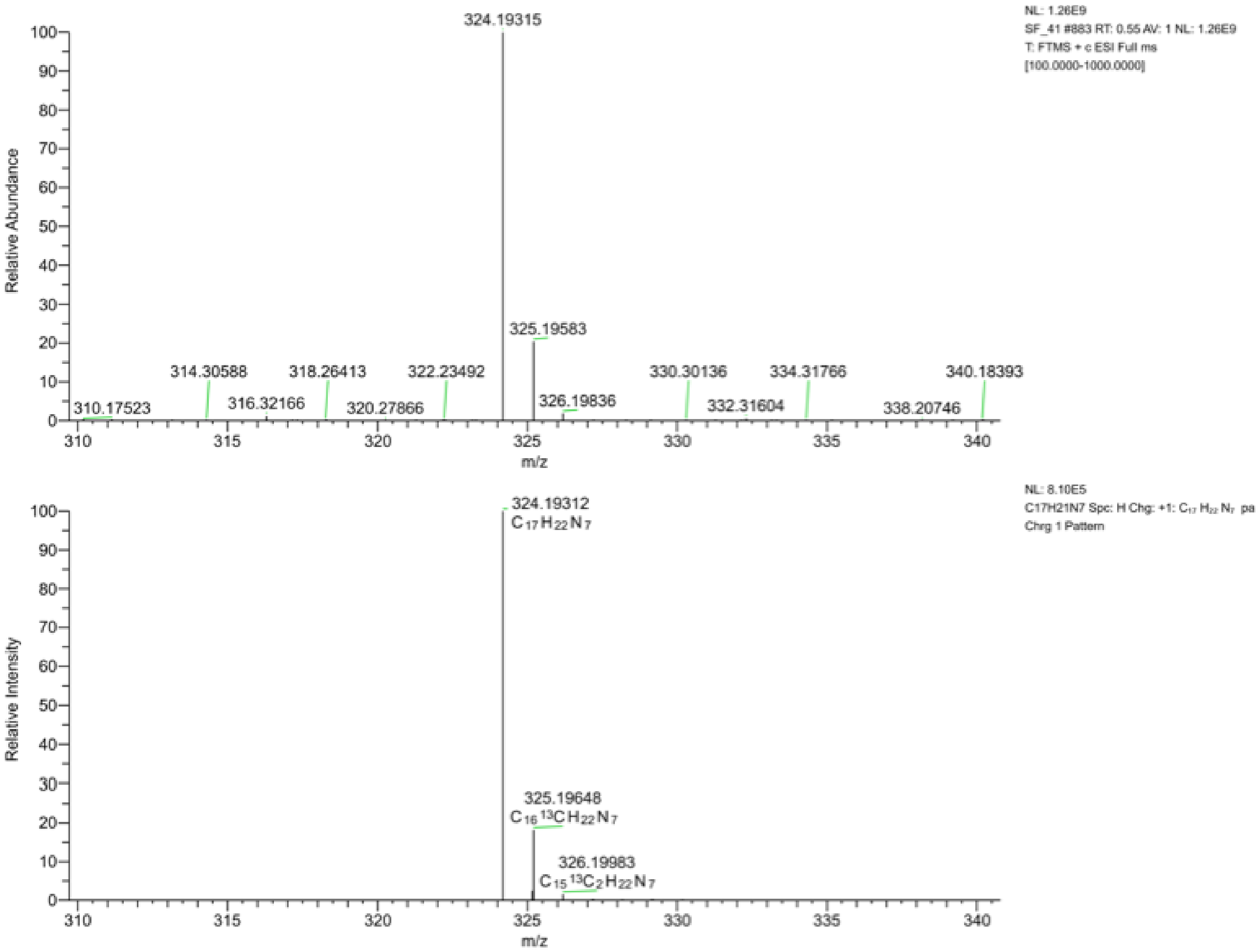

Figure S46. HRMS (ESI) spectrum of **Compound 14**.

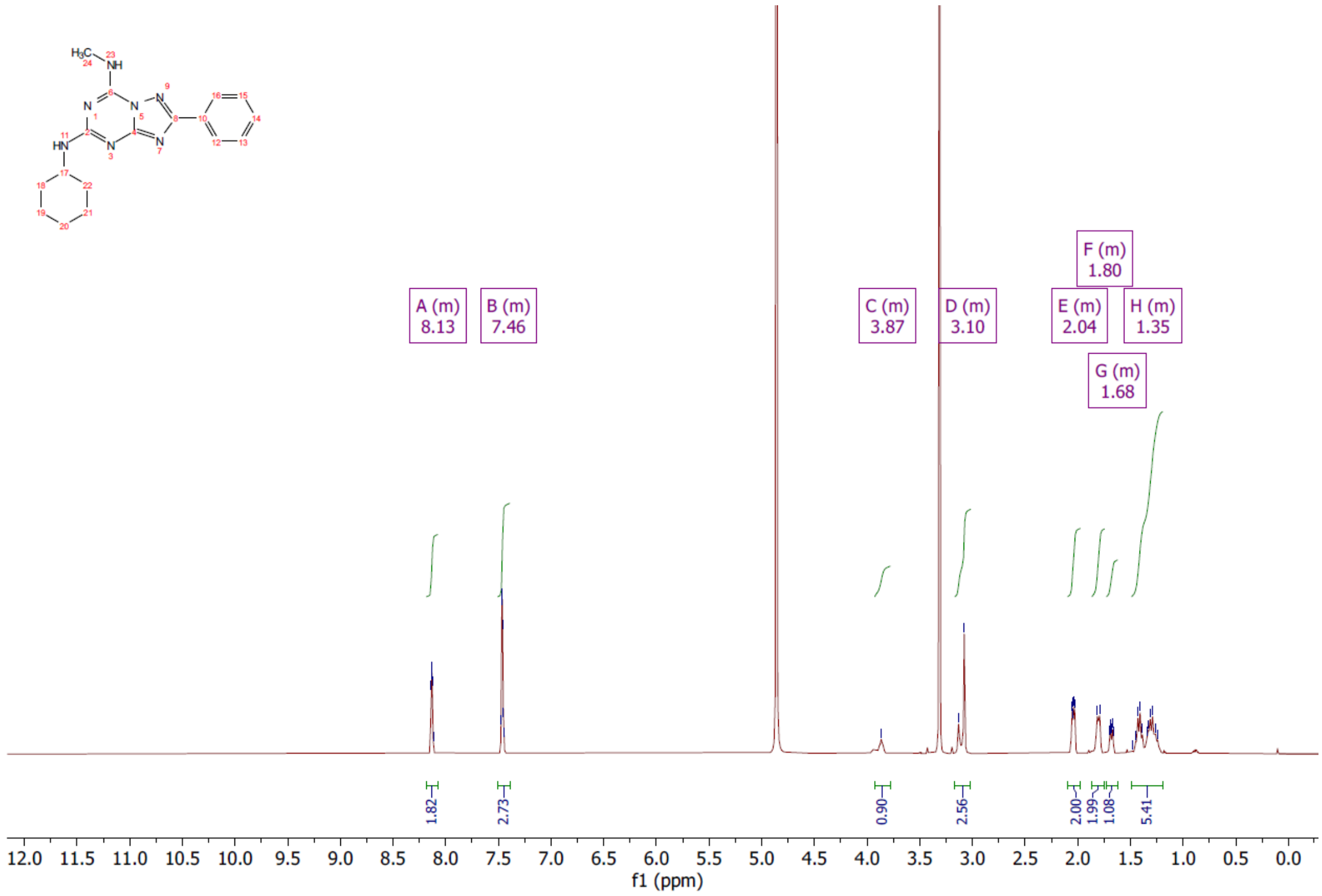

Figure S47. ^{1}H NMR spectrum of **Compound 14** (CD_3OD).

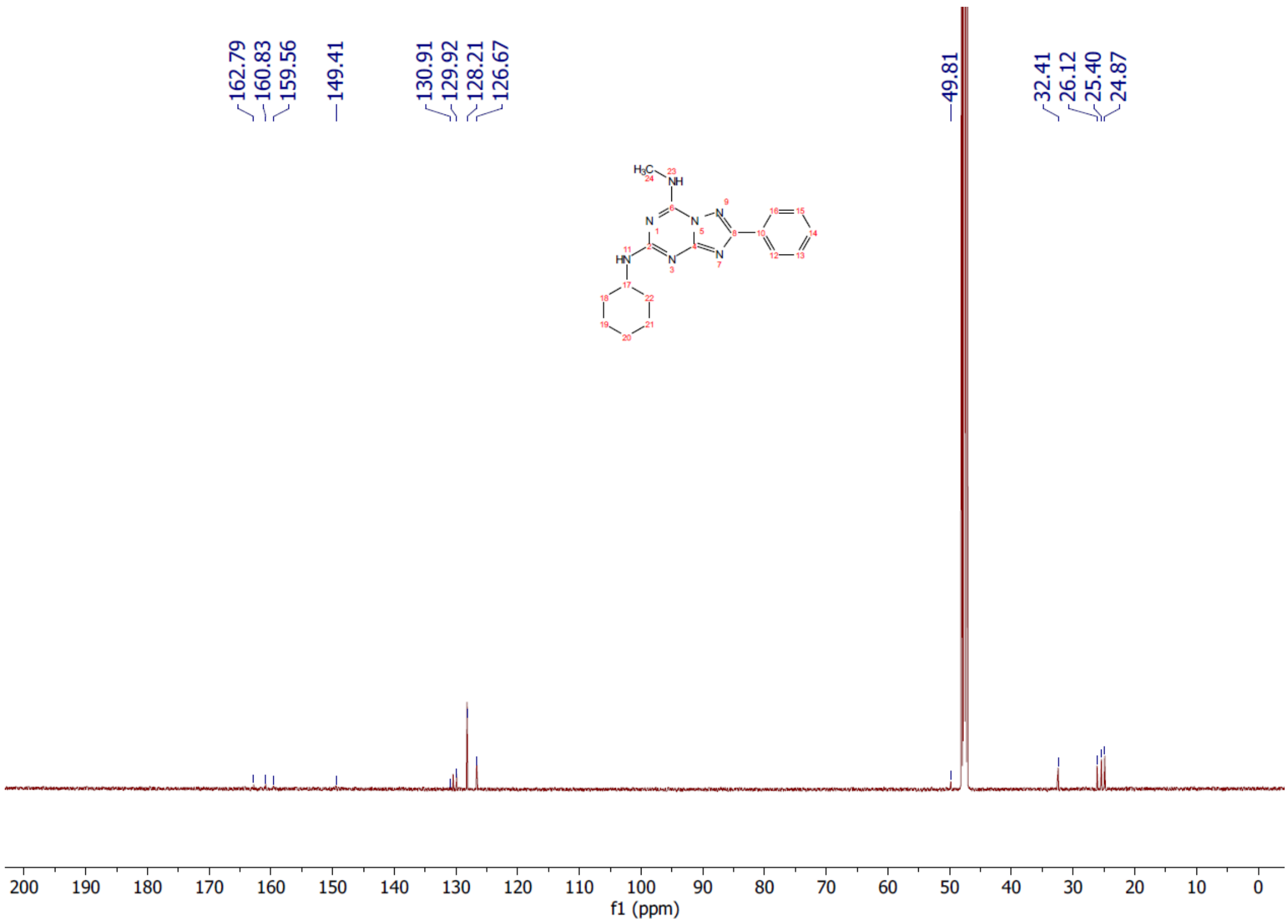

Figure S48. ^{13}C NMR spectrum of **Compound 14** (CD_3OD).

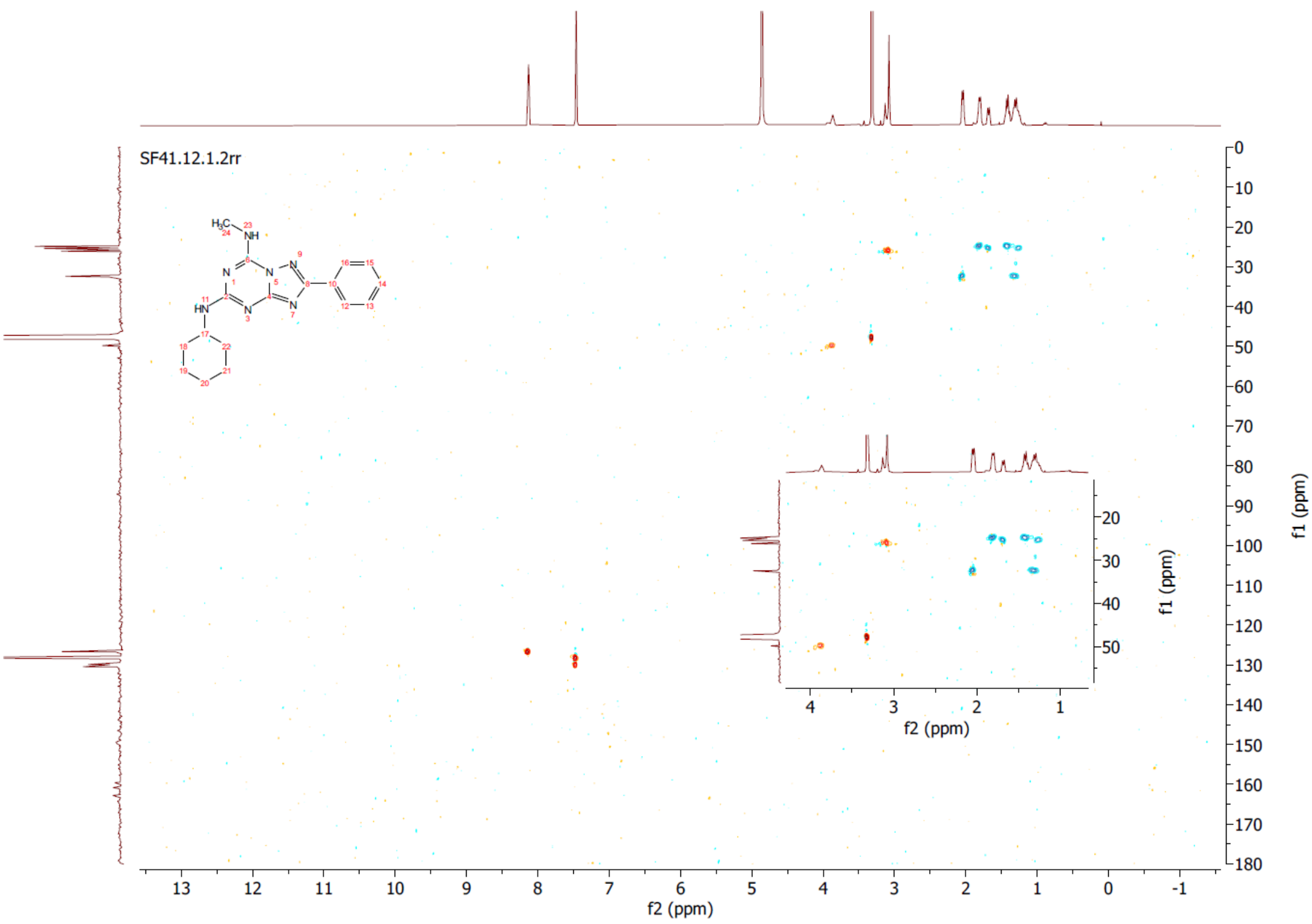

Figure S49. HSQC ^{1}H-^{13}C NMR spectrum of **Compound 14** (CD_3OD).

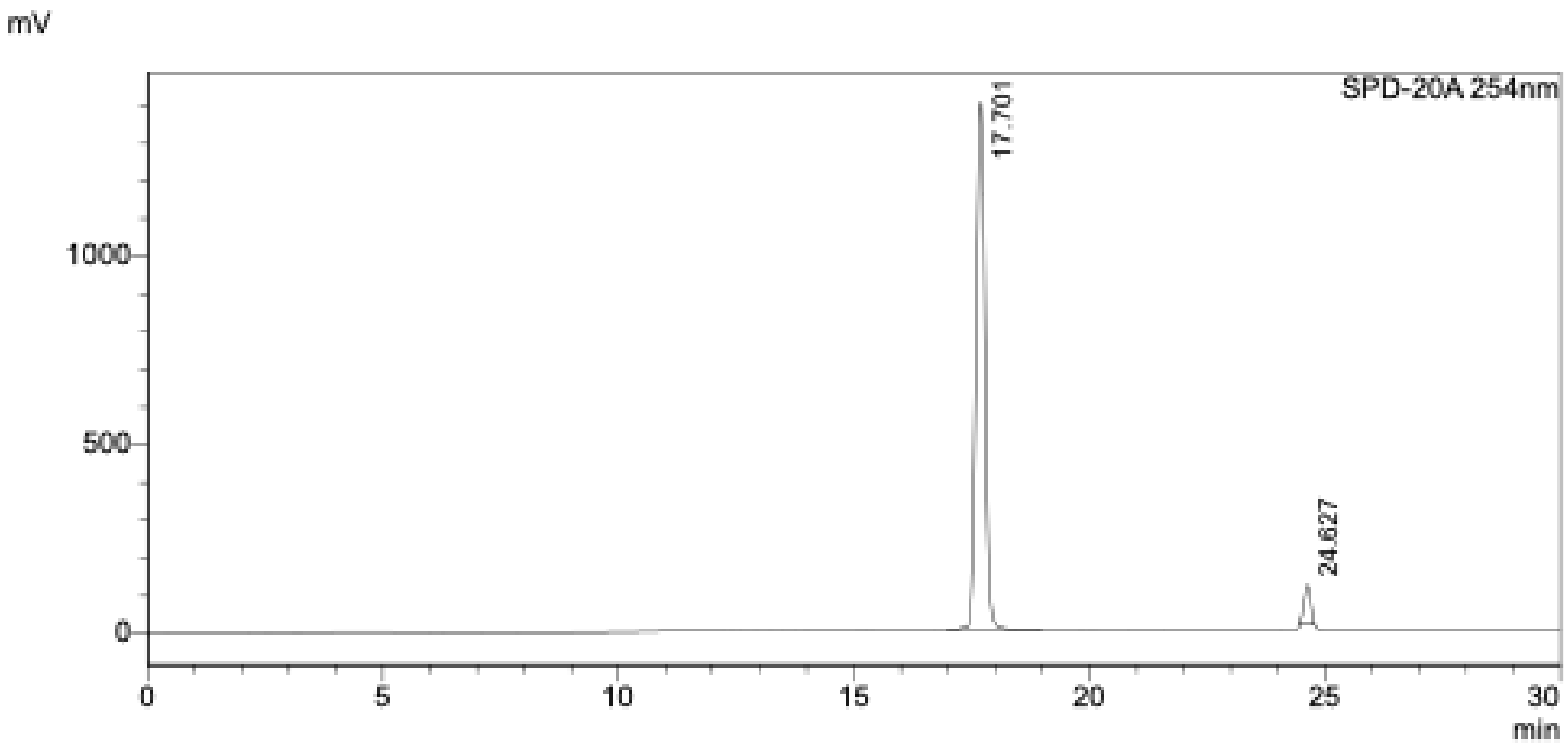

<Peak Table>

SPD-20A 254nm

Peak#	Ret. Time	Area	Height	Area%
1	17.701	17975514	1400881	94.984
2	24.627	949232	101158	5.016
Total		18924746	1502039	100.000

Figure S50 HPLC Chromatogram of **Compound 14**.

N[5]-benzyl-N[7]-methyl-2-phenyl-[1,2,4]triazolo[1,5-*a*][1,3,5]triazine-5,7-diamine **15**

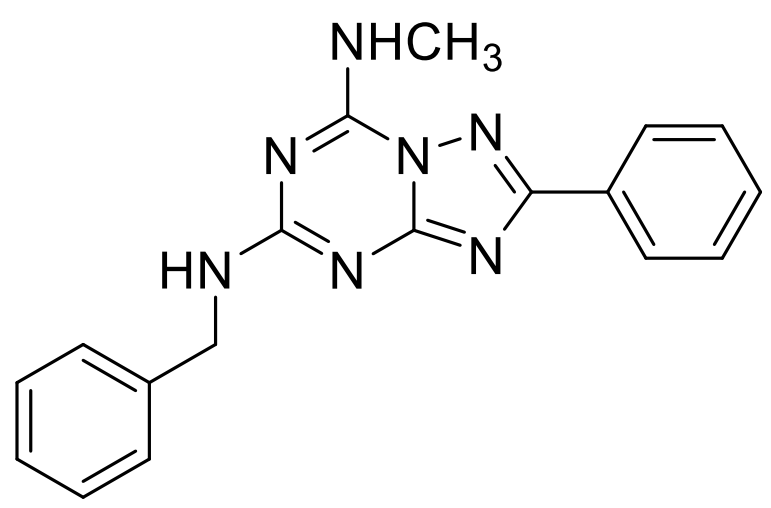

Chemical Formula: $C_{18}H_{17}N_7$
Molecular Weight: 331,37

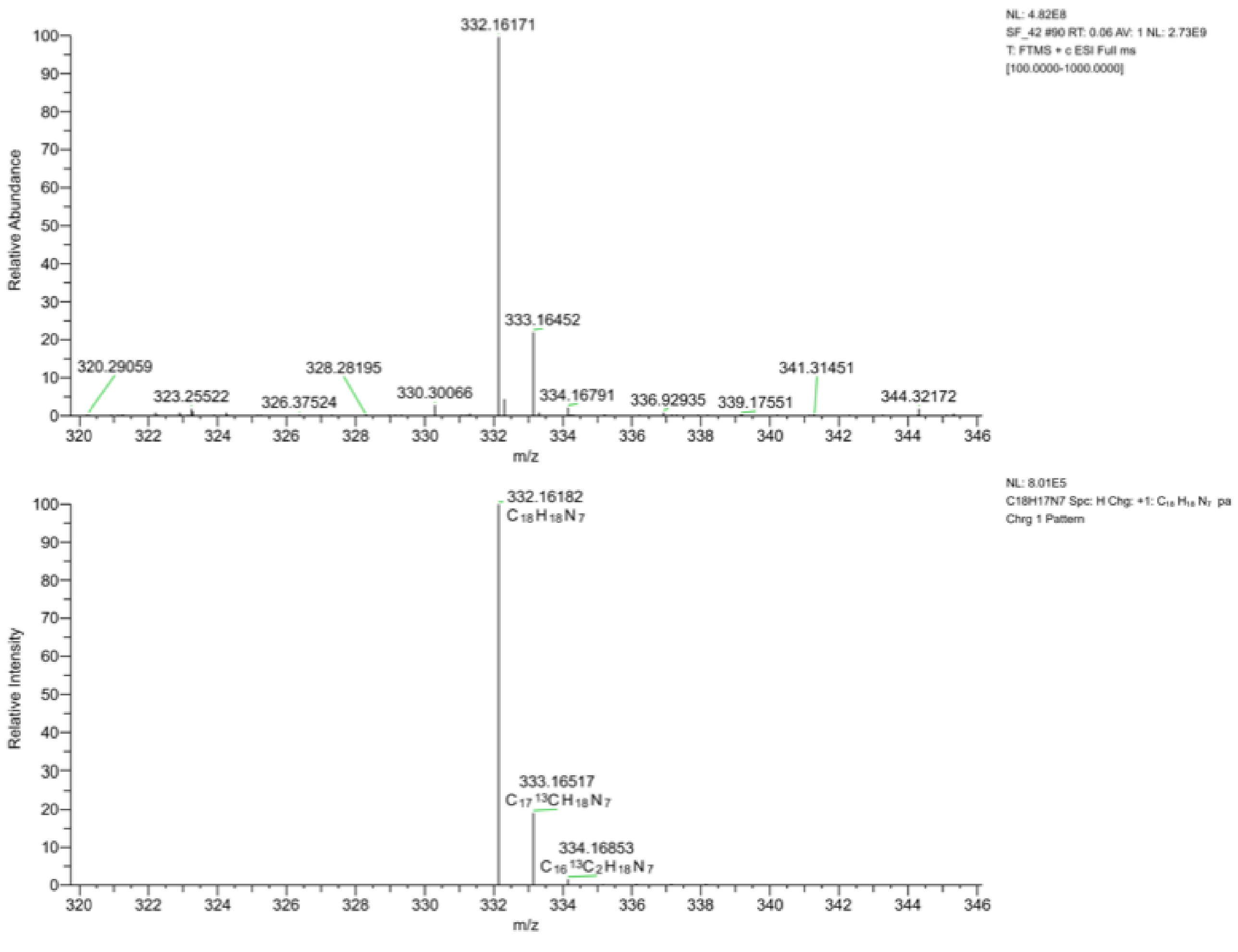

Figure S51. HRMS (ESI) spectrum of **Compound 15**.

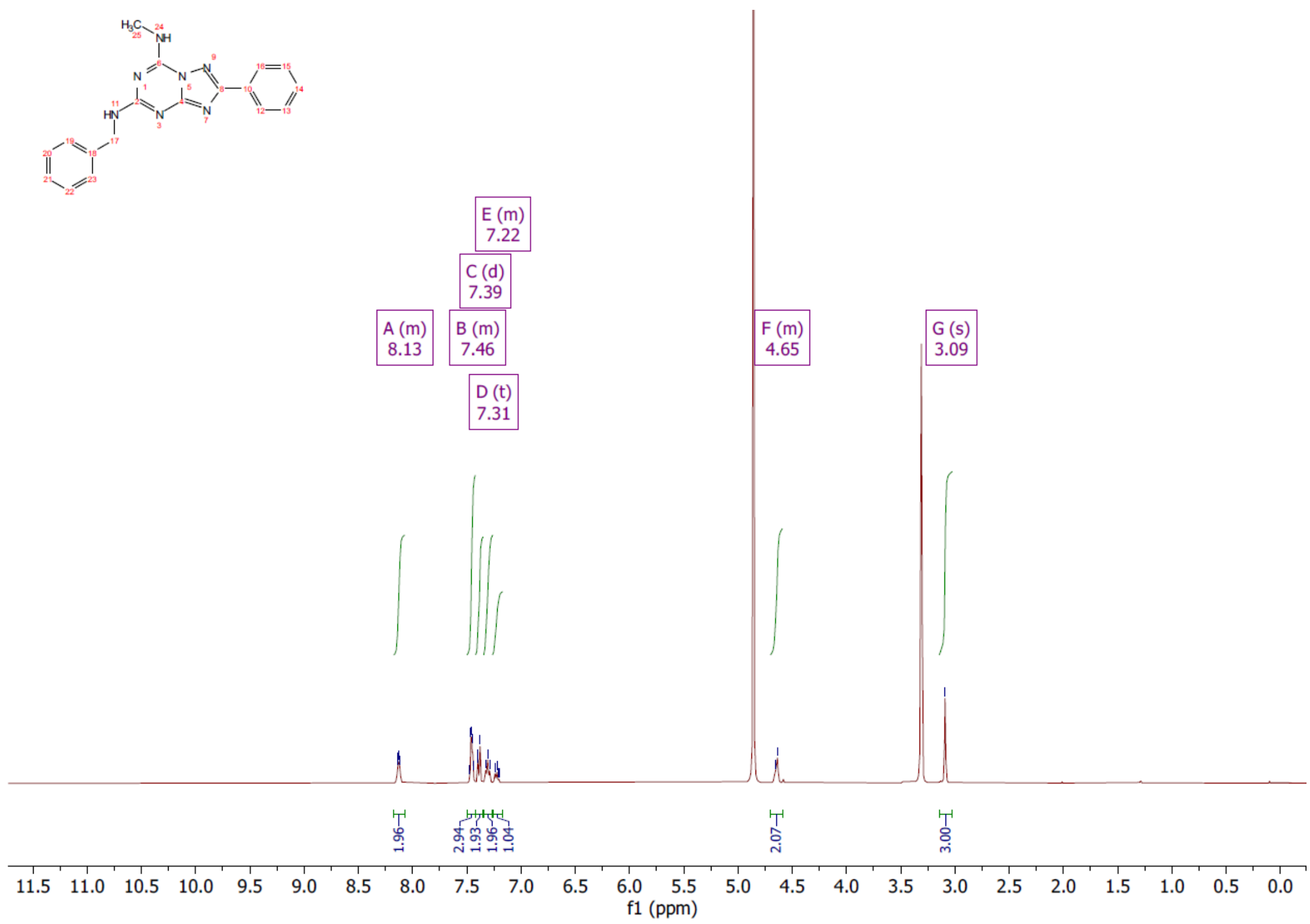

Figure S52. ^{1}H NMR spectrum of **Compound 15** (CD_3OD).

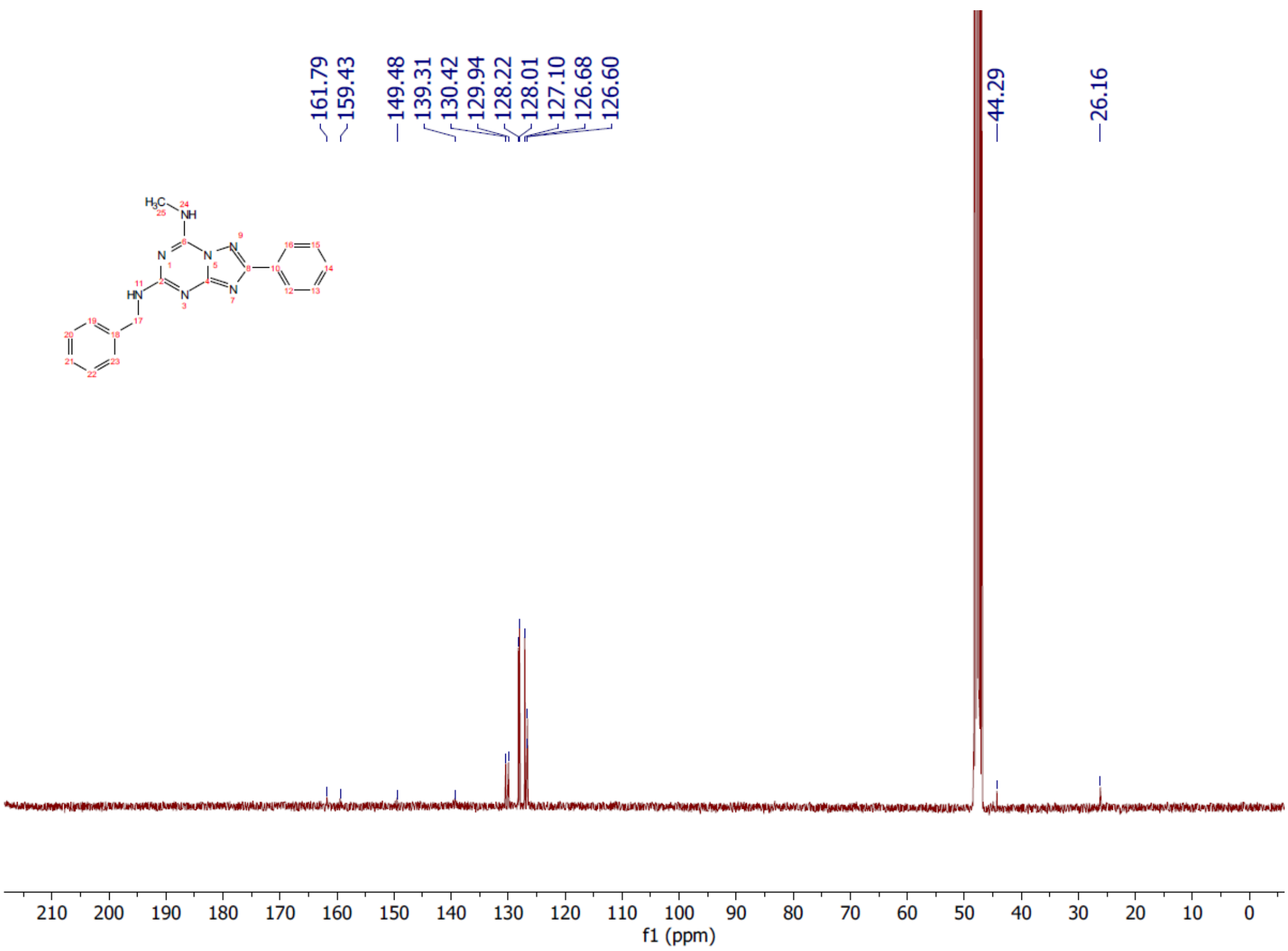

Figure S53. ^{13}C NMR spectrum of **Compound 15** (CD_3OD).

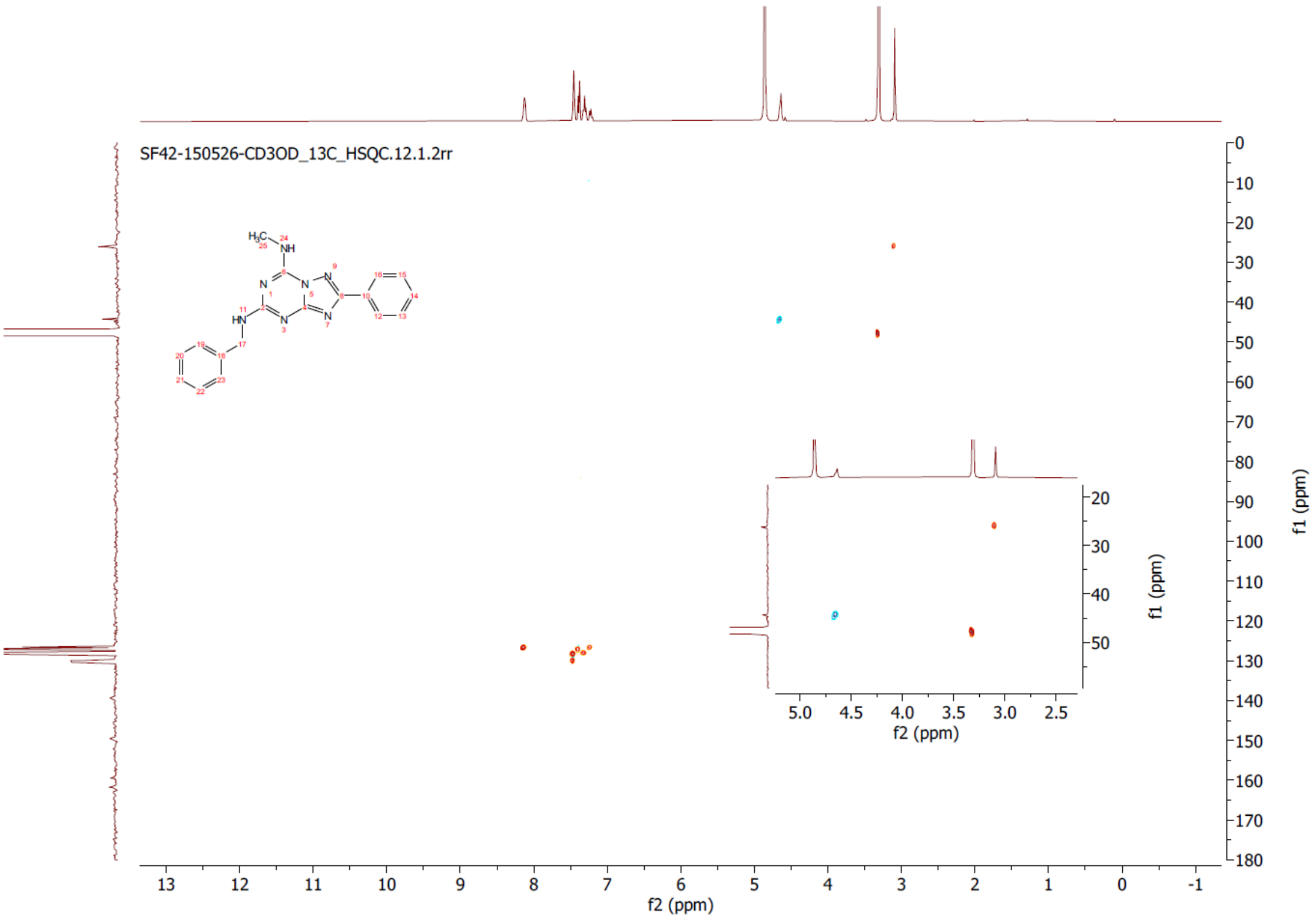

Figure S54. HSQC ^{1}H-^{13}C NMR spectrum of **Compound 15** (CD_3OD).

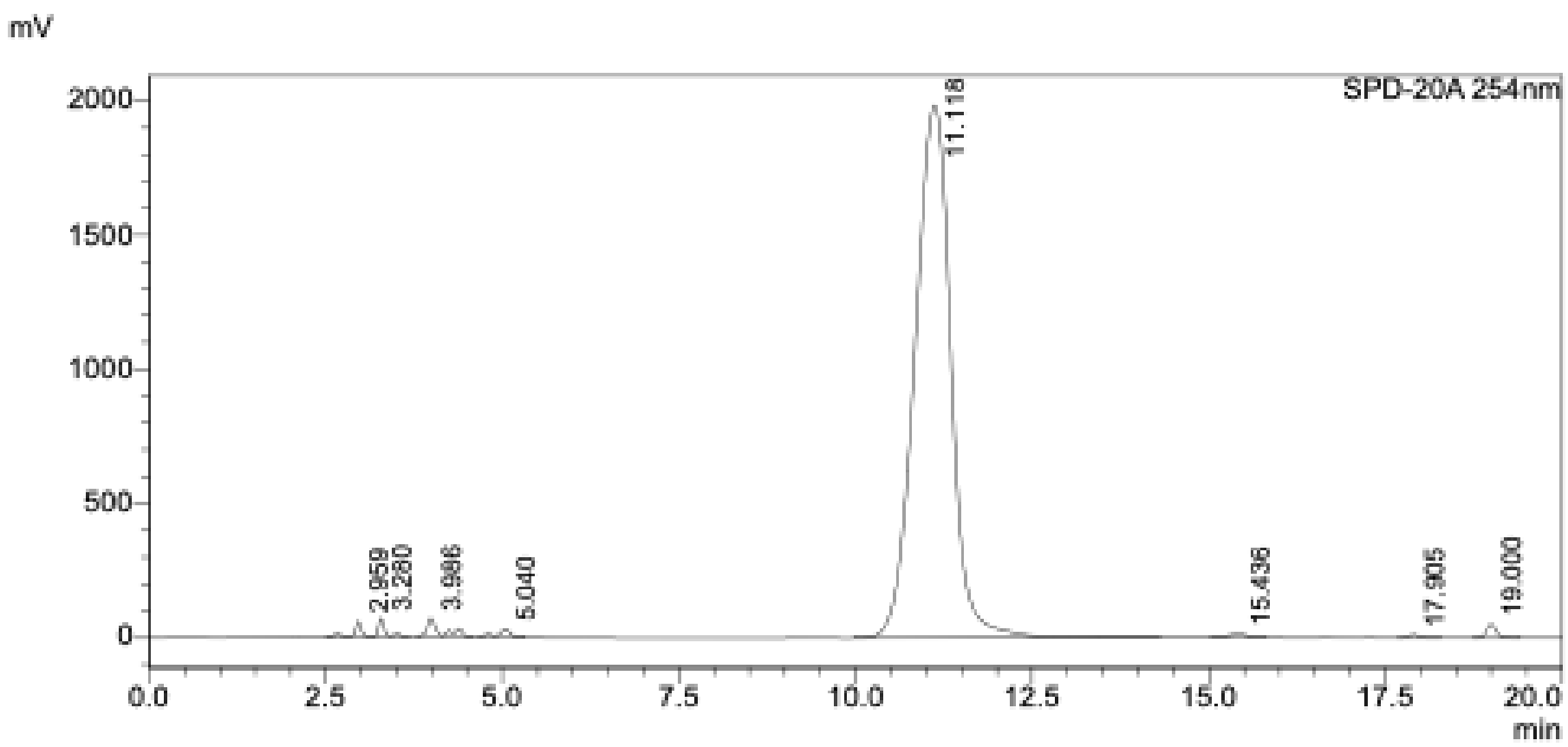

<Peak Table>

SPD-20A 254nm

Peak#	Ret. Time	Area	Height	Area%
1	2.959	383880	57255	0.523
2	3.280	436495	67423	0.594
3	3.986	829273	66654	1.129
4	5.040	362502	29416	0.494
5	11.118	70578452	1983667	96.100
6	15.436	251178	13874	0.342
7	17.905	120920	11906	0.165
8	19.000	480036	50403	0.654
Total		73442737	2280598	100.000

Figure S55. HPLC Chromatogram of **Compound 15**.

N[7]-cyclopropyl-N[5]-methyl-2-phenyl-[1,2,4]triazolo[1,5-*a*][1,3,5]triazine-5,7-diamine **16**

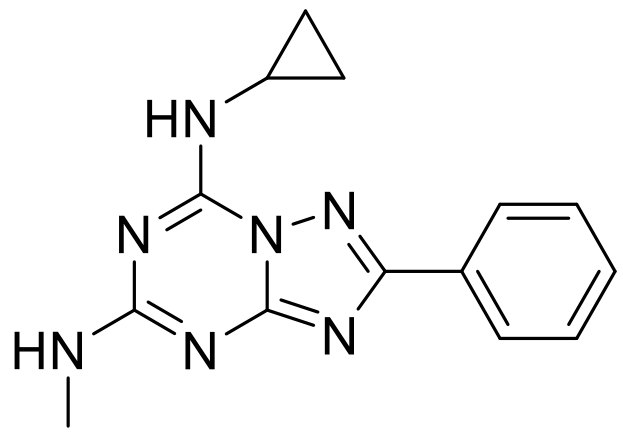

Chemical Formula: $C_{14}H_{15}N_7$
Molecular Weight: 281,32

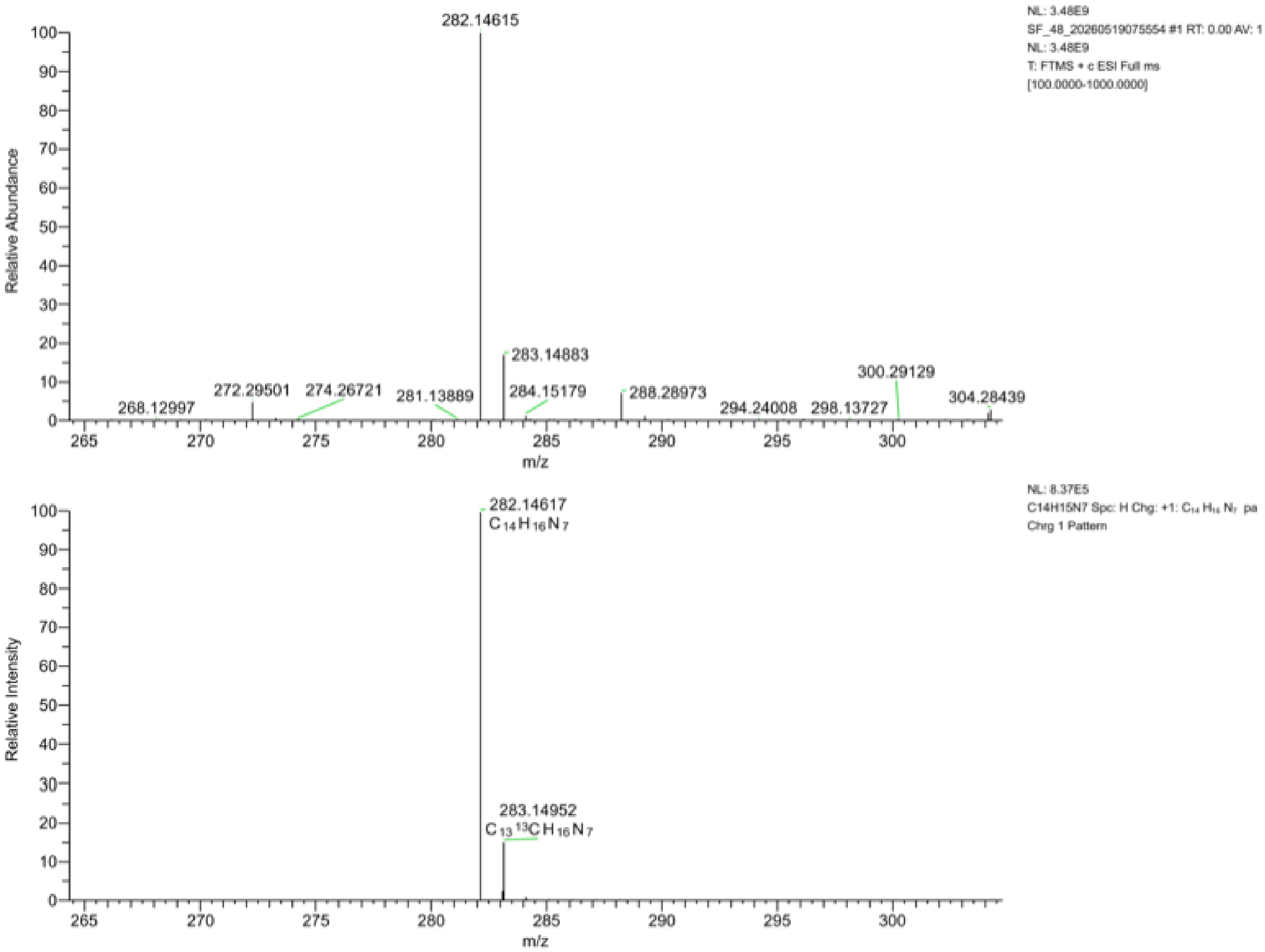

Figure S56. HRMS (ESI) spectrum of **Compound 16**.

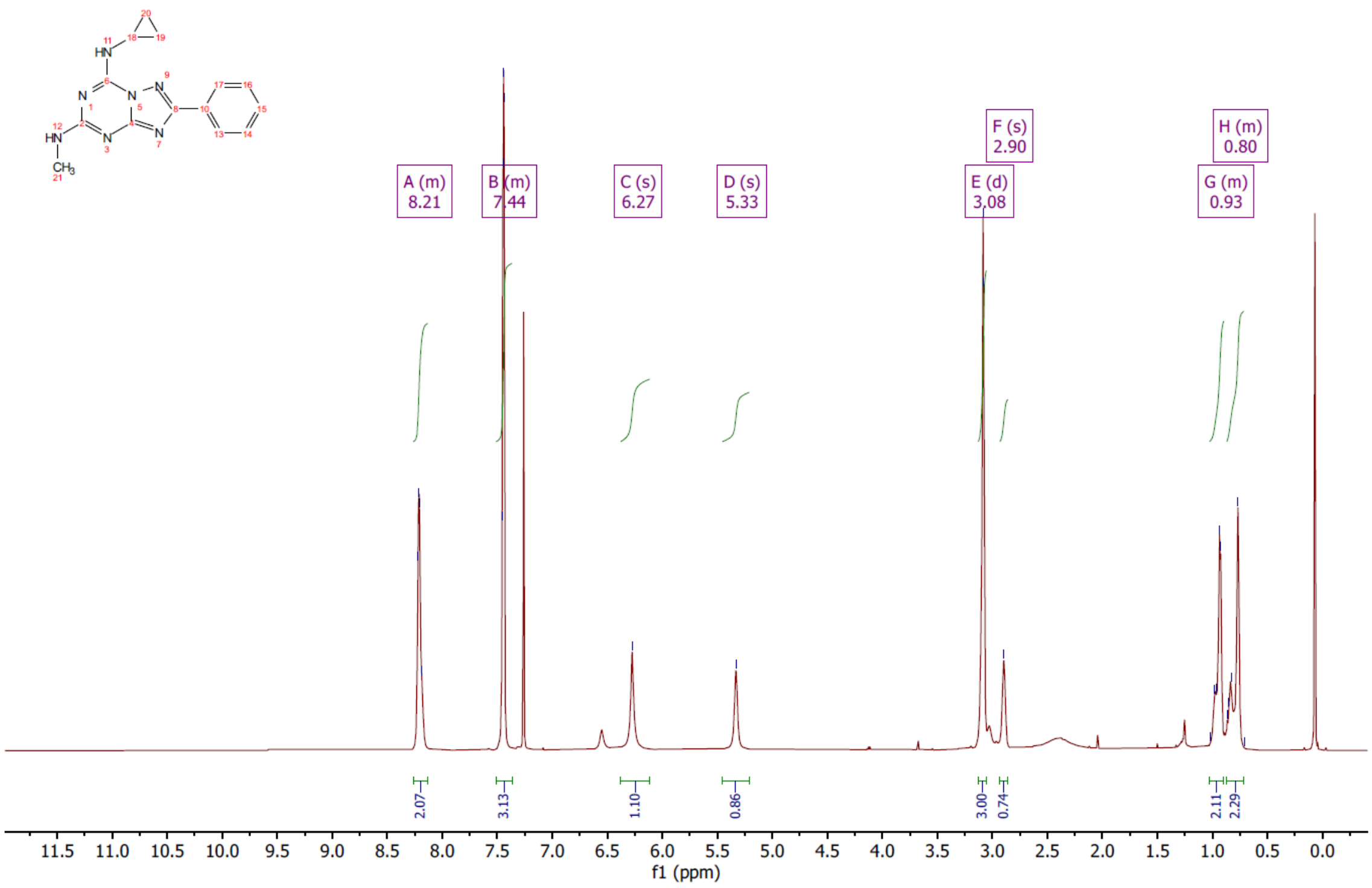

Figure S57. ^{1}H NMR spectrum of **Compound 16** ($CDCl_3$)

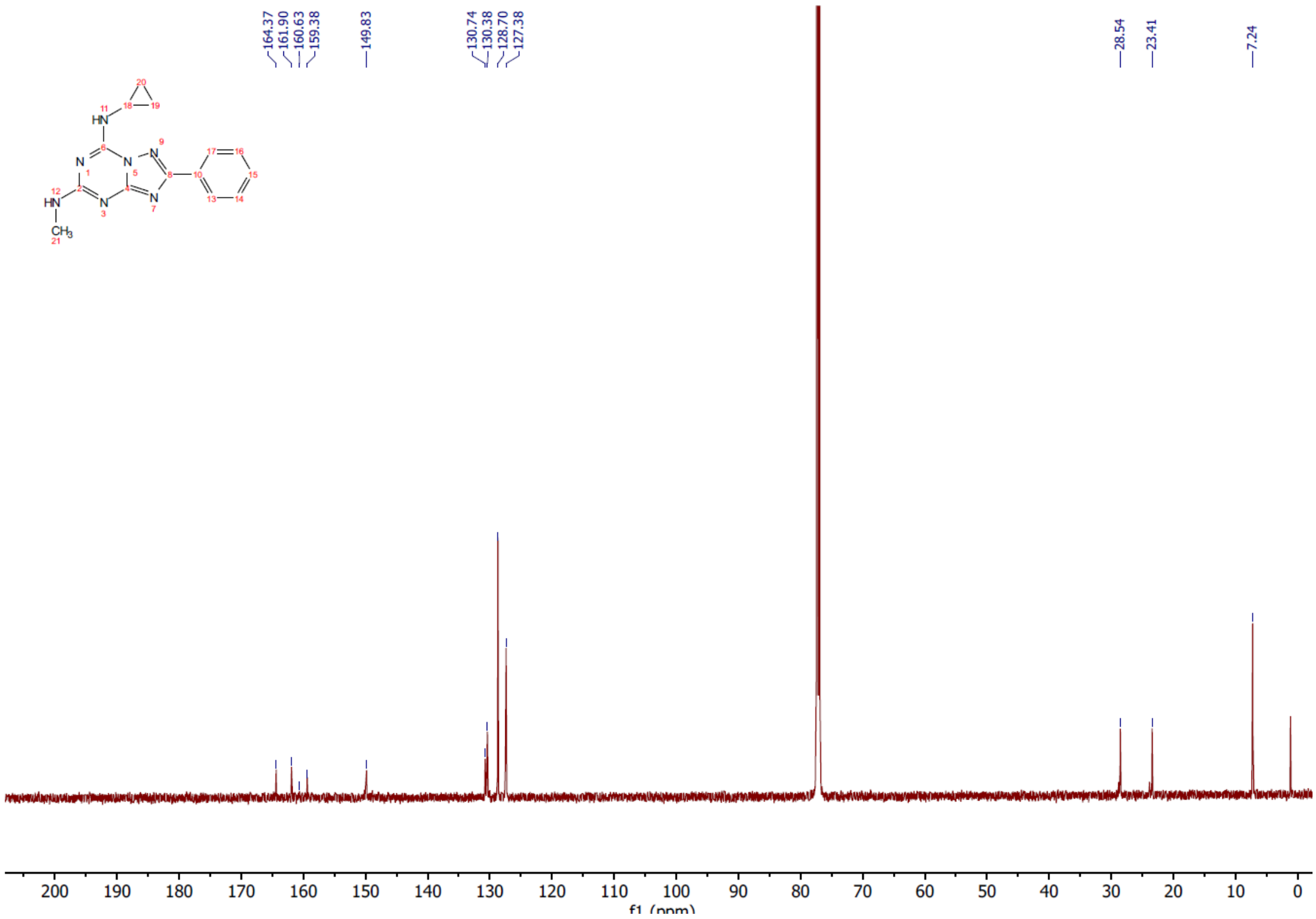

Figure S58. ^{13}C NMR spectrum of **Compound 16** ($CDCl_3$).

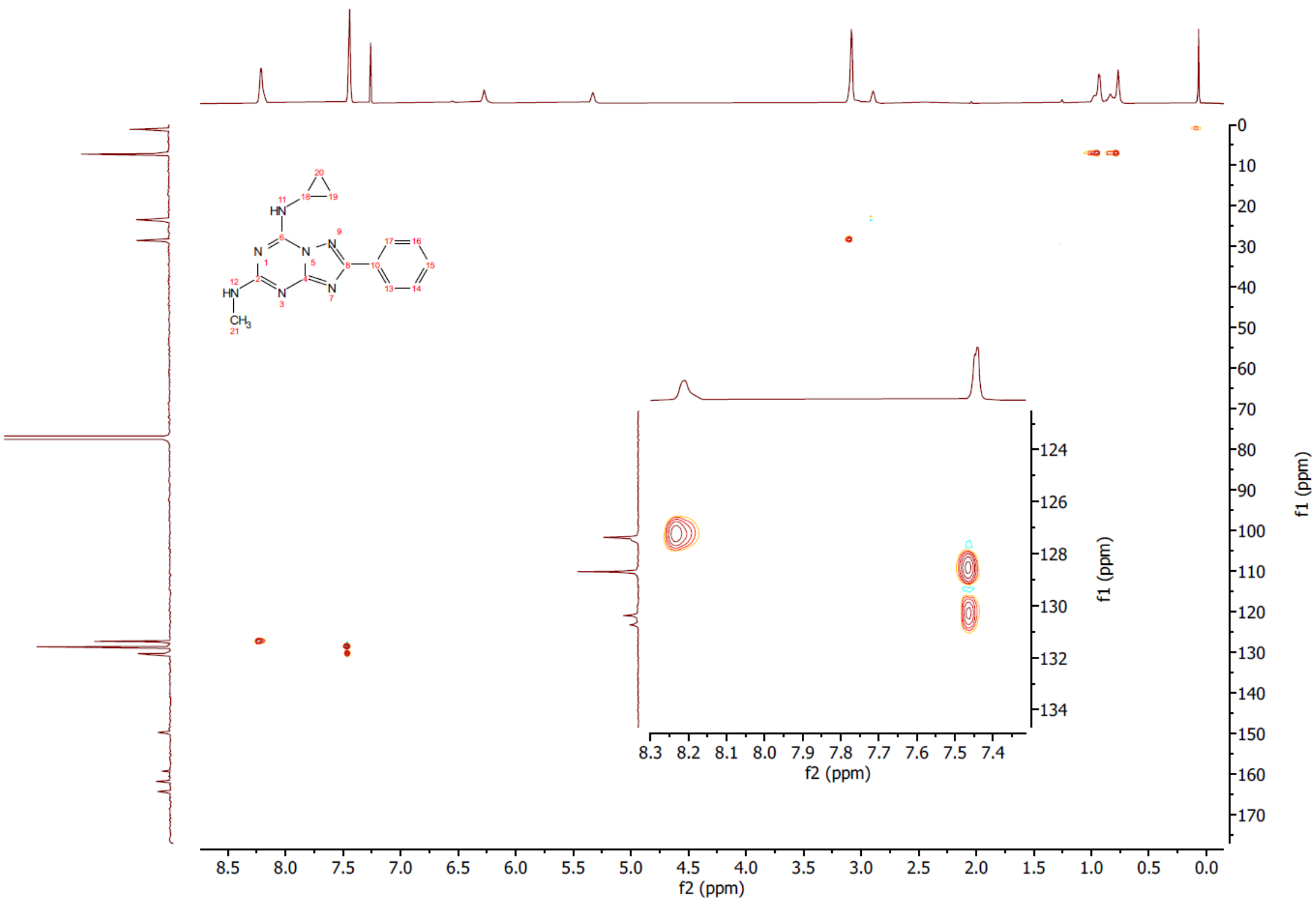

Figure S59. HSQC ^{1}H-^{13}C NMR spectrum of **Compound 16** ($CDCl_3$).

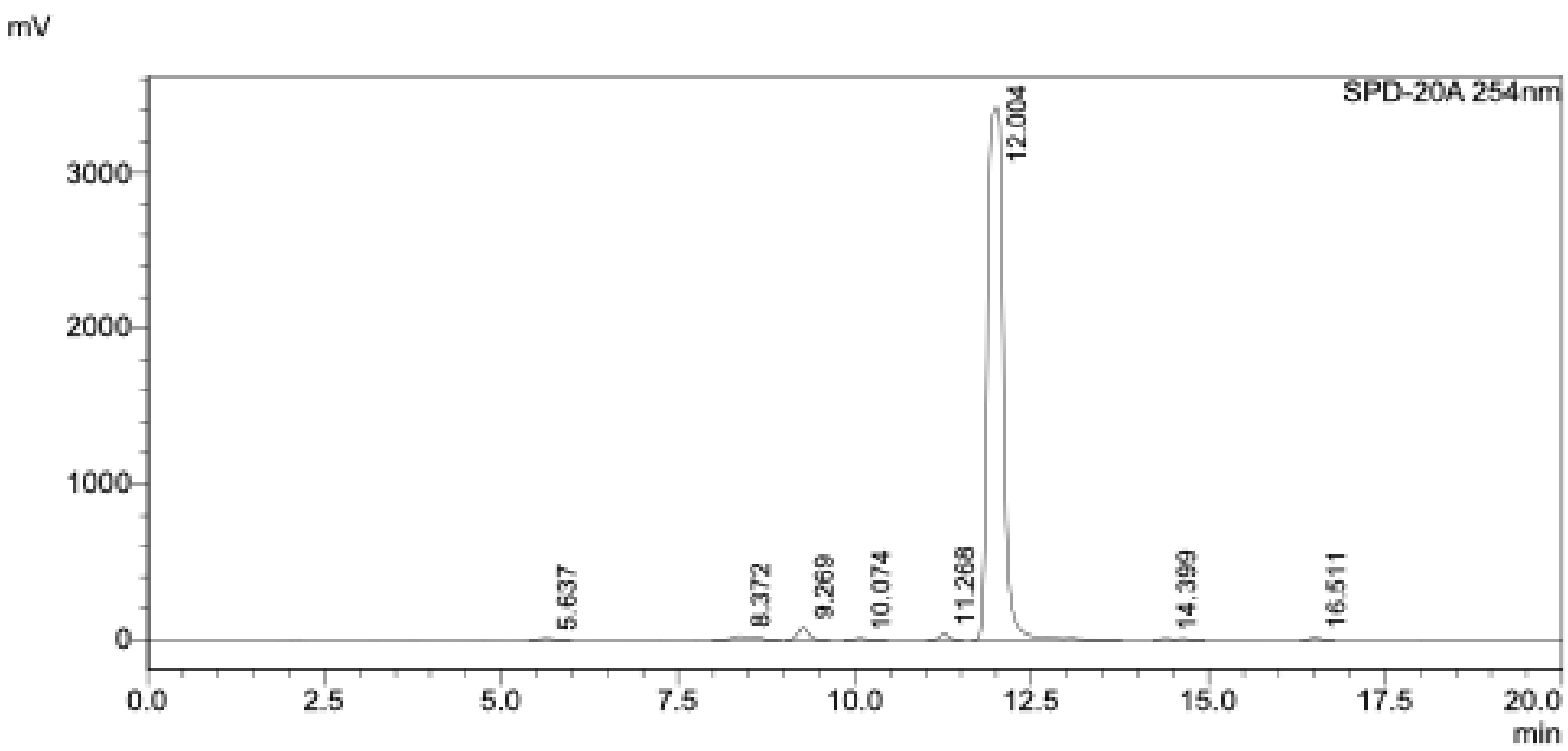

<Peak Table>

SPD-20A 254nm

Peak#	Ret. Time	Area	Height	Area%
1	5.637	138348	10004	0.249
2	8.372	534945	25952	0.964
3	9.269	821334	76434	1.481
4	10.074	97327	10570	0.175
5	11.268	331496	37074	0.598
6	12.004	53284264	3419976	96.054
7	14.399	120826	7719	0.218
8	16.511	144903	18414	0.261
Total		55473445	3606143	100.000

Figure S60. HPLC Chromatogram of **Compound 16**.

N^5,N^7-dicyclopropyl-2-phenyl-[1,2,4]triazolo[1,5-*a*][1,3,5]triazine-5,7-diamine **17**

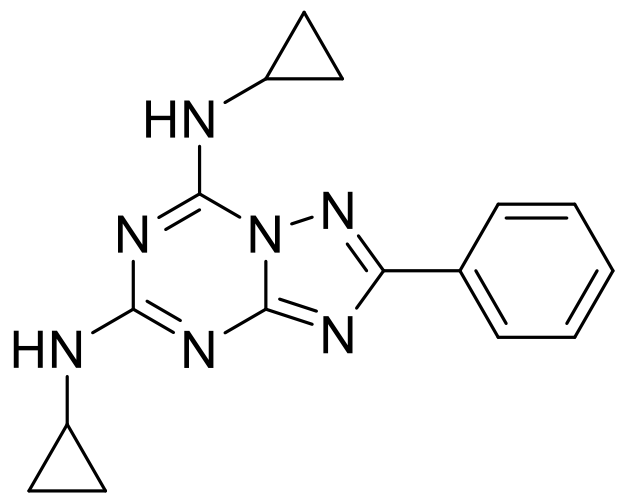

Chemical Formula: $C_{16}H_{17}N_7$
Molecular Weight: 307.35

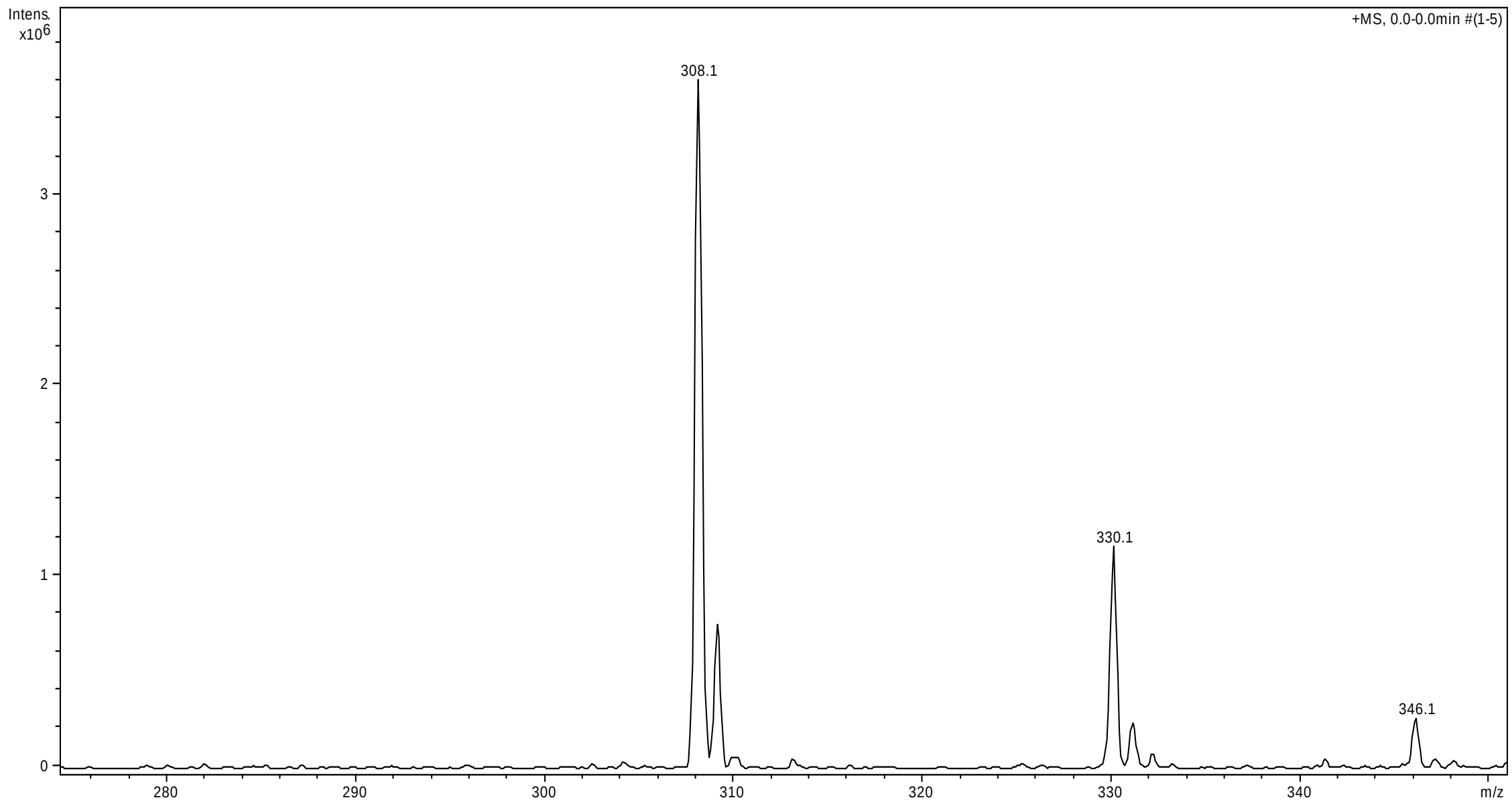

Figure S61. ESI-MS spectrum of **Compound 17**.

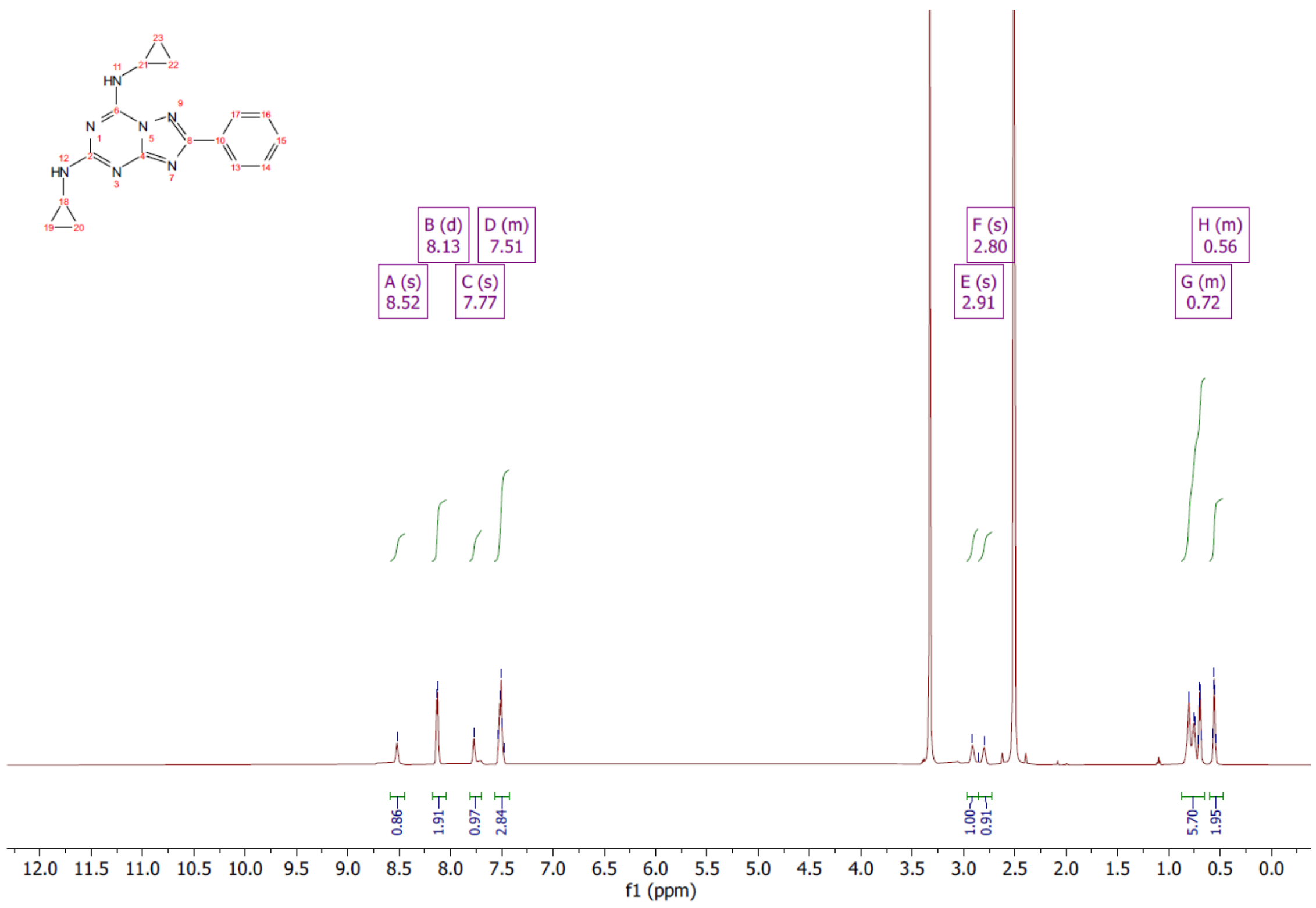

Figure S62. ^{1}H NMR spectrum of **Compound 17** (DMSO-d_6).

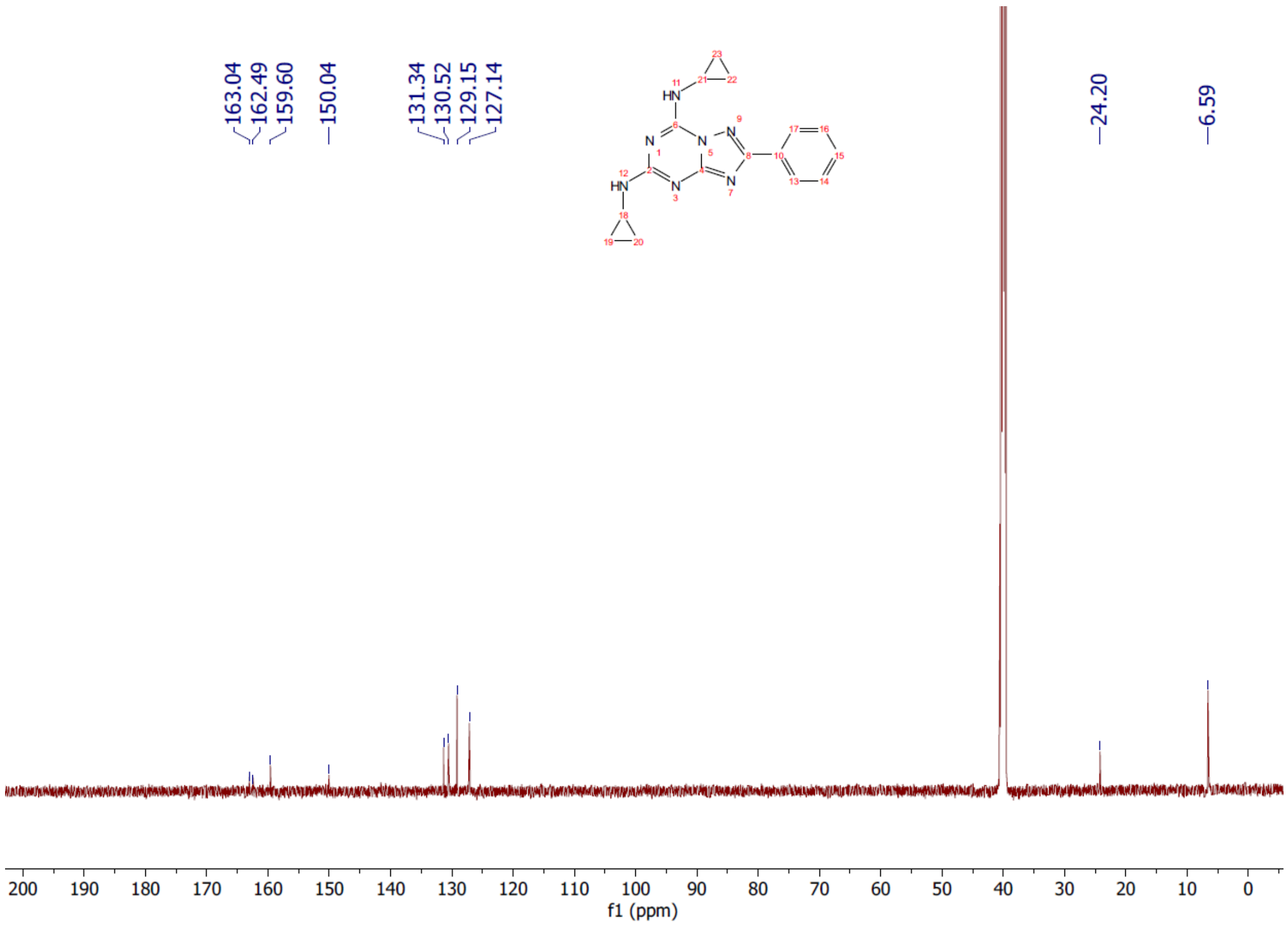

Figure S63. ^{13}C NMR spectrum of **Compound 17** (DMSO-d_6).

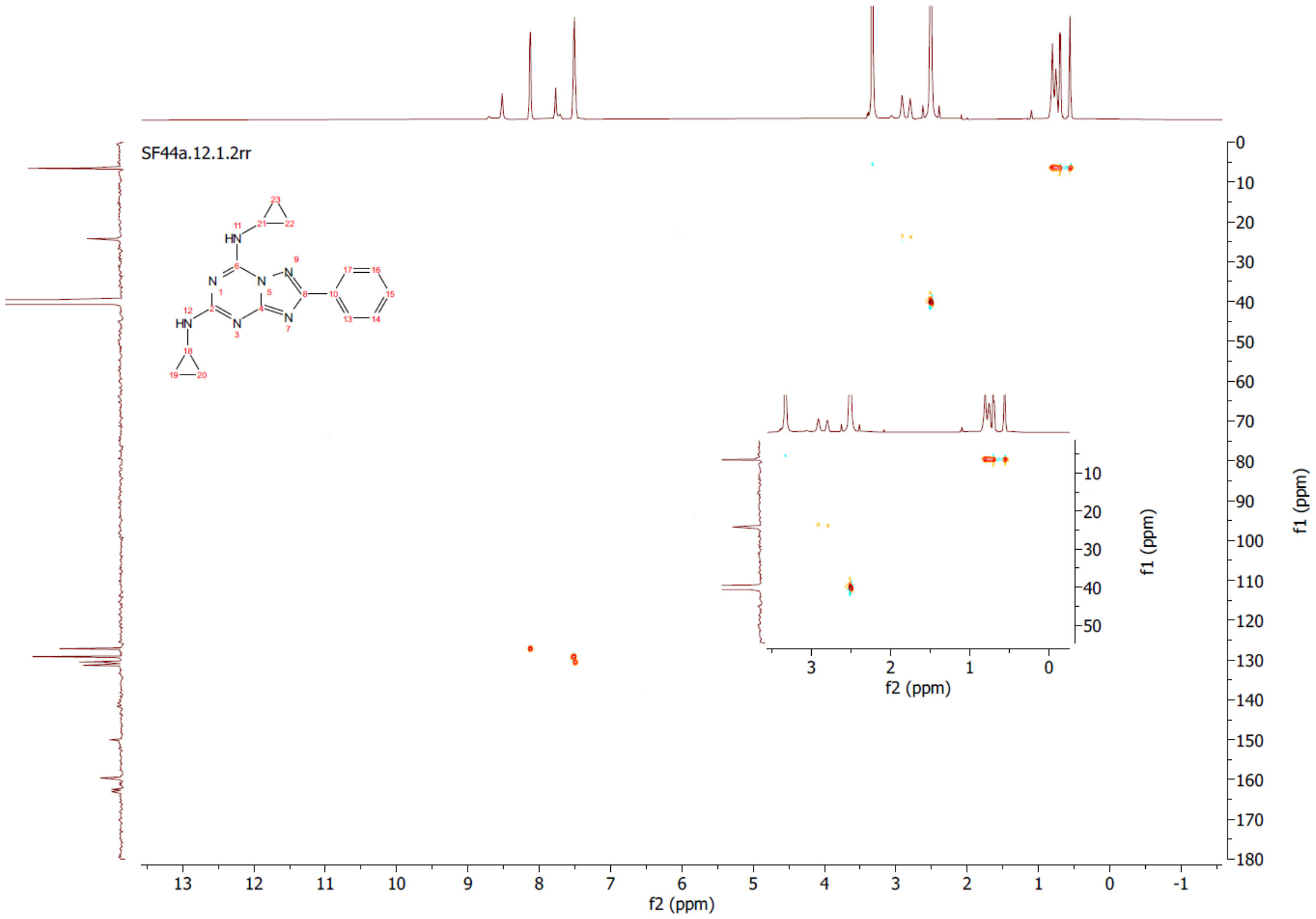

Figure S64. HSQC ^1H-^{13}C NMR spectrum of **Compound 17** (DMSO-d_6).

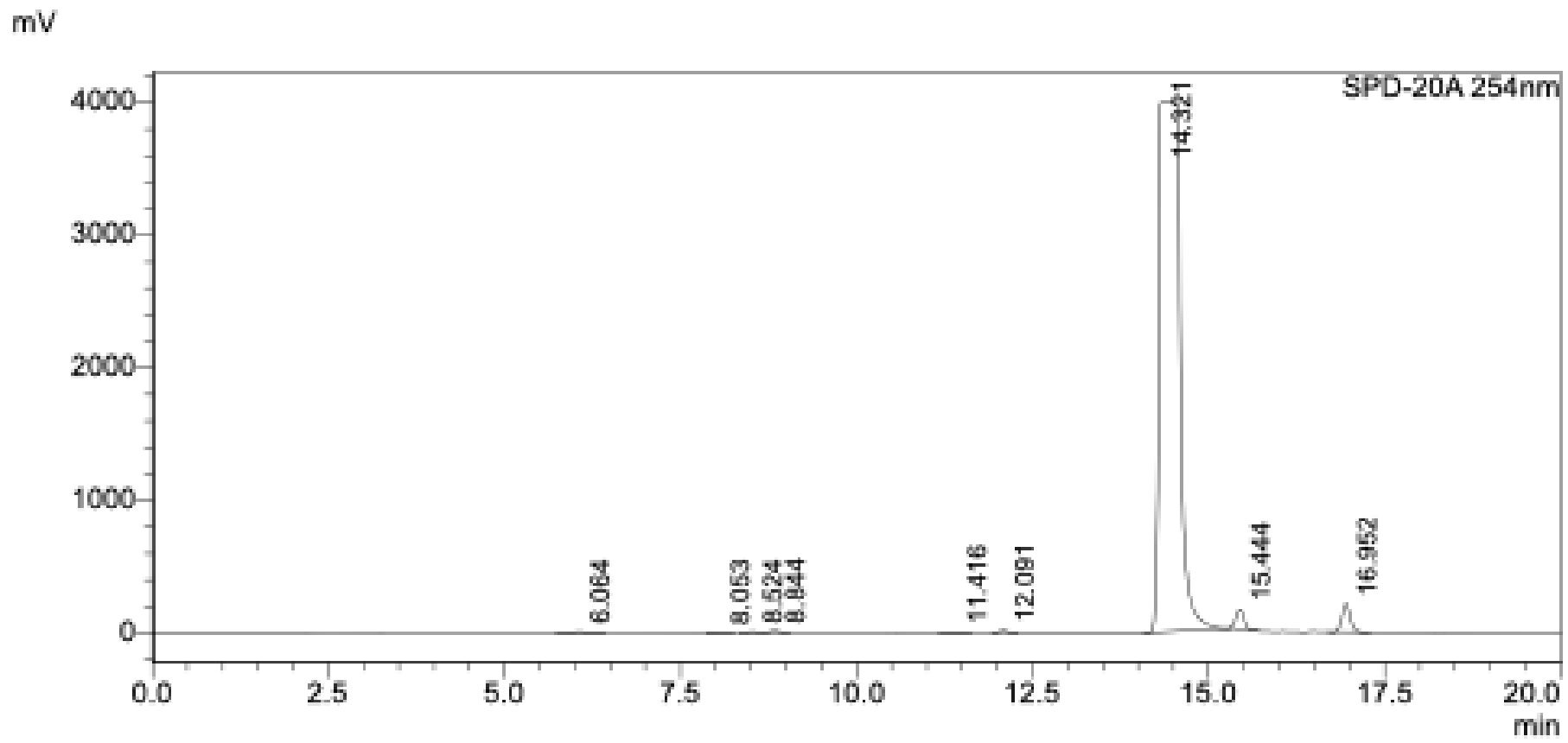

<Peak Table>

SPD-20A 254nm

Peak#	Ret. Time	Area	Height	Area%
1	6.064	123039	9902	0.145
2	8.053	42134	3977	0.050
3	8.524	69471	7508	0.082
4	8.844	105731	12458	0.124
5	11.416	59256	5819	0.070
6	12.091	142233	16045	0.167
7	14.321	81527875	3991886	95.939
8	15.444	1161443	148668	1.367
9	16.952	1747291	213619	2.056
Total		84978473	4409882	100.000

Figure S65. HPLC chromatogram of **Compound 17**.

N^5-cyclohexyl-N^7-cyclopropyl-2-phenyl-[1,2,4]triazolo[1,5-*a*][1,3,5]triazine-5,7-diamine **18**

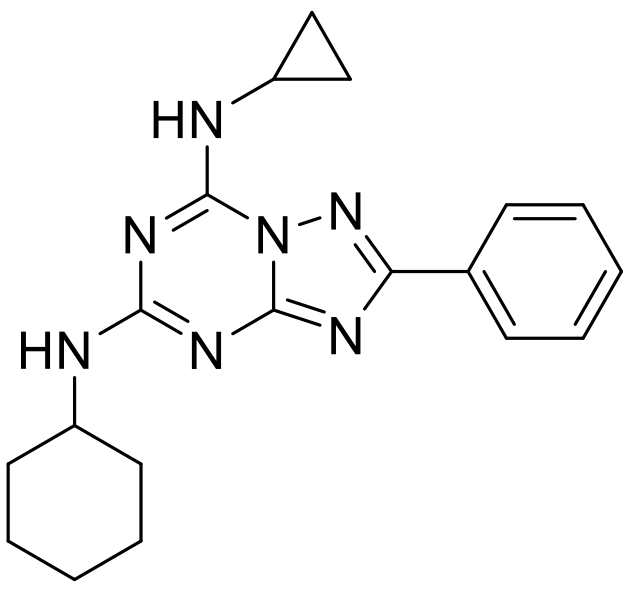

Chemical Formula: $C_{19}H_{23}N_7$
Molecular Weight: 349,43

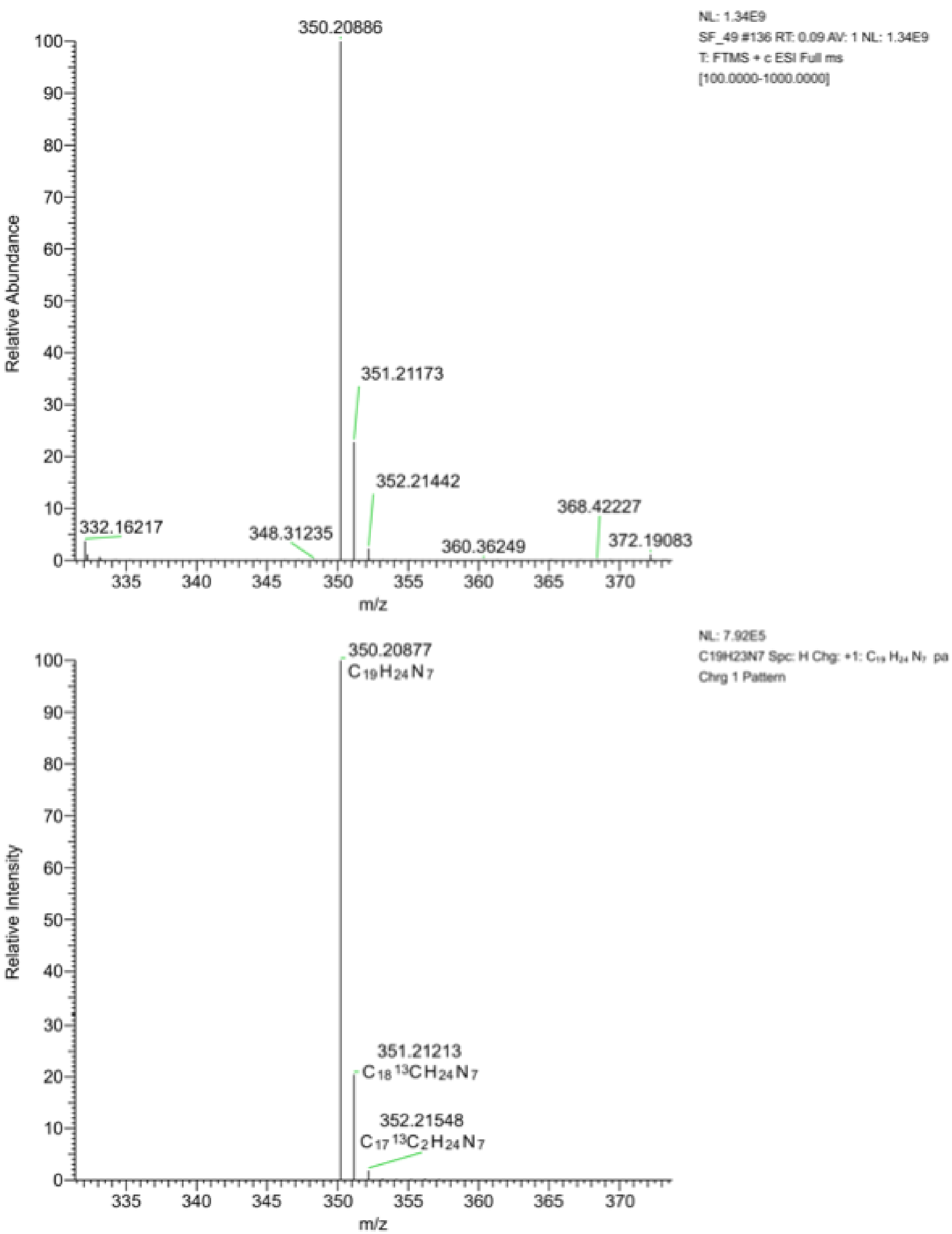

Figure S66. HRMS (ESI) spectrum of **Compound 18**.

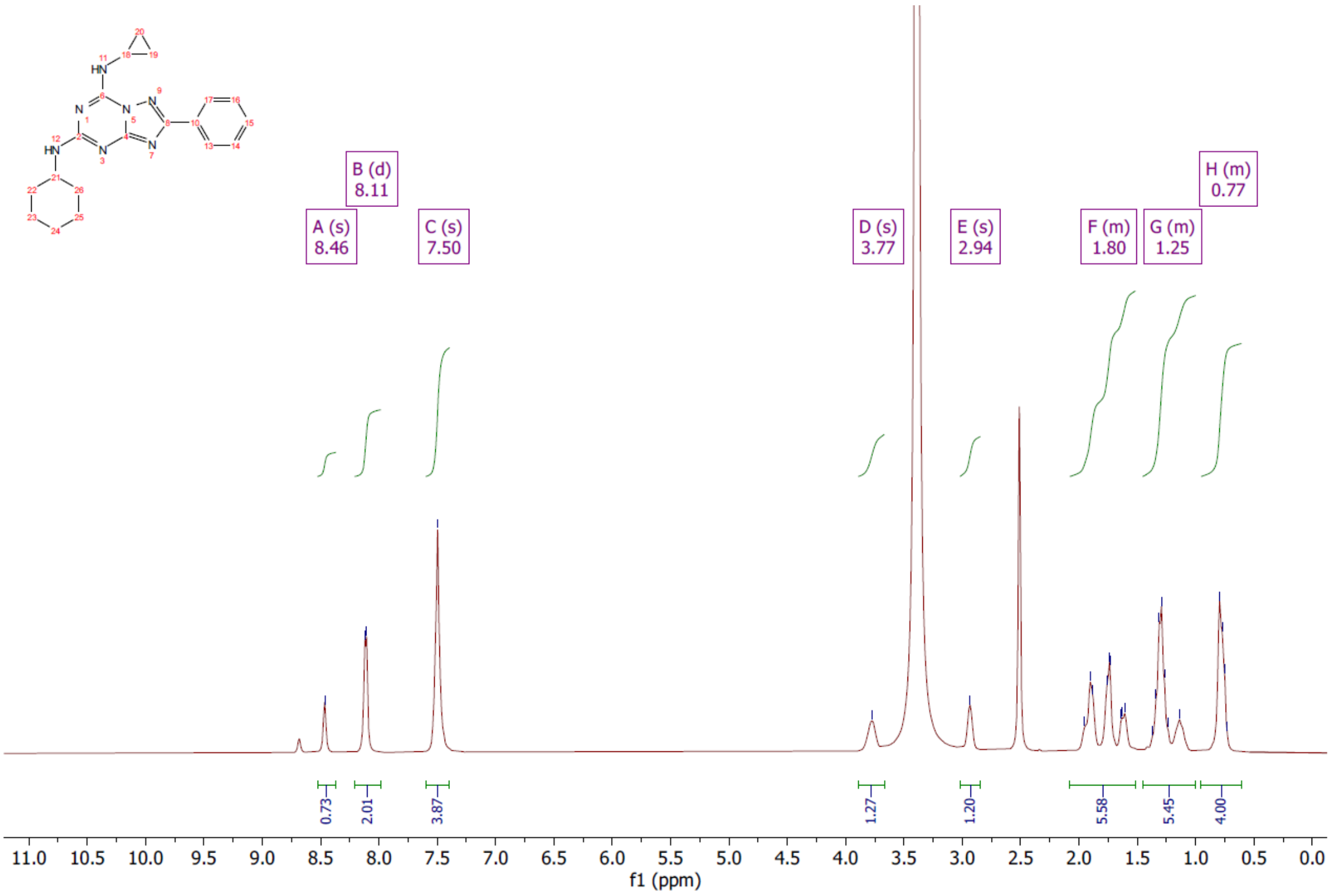

Figure S67. ^{1}H NMR spectrum of **Compound 18** (DMSO-d_6).

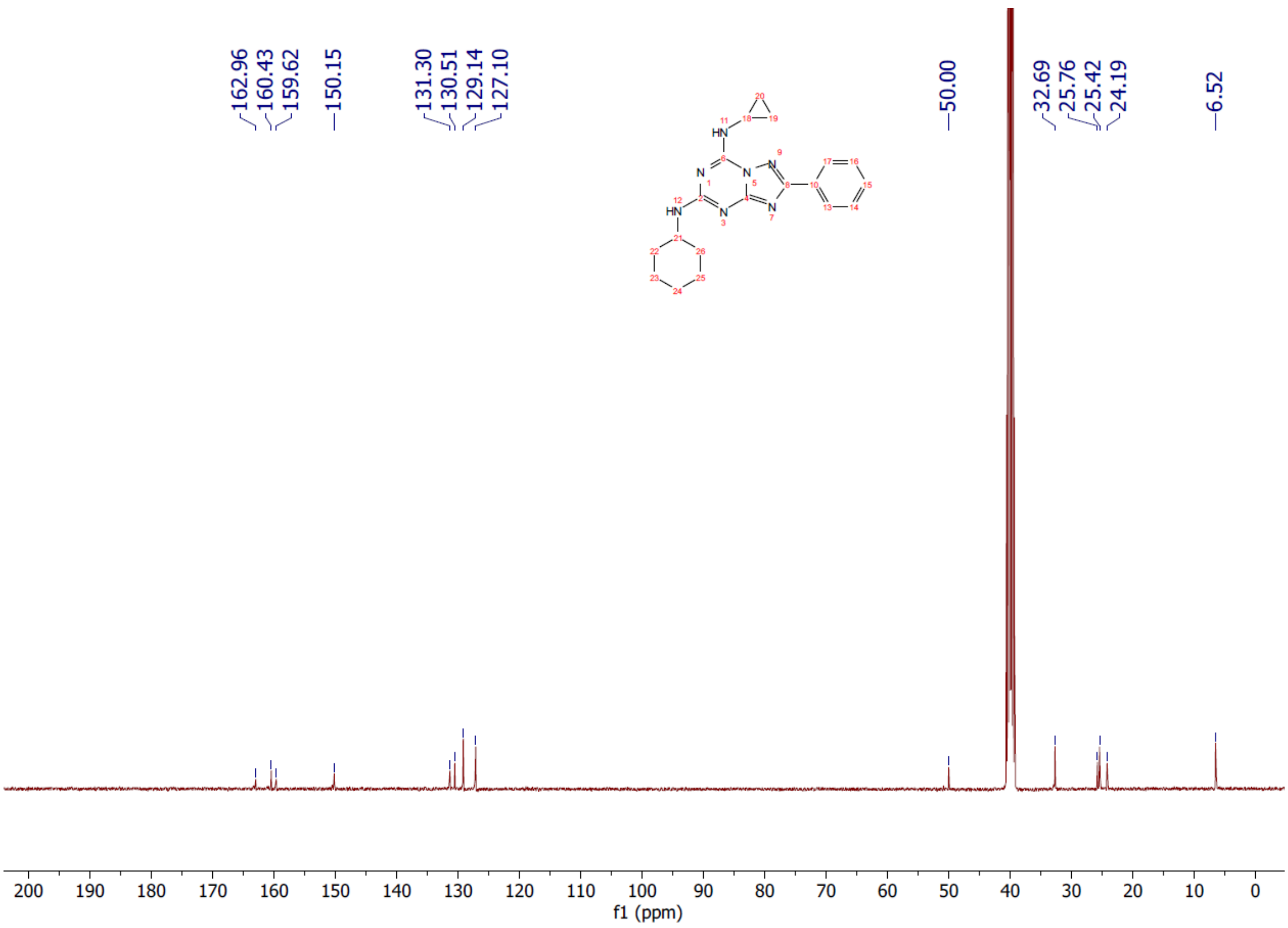

Figure S68. ^{13}C NMR spectrum of **Compound 18** (DMSO-d_6).

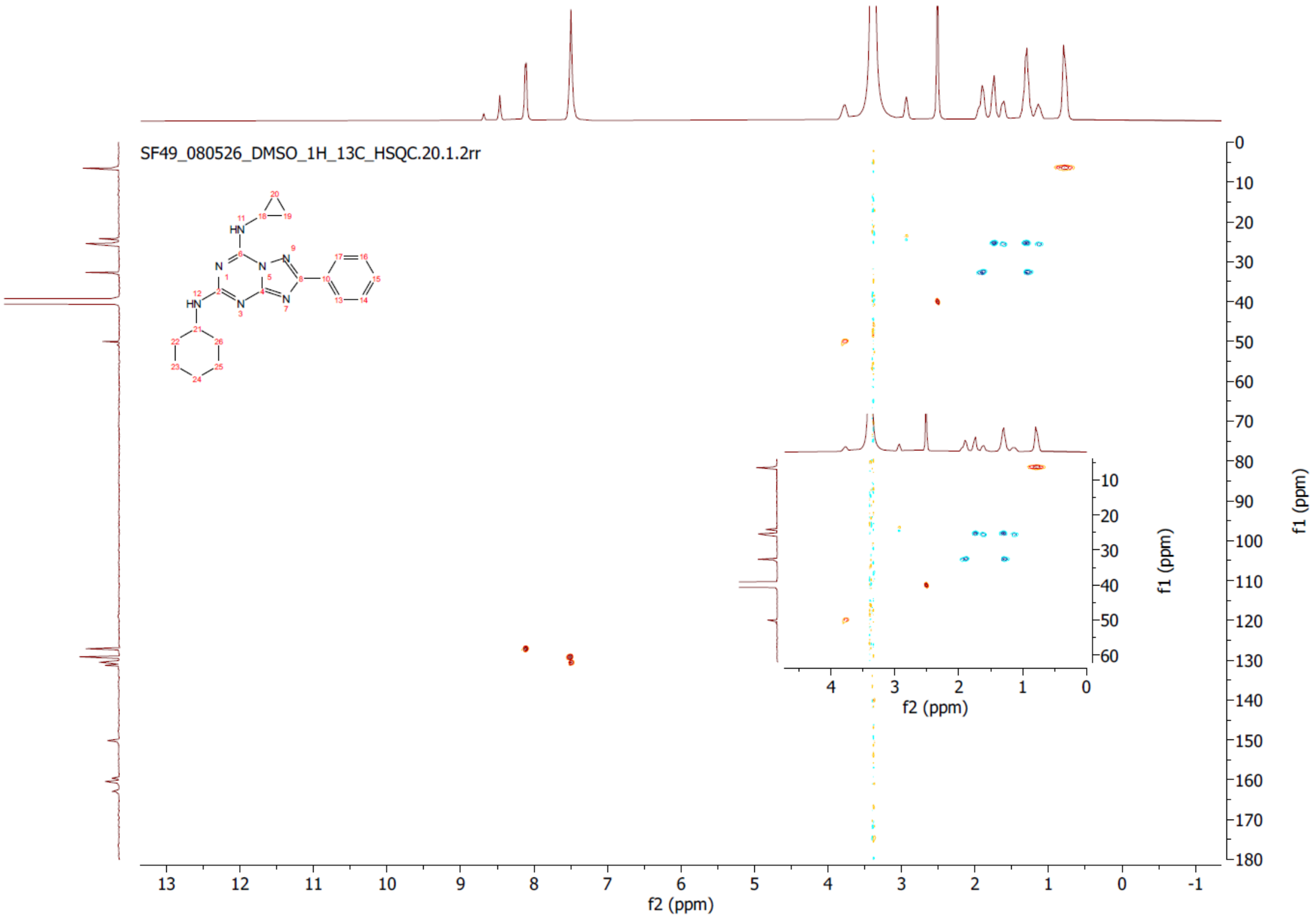

Figure S69. HSQC ^{1}H-^{13}C NMR spectrum of **Compound 18** ($CDCl_3$).

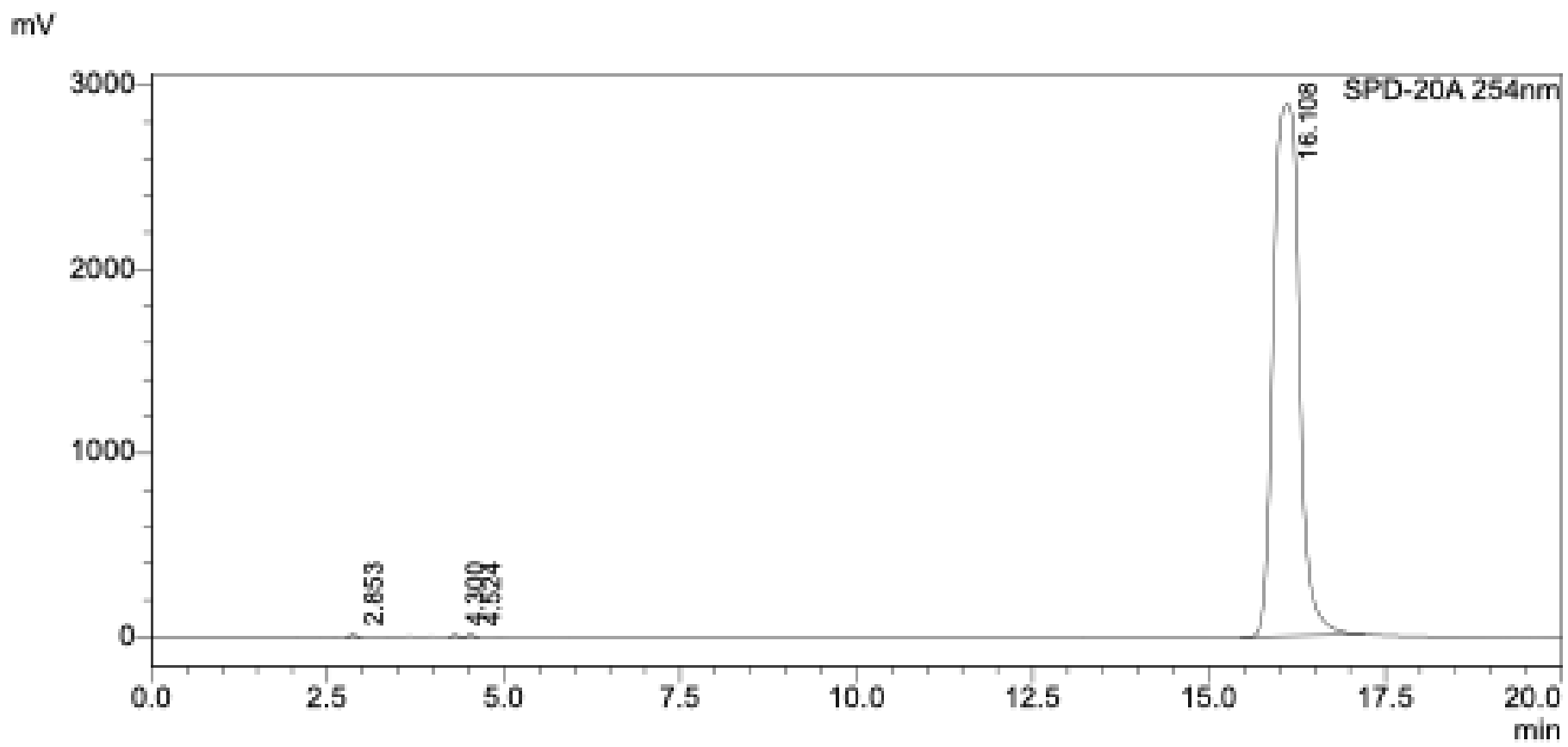

<Peak Table>

SPD-20A 254nm

Peak#	Ret. Time	Area	Height	Area%
1	2.853	57864	12494	0.078
2	4.300	43123	8971	0.058
3	4.524	55733	11176	0.075
4	16.108	74294529	2892854	99.790
Total		74451248	2925494	100.000

Figure S70. HPLC Chromatogram of **Compound 18**.

N[5]-benzyl-N[7]-cyclopropyl -2-phenyl-[1,2,4]triazolo[1,5-*a*][1,3,5]triazine-5,7-diamine **19**

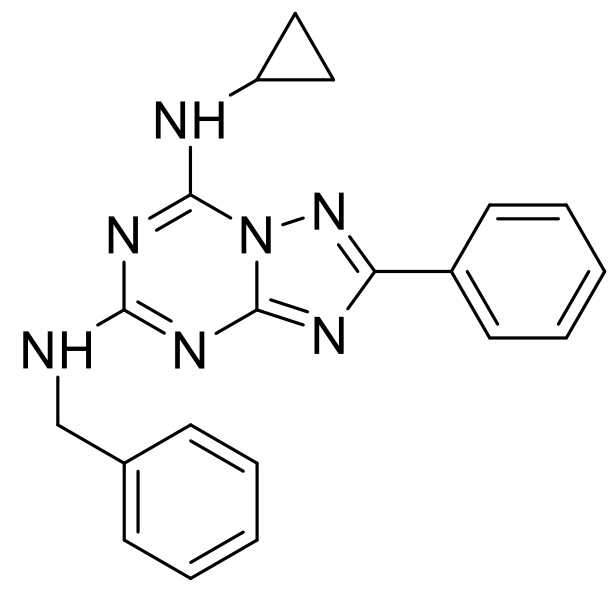

Chemical Formula: $C_{20}H_{19}N_7$
Molecular Weight: 357,41

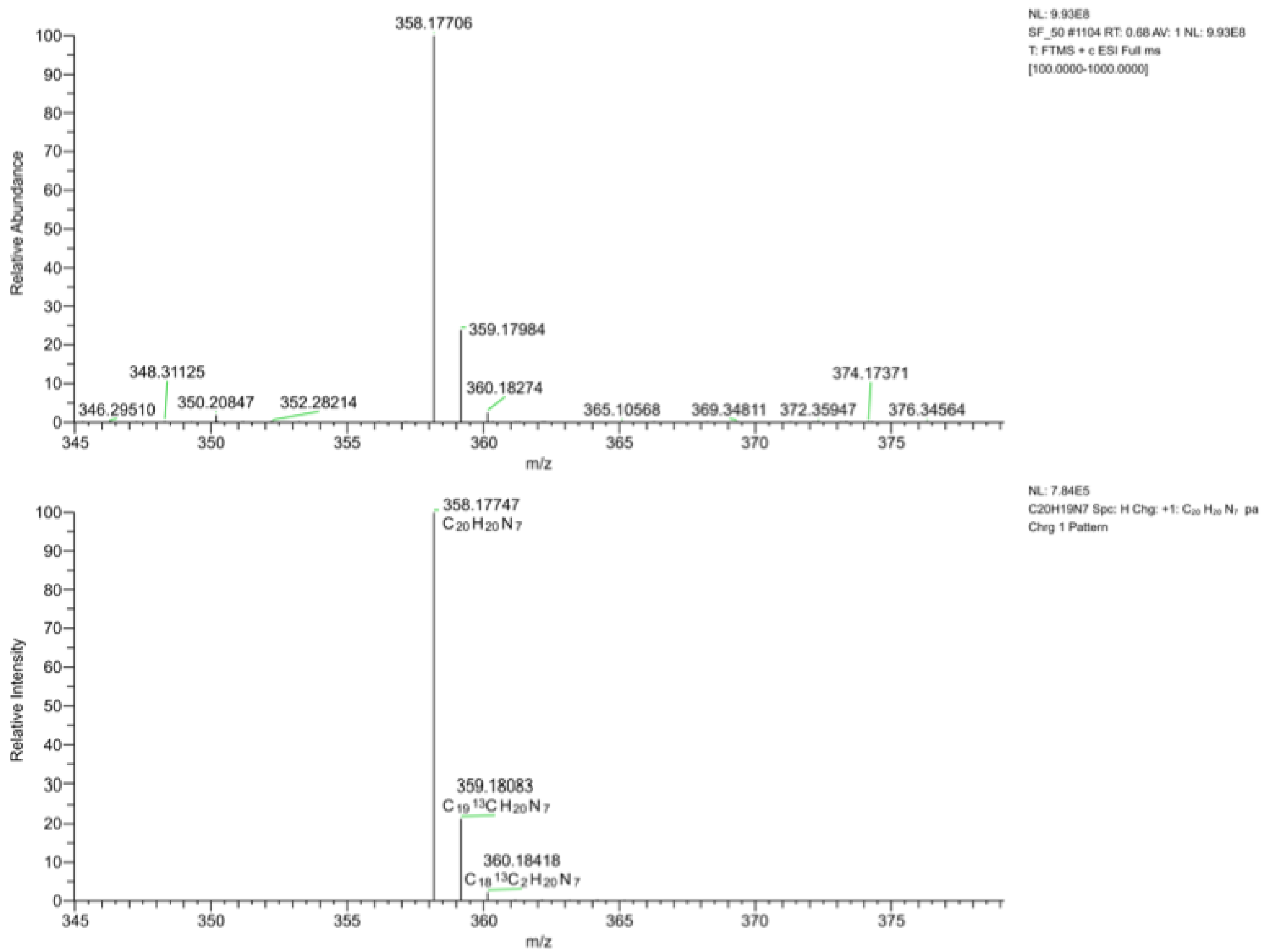

Figure S71. HRMS (ESI) of **Compound 19**.

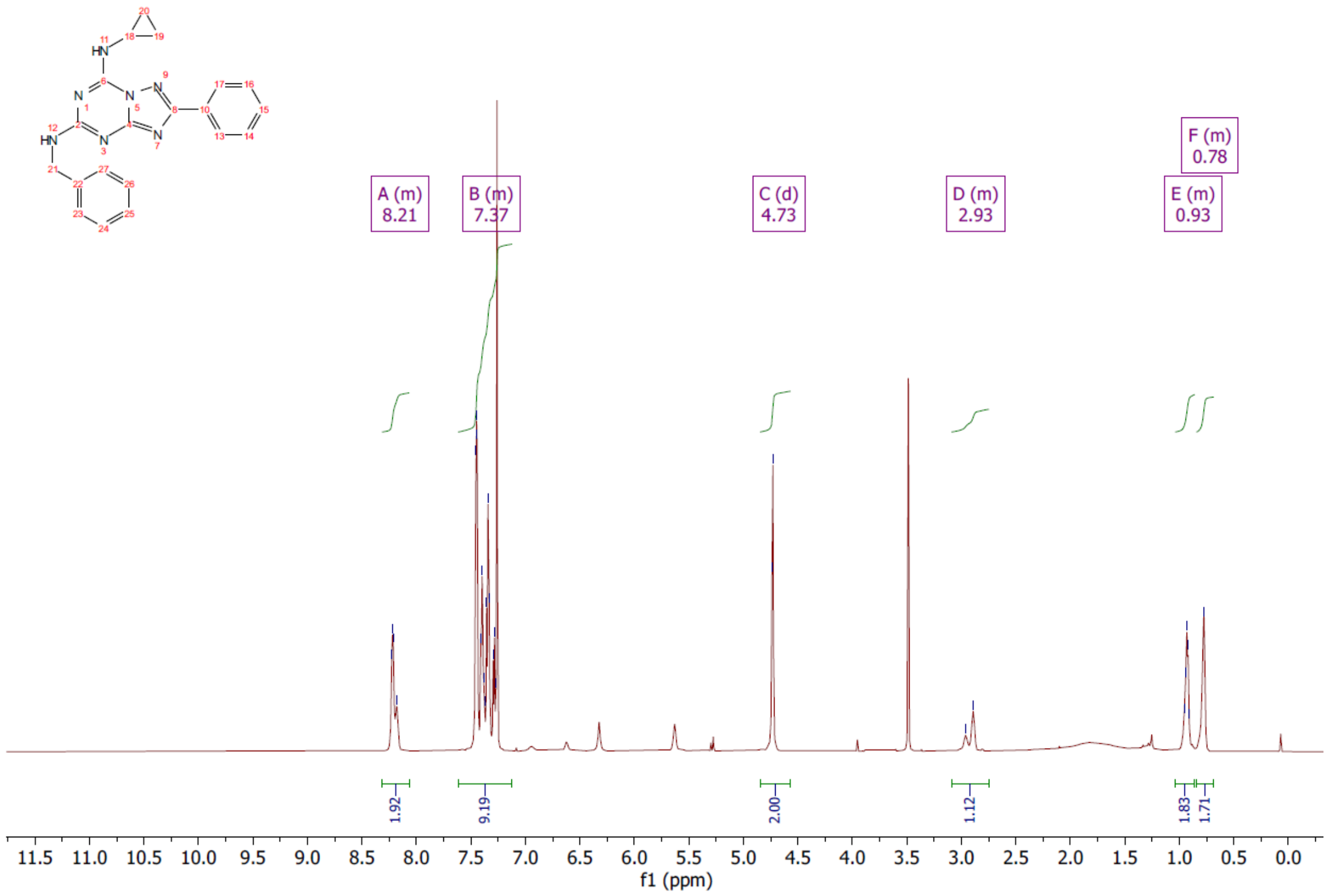

Figure S72. ^{1}H NMR spectrum of **Compound 19** ($CDCl_3$)

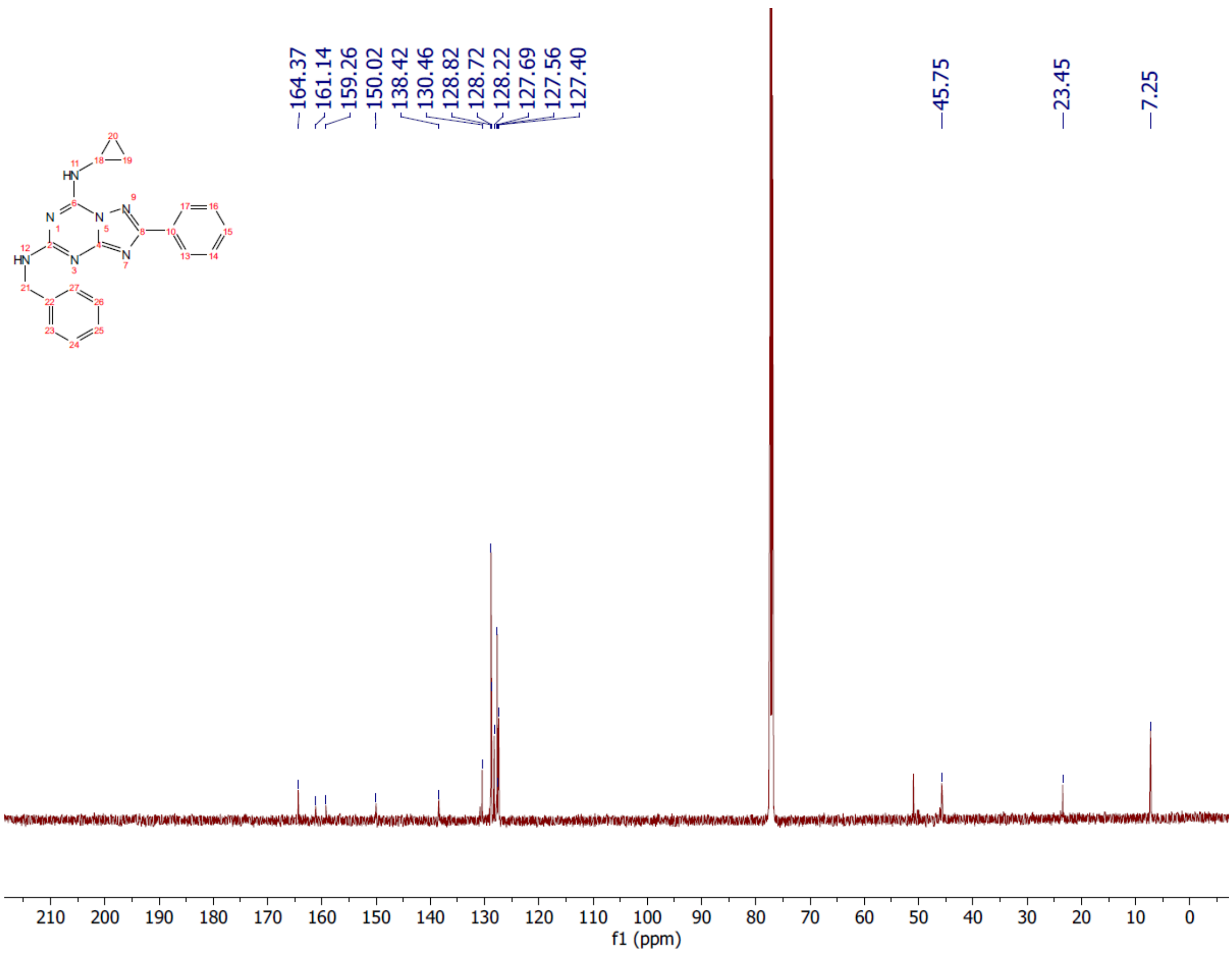

Figure S73. ^{13}C NMR spectrum of **Compound 19** ($CDCl_3$)

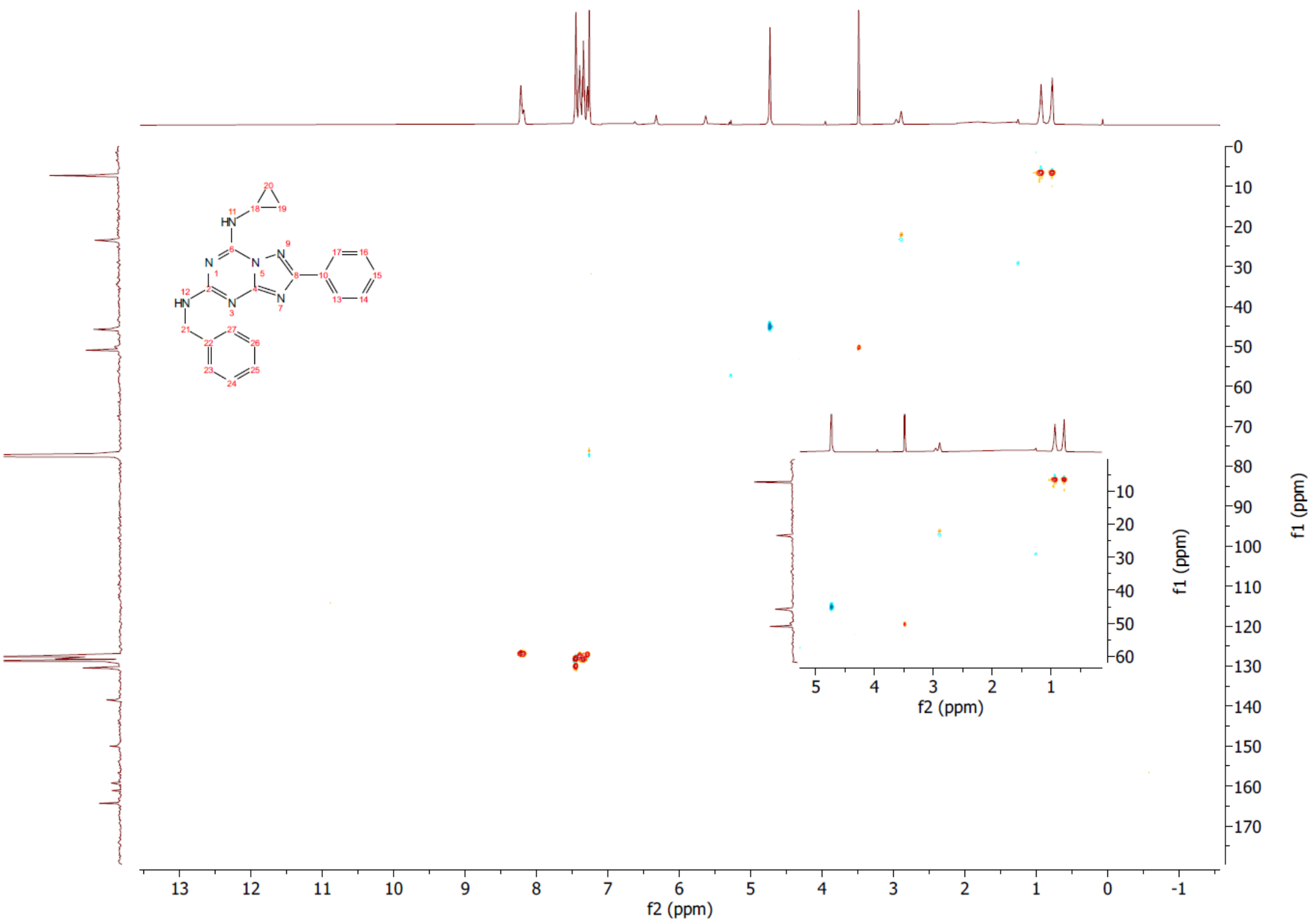

Figure S74. HSQC ^1H-^{13}C NMR spectrum of **Compound 19** ($CDCl_3$).

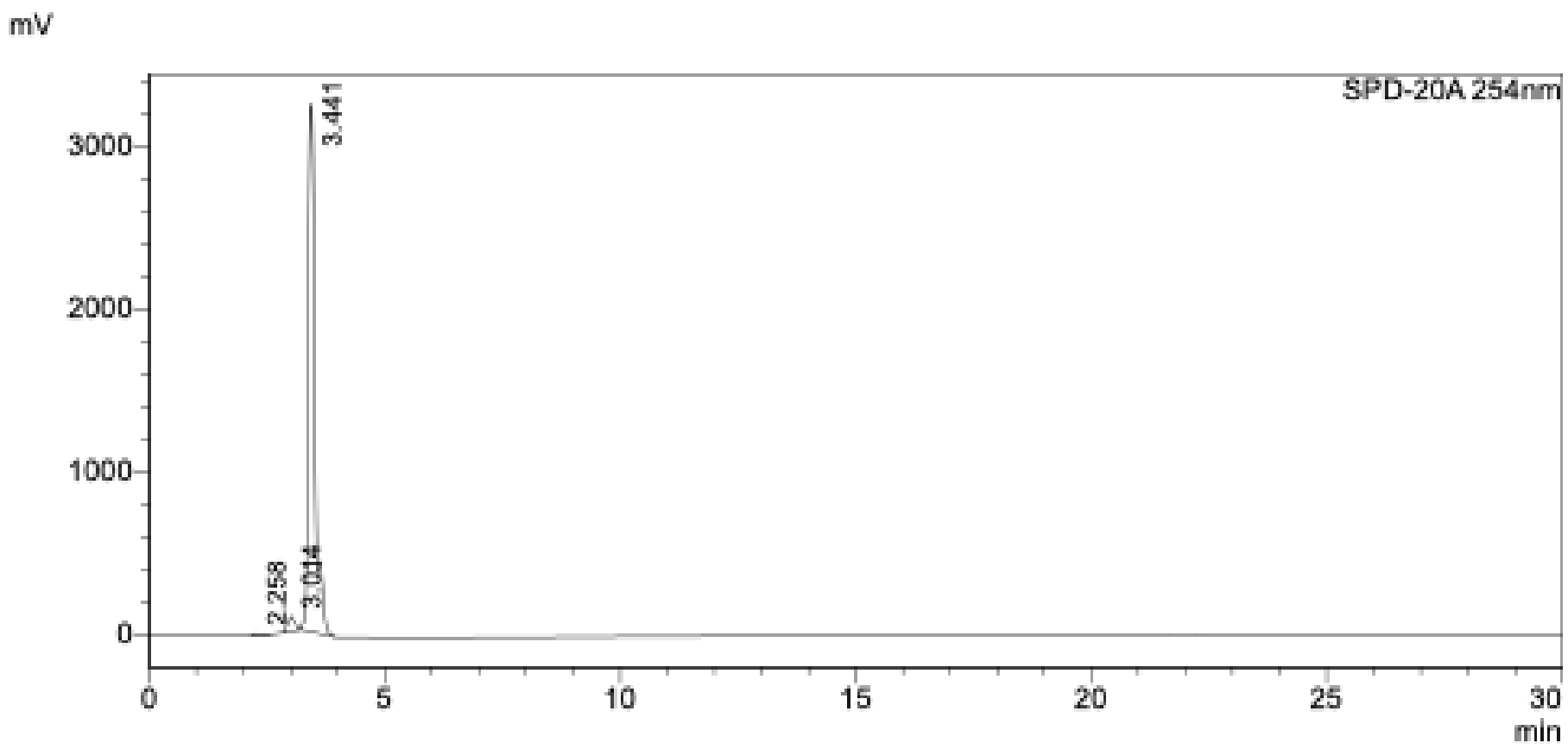

<Peak Table>

SPD-20A 254nm

Peak#	Ret. Time	Area	Height	Area%
1	2.258	75307	10097	0.229
2	3.014	929868	90865	2.823
3	3.441	31938260	3240816	96.949
Total		32943435	3341779	100.000

Figure S75. HPLC Chromatogram of **Compound 19.**

N^7-cyclohexyl-N^5-methyl-2-phenyl-[1,2,4]triazolo[1,5-*a*][1,3,5]triazine-5,7-diamine **20**

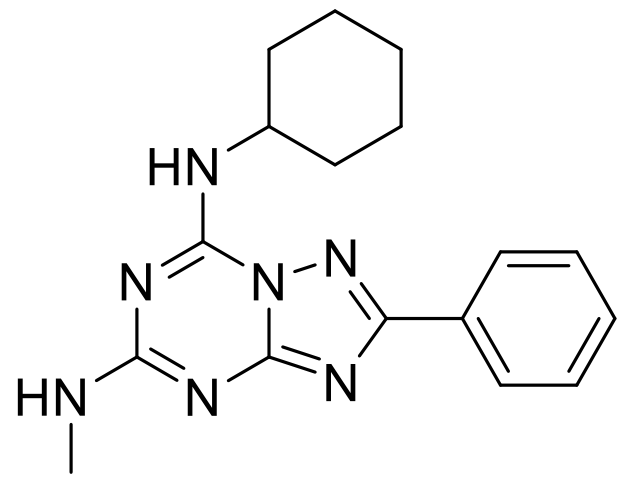

Chemical Formula: $C_{17}H_{21}N_7$
Molecular Weight: 323,40

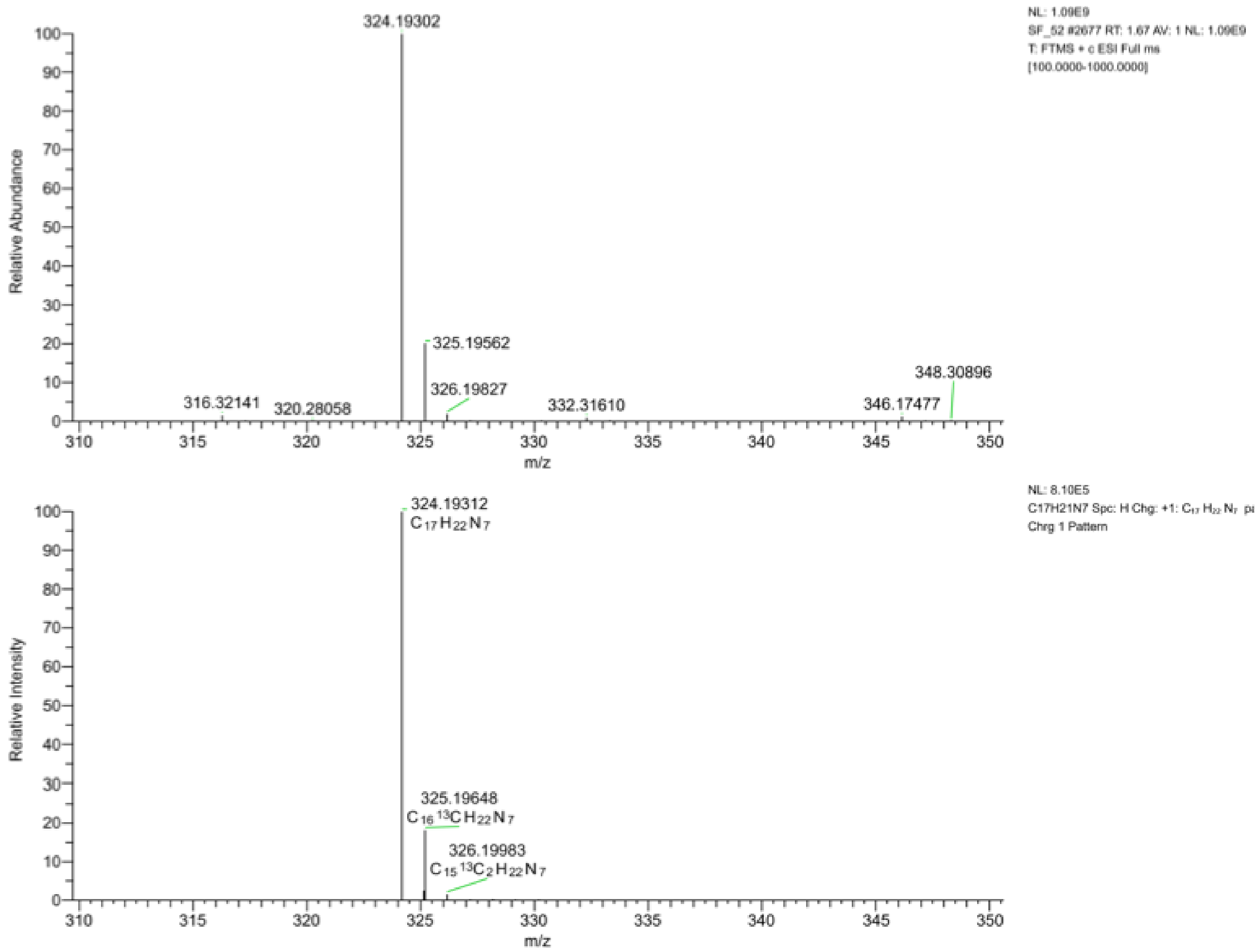

Figure S76. HRMS (ESI) spectrum of **Compound 20**.

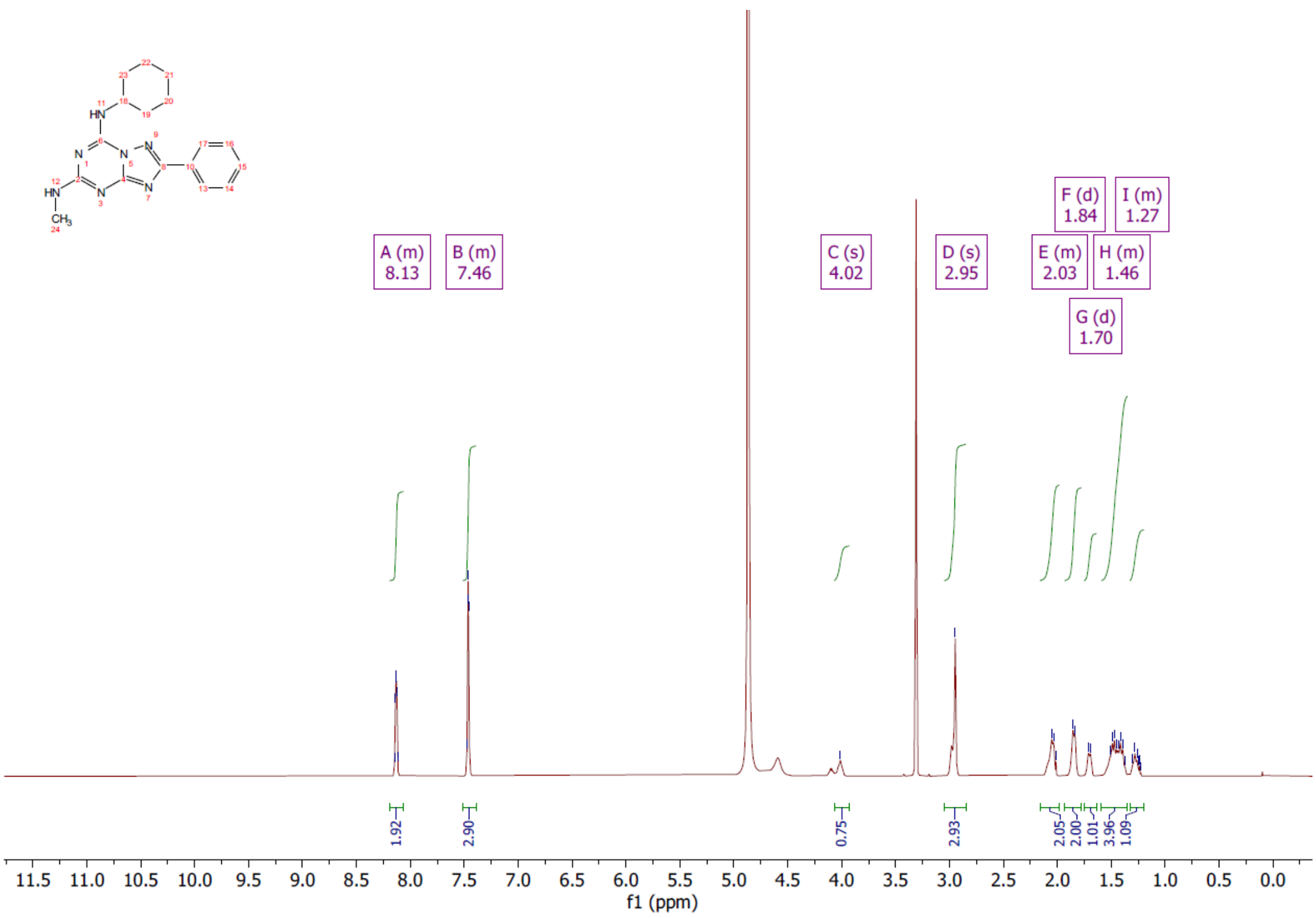

Figure S77. ^{1}H NMR spectrum of **Compound 20** (CD_3OD).

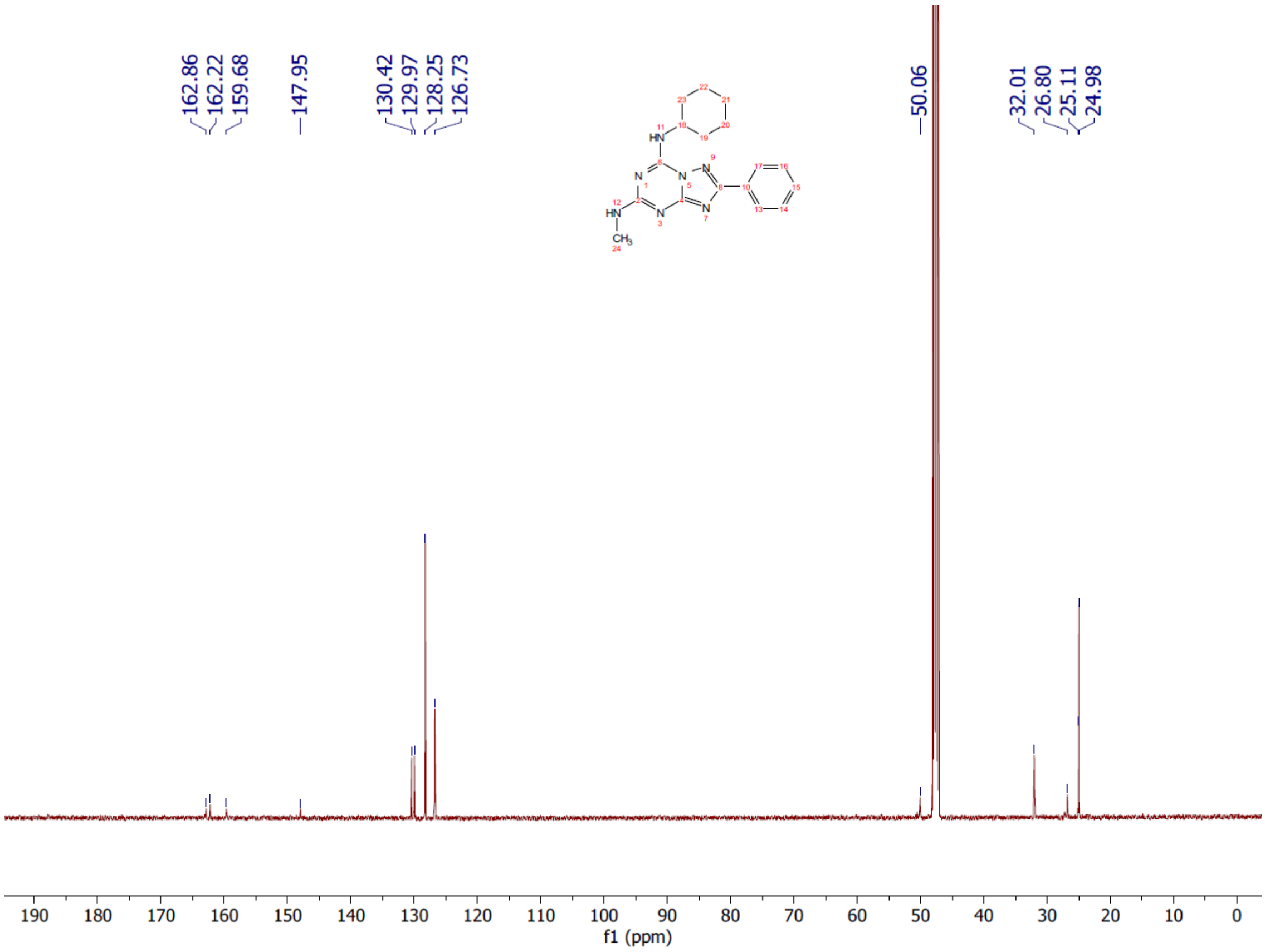

Figure S78. ^{13}C NMR spectrum of **Compound 20** (CD_3OD).

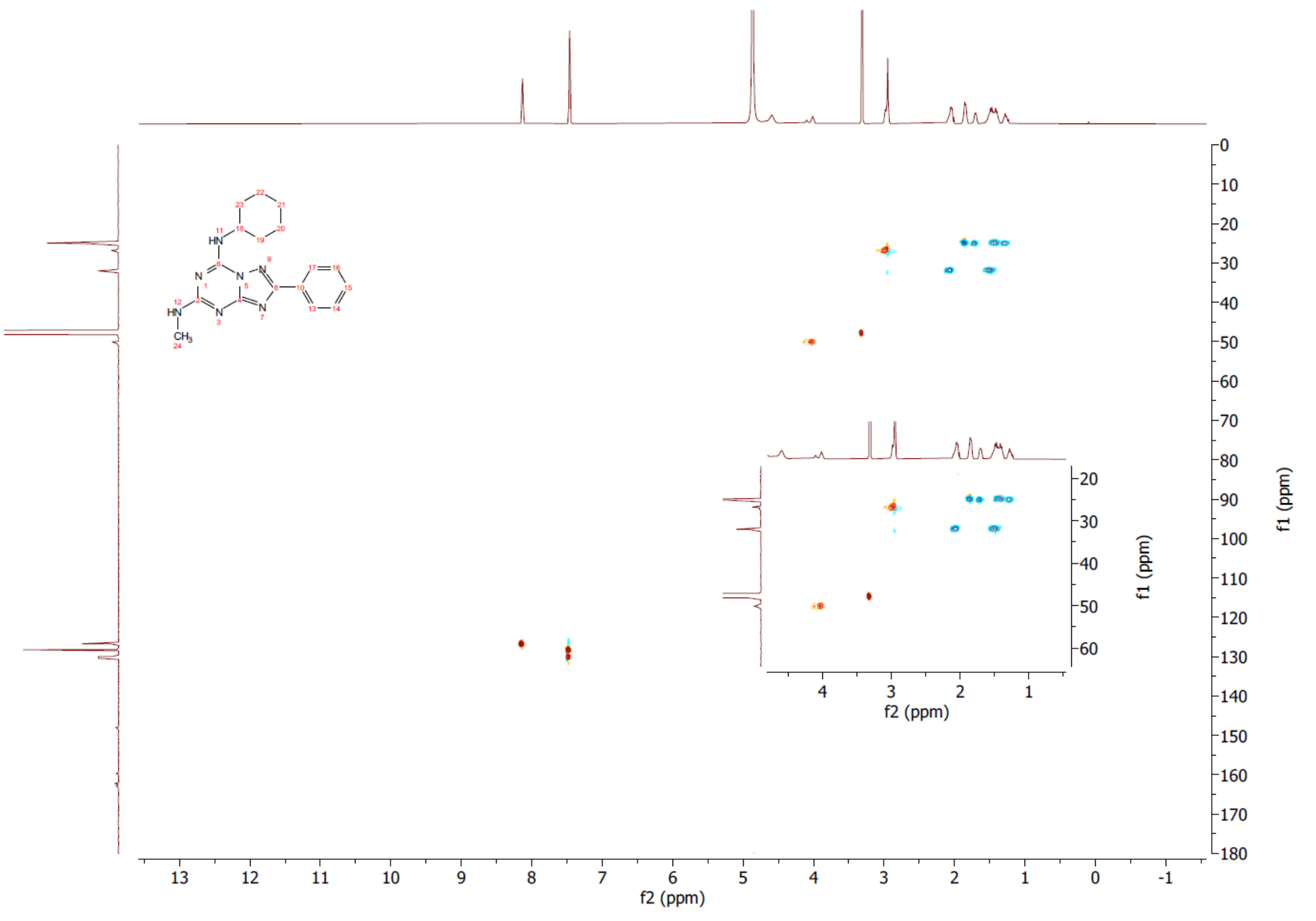

Figure S79. HSQC ^{1}H-^{13}C NMR spectrum of **Compound 20** (CD_3OD).

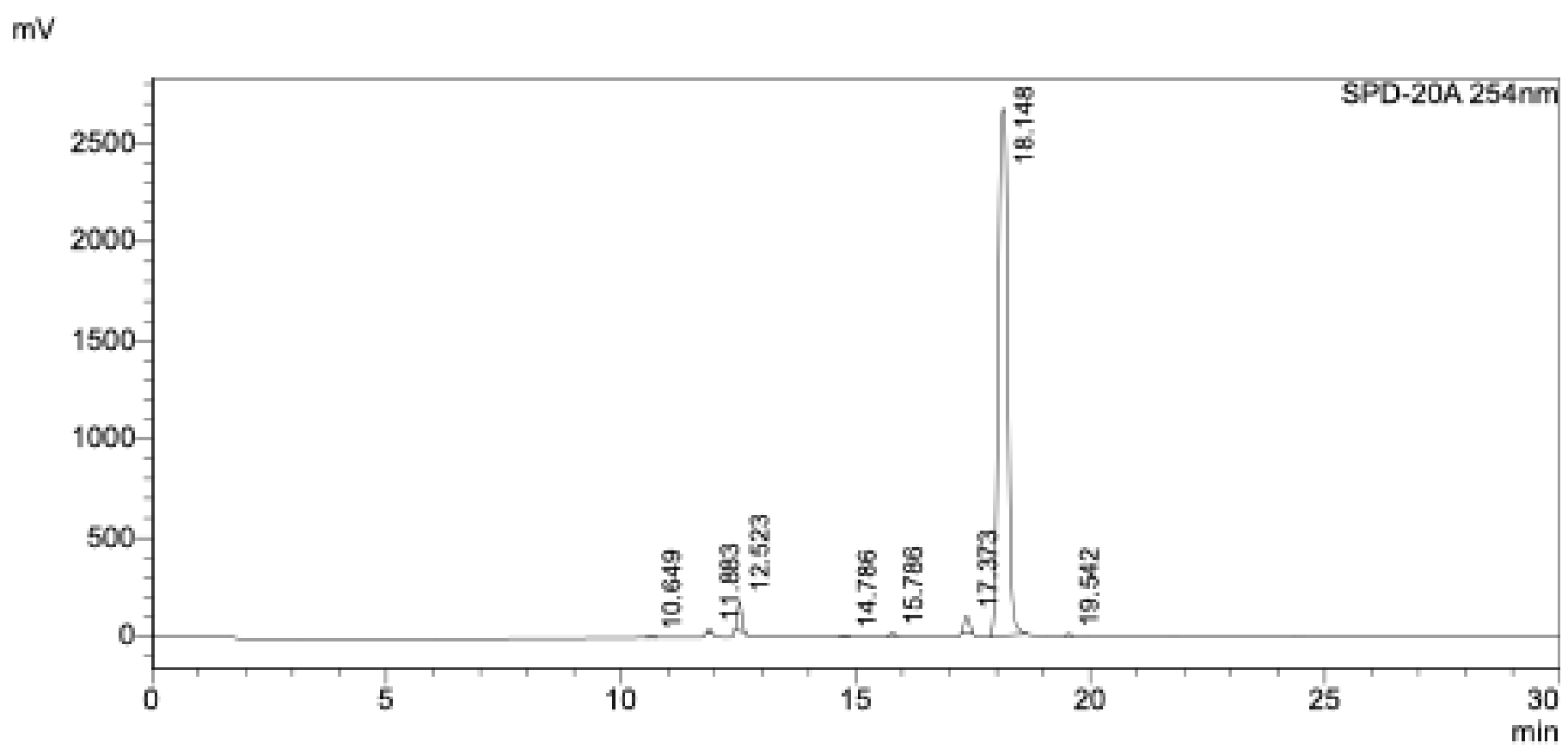

<Peak Table>

SPD-20A 254nm

Peak#	Ret. Time	Area	Height	Area%
1	10.649	8839	1353	0.022
2	11.883	113587	23745	0.287
3	12.523	1032508	150591	2.606
4	14.786	15752	2539	0.040
5	15.786	63975	11168	0.161
6	17.373	665183	83530	1.679
7	18.148	37691907	2670265	95.144
8	19.542	23732	4712	0.060
Total		39615483	2947903	100.000

Figure S80. HPLC Chromatogram of **Compound 20.**

N[7]-cyclohexyl-N[5]-cyclopropyl-2-phenyl-[1,2,4]triazolo[1,5-*a*][1,3,5]triazine-5,7-diamine **21**

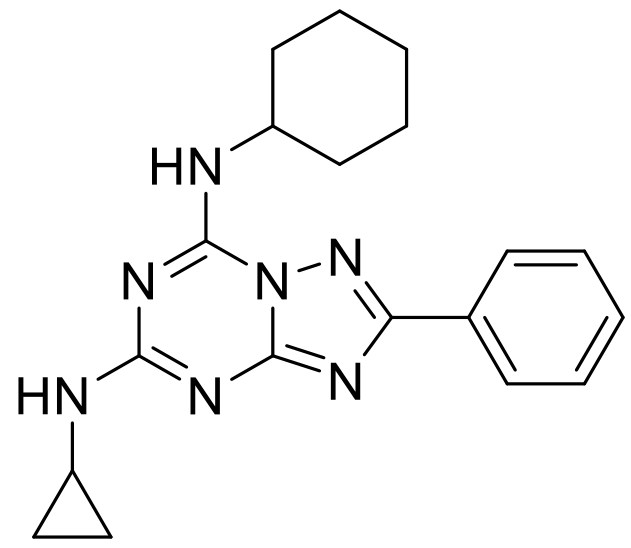

Chemical Formula: $C_{19}H_{23}N_7$
Molecular Weight: 349,43

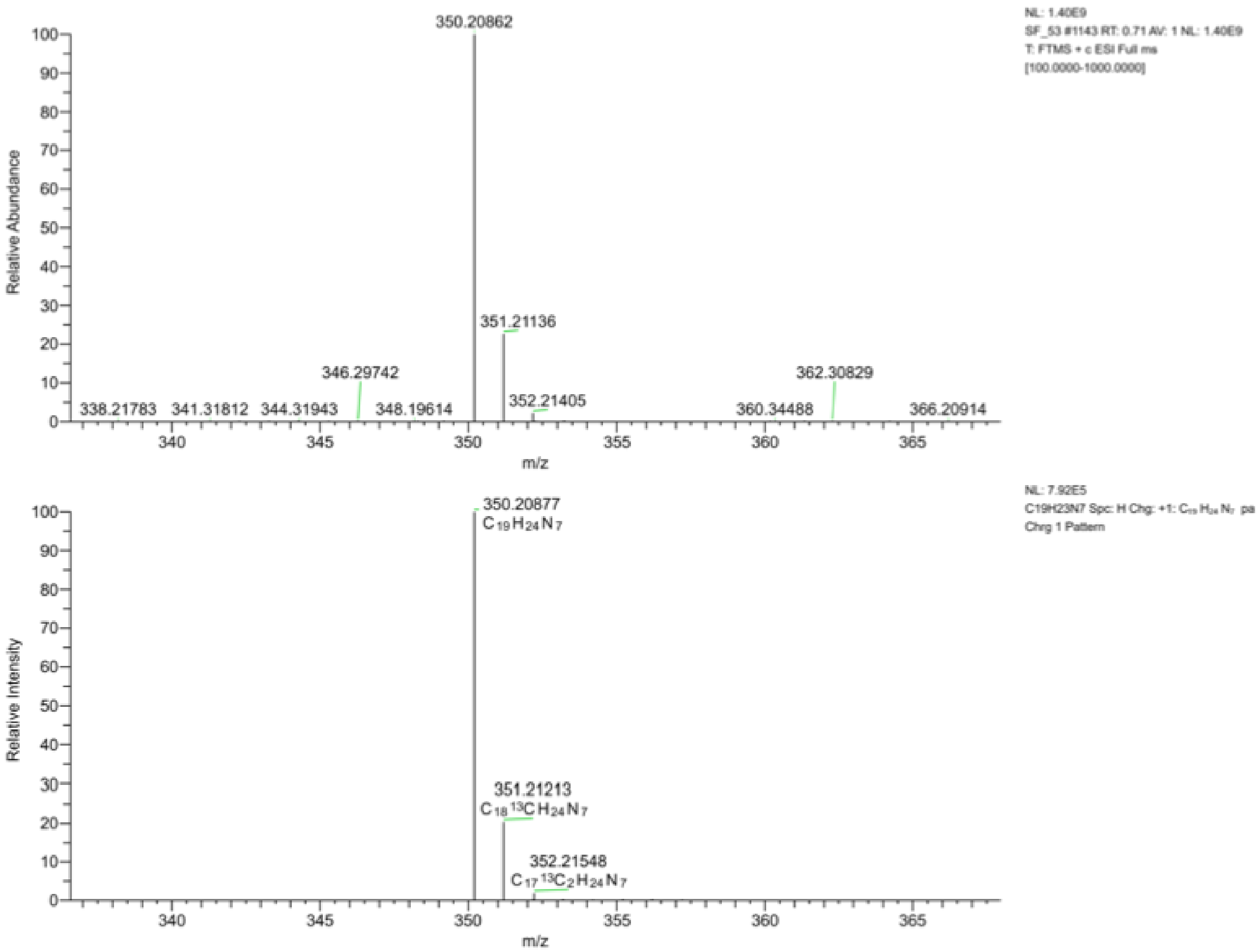

Figure S81. HRMS (ESI) spectrum of **Compound 21**.

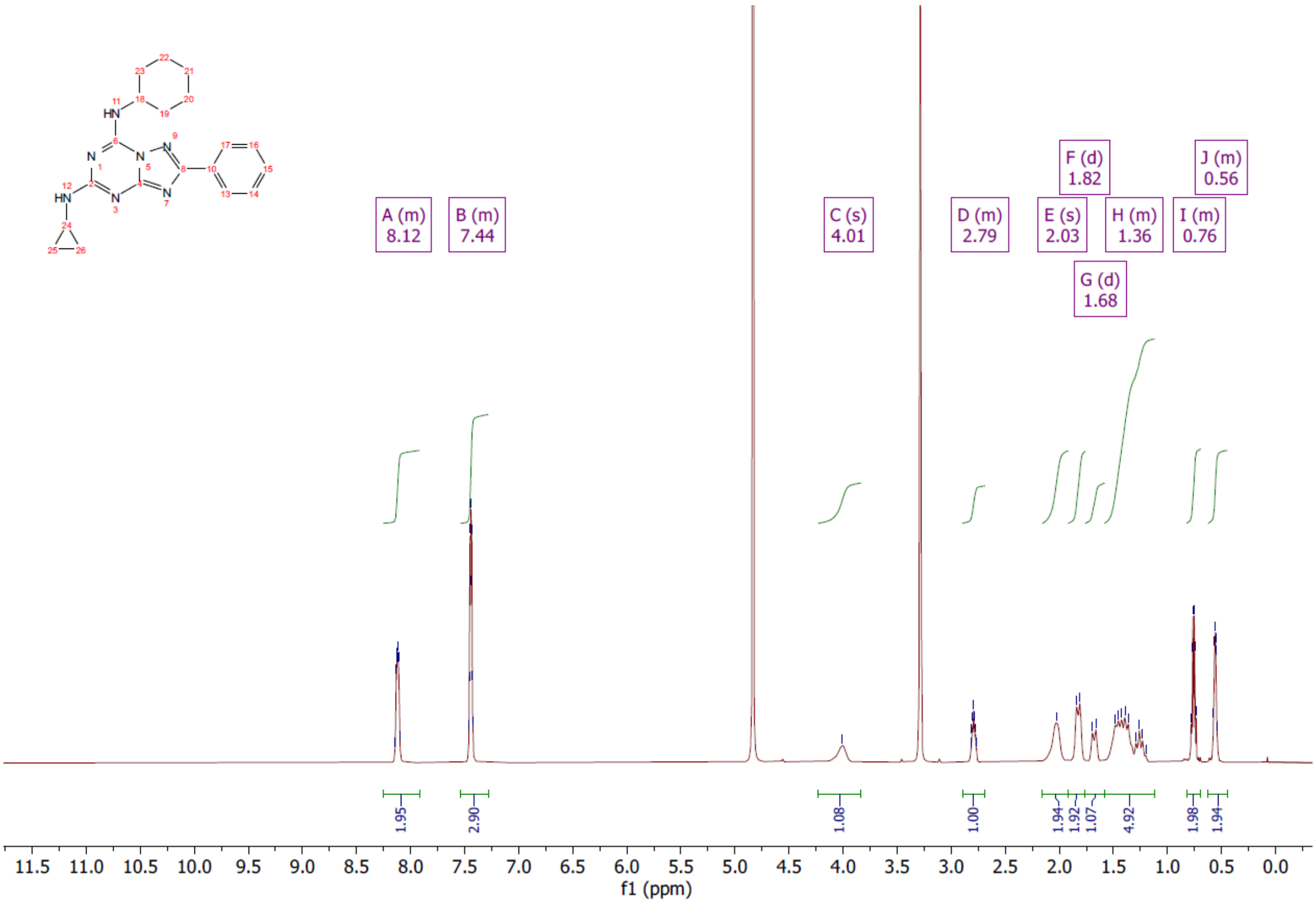

Figure S82. ^{1}H NMR spectrum of **Compound 21** (CD_3OD).

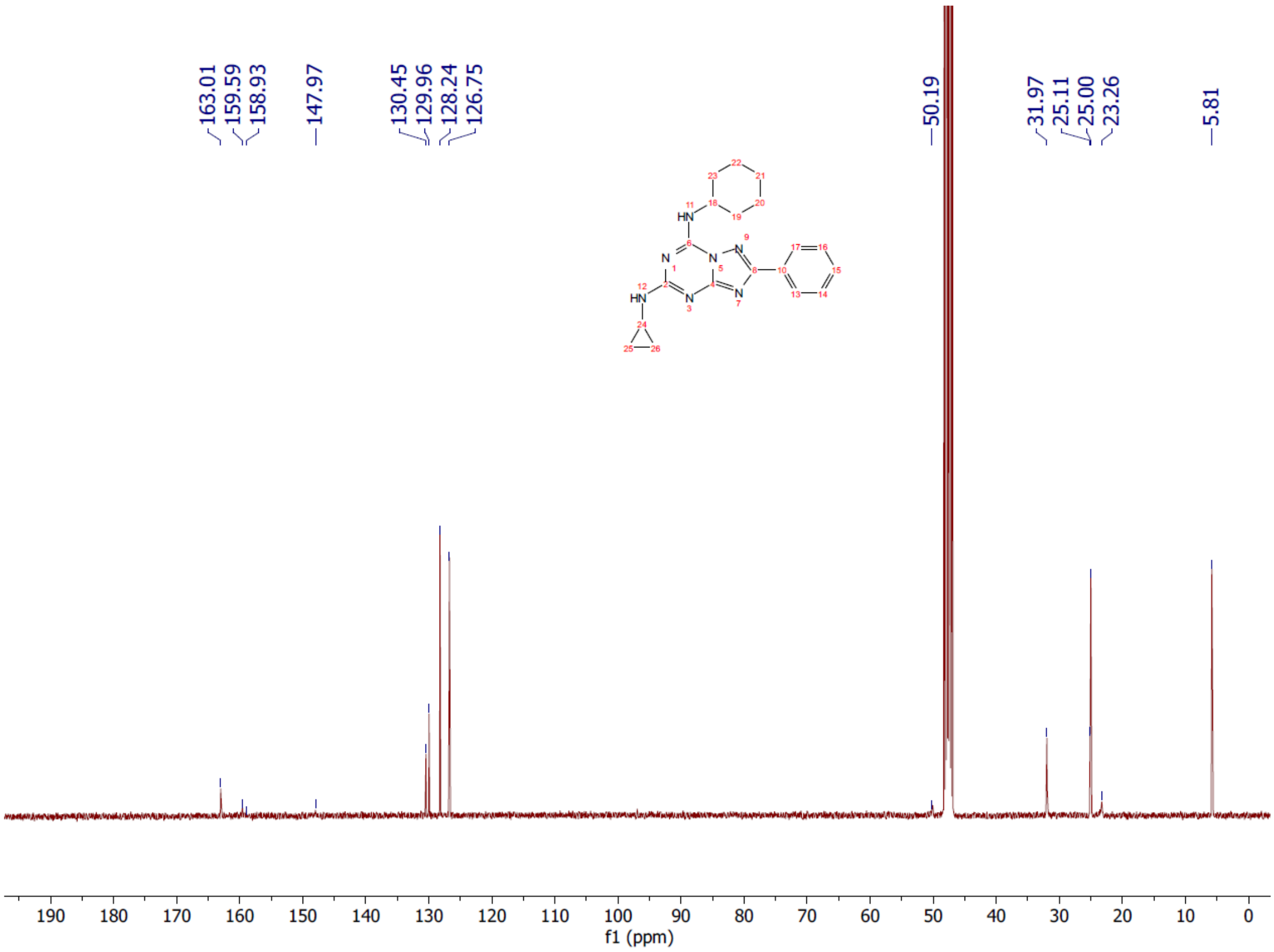

Figure S83. ^{13}C NMR spectrum of **Compound 21** (CD_3OD).

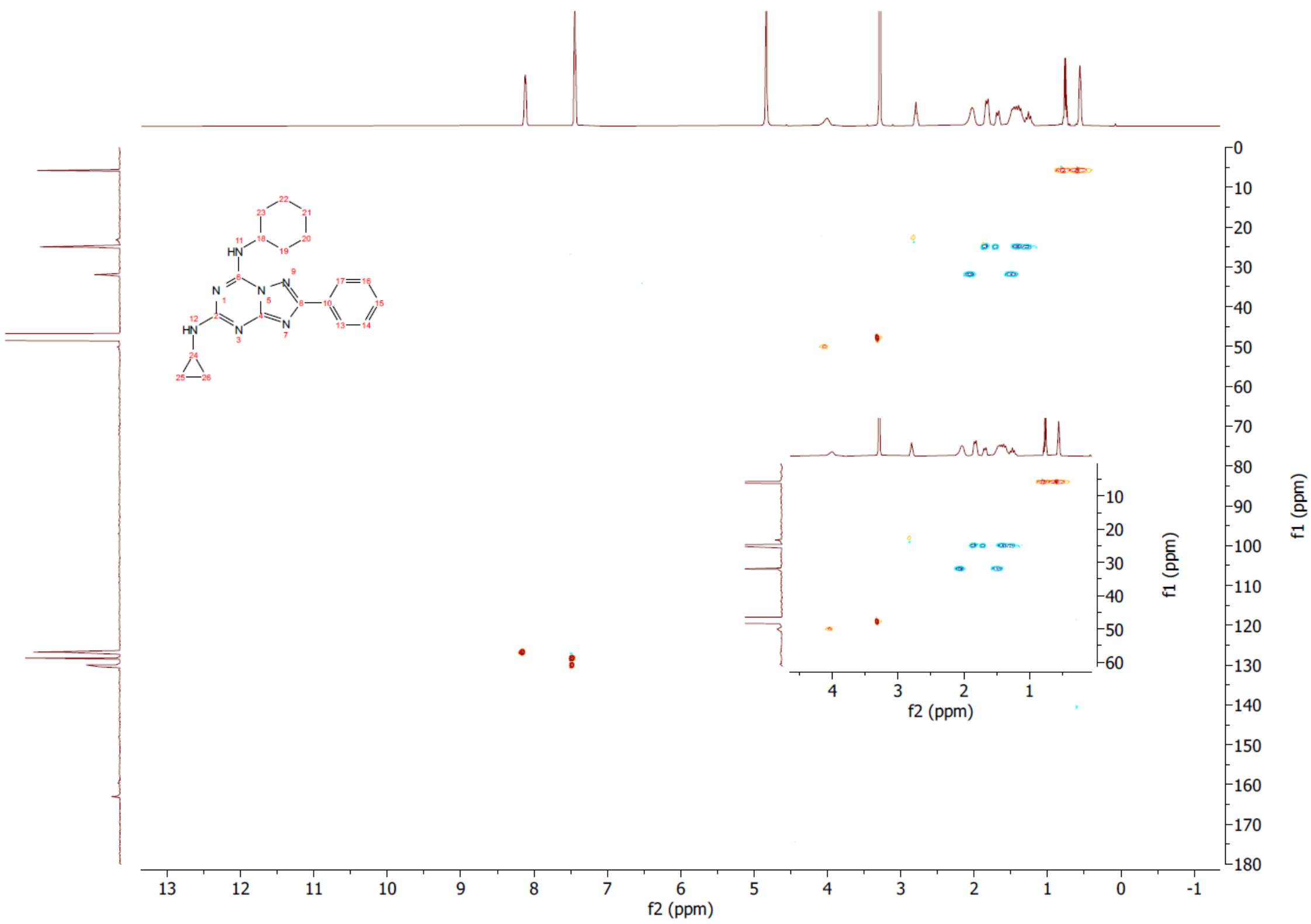

Figure S84. HSQC ^{1}H-^{13}C NMR spectrum of **Compound 21** (CD_3OD).

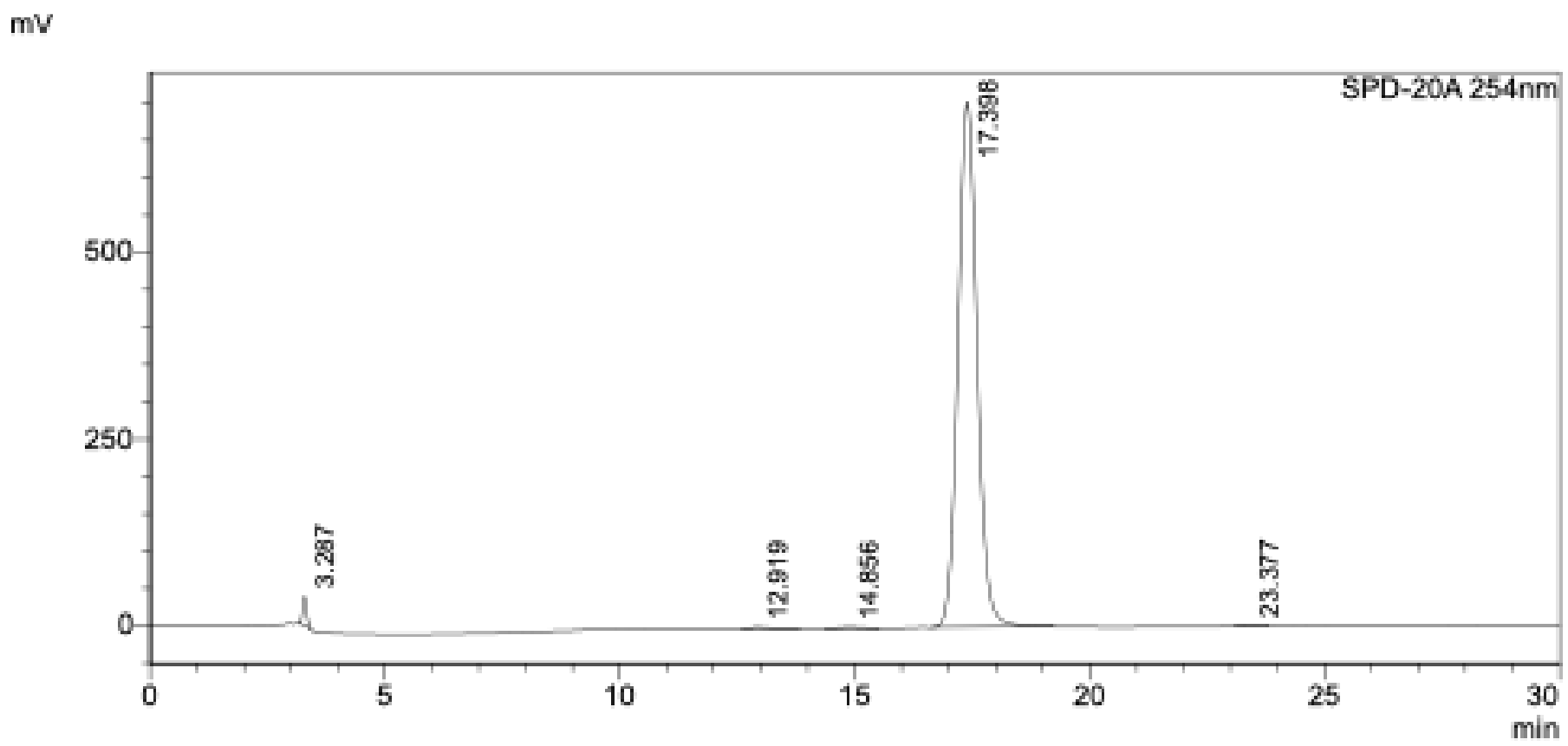

<Peak Table>

SPD-20A 254nm

Peak#	Ret. Time	Area	Height	Area%
1	3.287	241868	39007	1.189
2	12.919	46250	1748	0.227
3	14.856	31610	1175	0.155
4	17.398	19996190	702178	98.328
5	23.377	20322	1607	0.100
Total		20336241	745715	100.000

Figure S85. HPLC Chromatogram of **Compound 21**.

N5,N7-dicyclohexyl-2-phenyl-[1,2,4]triazolo[1,5-*a*][1,3,5]triazine-5,7-diamine **22**

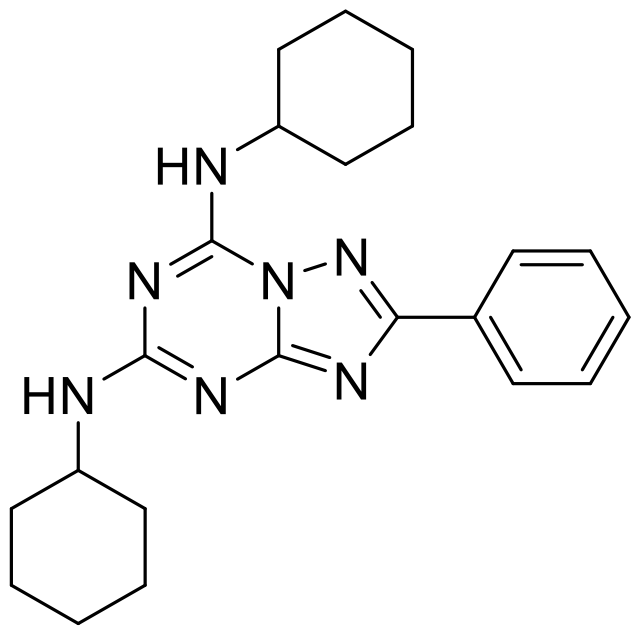

Chemical Formula: $C_{22}H_{29}N_7$
Molecular Weight: 391,51

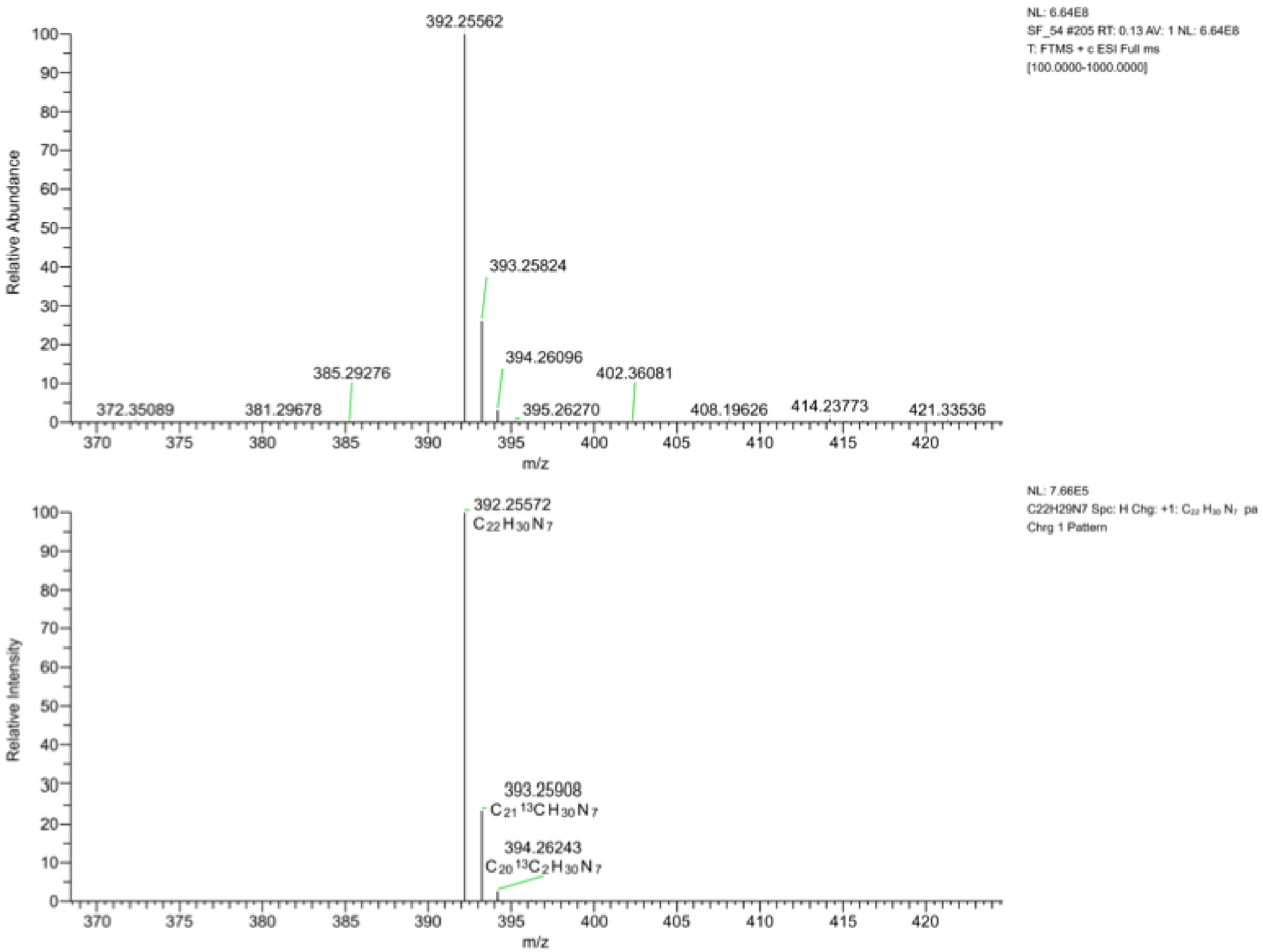

Figure S86. HRMS (ESI) spectrum of **Compound 22**.

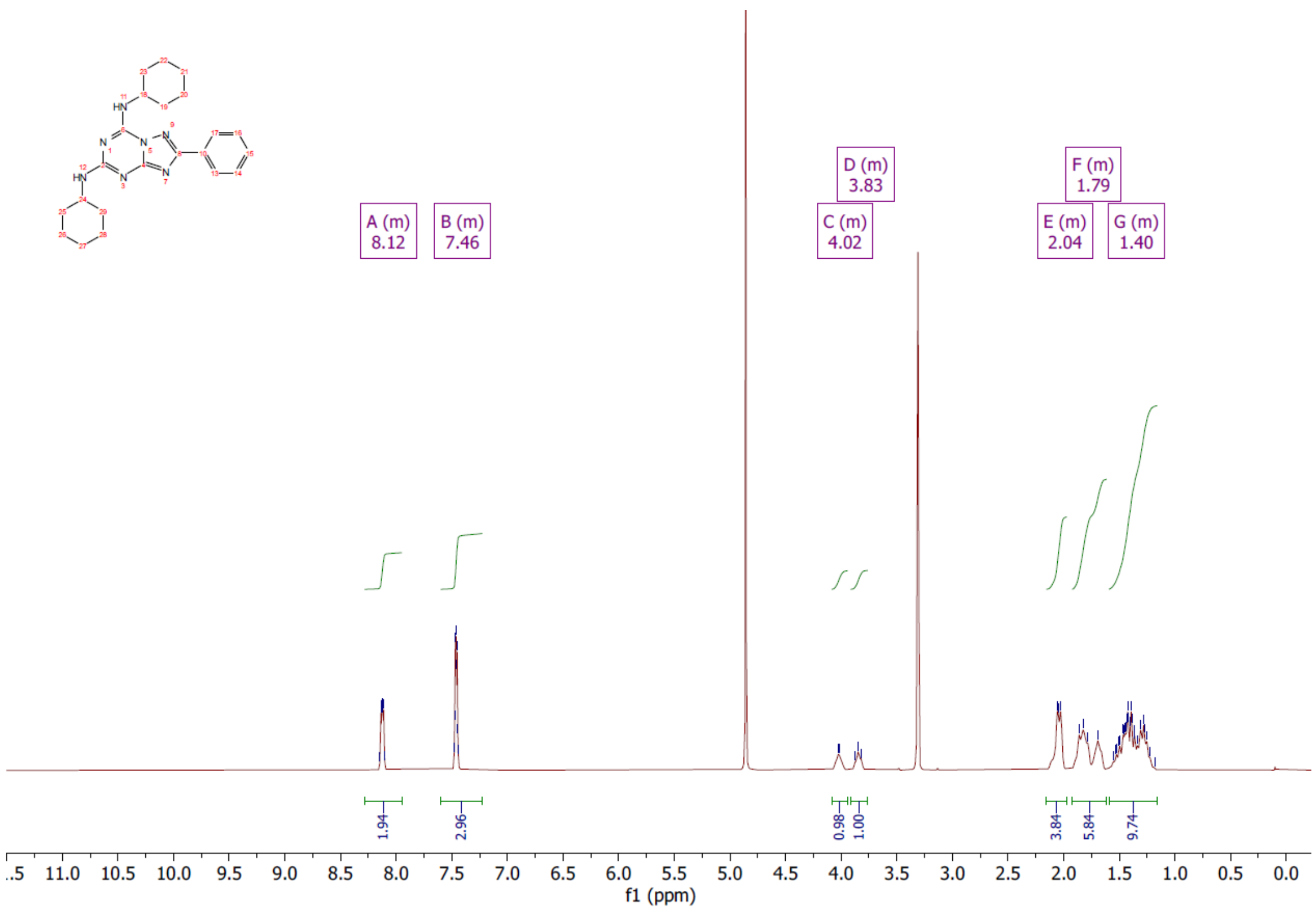

Figure S87. ^{1}H NMR spectrum of **Compound 22** (CD_3OD).

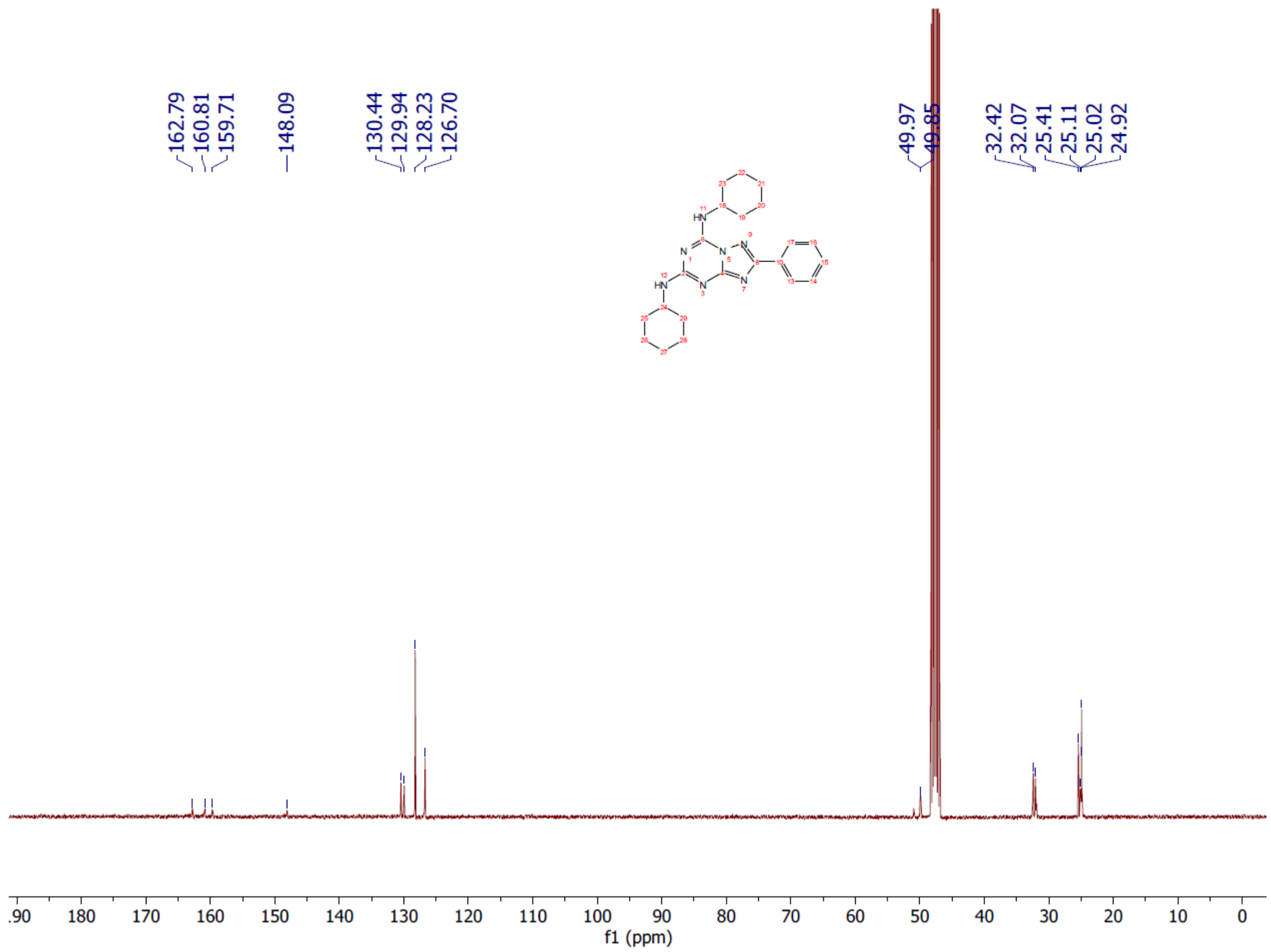

Figure S88. ^{13}C NMR spectrum of **Compound 22** (CD_3OD).

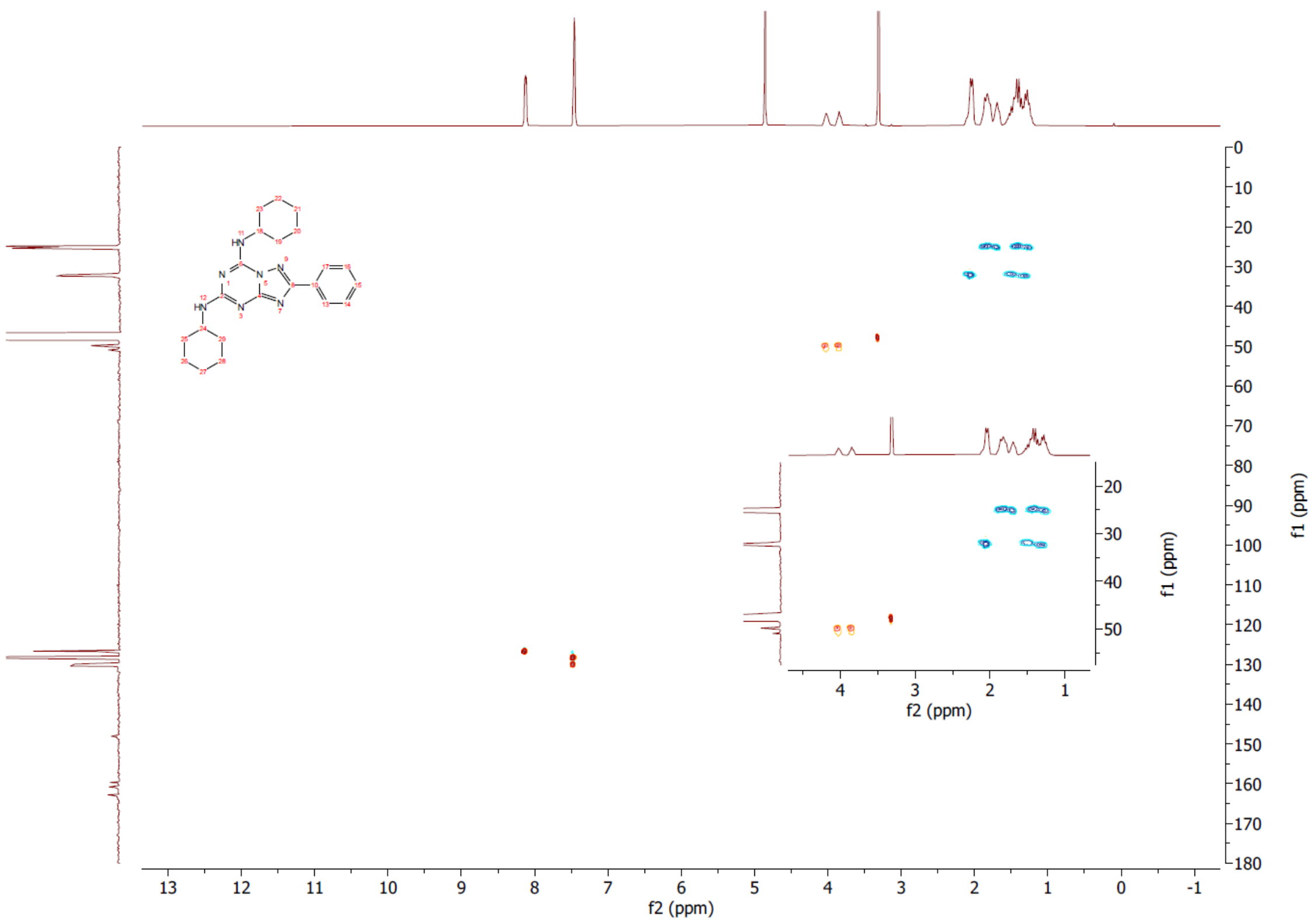

Figure S89. HSQC ^{1}H-^{13}C NMR spectrum of **Compound 22** (CD_3OD).

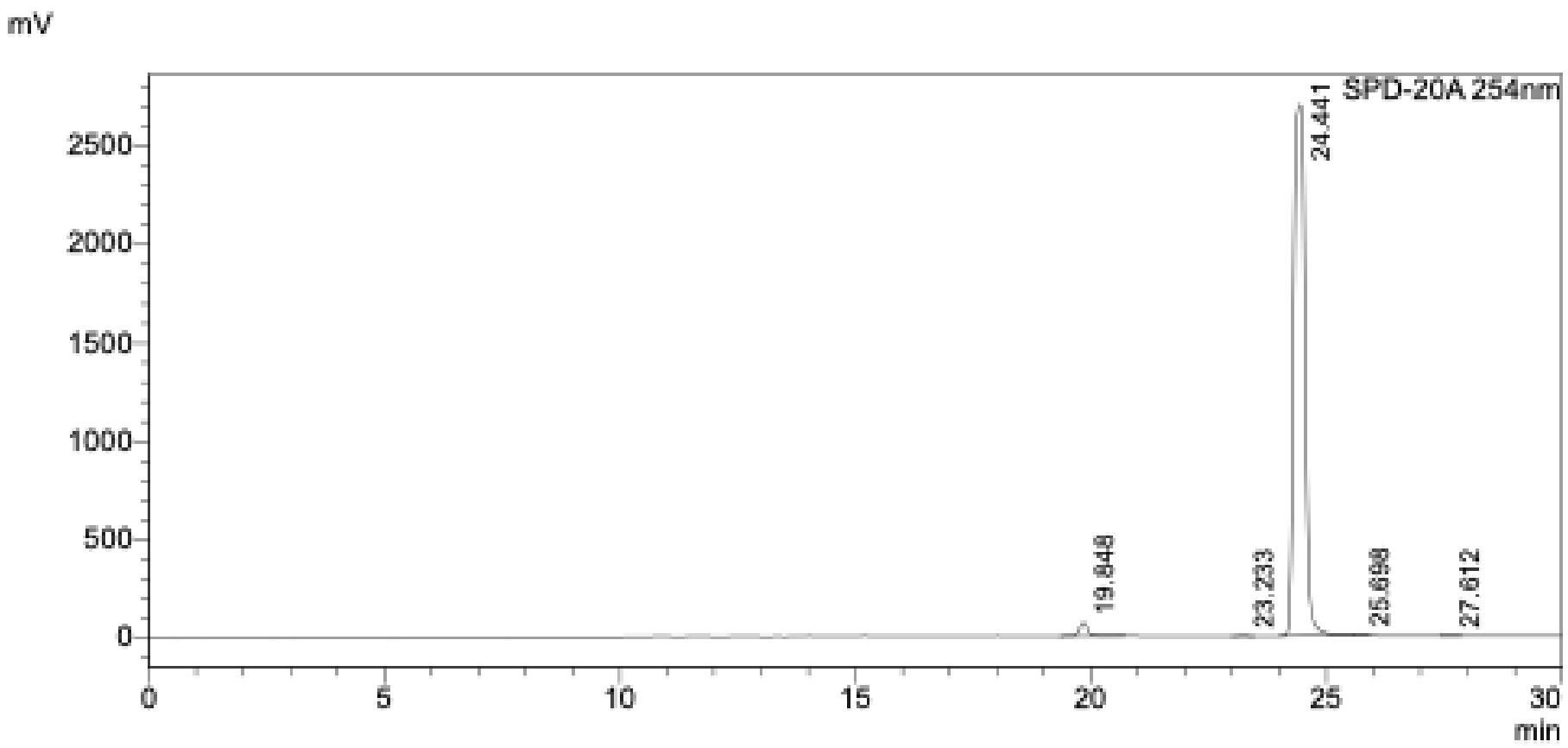

<Peak Table>

SPD-20A 254nm

Peak#	Ret. Time	Area	Height	Area%
1	19.848	834367	69275	1.825
2	23.233	12759	1142	0.028
3	24.441	44861038	2701515	98.110
4	25.698	10994	1108	0.024
5	27.612	6016	498	0.013
Total		45725173	2773537	100.000

Figure S90. HPLC Chromatogram of **Compound 22.**

N[7]-cyclohexyl-N[5]-benzyl-2-phenyl-[1,2,4]triazolo[1,5-*a*][1,3,5]triazine-5,7-diamine **23**

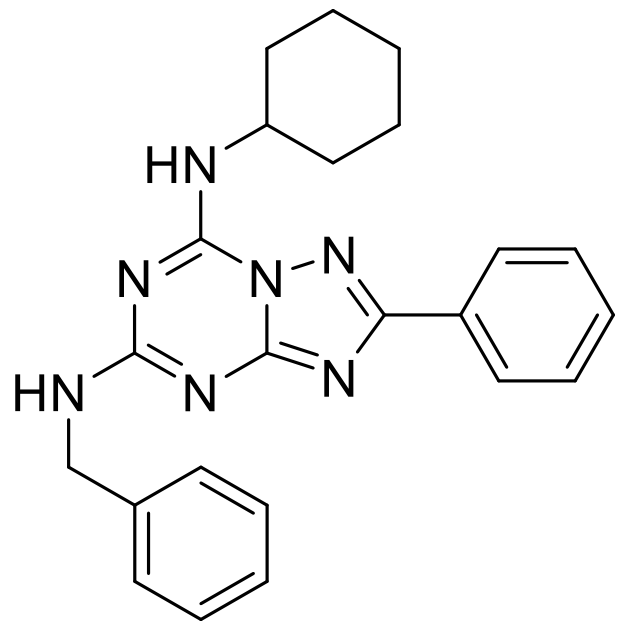

Chemical Formula: $C_{23}H_{25}N_7$
Molecular Weight: 399,49

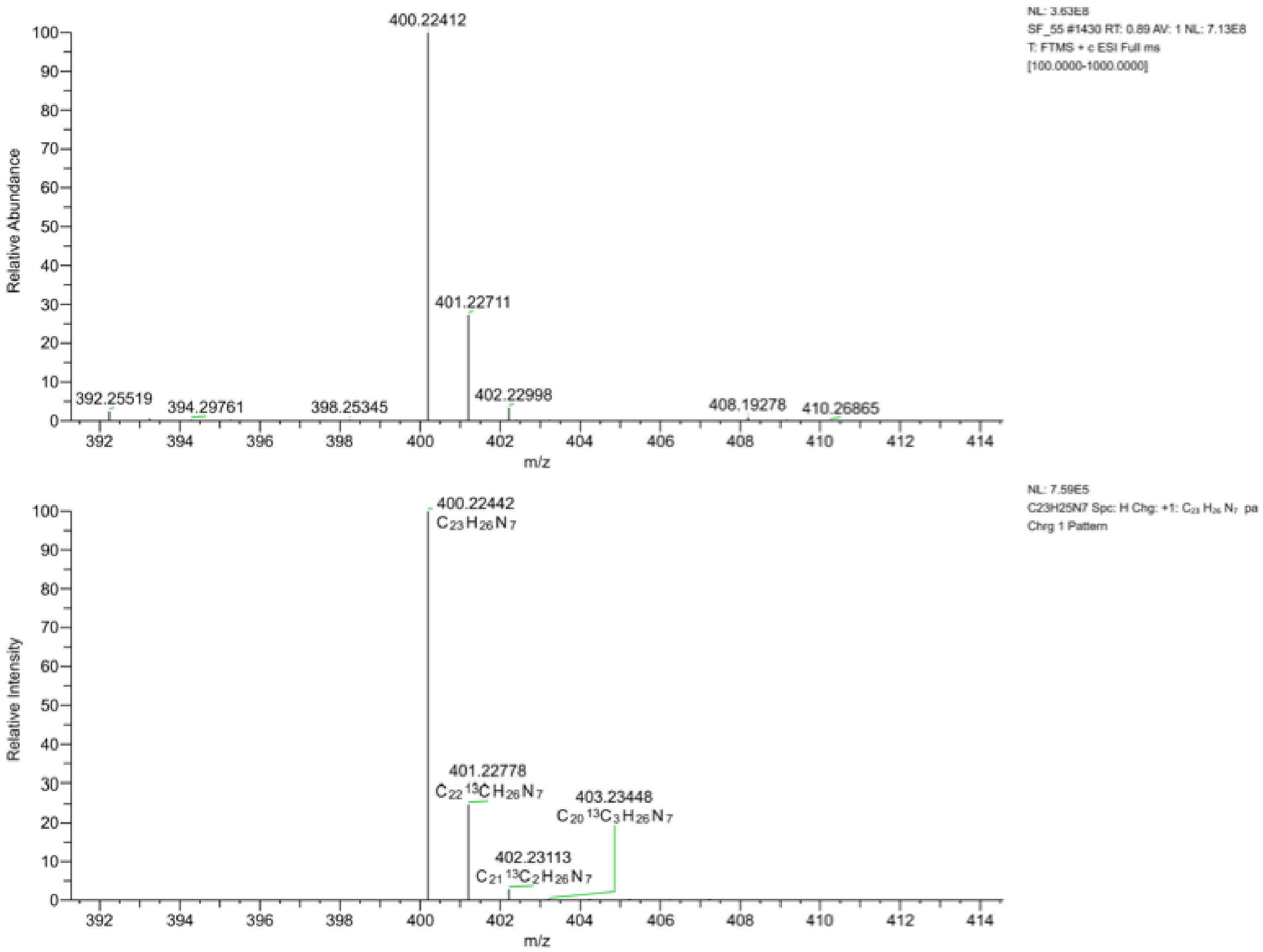

Figure S91. HRMS (ESI) spectrum of **Compound 23**.

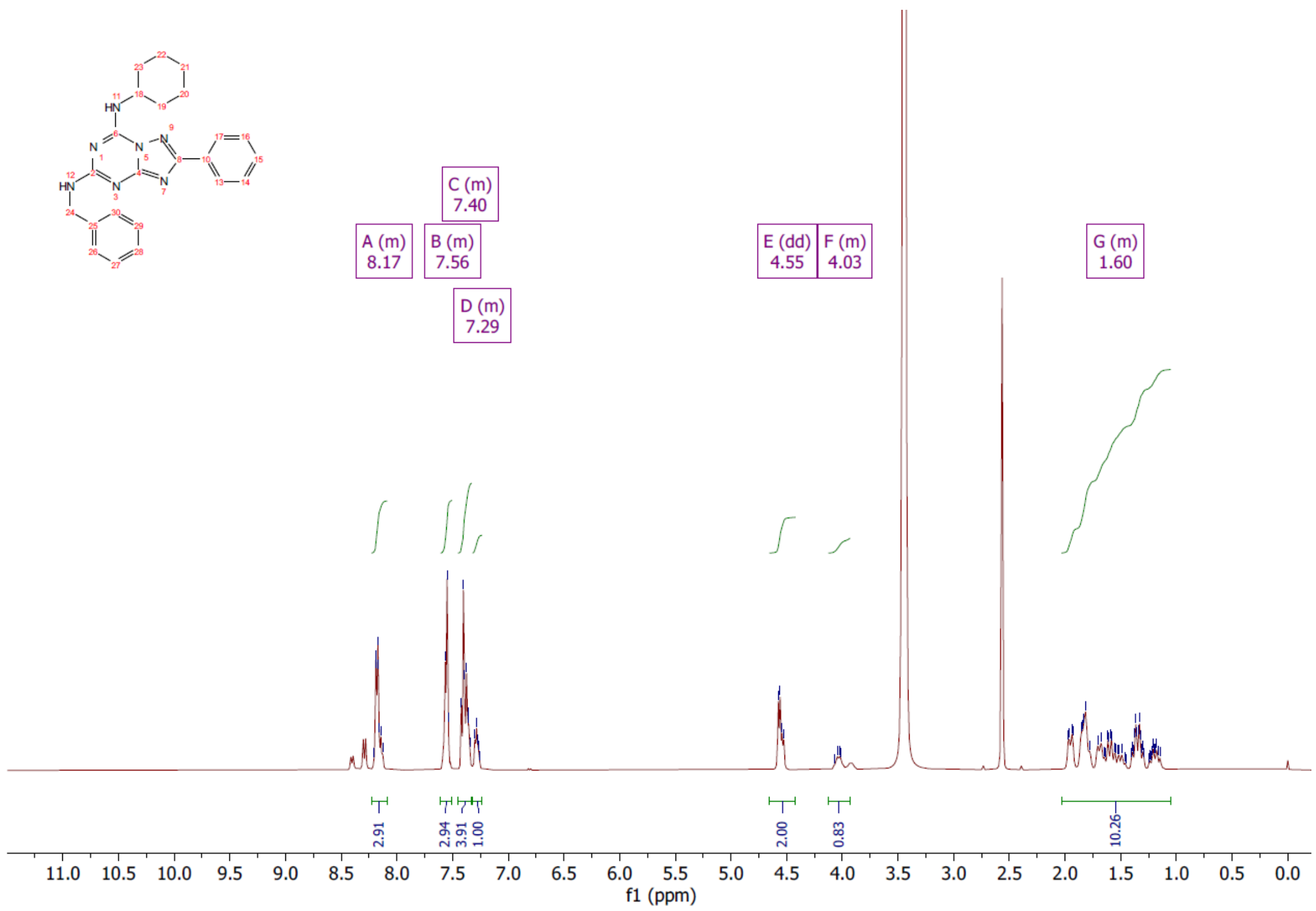

Figure S92. ^{1}H NMR spectrum of **Compound 23** (DMSO-d_6).

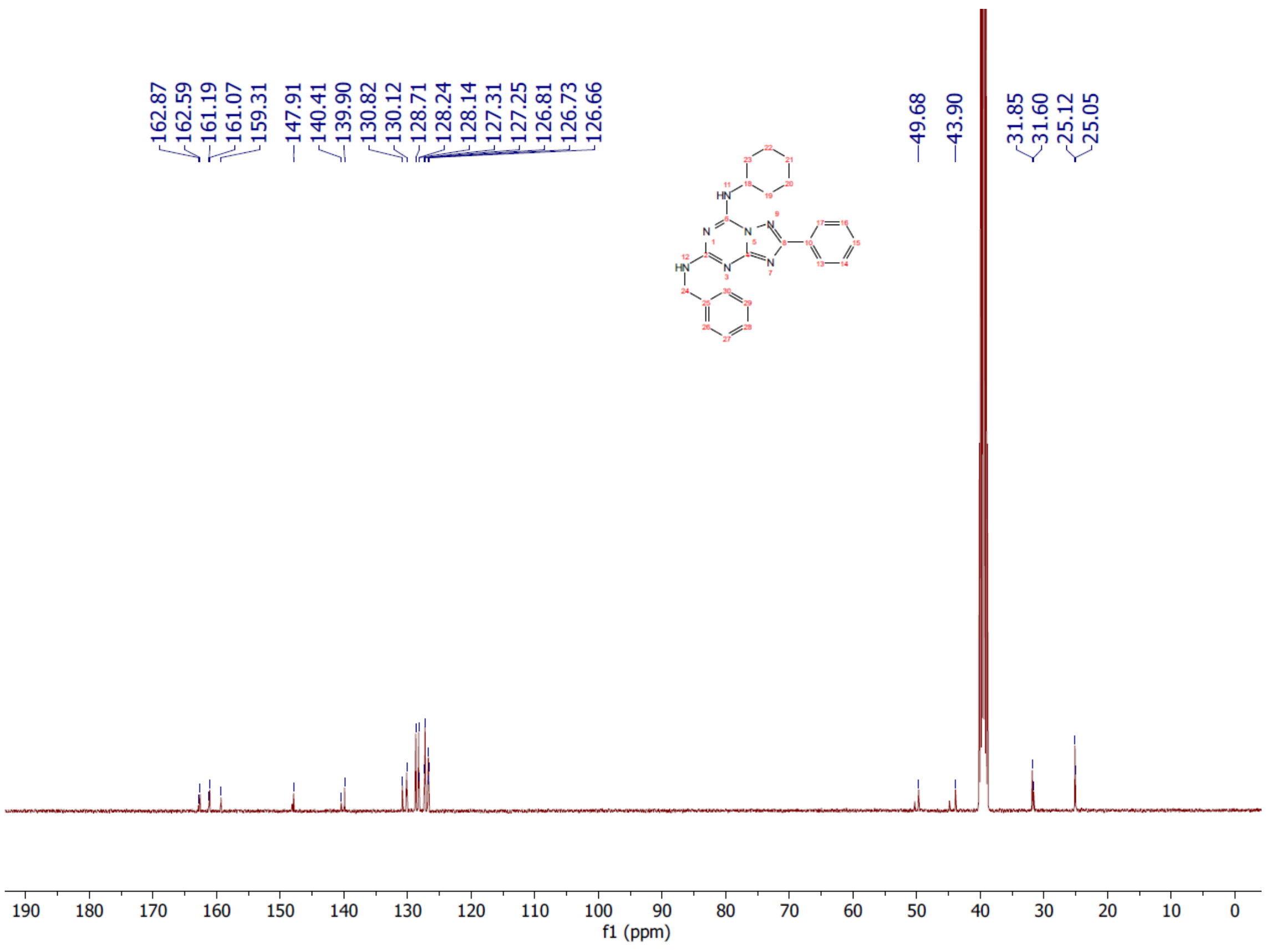

Figure S93. ^{13}C NMR spectrum of **Compound 23** (DMSO-d_6).

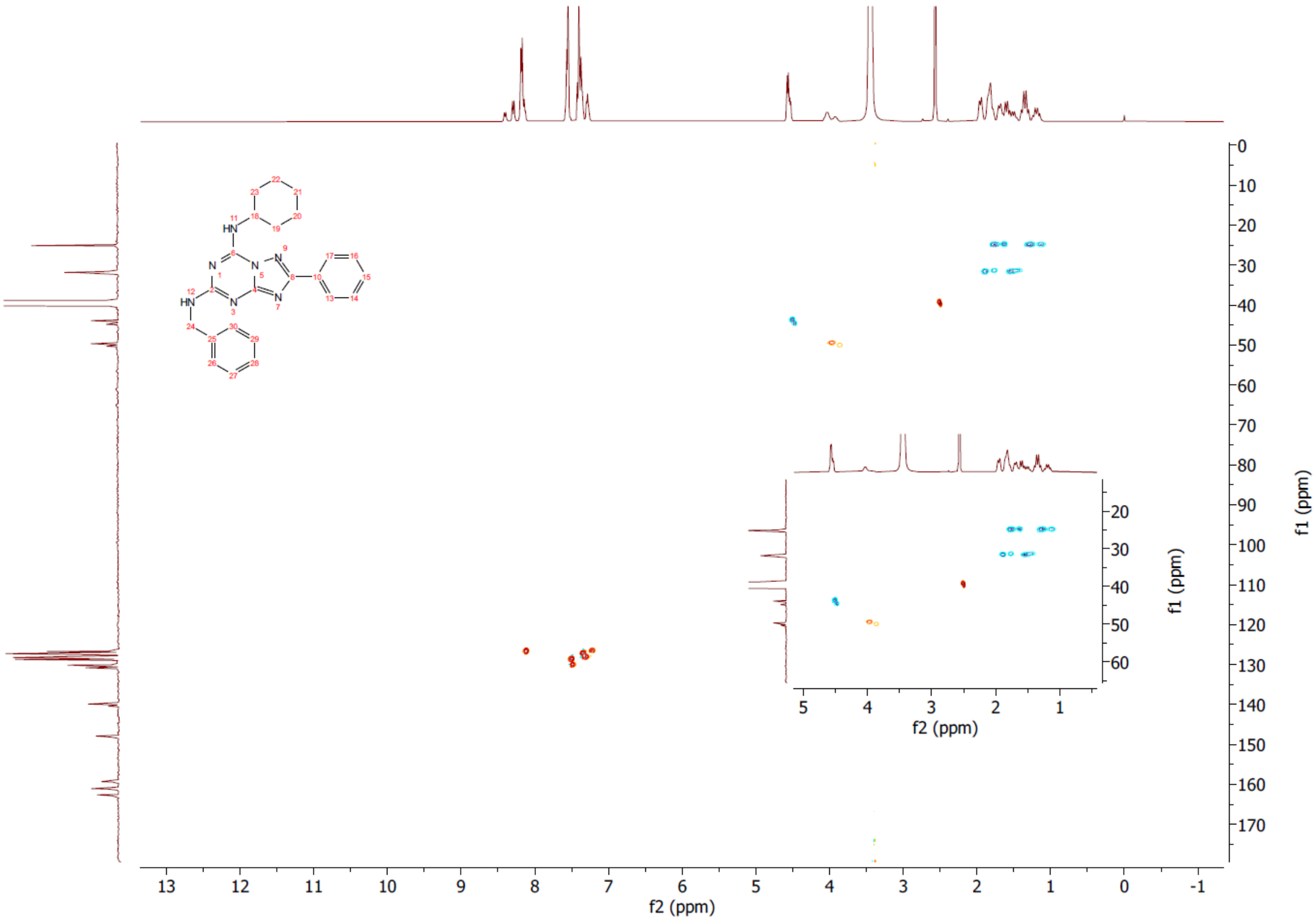

Figure S94. HSQC ^{1}H-^{13}C NMR spectrum of **Compound 23** (DMSO-d_6).

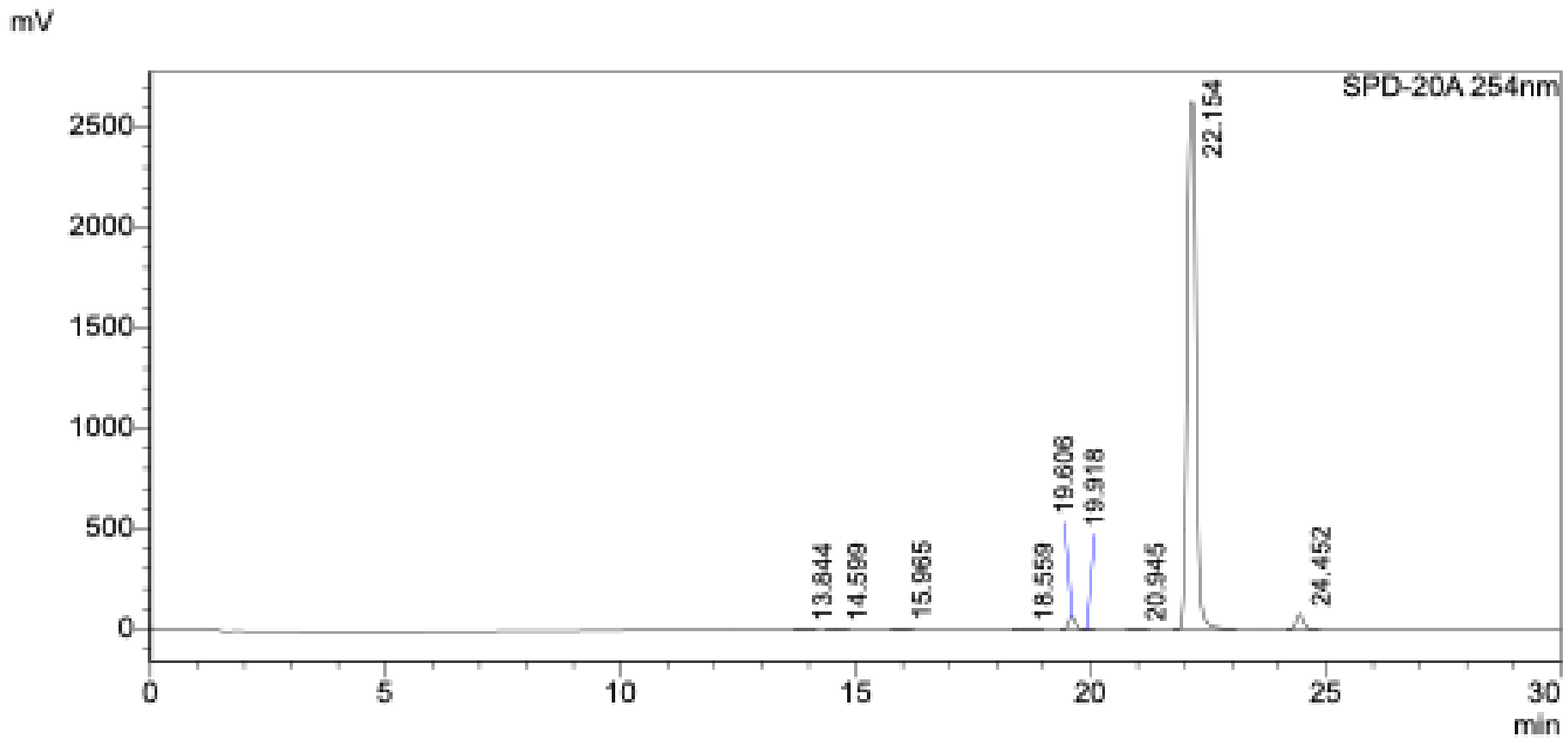

<Peak Table>

SPD-20A 254nm

Peak#	Ret. Time	Area	Height	Area%
1	13.844	26956	3015	0.075
2	14.599	14870	1679	0.041
3	15.965	74280	7760	0.206
4	18.559	33967	2843	0.094
5	19.606	624298	63645	1.735
6	19.918	7838	1159	0.022
7	20.945	13357	1421	0.037
8	22.154	34372867	2629321	95.530
9	24.452	812868	76945	2.259
Total		35981300	2787787	100.000

Figure S95. HPLC Chromatogram of **Compound 23.**

N[7]-benzyl-N[5]-methyl-2-phenyl-[1,2,4]triazolo[1,5-*a*][1,3,5]triazine-5,7-diamine **24**

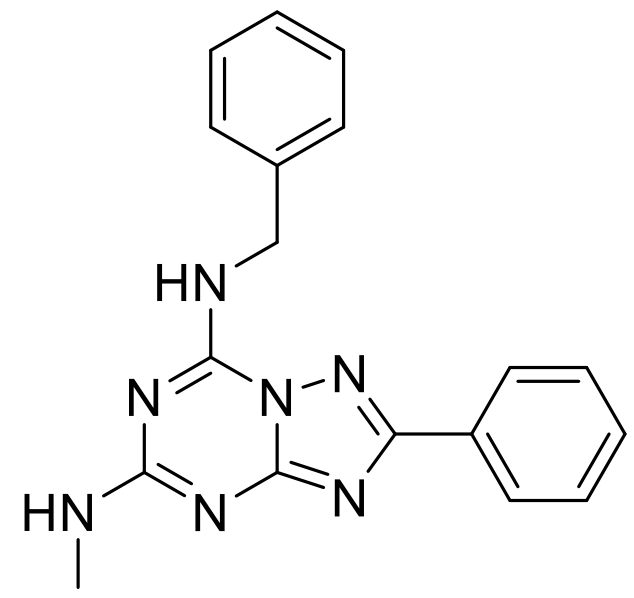

Chemical Formula: $C_{18}H_{17}N_7$
Molecular Weight: 331,37

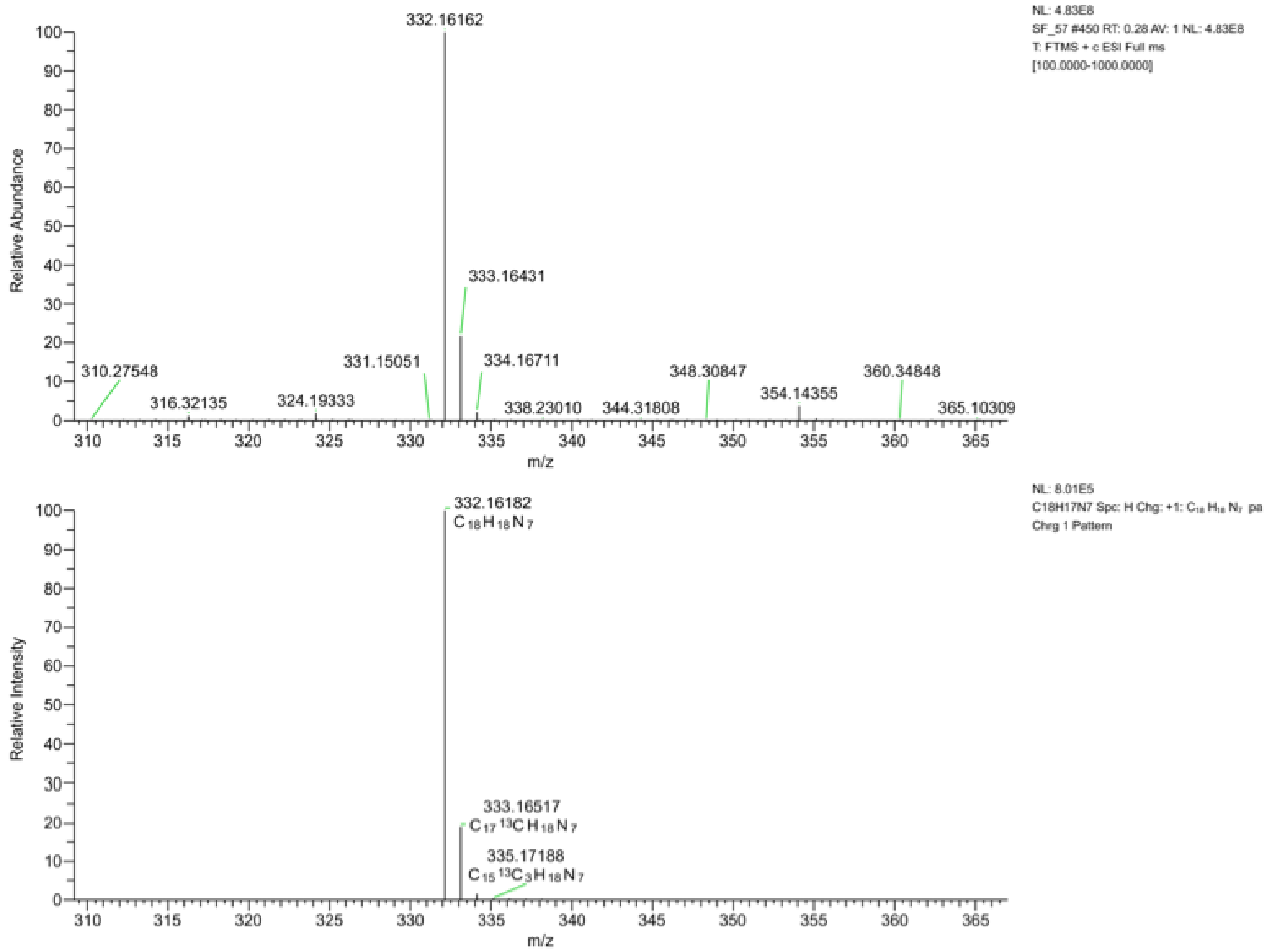

Figure S96. HRMS (ESI) spectrum of **Compound 24**.

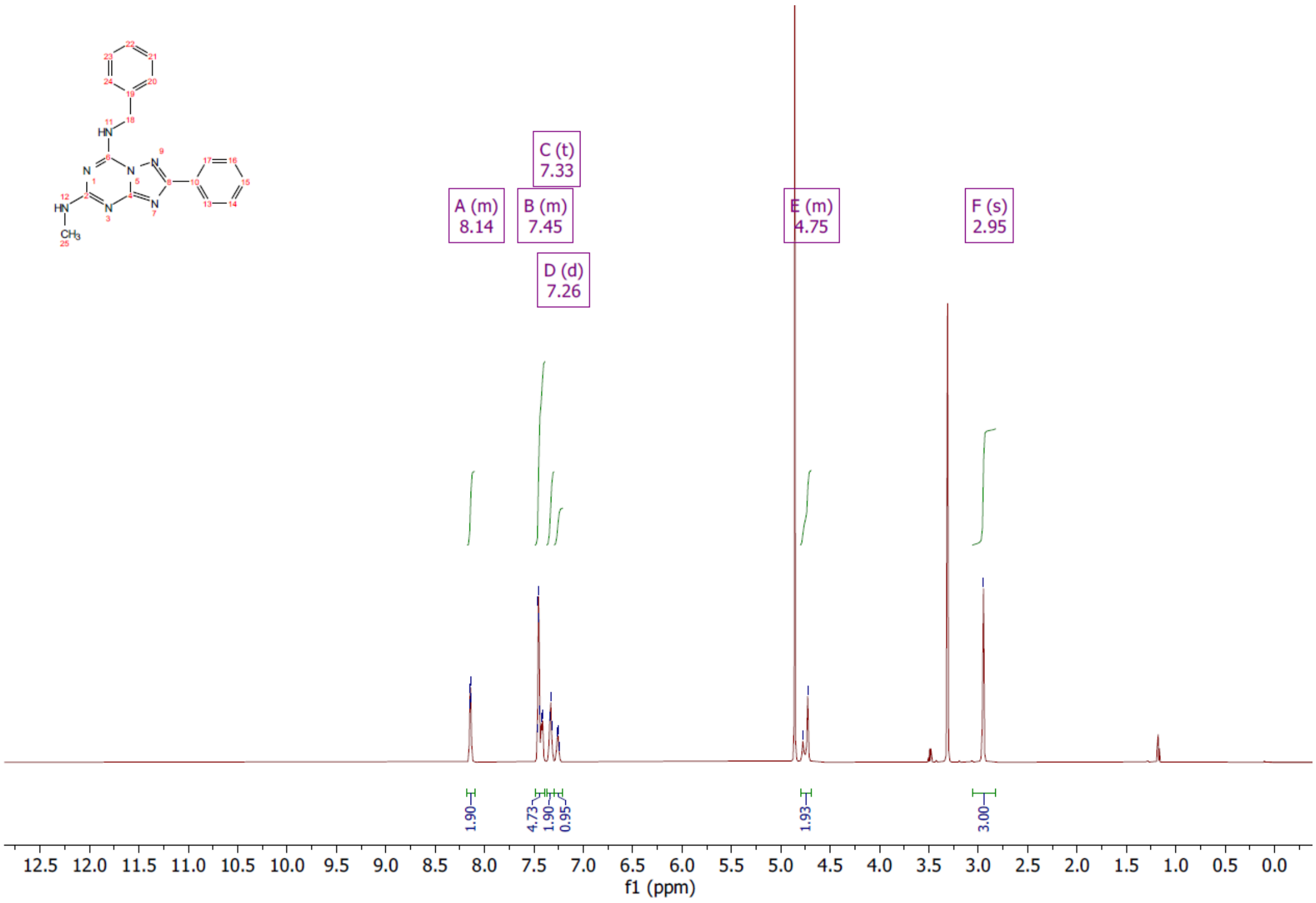

Figure S97. ^1H NMR spectrum of **Compound 24** (CD_3OD).

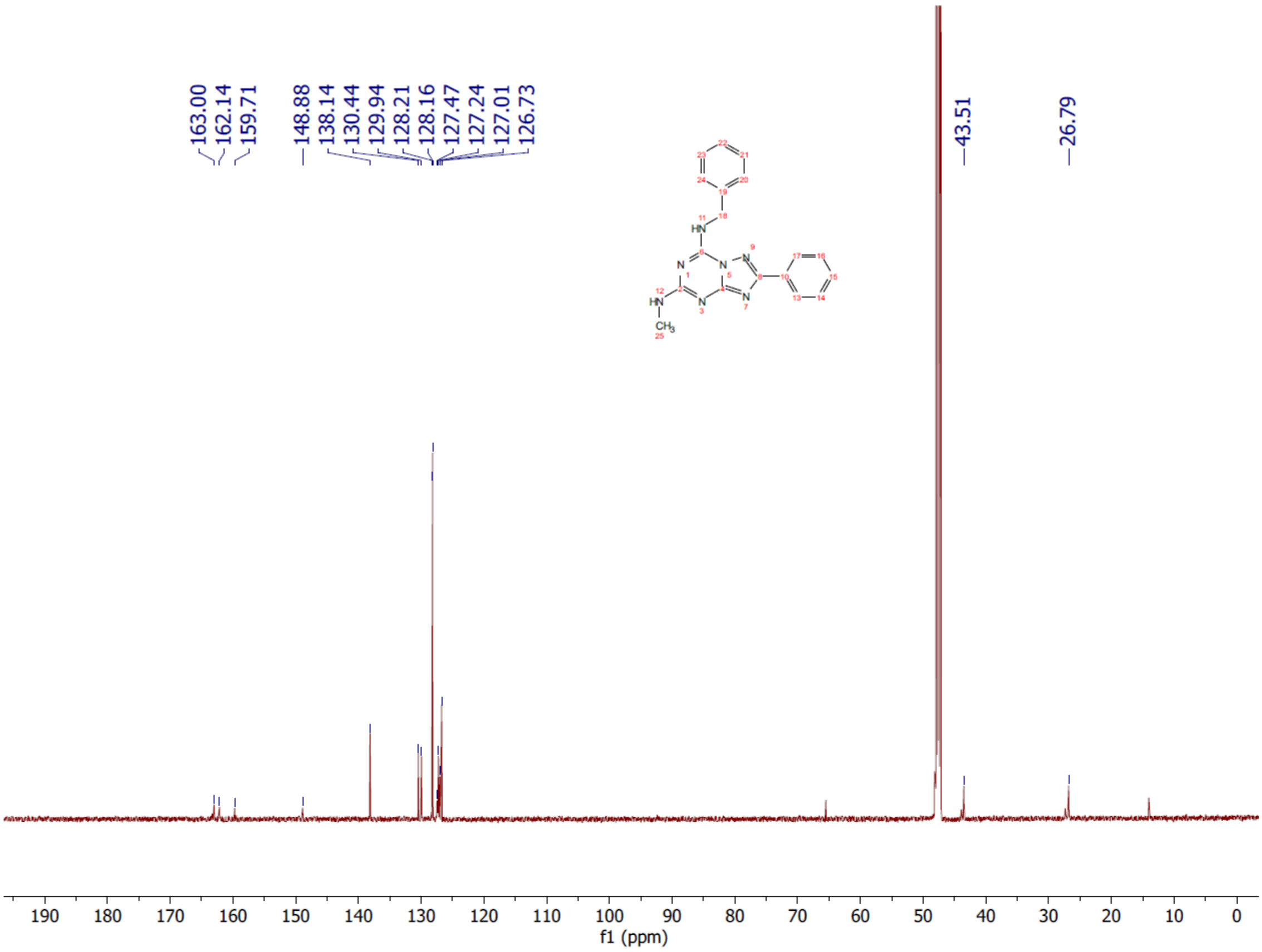

Figure S98. ^{13}C NMR spectrum of **Compound 24** (CD_3OD).

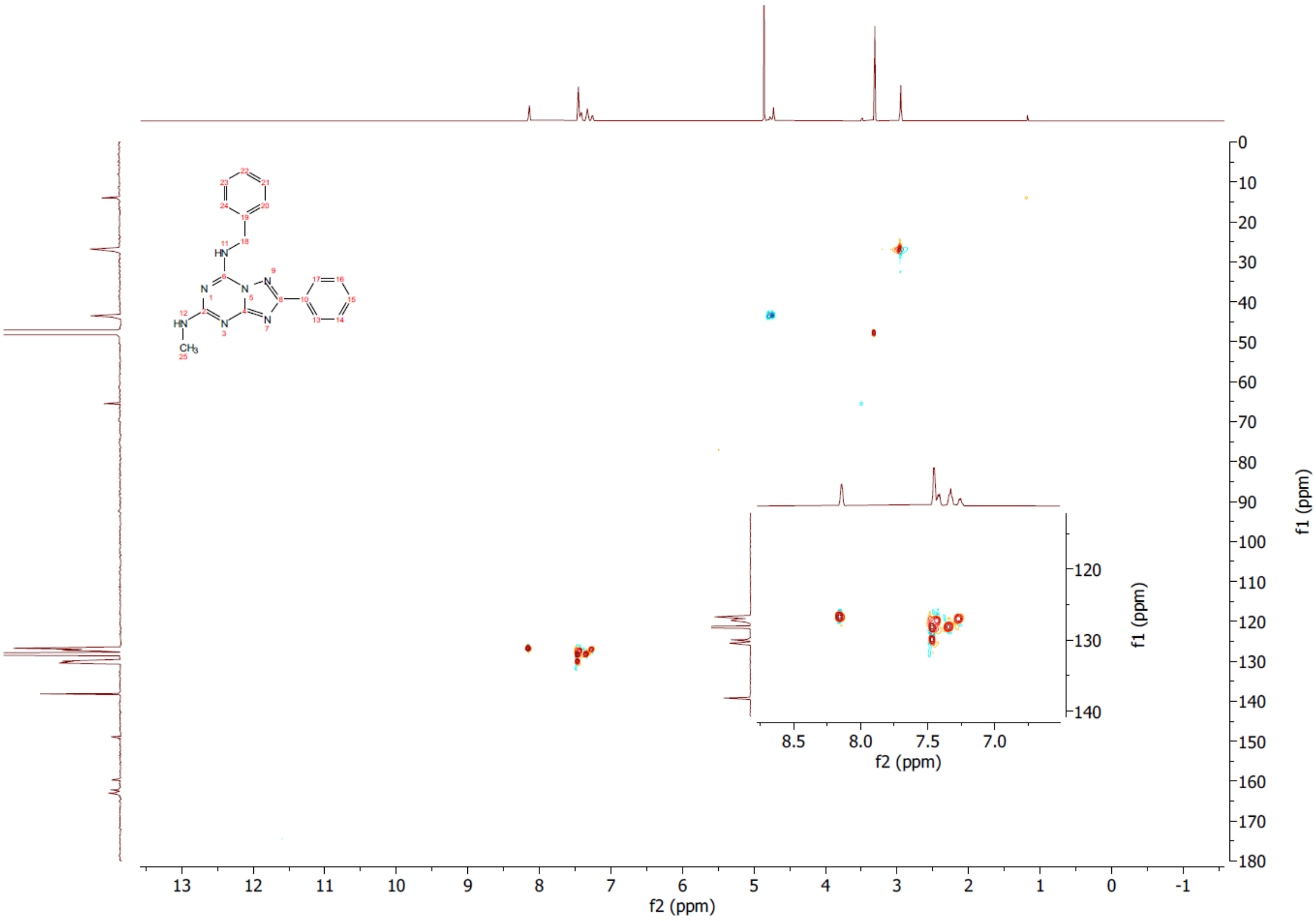

Figure S99. HSQC ^{1}H-^{13}C NMR spectrum of **Compound 24** (CD_3OD).

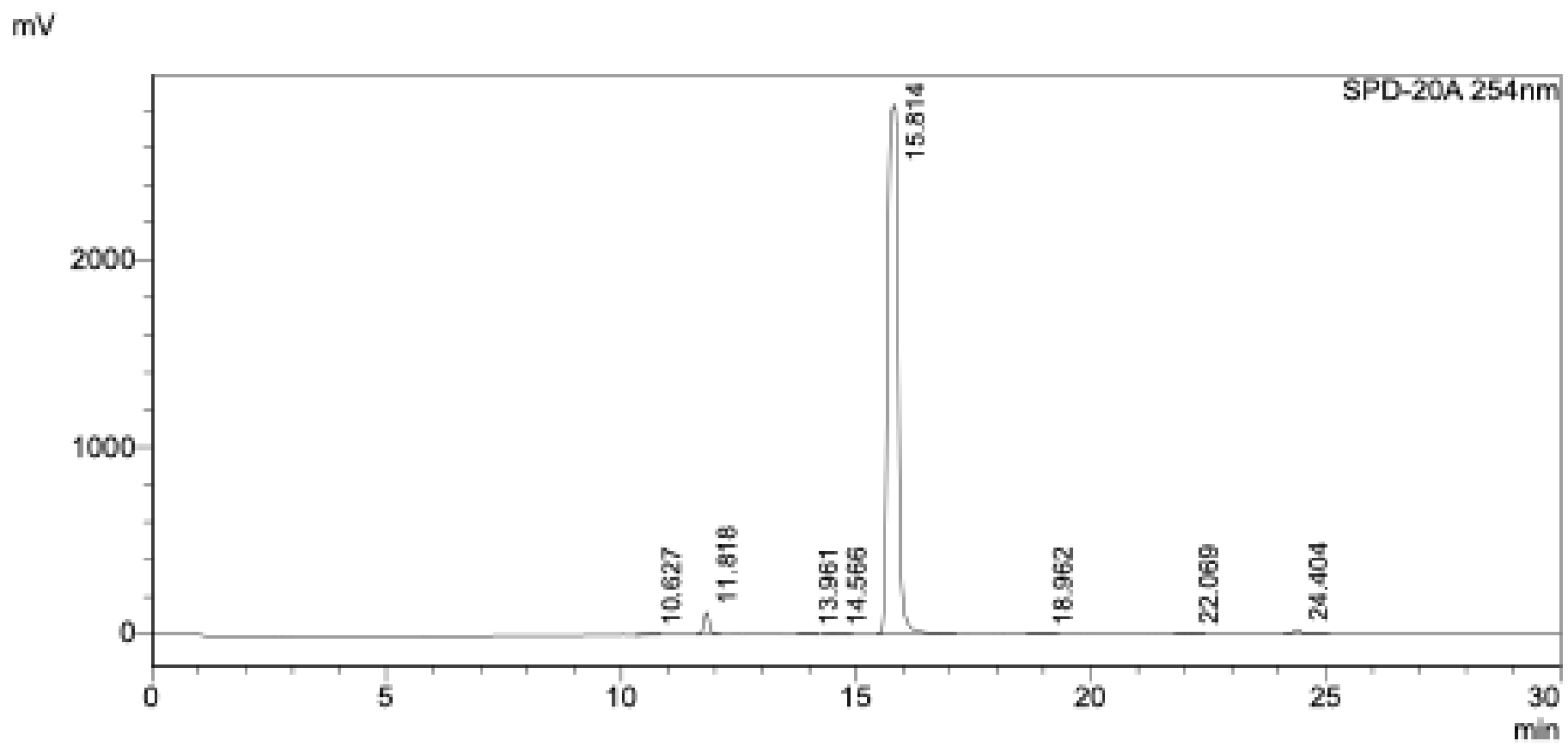

<Peak Table>

SPD-20A 254nm

Peak#	Ret. Time	Area	Height	Area%
1	10.627	30044	3503	0.066
2	11.818	930508	119884	2.041
3	13.961	25135	2961	0.055
4	14.566	28459	2644	0.062
5	15.814	44160534	2830743	96.887
6	18.962	39066	3378	0.086
7	22.069	79024	7684	0.173
8	24.404	286871	27012	0.629
Total		45579641	2997810	100.000

Figure S100. HPLC Chromatogram of **Compound 24.**

N^7-benzyl-N^5-cyclopropyl-2-phenyl-[1,2,4]triazolo[1,5-*a*][1,3,5]triazine-5,7-diamine **25**

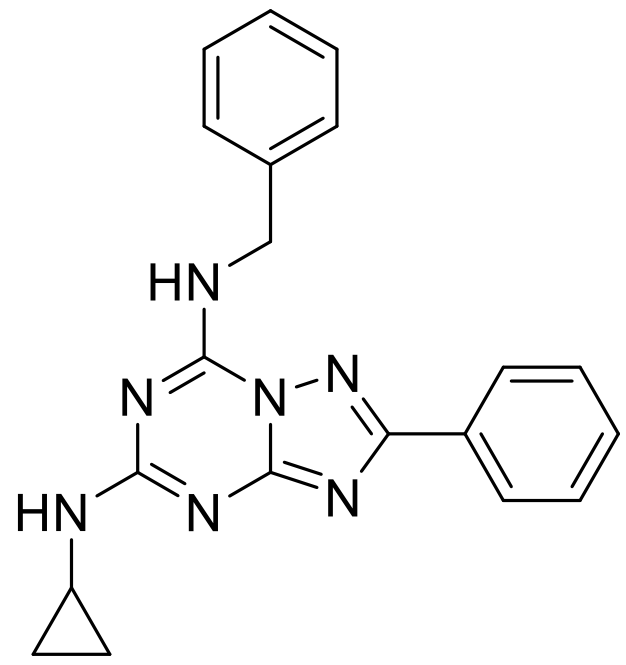

Chemical Formula: $C_{20}H_{19}N_7$
Molecular Weight: 357,41

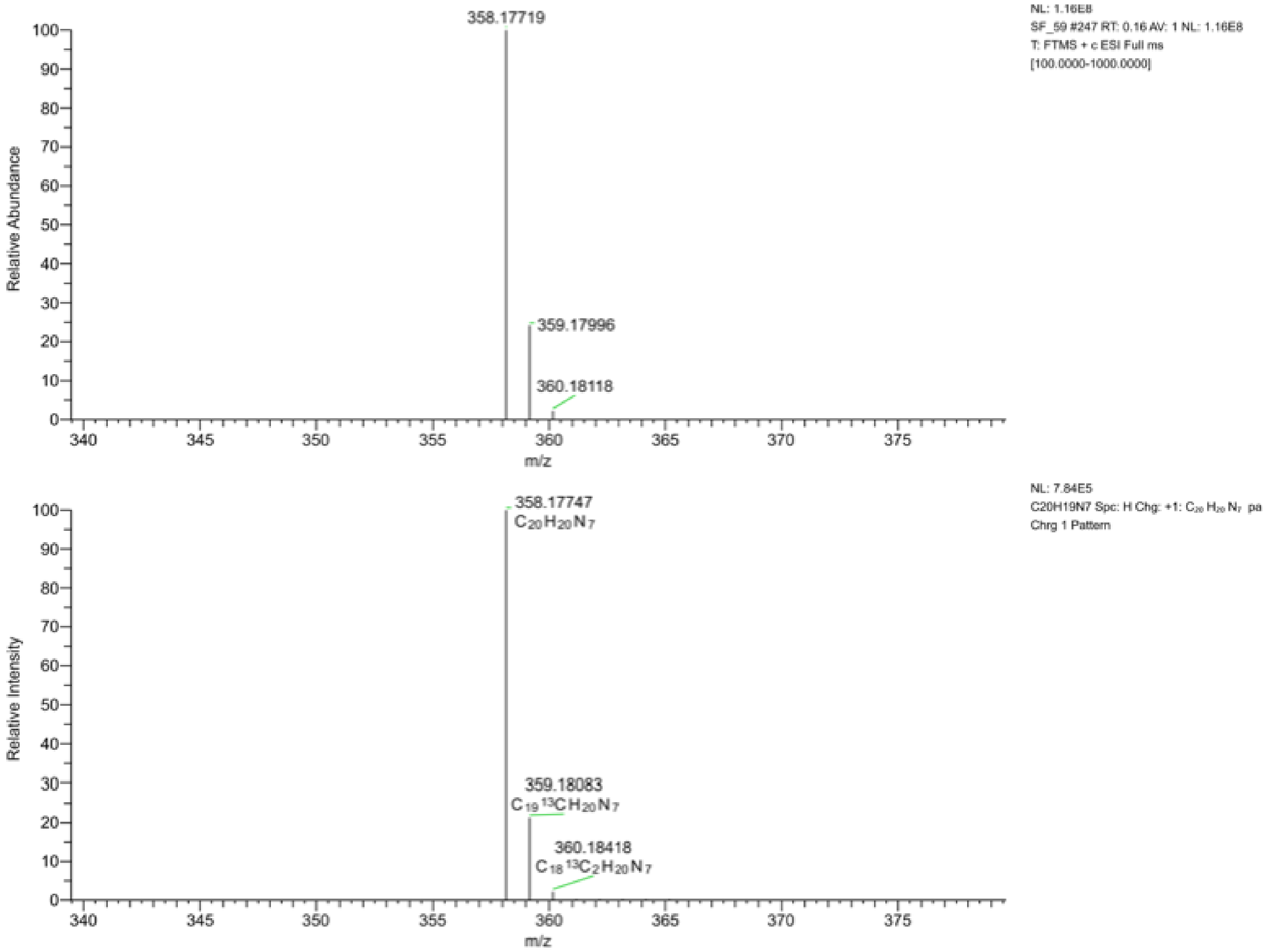

Figure S101. HRMS (ESI) spectrum of **Compound 25**.

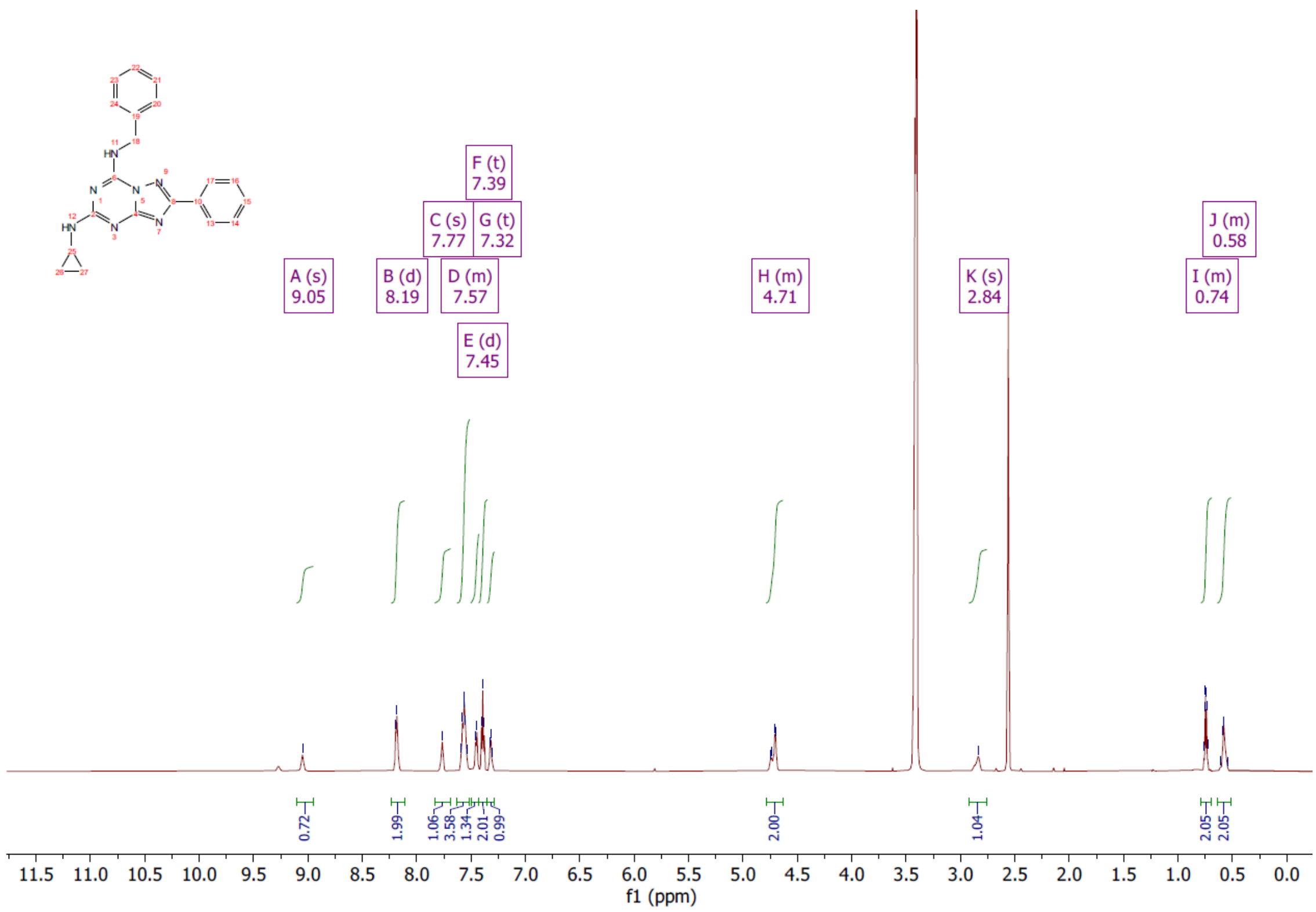

Figure S102. ^{1}H NMR spectrum of **Compound 25** (DMSO-d_6).

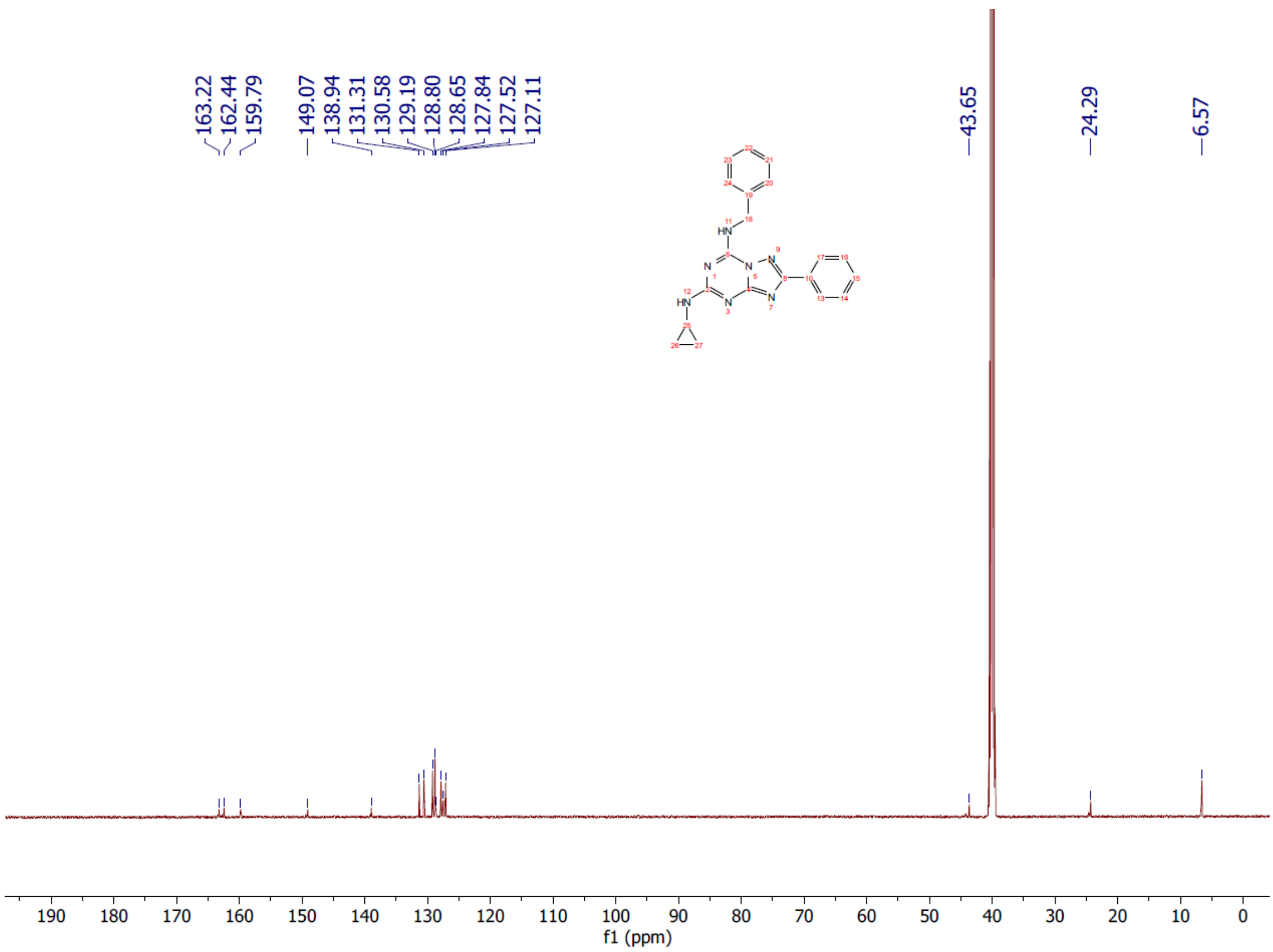

Figure S103. ^{13}C NMR spectrum of **Compound 25** (DMSO-d_6).

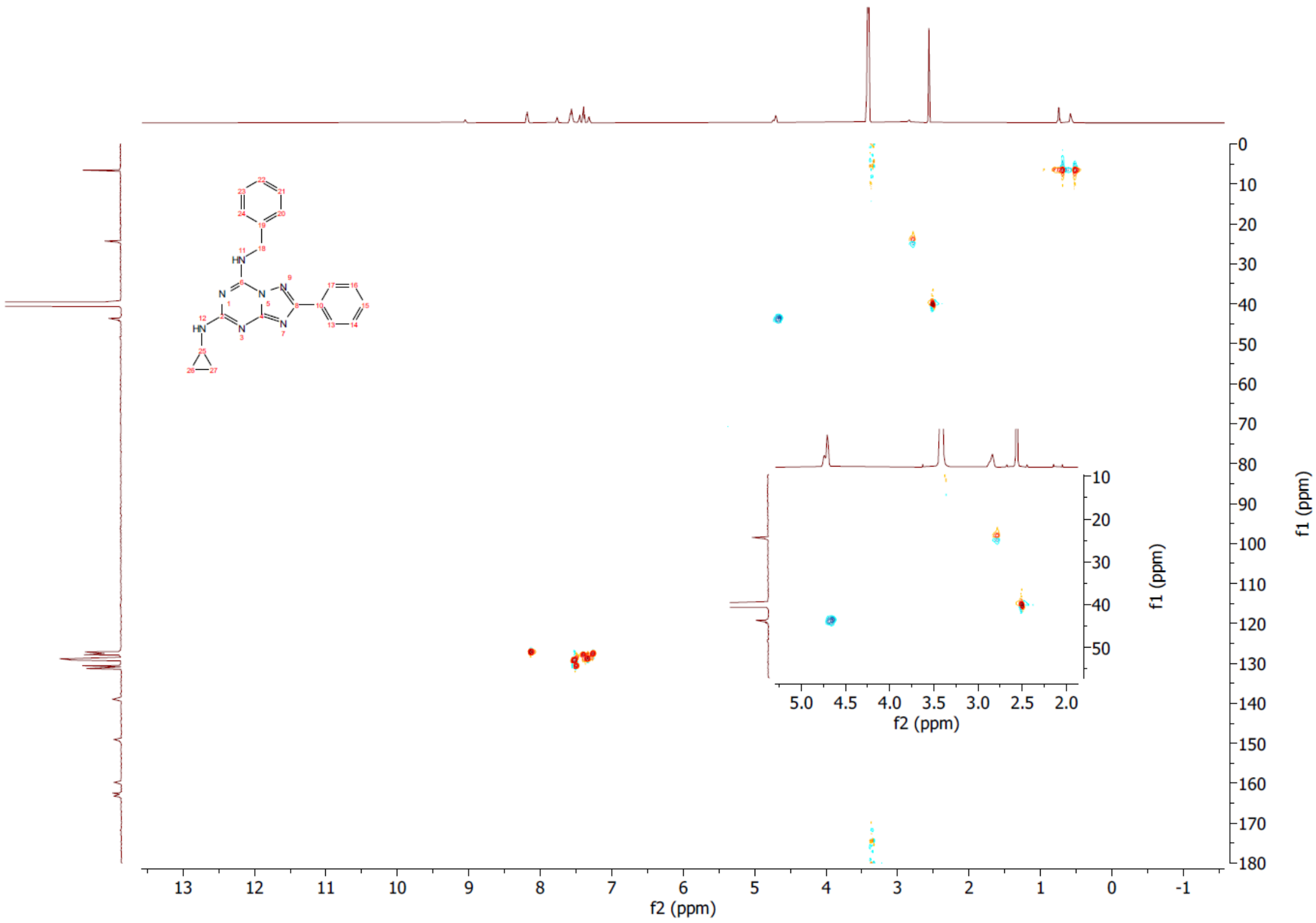

Figure S104. HSQC ^1H-^{13}C NMR spectrum of **Compound 25** (DMSO-*d*$_6$).

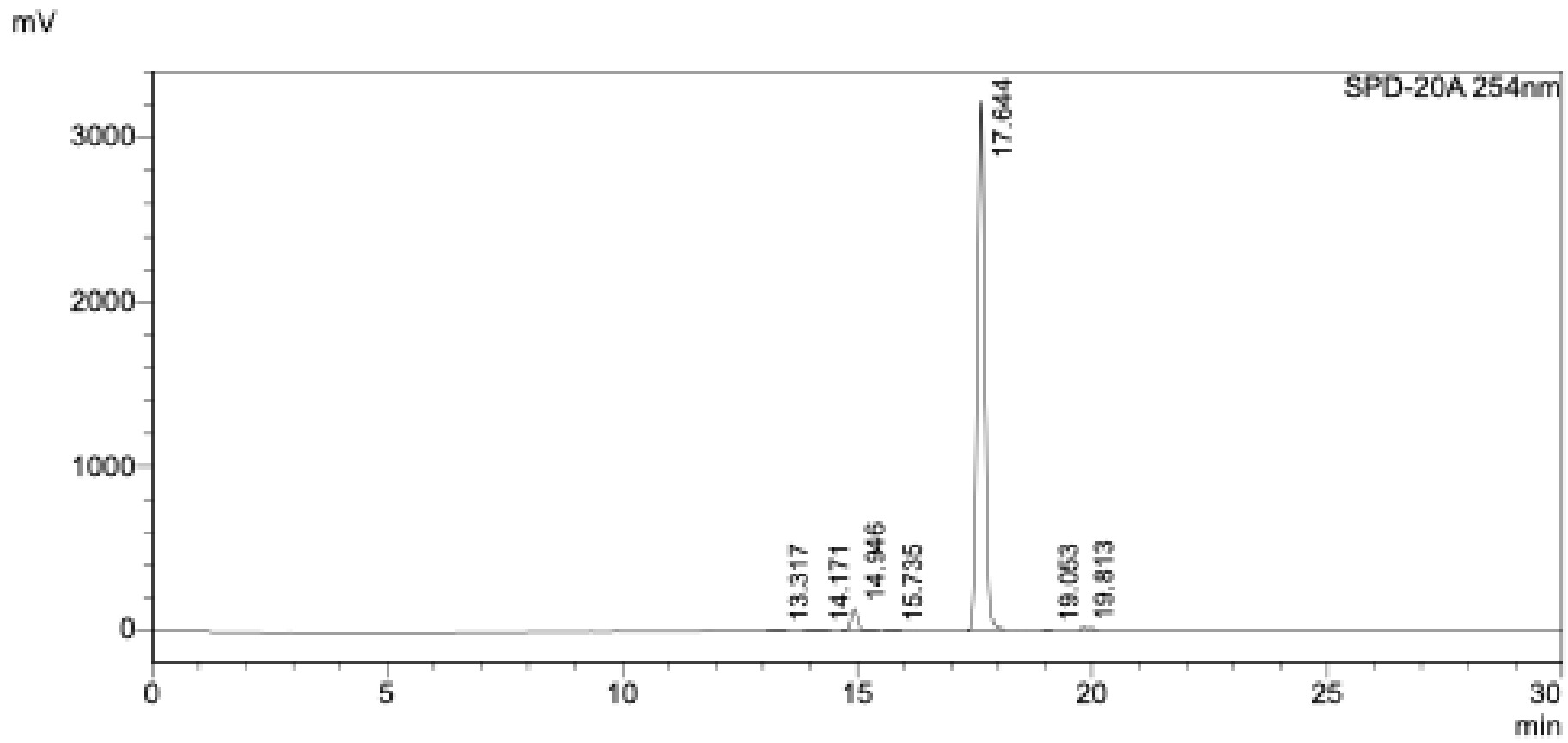

<Peak Table>

SPD-20A 254nm

Peak#	Ret. Time	Area	Height	Area%
1	13.317	35773	4226	0.102
2	14.171	52342	5415	0.150
3	14.946	1331512	133979	3.809
4	15.735	42479	5040	0.122
5	17.644	33403942	3219209	95.547
6	19.053	15438	2652	0.044
7	19.813	79246	6939	0.227
Total		34960731	3377459	100.000

Figure S105. HPLC Chromatogram of **Compound 25.**

N^7-benzyl-N^5-cyclohexyl-2-phenyl-[1,2,4]triazolo[1,5-*a*][1,3,5]triazine-5,7-diamine **26**

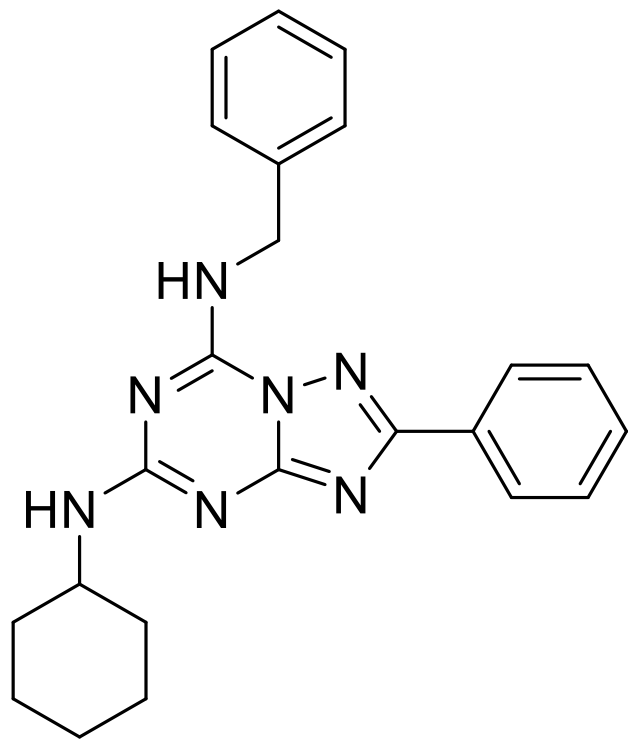

Chemical Formula: $C_{23}H_{25}N_7$
Molecular Weight: 399.49

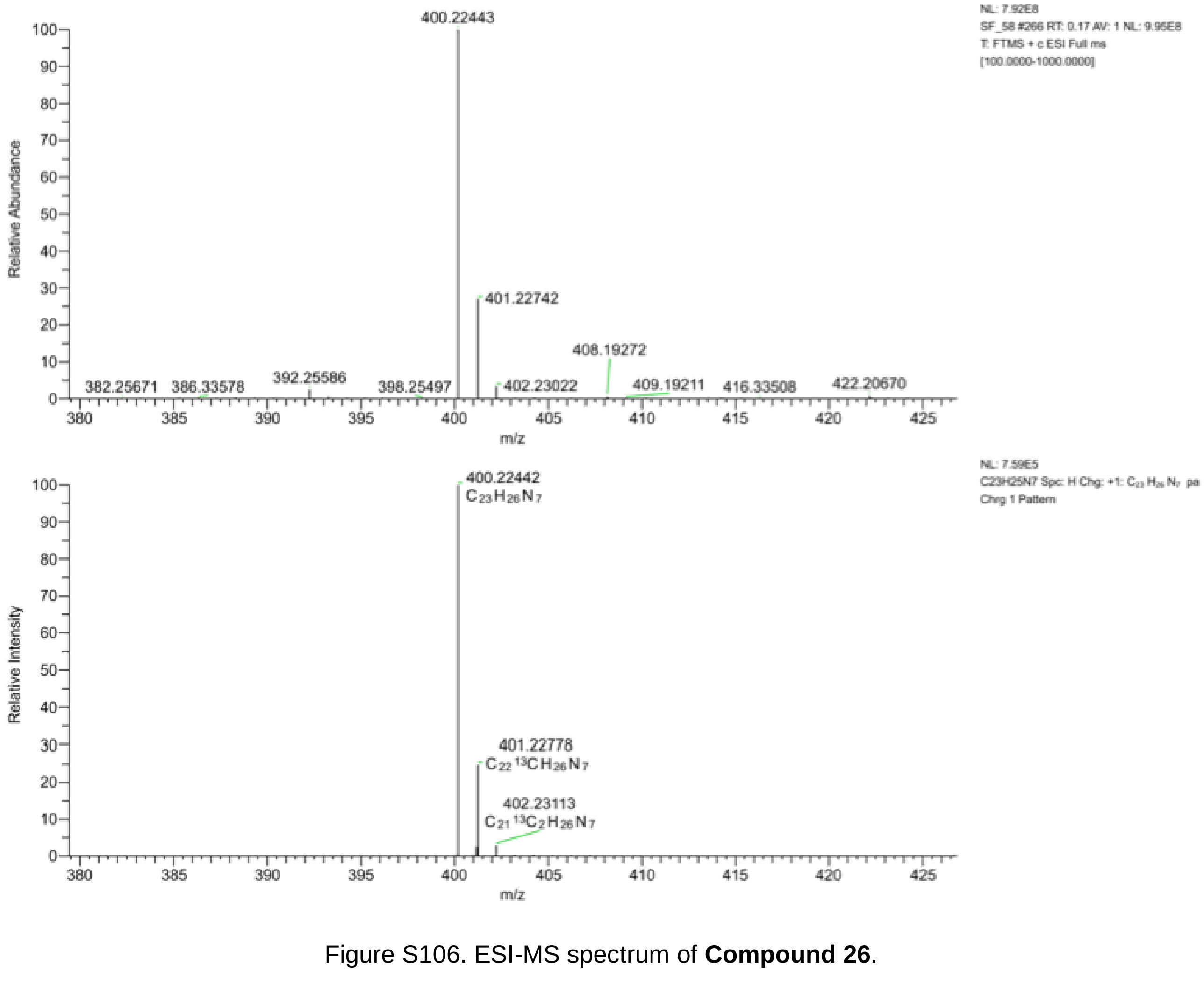

Figure S106. ESI-MS spectrum of **Compound 26**.

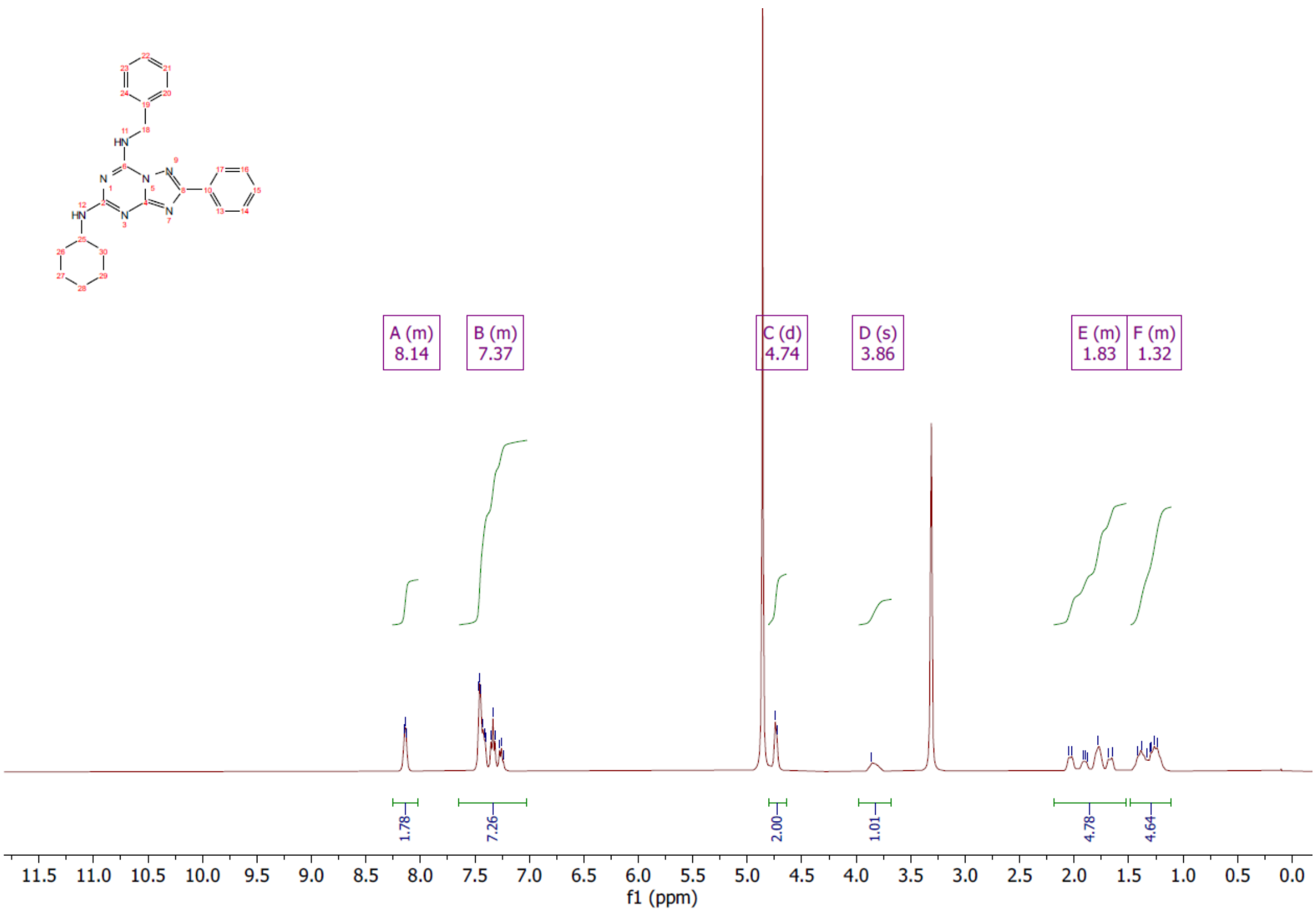

Figure S107. ^{1}H NMR spectrum of **Compound 26** (CD_3OD).

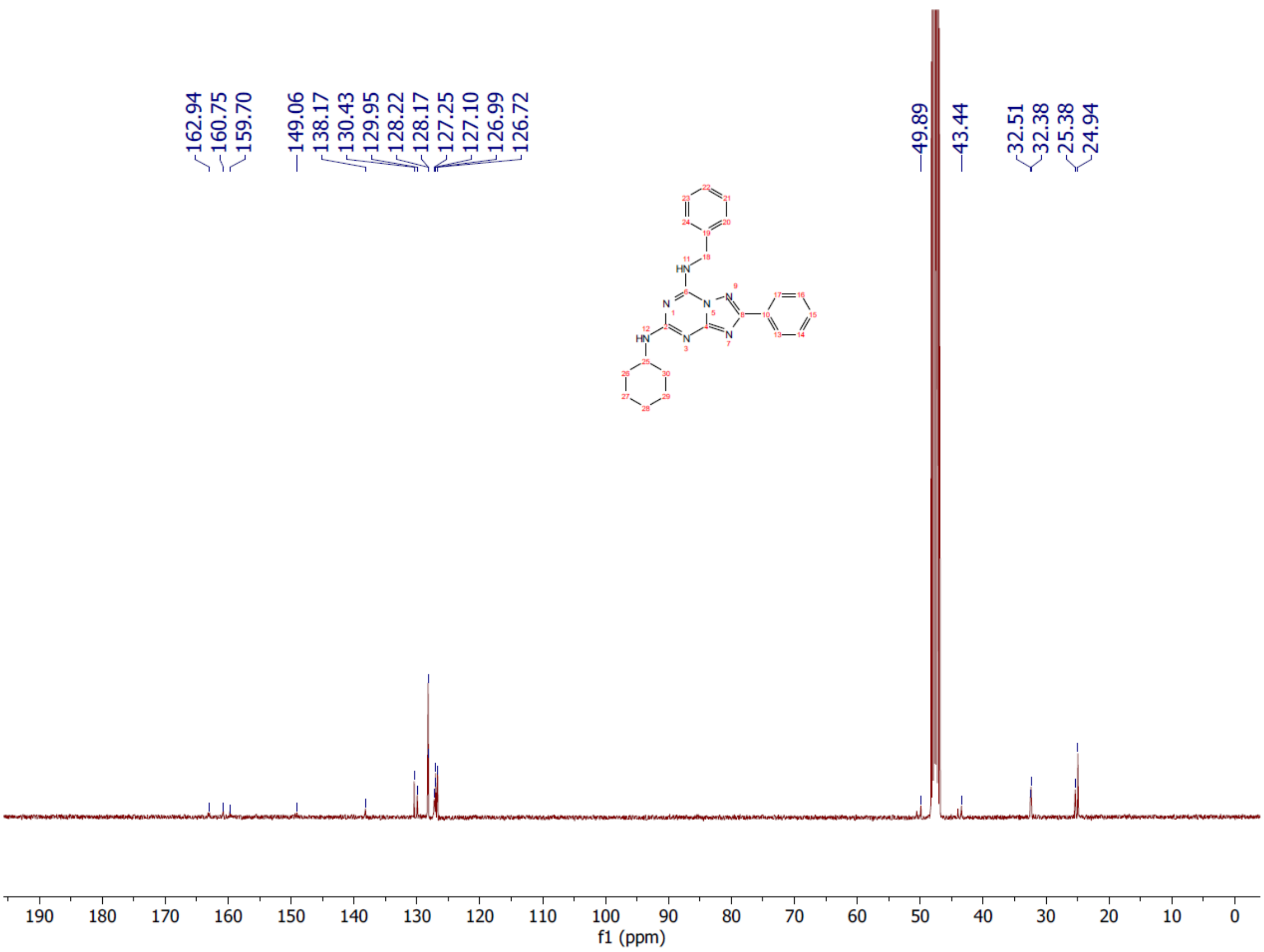

Figure S108. ^{13}C NMR spectrum of **Compound 26** (CD_3OD).

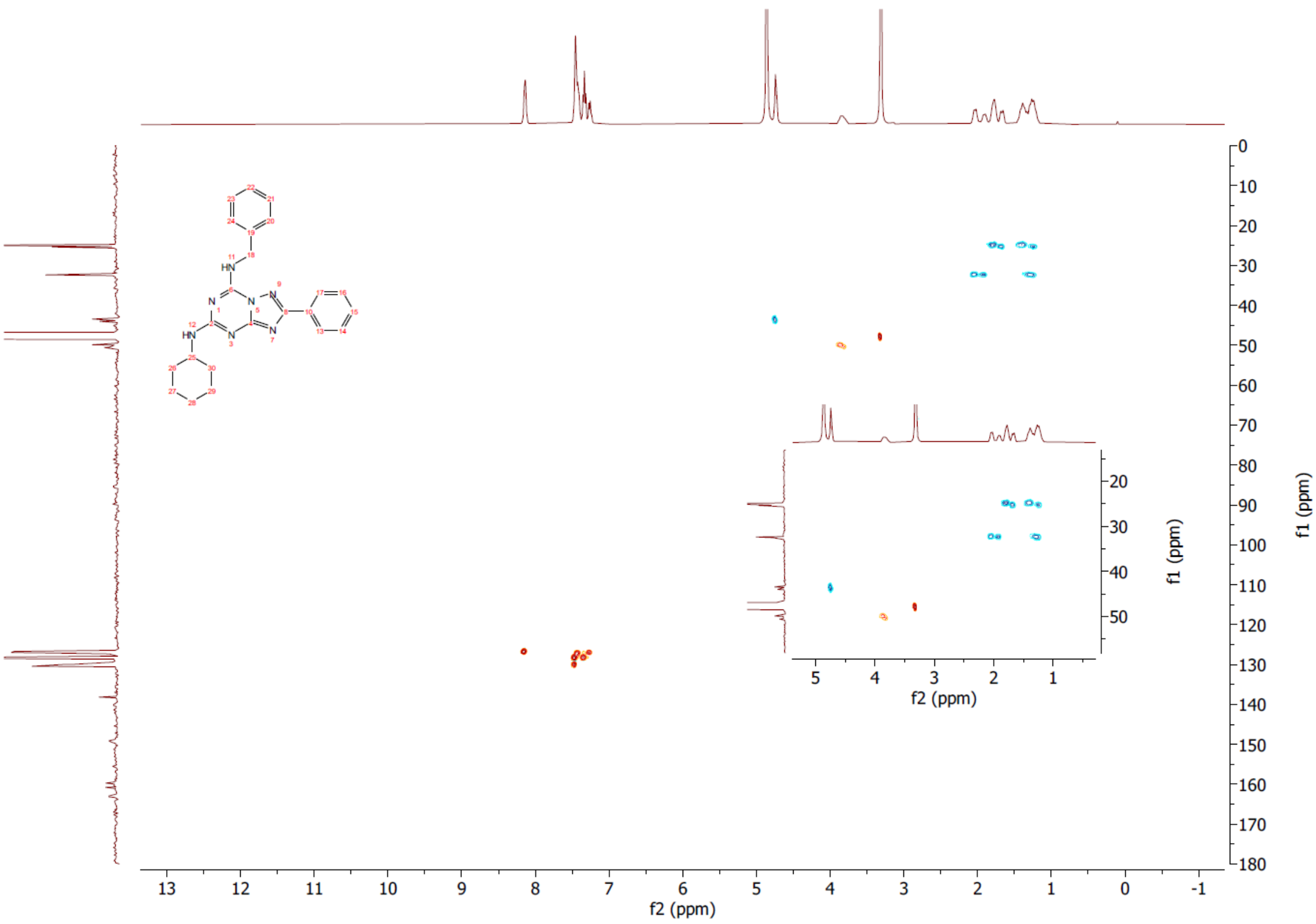

Figure S109. HSQC ^{1}H-^{13}C NMR spectrum of **Compound 26** (101 MHz, CD_3OD).

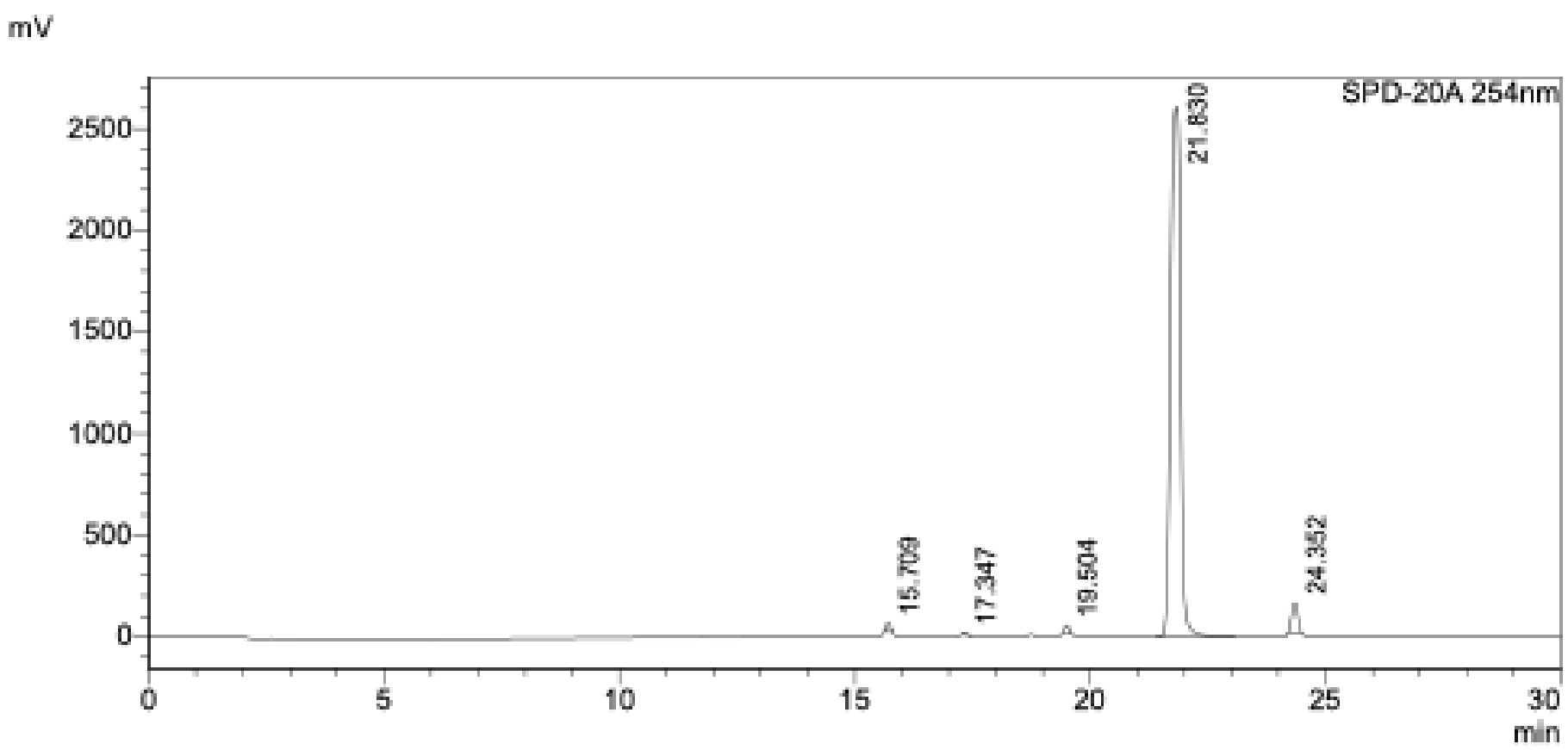

<Peak Table>

SPD-20A 254nm

Peak#	Ret. Time	Area	Height	Area%
1	15.709	245518	41086	0.648
2	17.347	37464	7164	0.099
3	19.504	207329	32704	0.547
4	21.830	36037740	2607666	95.072
5	24.352	1377681	151235	3.634
Total		37905733	2839856	100.000

Figure S110. HPLC Chromatogram of **Compound 26**.

N5,N7-dibenzyl-2-phenyl-[1,2,4]triazolo[1,5-*a*][1,3,5]triazine-5,7-diamine **27**

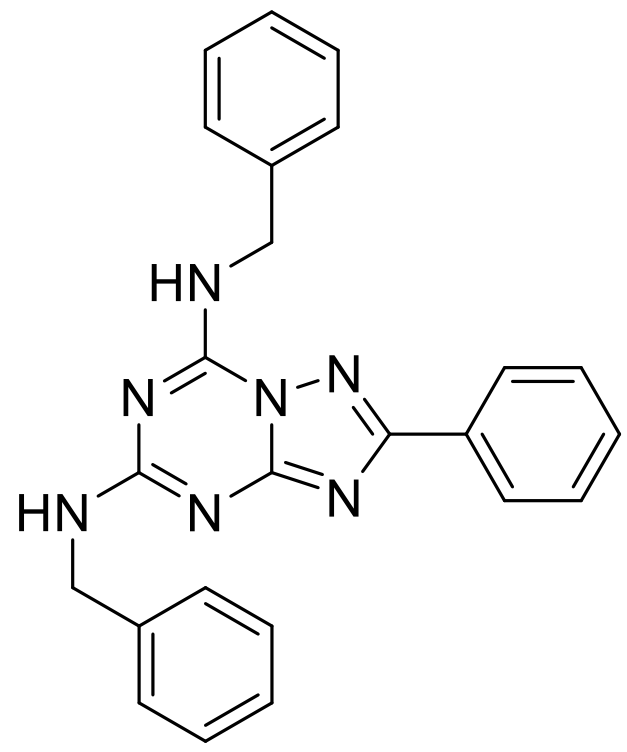

Chemical Formula: $C_{24}H_{21}N_7$
Molecular Weight: 407,47

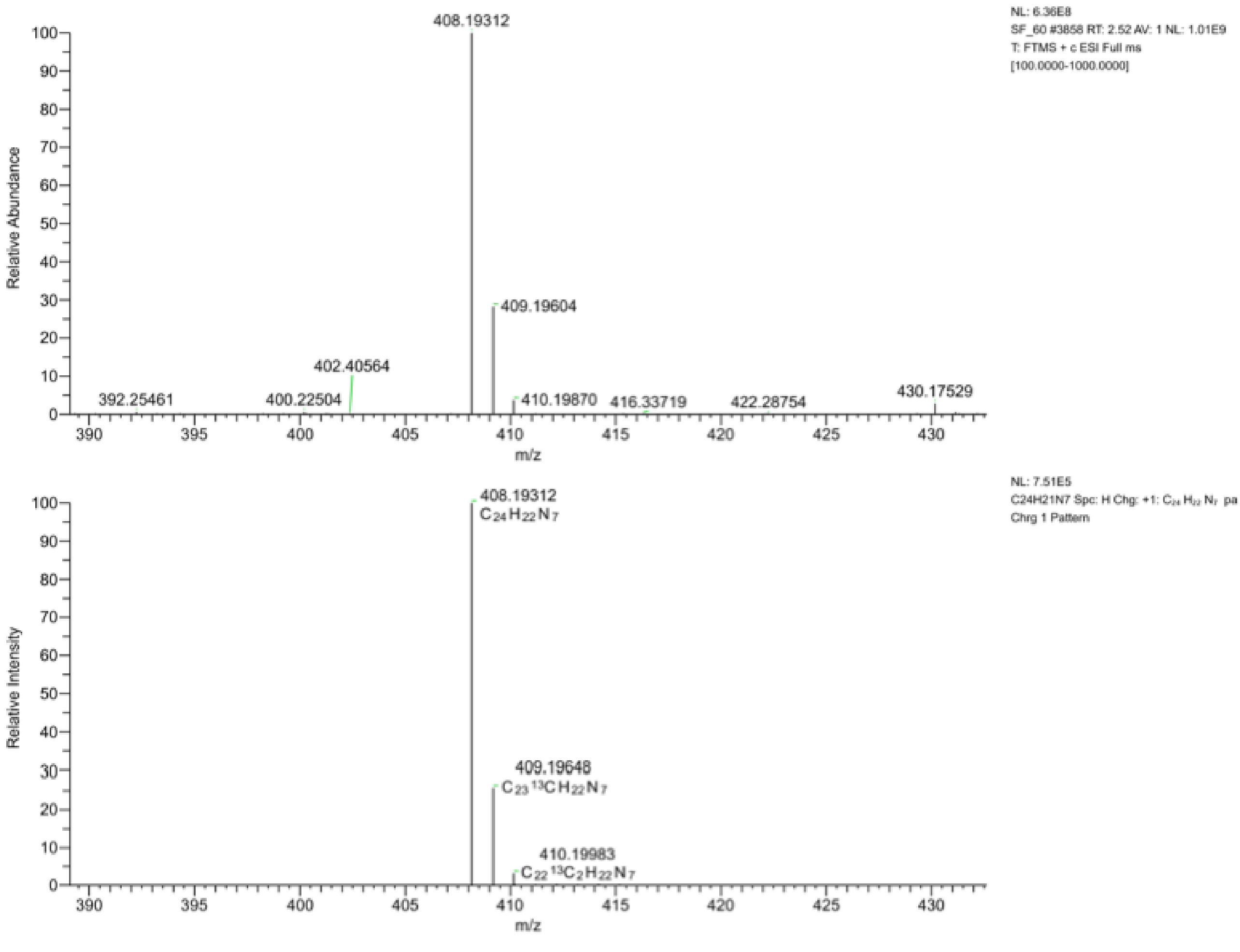

Figure S111. HRMS (ESI) spectrum of **Compound 27**.

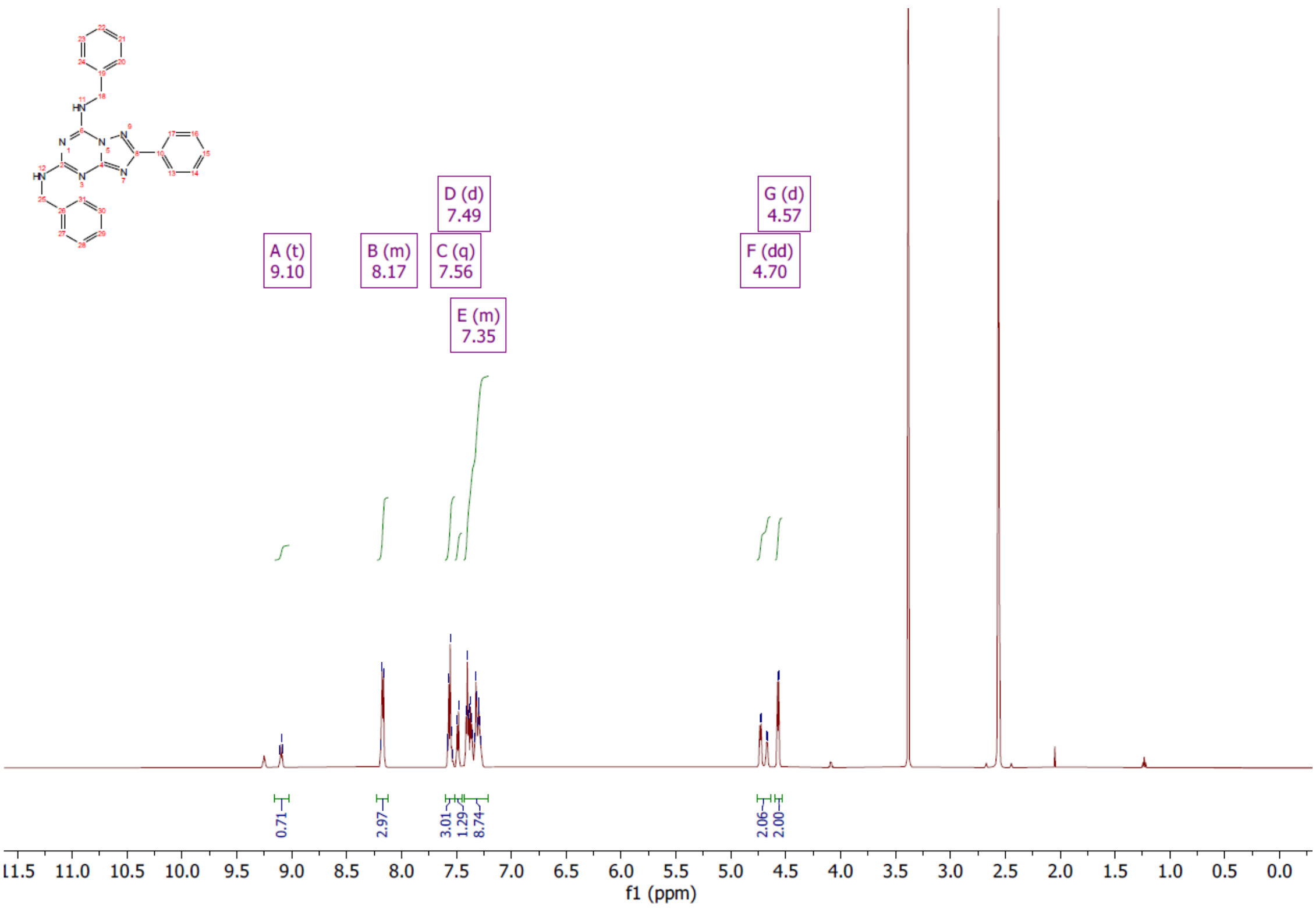

Figure S112. ^{1}H NMR spectrum of **Compound 27** (DMSO-d_6).

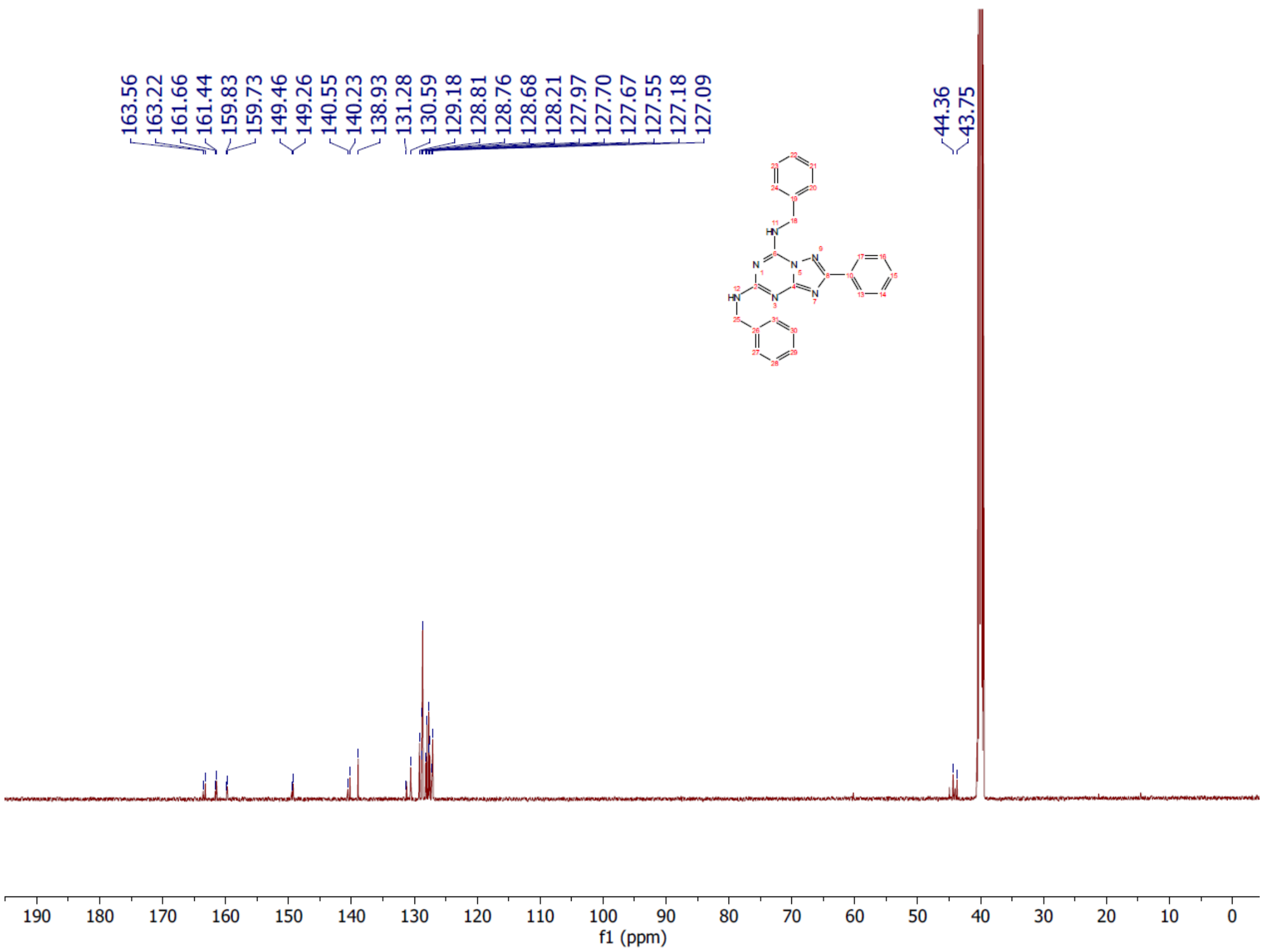

Figure S113. ^{13}C NMR spectrum of **Compound 27** (DMSO-d_6).

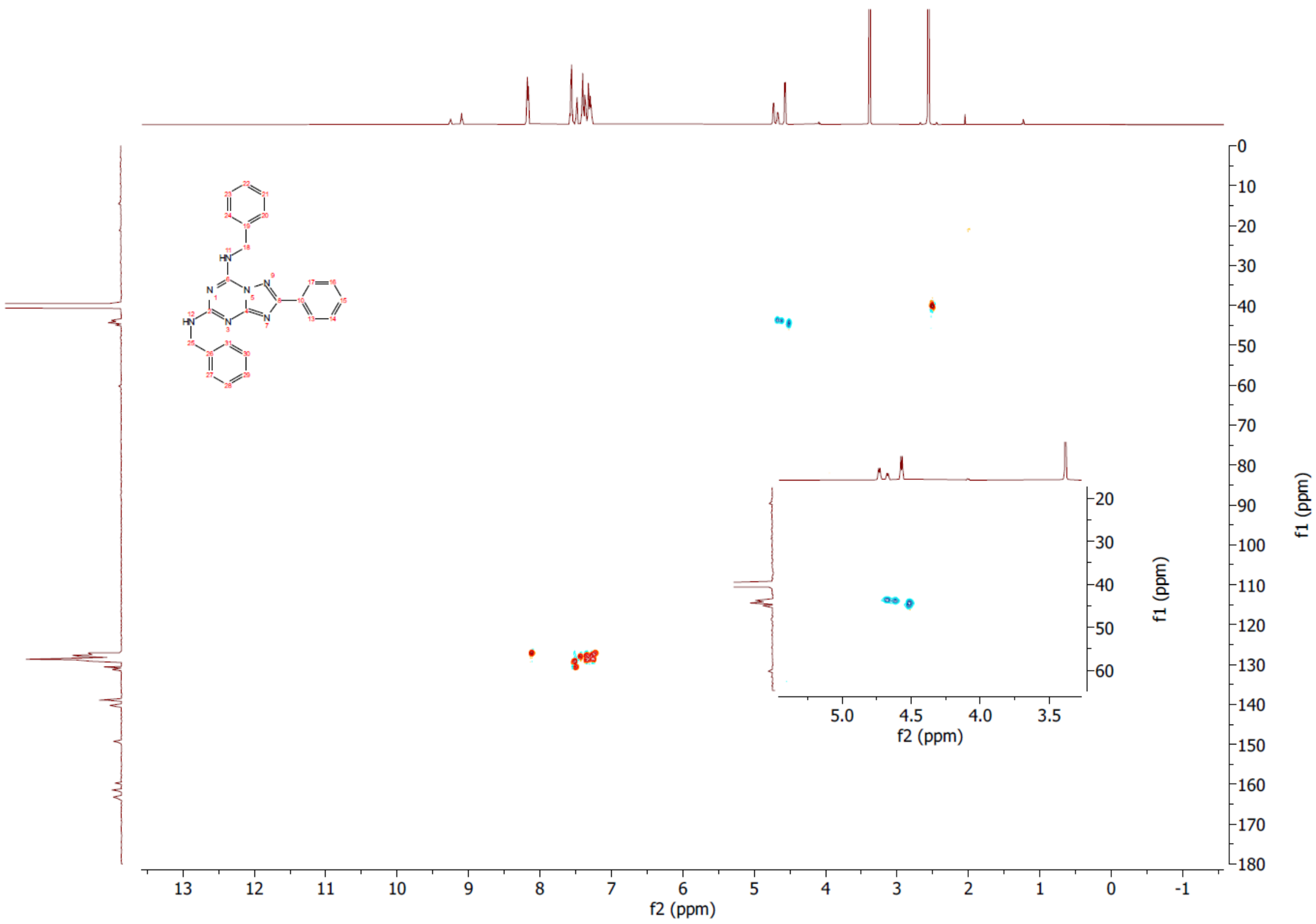

Figure S114. HSQC ^{1}H-^{13}C NMR spectrum of **Compound 27** (DMSO-d_6).

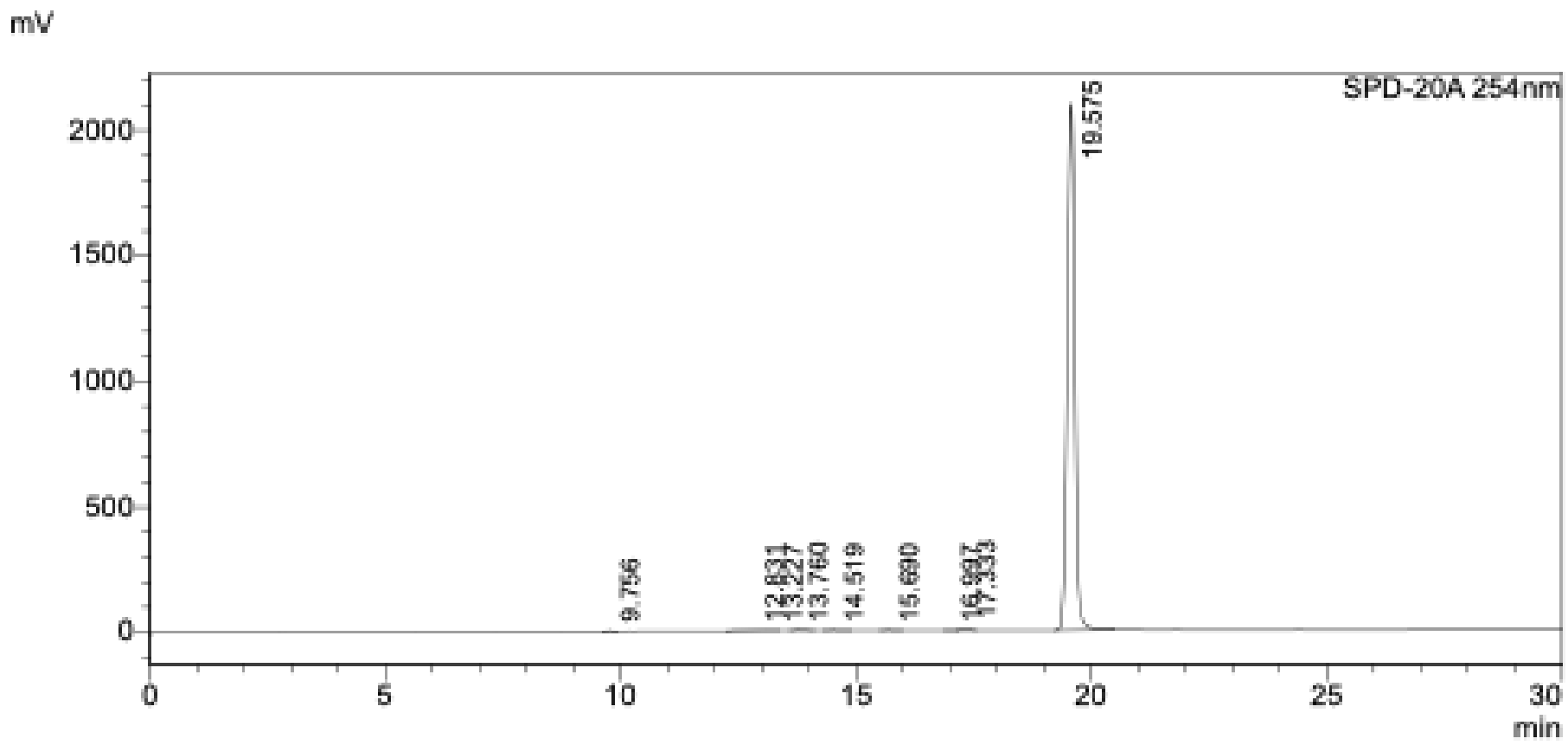

<Peak Table>

SPD-20A 254nm

Peak#	Ret. Time	Area	Height	Area%
1	9.756	22374	3369	0.094
2	12.831	25161	890	0.106
3	13.227	8167	1056	0.034
4	13.760	46674	3063	0.196
5	14.519	24175	1636	0.102
6	15.690	16753	1738	0.070
7	16.997	3218	594	0.014
8	17.333	108390	10291	0.455
9	19.575	23550521	2098873	98.929
Total		23805432	2121510	100.000

Figure S115. HPLC Chromatogram of **Compound 27.**

N^7-benzyl-N^5-isopropyl-2-(4-methoxyphenyl)-[1,2,4]triazolo[1,5-*a*][1,3,5]triazine-5,7-diamine **29**

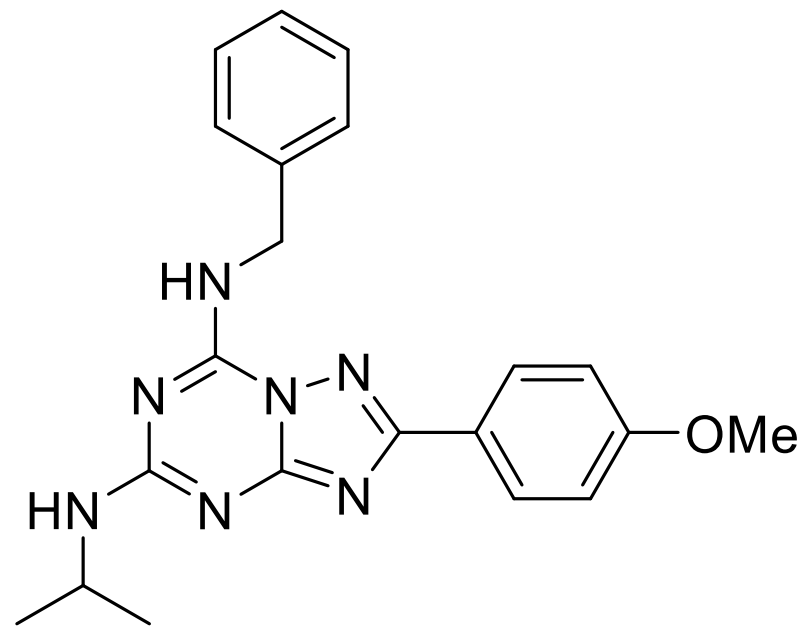

Chemical Formula: $C_{21}H_{23}N_7O$
Molecular Weight: 389,45

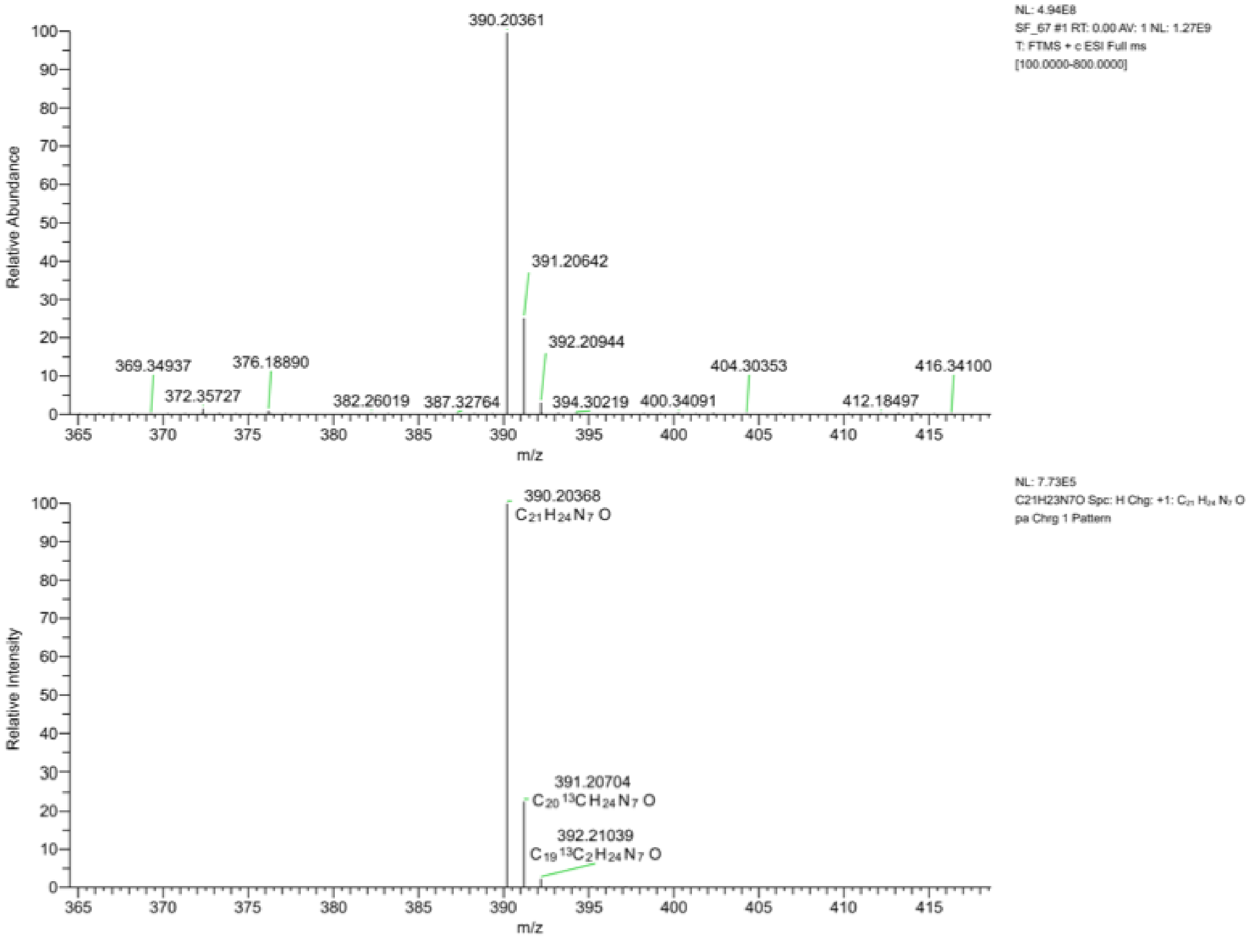

Figure S116. HRMS (ESI) spectrum of **Compound 29**.

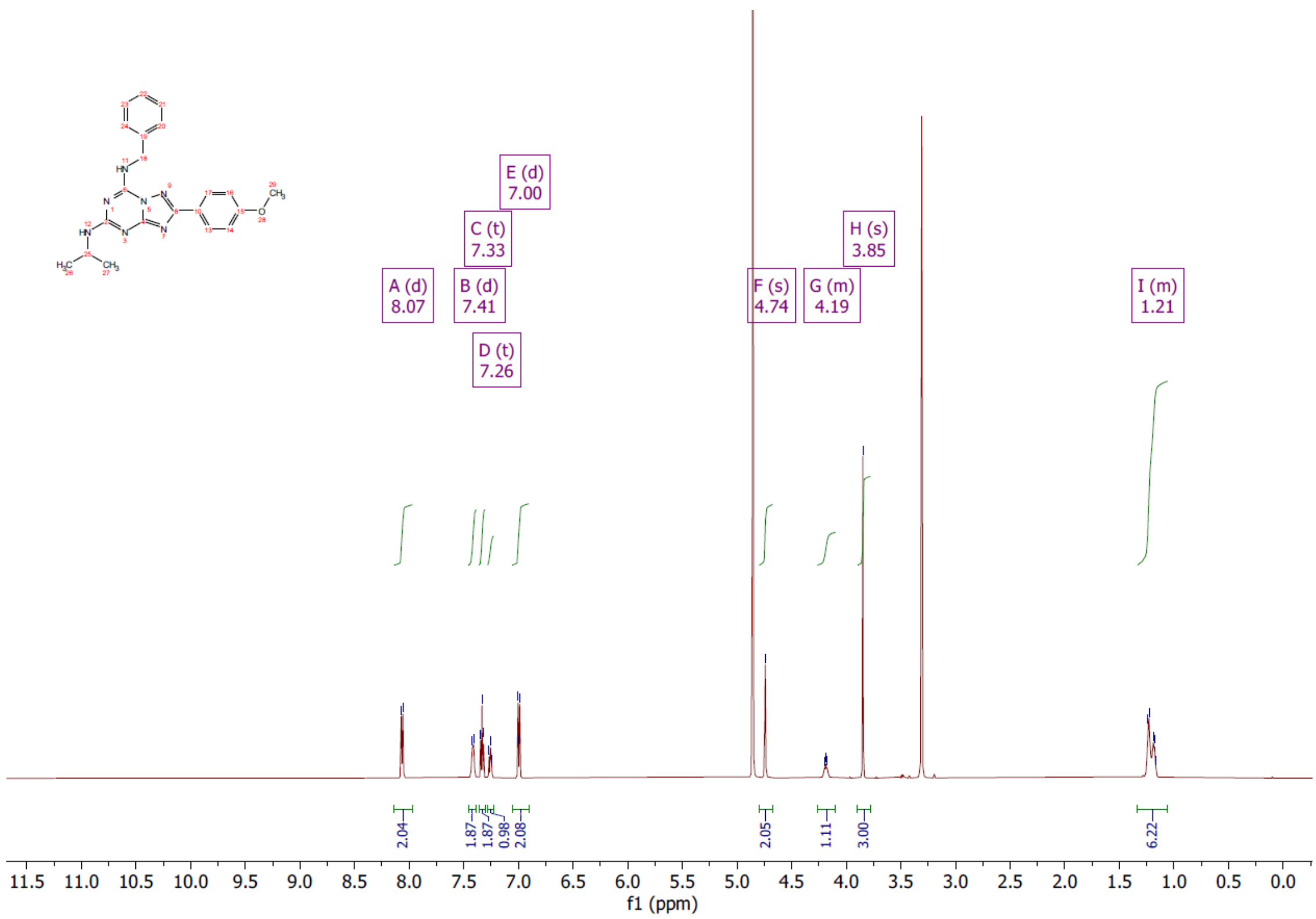

Figure S117. ^{1}H NMR spectrum of **Compound 29** (CD_3OD).

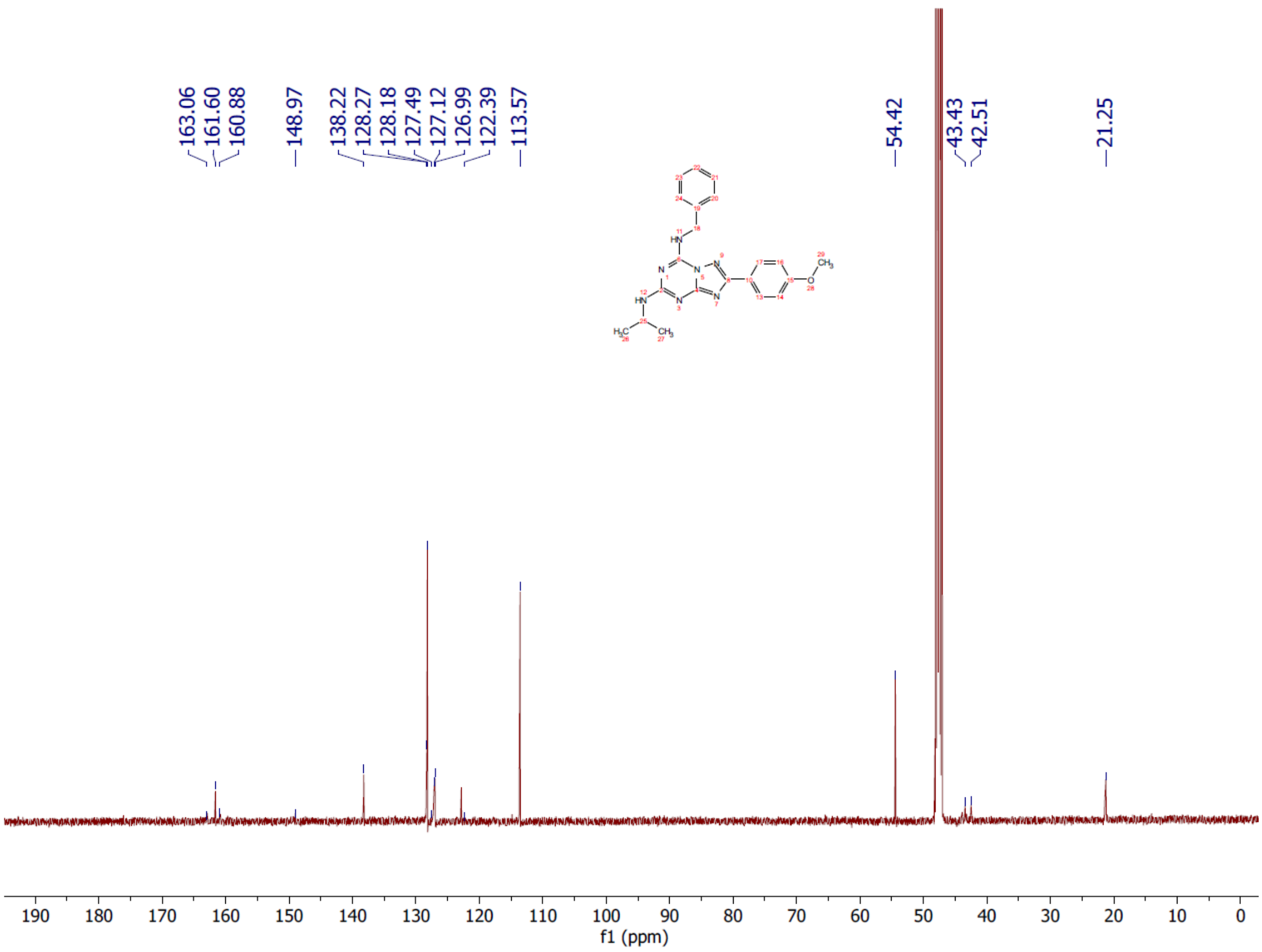

Figure S118. ^{13}C NMR spectrum of **Compound 29** (CD_3OD).

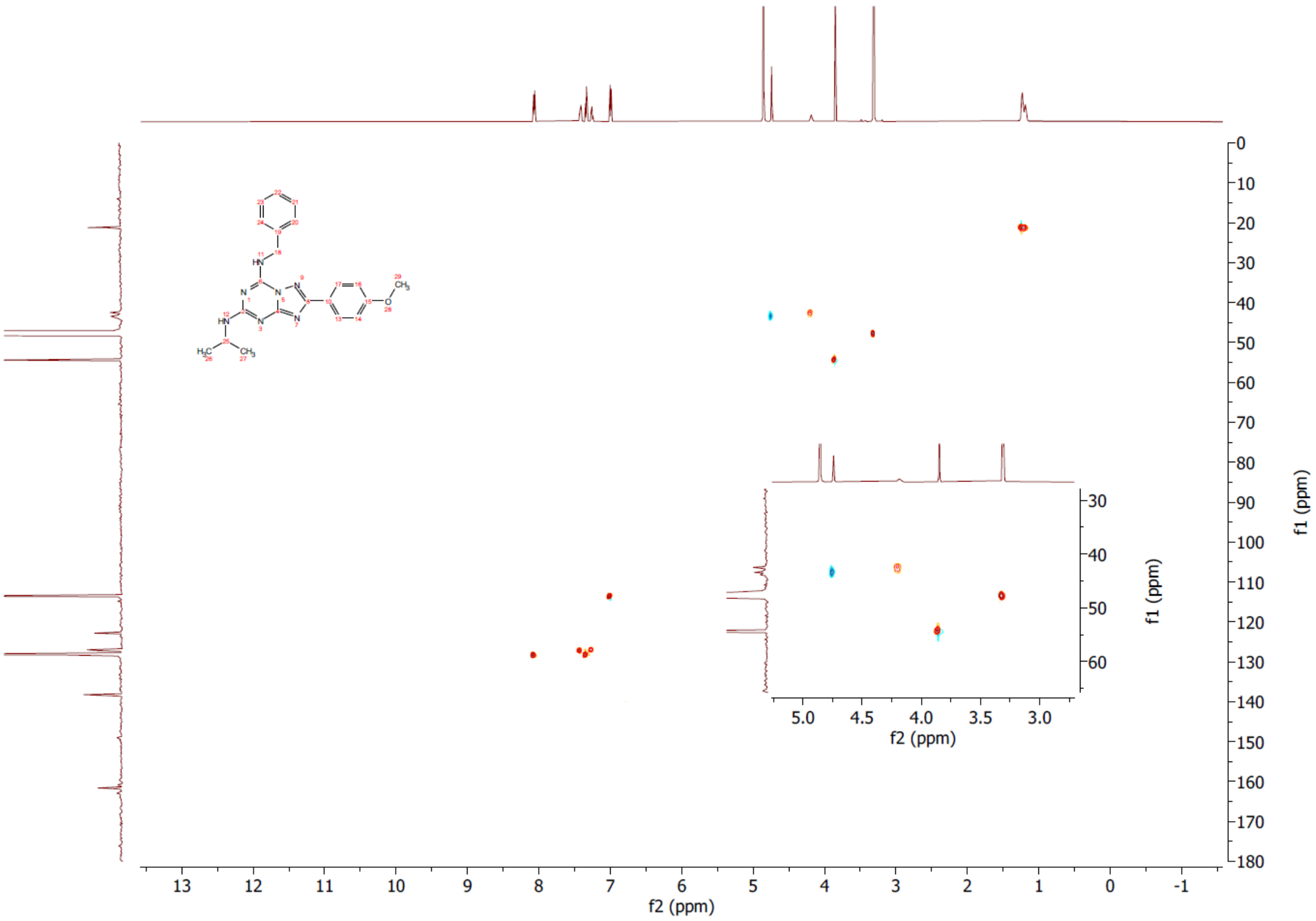

Figure S119. HSQC ^{1}H-^{13}C NMR spectrum of **Compound 29** (CD_3OD).

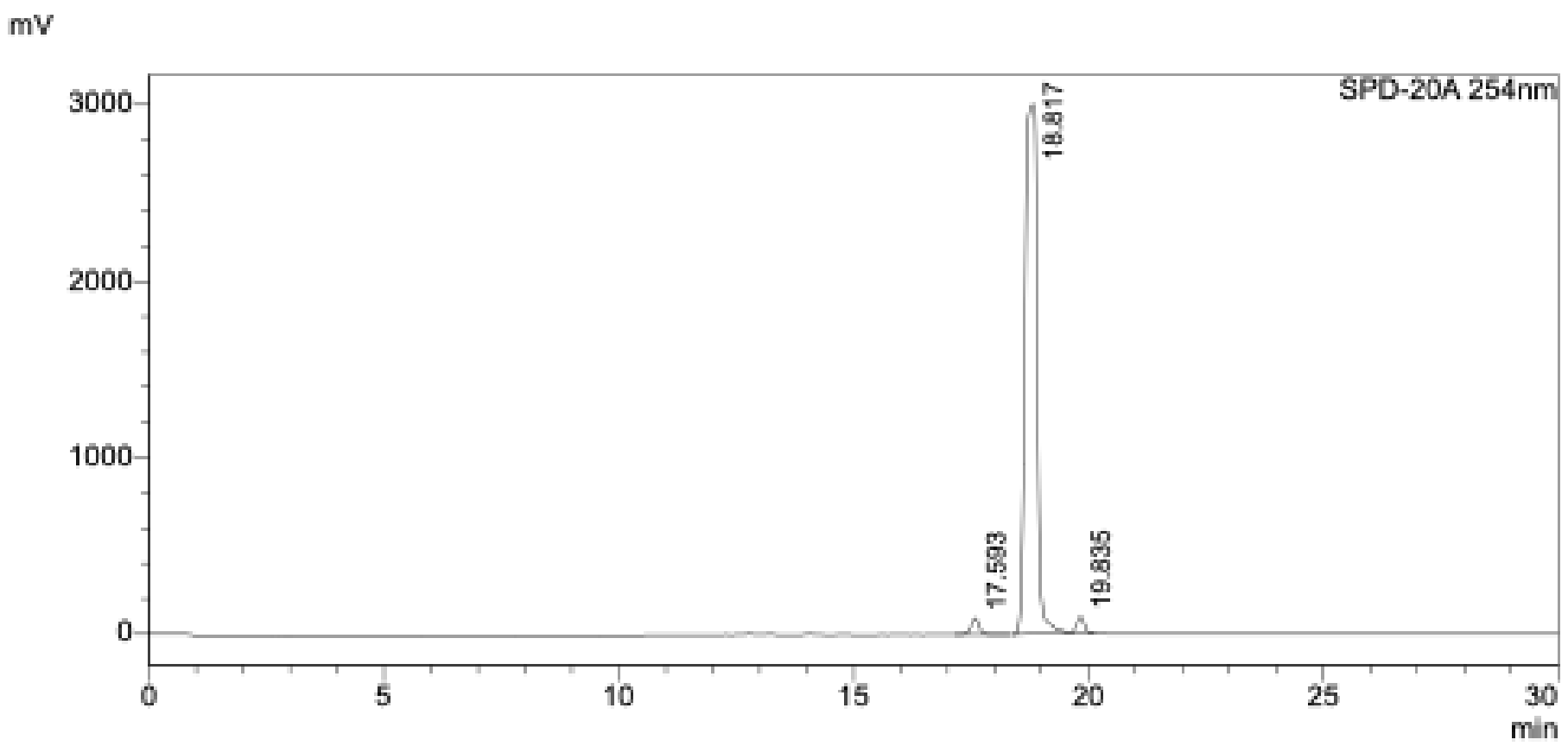

<Peak Table>

SPD-20A 254nm

Peak#	Ret. Time	Area	Height	Area%
1	17.593	1063607	92352	1.916
2	18.817	53486572	2999741	96.369
3	19.835	951798	89436	1.715
Total		55501977	3181529	100.000

Figure S120. HPLC Chromatogram of **Compound 29.**

N^5,N^7-dibenzyl-2-(4-methoxyphenyl)-[1,2,4]triazolo[1,5-*a*][1,3,5]triazine-5,7-diamine **30**

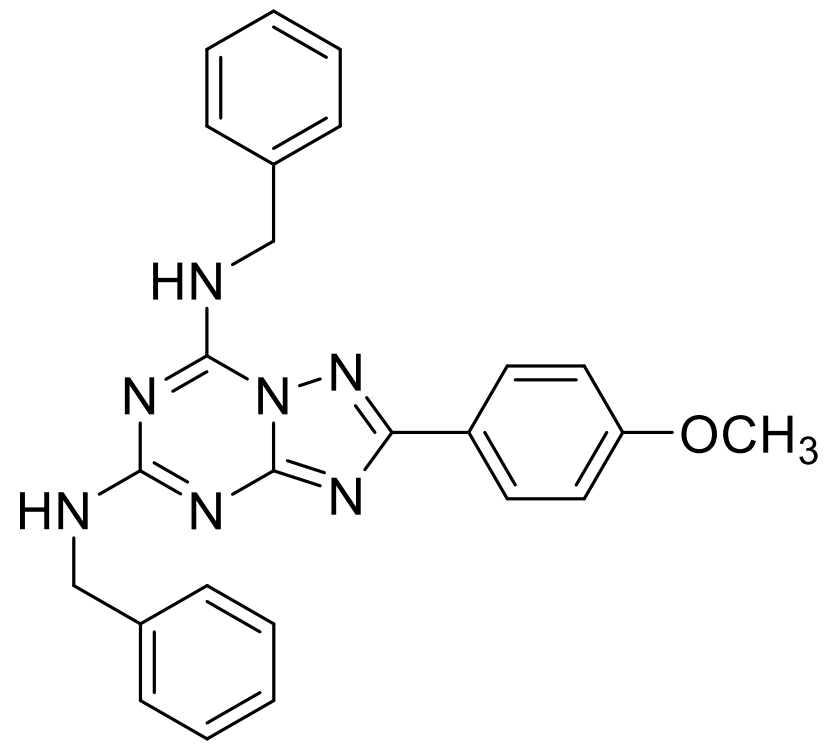

Chemical Formula: $C_{25}H_{23}N_7O$
Molecular Weight: 437,50

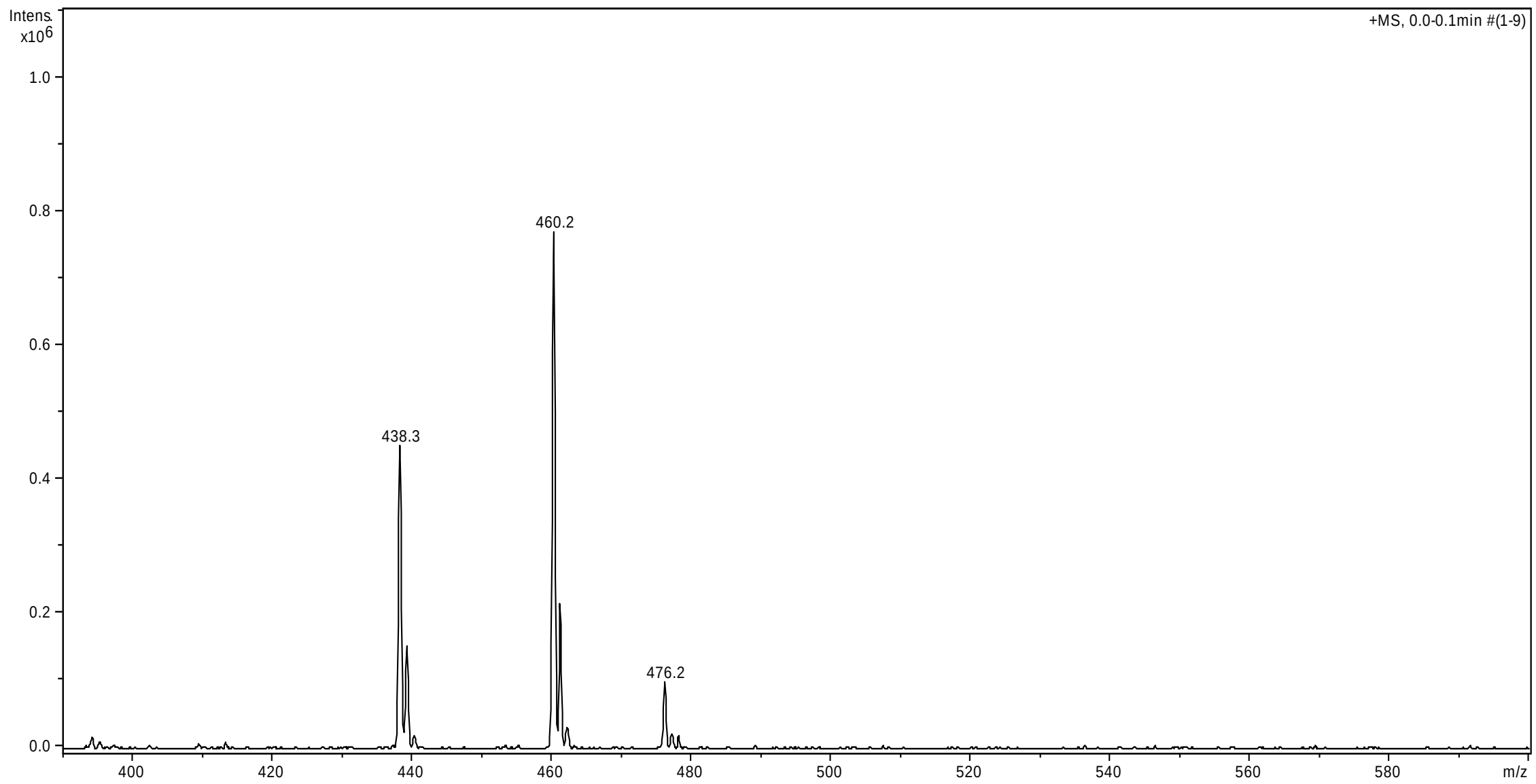

Figure S121. ESI-MS spectrum of **Compound 30**.

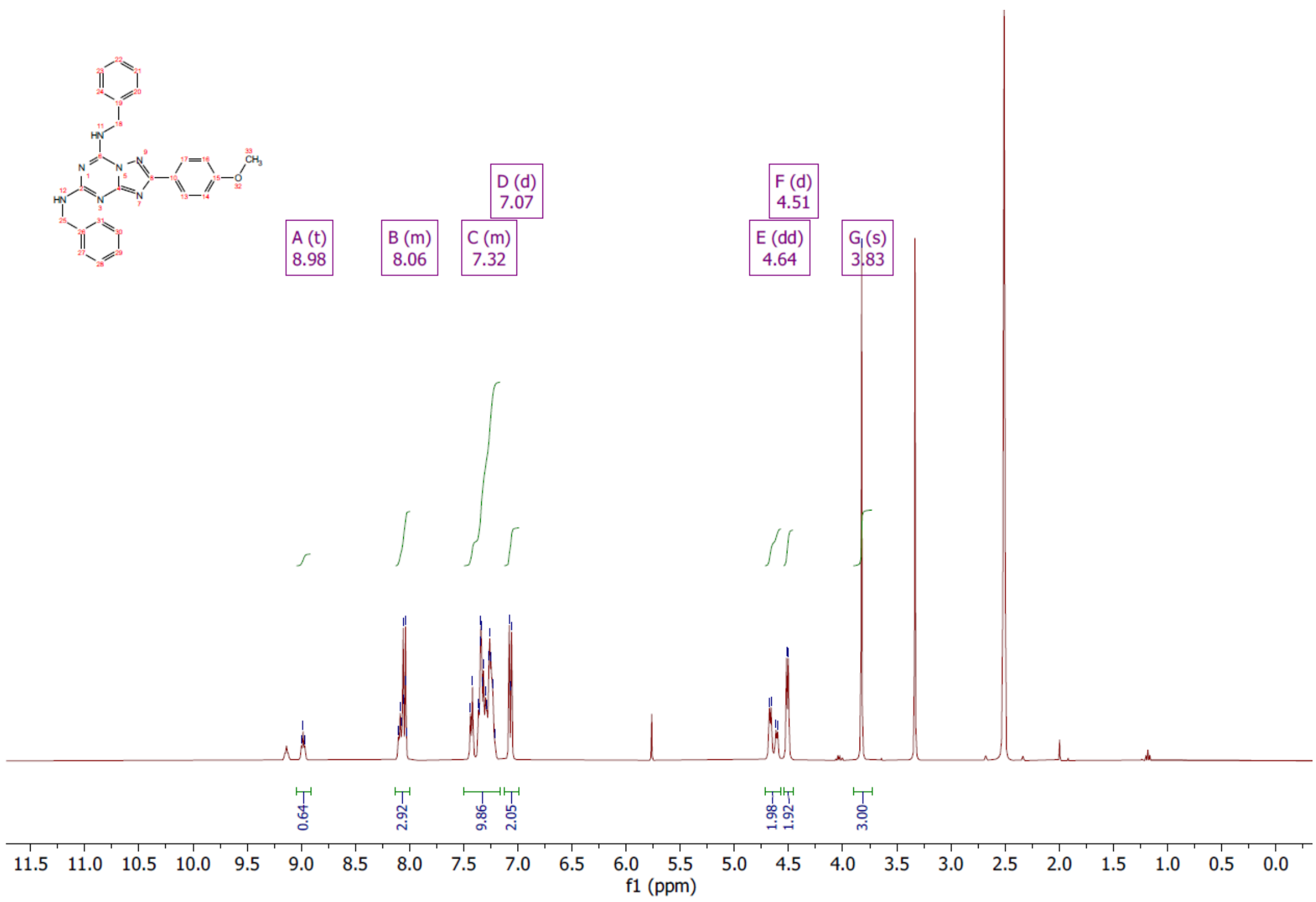

Figure S122. ^{1}H NMR spectrum of **Compound 30** (DMSO-d_6).

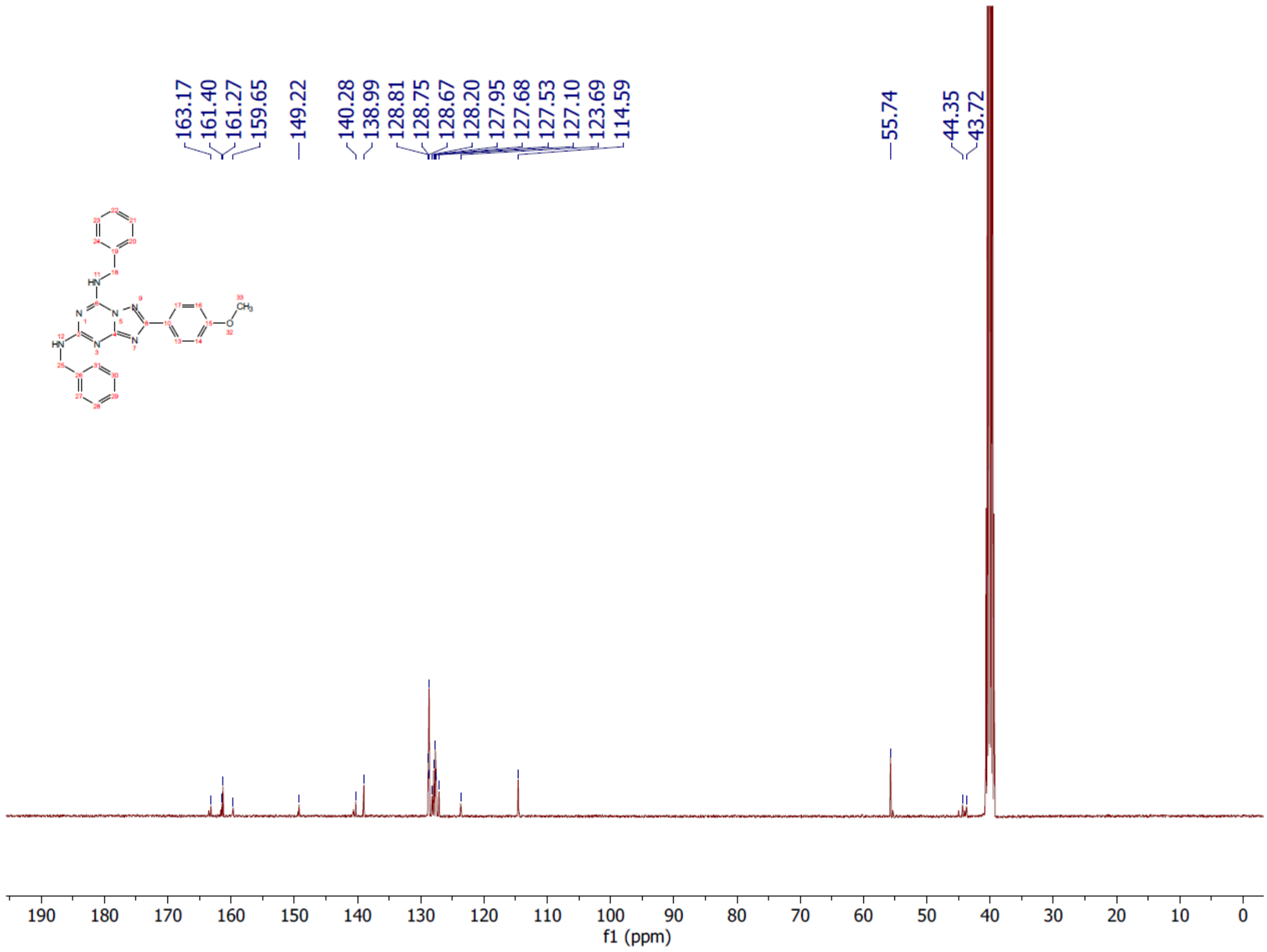

Figure S123. ^{13}C NMR spectrum of **Compound 30** (DMSO-d_6).

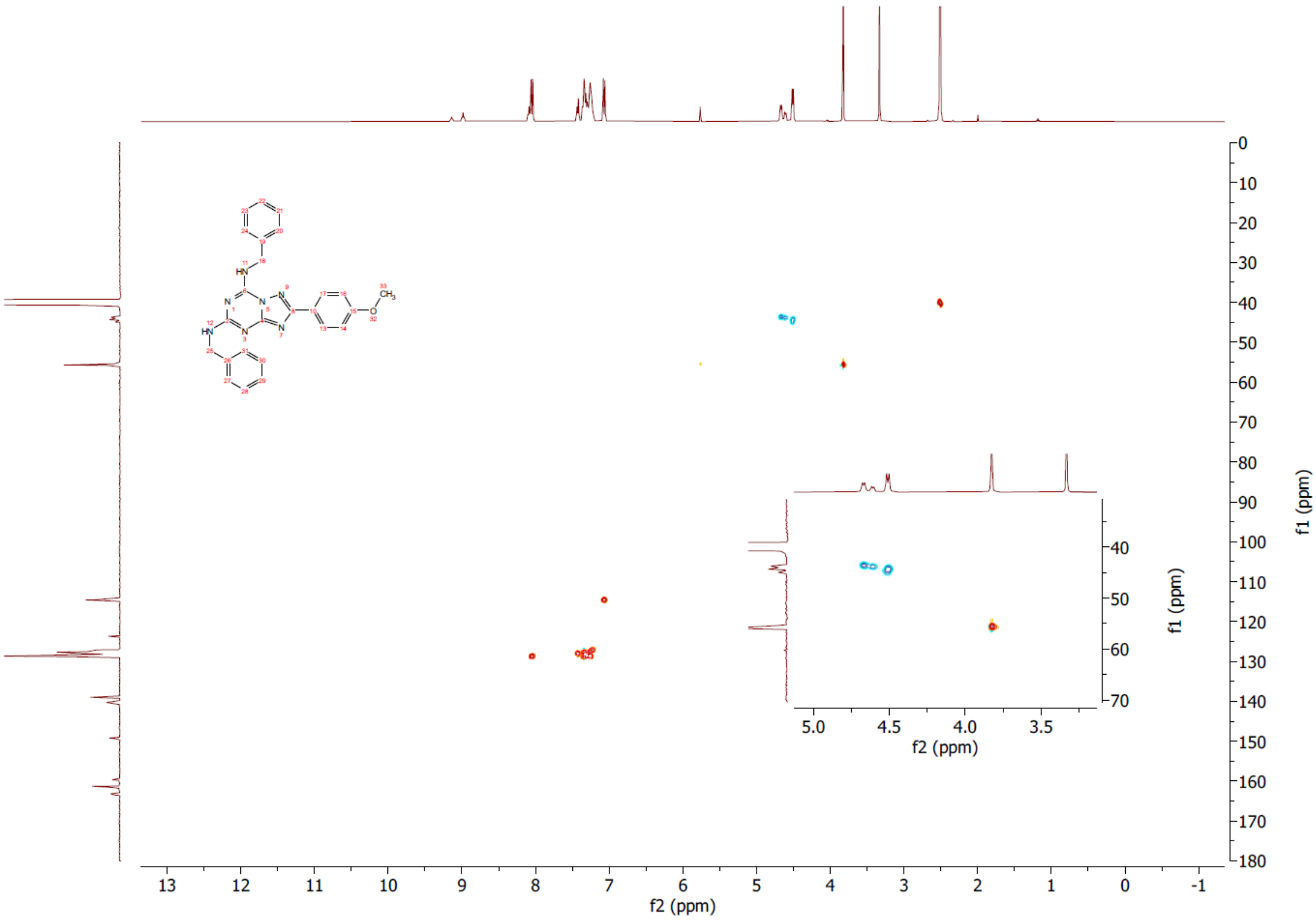

Figure S124. HSQC ^{1}H-^{13}C NMR spectrum of **Compound 30** (DMSO-d_6).

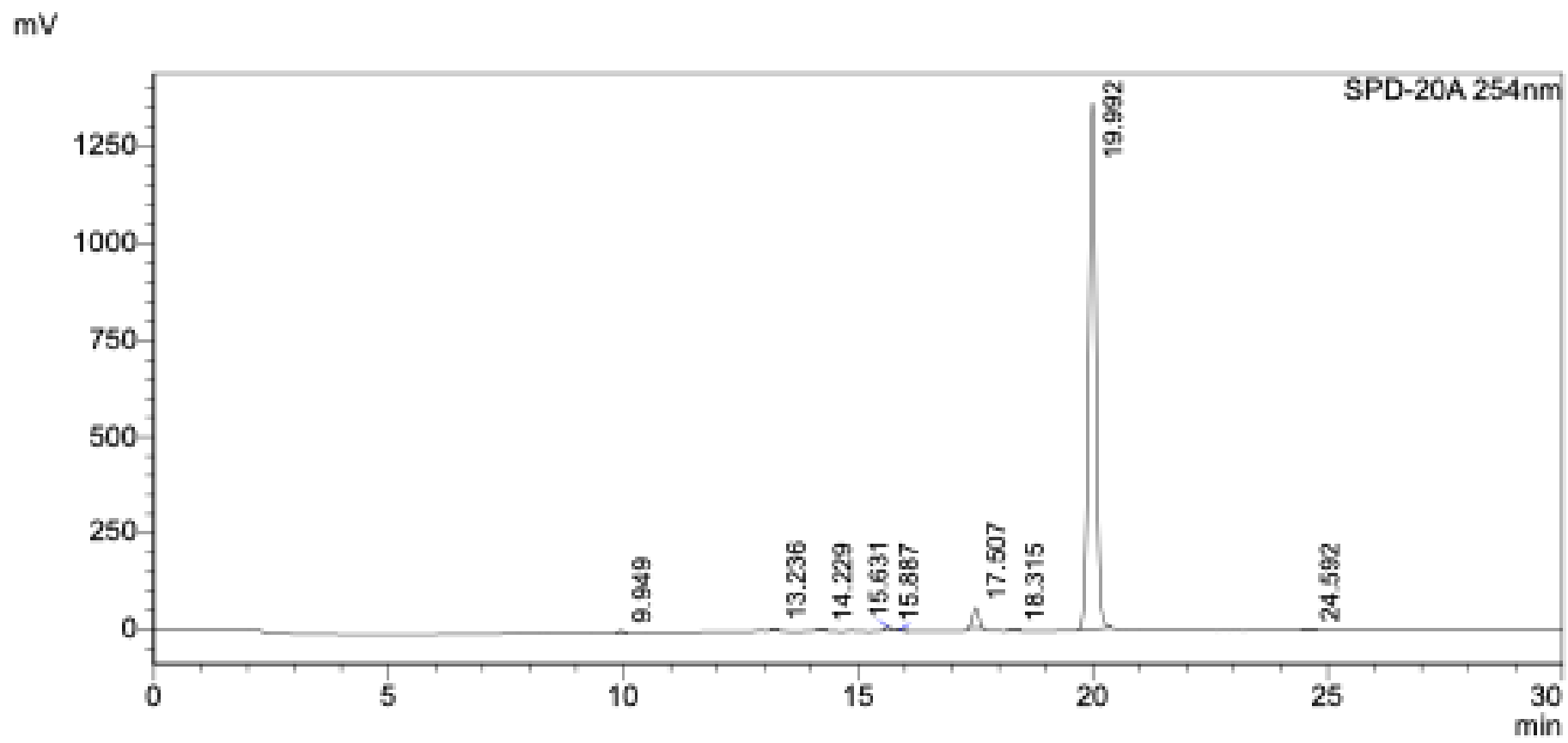

<Peak Table>

SPD-20A 254nm

Peak#	Ret. Time	Area	Height	Area%
1	9.949	21979	3197	0.141
2	13.236	31811	4909	0.204
3	14.229	24214	3400	0.156
4	15.631	59319	8423	0.381
5	15.887	14866	2184	0.096
6	17.507	479875	51822	3.084
7	18.315	20153	2331	0.130
8	19.992	14896998	1359475	95.734
9	24.592	11585	1297	0.074
Total		15560800	1437039	100.000

Figure S125. HPLC Chromatogram of **Compound 30.**
